# Some coagulase negative *Staphylococcus* spp. isolated from buffalo can be misidentified as *Staphylococcus aureus* by phenotypic and Sa442 PCR methods

**DOI:** 10.1186/s13104-018-3449-8

**Published:** 2018-05-30

**Authors:** Camila C. de Almeida, Lucas J. L. Pizauro, Glenn A. Soltes, Durda Slavic, Fernando A. de Ávila, João M. Pizauro, Janet I. MacInnes

**Affiliations:** 1 Agriculture and Livestock Microbiology Graduation Program, Department of Veterinary Pathology, São Paulo State University (Unesp), School of Agricultural and Veterinarian Sciences, Jaboticabal, Brazil; 2 Department of Veterinary Preventive Medicine and Animal Reproduction, São Paulo State University (Unesp), School of Agricultural and Veterinarian Sciences, Jaboticabal, Brazil; 3 Department of Pathobiology, University of Guelph, 50 Stone Rd. East, Guelph, ON N1G 2W1 Canada; 4 Animal Health Laboratory, University of Guelph, Post Office 3612, Guelph, ON N1H 6R8 Canada; 5 Department of Technology, São Paulo State University (Unesp), School of Agricultural and Veterinarian Sciences, Jaboticabal, Brazil

**Keywords:** Mastitis, *Staphylococcus aureus*, Species-specific PCR tests, *cydB* PCR

## Abstract

**Objective:**

*Staphylococcus aureus* is a commonly reported cause of buffalo mastitis. However, its prevalence may be overestimated. The aim of this study was to compare *S. aureus* identification by conventional phenotypic and genotypic assays versus Matrix-Assisted Laser Desorption Ionization-Time of Flight Mass Spectrometry (MALDI-TOF MS) and novel real-time quantitative PCR tests for the cytochrome oxidase subunit D II *(cyd*B) and staphylocoagulase (*coa*) genes.

**Results:**

From 408 samples obtained from buffalo milk/milking environment, 32 putative *S. aureus* strains were identified based on characteristic growth on Baird Parker agar, positive catalase reaction, ability to clot rabbit plasma, and positive Sa442 PCR assay. However, in further testing, only 10 of these strains were positive in latex agglutination tests and by MALDI-TOF MS, only eight of the 32 strains were *S. aureus* while the rest were *S. chromogenes* (19), *S. agnetis* (3), *S. cohnii* (1), or *S. xylosus* (1). All eight strains identified as *S. aureus* by MALDI-TOF analysis and confirmed by 16S RNA gene sequencing were positive in a *S. aureus*-specific *cydB* PCR test. As well, 7/8 *S. aureus* strains were PCR positive in a real-time *coa* PCR test as were 2/69 *S. chromogenes* and the lone *S. xylosus* strain tested.

**Electronic supplementary material:**

The online version of this article (10.1186/s13104-018-3449-8) contains supplementary material, which is available to authorized users.

## Introduction

Buffalo milk and its derivatives have become increasingly important worldwide [1] and *Staphylococcus aureus* is one of the most significant pathogens responsible for contagious mastitis in dairy buffaloes [2]. Antibiotic treatment of *S. aureus* mastitis is often unsuccessful and treatment failures can lead to spread of the infection. As a result, animals with chronic *S. aureus* infection are often culled [3].

The initial identification of *S. aureus* is based on culture and phenotype on specific media; other assays commonly used to identify *S. aureus* are the Sa442 PCR, *nuc* gene PCR, and latex agglutination tests. The Sa442 PCR test, developed by Martineau et al. [4] targets a chromosomal DNA fragment thought to be specific for *S. aureus,* the *nuc* gene encodes a species-specific thermonuclease, while commercially available latex agglutination kits such as the Staphaurex latex test are based on the interaction of *S. aureus* surface-anchored proteins with human IgG and fibrinogen bound to latex particles [5]. However, these tests may not be accurate and can lead to erroneous identification, and in turn, to unnecessary culling [5, 6]. Here we describe how common testing approaches can lead to misidentification of “non-*S. aureus*” strains as *S. aureus* and the development of new a *cydB* real-time PCR assay that can be used for accurate *S. aureus* identification.

## Main text

### Methods

#### Sample collection

Milk samples (n = 320) were collected from 80 randomly selected female buffaloes from a private dairy farm located in Sao Paulo State, Brazil from November 2013 to April 2014. After physical examination of the mammary glands [7], teats were cleaned with 70% alcohol and milk from each quarter was evaluated by strip cup and California mastitis tests [8]. Hand samples from 16 consenting milkers and 64 samples from liners were collected using sterile swabs (Pro-Lab Diagnostics) and stored in peptone water as described previously [9].

#### CoPS isolation and identification

Isolation and identification of *S. aureus* was done according to compendium of methods for the microbiological examination of foods [10]. Strains with positive egg yolk reactions [11] were tested for Gram and catalase reactions, haemolytic activity, and ability to clot rabbit plasma using a Coagu-Plasma kit (Laborclin, Pinhais, Brazil) according to manufacturer’s instructions with *S. aureus* ATCC 25293 and *S. epidermidis* ATCC 12228 as the positive and negative controls respectively. In addition, isolates were tested with the Staphyclin latex test (Laborclin, Pinhais, Brazil) according to the manufacturer’s instructions.

#### DNA extraction

For DNA extraction, well-isolated colonies were inoculated into BHI broth, incubated at 37 °C for 18 h and extracted according to the method of Kuramae-Izioka [12] with minor modifications as described previously [13].

#### Sa442 detection

Isolates were characterized using the PCR assay of Martineau et al. [4] with minor modifications. PCR primers (Table 1) were used at 10 pmol/µL in 25 µL reaction mixtures with 50 ng/µL of DNA and 20 µL LightCycler^®^ 480 SYBER Green Master mix (Roche Diagnostics, Indianapolis, IN). Amplification parameters were: one cycle at 95 °C for 10 min, followed by 40 cycles at 95 °C for 10 s, 55 °C for 20 s, and 72 °C for 12 s in a Roche LightCycler^®^ 480 (LC480) thermocycler. The ramp rates were 4.4, 2.2, and 4.4 °C/s, respectively. *Staphylococcus chromogenes* and *Streptococcus suis* DNAs and water were used in negative control reactions and *S. aureus* strains COL, NewMan, MW2, Mu50 and ATCC 25923 were used as positive controls. A melting step (62 °C) was done to confirm single product.

**Table 1:** Sequence of primers used in PCR assays

“Gene”	Primer	Sequence (5′–3′)	Product (bp)	Reference
*coa*	Forward	GTCTTGAAAGTAGCTCATCTAAACTTG	228	This study
	Reverse	ATCCAAATGTTCCATCGTTGTATTC		
*cyd*-*aureus*	Forward	CCCATTTGCTTGGTCTGTAGTA	432	This study
	Reverse	GTCCAGCCCATTTCTGGATTA		
Sa442	Forward	AATCTTTGTCGGTACACGATATTCTTCACG	108	Martineau et al. [4]
	Reverse	CGTAATGAGATTTCAGTAGATAATACAACA		

#### Matrix-Assisted Laser Desorption Ionization-Time of Flight Mass Spectrometry (MALDI–TOF MS)

Identification of putative *Staphylococcus aureus* strains was done using a MALDI Bruker Biotyper system (Bruker Daltonics Inc., Billerica, MA, USA) at the Animal Health Laboratory, University of Guelph, Guelph, Ontario, Canada, as described previously [13].

#### 16S rRNA gene sequencing

16S rRNA gene sequencing (~ 1000 bp) was also done at the Animal Health Laboratory and sequences were compared with the 16S rRNA gene of *Staphylococcus aureus* MCRF184 (CP014791.1) and other *Staphylococcus* spp. sequences using blastn.

#### Design of cydB species-specific primers

PCR primers to the *S. aureus cydB* gene (cytochrome D ubiquinol oxidase subunit II; NCBI accession number NC_007795.1) were designed as described previously [13] and produced a 432 bp amplicon. The amplicons were confirmed by DNA sequencing as described above (Table 1). As well, an additional 84 putative staphylococci from buffalo milk/milking environment were evaluated using the *cydB* test [13].

#### Design of coagulase gene primers

Published *coa* primers [14] generate products of different sizes so a new primer pair (coaF and coaR; Table 1) was designed using PrimerQuest software (Integrated DNA technologies, Inc. http://www.idtdna.com) to the coagulase gene of *S. aureus* JCSC 7638 (AB488509.1). A conserved region of the gene was identified by aligning 103 strains in GenBank using CLC Sequence View 7 software (Additional file 1: Fig. S1).

#### Real-time PCR

Real-time PCR primers (Table 1) were used at 10 pmol/µL in 25 µL reaction mixtures containing 50 ng/µL of DNA and 20 µL Light Cycler 480 (LC480) SYBER Green Master mix (Roche Diagnostics, Indianapolis, IN). For *cydB* and *coa* gene amplifications the parameters were: one cycle at 95 °C for 10 min, followed by 40 cycles at 95 °C for 10 s, 55 °C for 20 s, and 72 °C for 25 s in a Roche Light Cycler 480 (LC480). Ramp rates were 4.4, 2.2, and 4.4 °C/s, respectively; *Streptococcus suis* DNA and water were used as negative controls. In addition, a melting step (62 °C) was done to confirm a single product. *S. aureus* strains COL, NewMan, MW2, Mu50 and ATCC 25923 were used as positive controls. All real-time PCR were performed in triplicate.

### Results

#### *Staphylococcus* spp. isolation and preliminary identification

Thirty-two putative *S. aureus* strains were selected based on their characteristic phenotype on Baird Parker agar. These Gram positive strains were catalase positive and were positive in the *S. aureus* species specific Sa442 PCR assay of Martineau et al. [4]. In further testing, 24 samples were consistently positive in the coagulase test; while eight gave at least one discordant result. Also, 21 of the 32 putative *S. aureus* strains were β-haemolytic, two were α-haemolytic and nine were non-haemolytic.

#### Latex test, identification by MALDI-TOF and 16S rRNA sequencing

Only ten of the 32 strains gave a positive latex agglutination result and MALDI-TOF MS analysis revealed that only eight of the 32 were *S. aureus* with the remainder being *S. chromogenes* (n = 19), *S. agnetis* (n = 3), *S. xylosus* (n = 1), or *S. cohnii* (n = 1) (Table 2). All eight strains identified as *S. aureus* by MALDI-TOF analysis had 100% identity with the 16S rRNA gene of *Staphylococcus aureus* MCRF184 (NZ_CP014791.1). The two latex false positive strains were *S. agnetis* and *chromogenes* by 16S sequencing and MALDI_TOF.

**Table 2:** Comparison of *Staphylococcus* spp. identification by MALDI-TOF MS and positive rabbit plasma clotting, Sa442 PCR, latex agglutination, *coa* and *S. aureus cydB* PCR tests

Species	MALDI-TOF MS	Plasma clotting	Sa442 PCR	Latex test	*cydB* PCR	*coa* PCR
*S. agnetis*	17	3	3	1	0	0
*S. aureus*	8	8	8	8	8	7
*S. caprae* ^a^	1	0	0	0	0	0
*S. equorum* ^a^	3	0	0	0	0	0
*S. epidermidis*	8	0	0	0	0	0
*S. haemolyticus* ^a^	2	0	0	0	0	0
*S. hominis* ^a^	1	0	0	0	0	0
*S. pateuri* ^a^	2	0	0	0	0	0
*S. saprophyticus* ^a^	1	0	0	0	0	0
*S. sciuri* ^a^	1	0	0	0	0	0
*S. warneri* ^a^	1	0	0	0	0	0
*S. xylosus*	1	1	1	0	0	1
*S. chromogenes*	69	19	19	1	0	2
*S. cohnii*	1	1	1	0	0	0
Total	116	32	32	10	8	10

^a^Data from Pizauro et al. [13]

#### cydB gene analysis and detection

Alignment of gene sequences (https://www.ncbi.nlm.nih.gov/genbank/) suggested that the *cydB* gene is well conserved in *Staphylococcus* and thus allowed for the design of species-specific primers (Additional file 2: Fig. S2). The eight *S. aureus* isolates were positive for the *S. aureus*-specific *cydB* primers and resultant amplicons had 99–100% identity with *S. aureus* NCTC 8325. Further, the *S. aureus* specific *cydB* primers did not amplify any of the other CoPS tested in this study nor the 84 strains of other putative staphylococci evaluated by Pizauro et al. [13].

#### coa gene analysis

Alignment of 103 coagulase gene sequences (https://www.ncbi.nlm.nih.gov/genbank/) revealed that although *coa* genes possess many polymorphic areas, a region between 1300 and 1600 bp has sufficient homology to be used for detection in *S. aureus* strains (Table 1, Additional file 1: Fig. S1). All of the *S. aureus* positive control strains tested [COL (NC_002951.2), NewMan (NC_009641.1), MW2 (NC_003923.1), Mu50 (NC_002745.2) and *Staphylococcus aureus* ATCC (25923)] were positive using the *coa*F and *coa*R primer pair.

#### Coagulase test and coa gene detection

Twenty-four strains clotted rabbit plasma (Table 2). Eight strains gave discordant results with at least one negative and one positive. Of these strains, seven of the *S. aureus* (n = 8) and two of the *S. chromogenes* (n = 19) and one *S. xylosus* strain were positive for the *coa* gene while none of the *S. agnetis* (n = 3) nor the *S. cohnii* were positive for the *coa* gene. The sequence of the PCR products had 98% identity with the *S. aureus coa* gene from strain JCSC 7633 (accession number AB488507.1) and 99% identity with the *coa* gene in *S. aureus* strain MW2 genome (accession number BA000033.2).

### Discussion

A number of typically coagulase negative *Staphylococcus* spp. including more than a quarter of *S. chromogenes* isolates (19/69) and at least some *S. xylosus* (1/1), *S. cohnii* (1/1), and *S. agnetis* (3/17) were coagulase positive. This finding is consistent with studies of Santos et al. [15] in which 23/42 CoNS strains clotted rabbit plasma. These authors suggested that this phenotype was related to specific PFGE-types, but not with the clumping factor test. The presence of coagulase is an indicator of pathogenicity since it enables bacteria to resist phagocytosis and cause chronic infections [16]. Host specific adaptations can be acquired though mobile genetic elements (MGEs) [16, 17] from nearby *S. aureus* [16] or other CoPS such as *S. pseudointermedis* [15]. Thus, a *coa* gene in CoNS with the newly described *coa* primers may be the result of such transfer. Coagulase activity in the current study may also be related to another gene such as the one described in *S. chromogenes* that shares 41% identity with the predicted coagulase gene of the *S. pseudintermedius* [15].

In this study, non-*S. aureus* stains able to clot plasma (19/19 *S. chromogenes,* 3/3 *S. agnetis,* 1/1 *S. cohnii* and 1/1 *S. xylosus*) were also Sa442 positive. This is the first report of false positive reactions with these species; however, further stains/herds should be tested to know whether these findings can be generalized. When the sequences of the Sa442 primers [4] were compared with the available genome of *S. chromogenes* MU 970 strain (NZ_JMJF00000000.1*),* no significant homology was detected; however, this draft whole shotgun sequence could be missing the region containing Sa442 sequences. Apart from having been established as unique for *S. aureus*, the Sa442 fragment has not been further characterized [18]. As well, Klaassen et al. [18] and Heilmann et al. [19] have reported false negative results with the Sa442 test and the *nuc* PCR is subject to strain variation [6].

Latex agglutination tests may also be problematic. In previous studies, false positive results have been observed at relatively low frequencies (e.g., 7.9% [20] and 9.3% [21]). The greater false positive reaction in this study (20%) may have been related to the population structure and/or the relatively small sample size.

Given the impact that *S. aureus* can have on both human and animal health, its diagnosis is important [22, 23]. Misidentifying more benign *Staphylococcus* species as *S. aureus,* though arguably less serious, is not without significant economic consequences. In the current study, the eight *S. aureus* strains (as identified by MALDI-TOF/16S rRNA sequencing) were positive with our novel *S. aureus*-specific *cydB* PCR test while no amplification was observed with the Sa442 and plasma clotting-positive *S. chromogenes* (n = 19), *S. agnetis* (n = 3), *S. xylosus* (n = 1) or *S. cohnii* (n = 1) strains tested. Also, it might be noted that *S. caprae, S. hyicus, S. hominis, S. epidermidis, S. haemolyticus, S. warneri, S. equorum, S. sciuri,* and *S. pasteuri* were negative in the *S. aureus*-specific *cydB* real-time PCR test in a complementary study [13] (Table 2).

### Conclusions

In summary, a significant number of CoNS strains could clot rabbit plasma and were positive for the Sa442 PCR test and so, could be misclassified as *S. aureus.* The bases of these phenotypes remain to be determined, but they could be the result of horizontal gene transfer or to the fact that these species are less homogenous than previously thought. On the other hand, MALDI-TOF and a species-specific real-time PCR test for the *cydB* gene may permit accurate identification of CoNS.

## Limitations

This study used samples from one buffalo herd which limits the generalisation of the results. In addition, it was beyond the scope of this study to determine the basis of the abnormal coagulase positive phenotype or to determine if there had been horizontal gene transfer to coagulase negative Staphylococcus strains.

## Additional files

**Additional file 1: Fig. S1.** *S. aureus* coagulase gene *(coa)* alignment using CLC Sequence View 7 software.

**Additional file 2: Fig. S2.** Alignment using CLC Sequence View 7 software of the *cydB* gene of 18 closely related *Staphylococcus* spp. in this study.

## Abbreviations

CoNS: coagulase negative Staphylococcus
MALDI-TOF MS: Matrix-Assisted Laser Desorption Ionization-Time of Flight Mass Spectrometry (MALDI-TOF MS)
qPCR: real-time quantitative PCR
PCR: polymerase chain reaction
CoPS: coagulase positive Staphylococcus
