# Supplementary material for: Some coagulase negative Staphylococcus spp. isolated from buffalo can be misidentified as Staphylococcus aureus by phenotypic and Sa442 PCR methods
Source: BMC Res Notes. 2018 May 30;11:346. doi: 10.1186/s13104-018-3449-8 (PMC5977496; doi:10.1186/s13104-018-3449-8)
Supplement: Supplementary file 1 — Additional file 1: Fig. S1. S. aureus coagulase gene (coa) alignment using CLC Sequence View 7 software. [file 13104_2018_3449_MOESM1_ESM.pdf]

```
                                      20                    40                    60                    80
                                       |                     |                     |                     |
CP007670.1   GTTTATTCTA ---------- -----GTTAA TATATAGTTA ATGTCTTTTA ATATTTTGTT T-CTTTAATG TAGATTGGGC
CP003033.1   GTTTATTCTA ---------- -----GTTAA TATATAGTTA ATGTCTTTTA ATATTTTGTT T-CTTTAATG TAGATTGGGC
CP018205.1   GTTTATTCTA ---------- -----GTTAA TATATAGTTA ATGTCTTTTA ATATTTTGTT T-CTTTAATG TAGATTGGGC
AP017377.1   GTTTATTCTA ---------- -----GTTAA TATATAGTTA ATGTCTTTTA ATATTTTGTT T-CTTTAATG TAGATTGGGC
LT598688.1   GTTTATTCTA ---------- -----GTTAA TATATAGTTA ATGTCTTTTA ATATTTTGTT T-CTTTAATG TAGATTGGGC
CP007676.1   GTTTATTCTA ---------- -----GTTAA TATATAGTTA ATGTCTTTTA ATATTTTGTT T-CTTTAATG TAGATTGGGC
CP007672.1   GTTTATTCTA ---------- -----GTTAA TATATAGTTA ATGTCTTTTA ATATTTTGTT T-CTTTAATG TAGATTGGGC
CP007674.1   GTTTATTCTA ---------- -----GTTAA TATATAGTTA ATGTCTTTTA ATATTTTGTT T-CTTTAATG TAGATTGGGC
CP011526.1   GTTTATTCTA ---------- -----GTTAA TATATAGTTA ATGTCTTTTA ATATTTTGTT T-CTTTAATG TAGATTGGGC
HF937103.1   GTTTATTCTA ---------- -----GTTAA TATATAGTTA ATGTCTTTTA ATATTTTGTT T-CTTTAATG TAGATTGGGC
AP009351.1   GTTTATTCTA ---------- -----GTTAA TATATAGTTA ATGTCTTTTA ATATTTTGTT T-CTTTAATG TAGATTGGGC
CP000046.1   GTTTATTCTA ---------- -----GTTAA TATATAGTTA ATGTCTTTTA ATATTTTGTT T-CTTTAATG TAGATTGGGC
CP000253.1   GTTTATTCTA ---------- -----GTTAA TATATAGTTA ATGTCTTTTA ATATTTTGTT T-CTTTAATG TAGATTGGGC
AC025591.8   GTTTATTCTA ---------- -----GTTAA TATATAGTTA ATGTCTTTTA ATATTTTGTT T-CTTTAATG TAGATTGGGC
LT671859.1   GTTTATTCTA ---------- -----GTTAA TATATAGTTA ATGTCTTTTA ATATTTTGTT T-CTTTAATG TAGATTGGGC
CP007499.1   GTTTATTCTA ---------- -----GTTAA TATATAGTTA ATGTCTTTTA ATATTTTGTT T-CTTTAATG TAGATTGGGC
X17679.1     GTTTATTCTA ---------- -----GTTAA TATATAGTTA ATGTCTTTTA ATATTTTGTT T-CTTTAATG TAGATTGGGC
CP007657.1   GTTTATTCTA ---------- -----GTTAA TATATAGTTA ATGTCTTTTA ATATTTTGTT T-CTTTAATG TAGATTGGGC
CP014444.1   GTTTATTCTA ---------- -----GTTAA TATATAGTTA ATGTCTTTTA ATATTTTGTT T-CTTTAATG TAGATTGGGC
CP014441.1   GTTTATTCTA ---------- -----GTTAA TATATAGTTA ATGTCTTTTA ATATTTTGTT T-CTTTAATG TAGATTGGGC
CP014438.1   GTTTATTCTA ---------- -----GTTAA TATATAGTTA ATGTCTTTTA ATATTTTGTT T-CTTTAATG TAGATTGGGC
CP014435.1   GTTTATTCTA ---------- -----GTTAA TATATAGTTA ATGTCTTTTA ATATTTTGTT T-CTTTAATG TAGATTGGGC
CP014432.1   GTTTATTCTA ---------- -----GTTAA TATATAGTTA ATGTCTTTTA ATATTTTGTT T-CTTTAATG TAGATTGGGC
CP014429.1   GTTTATTCTA ---------- -----GTTAA TATATAGTTA ATGTCTTTTA ATATTTTGTT T-CTTTAATG TAGATTGGGC
CP014426.1   GTTTATTCTA ---------- -----GTTAA TATATAGTTA ATGTCTTTTA ATATTTTGTT T-CTTTAATG TAGATTGGGC
CP014423.1   GTTTATTCTA ---------- -----GTTAA TATATAGTTA ATGTCTTTTA ATATTTTGTT T-CTTTAATG TAGATTGGGC
CP014420.1   GTTTATTCTA ---------- -----GTTAA TATATAGTTA ATGTCTTTTA ATATTTTGTT T-CTTTAATG TAGATTGGGC
CP014415.1   GTTTATTCTA ---------- -----GTTAA TATATAGTTA ATGTCTTTTA ATATTTTGTT T-CTTTAATG TAGATTGGGC
CP014412.1   GTTTATTCTA ---------- -----GTTAA TATATAGTTA ATGTCTTTTA ATATTTTGTT T-CTTTAATG TAGATTGGGC
CP014409.1   GTTTATTCTA ---------- -----GTTAA TATATAGTTA ATGTCTTTTA ATATTTTGTT T-CTTTAATG TAGATTGGGC
CP014407.1   GTTTATTCTA ---------- -----GTTAA TATATAGTTA ATGTCTTTTA ATATTTTGTT T-CTTTAATG TAGATTGGGC
CP014402.1   GTTTATTCTA ---------- -----GTTAA TATATAGTTA ATGTCTTTTA ATATTTTGTT T-CTTTAATG TAGATTGGGC
CP014397.1   GTTTATTCTA ---------- -----GTTAA TATATAGTTA ATGTCTTTTA ATATTTTGTT T-CTTTAATG TAGATTGGGC
CP014392.1   GTTTATTCTA ---------- -----GTTAA TATATAGTTA ATGTCTTTTA ATATTTTGTT T-CTTTAATG TAGATTGGGC
CP014387.1   GTTTATTCTA ---------- -----GTTAA TATATAGTTA ATGTCTTTTA ATATTTTGTT T-CTTTAATG TAGATTGGGC
CP014384.1   GTTTATTCTA ---------- -----GTTAA TATATAGTTA ATGTCTTTTA ATATTTTGTT T-CTTTAATG TAGATTGGGC
CP014381.1   GTTTATTCTA ---------- -----GTTAA TATATAGTTA ATGTCTTTTA ATATTTTGTT T-CTTTAATG TAGATTGGGC
CP014371.1   GTTTATTCTA ---------- -----GTTAA TATATAGTTA ATGTCTTTTA ATATTTTGTT T-CTTTAATG TAGATTGGGC
CP014368.1   GTTTATTCTA ---------- -----GTTAA TATATAGTTA ATGTCTTTTA ATATTTTGTT T-CTTTAATG TAGATTGGGC
CP014365.1   GTTTATTCTA ---------- -----GTTAA TATATAGTTA ATGTCTTTTA ATATTTTGTT T-CTTTAATG TAGATTGGGC
CP014362.1   GTTTATTCTA ---------- -----GTTAA TATATAGTTA ATGTCTTTTA ATATTTTGTT T-CTTTAATG TAGATTGGGC
CP014376.1   GTTTATTCTA ---------- -----GTTAA TATATAGTTA ATGTCTTTTA ATATTTTGTT T-CTTTAATG TAGATTGGGC
CP009423.1   GTTTATTCTA ---------- -----GTTAA TATATAGTTA ATGTCTTTTA ATATTTTGTT T-CTTTAATG TAGATTGGGC
CP016855.1   GTTTATTCTA ---------- -----GTTAA TATATAGTTA ATGTCTTTTA ATATTTTGTT T-CTTTAATG TAGATTGGGC
CP013231.1   GTTTATTCTA ---------- -----GTTAA TATATAGTTA ATGTCTTTTA ATATTTTGTT T-CTTTAATG TAGATTGGGC
CP010300.1   GTTTATTCTA ---------- -----GTTAA TATATAGTTA ATGTCTTTTA ATATTTTGTT T-CTTTAATG TAGATTGGGC
CP010299.1   GTTTATTCTA ---------- -----GTTAA TATATAGTTA ATGTCTTTTA ATATTTTGTT T-CTTTAATG TAGATTGGGC
CP010298.1   GTTTATTCTA ---------- -----GTTAA TATATAGTTA ATGTCTTTTA ATATTTTGTT T-CTTTAATG TAGATTGGGC
CP010297.1   GTTTATTCTA ---------- -----GTTAA TATATAGTTA ATGTCTTTTA ATATTTTGTT T-CTTTAATG TAGATTGGGC
CP010296.1   GTTTATTCTA ---------- -----GTTAA TATATAGTTA ATGTCTTTTA ATATTTTGTT T-CTTTAATG TAGATTGGGC
CP010295.1   GTTTATTCTA ---------- -----GTTAA TATATAGTTA ATGTCTTTTA ATATTTTGTT T-CTTTAATG TAGATTGGGC
CP007690.1   GTTTATTCTA ---------- -----GTTAA TATATAGTTA ATGTCTTTTA ATATTTTGTT T-CTTTAATG TAGATTGGGC
CP007176.1   GTTTATTCTA ---------- -----GTTAA TATATAGTTA ATGTCTTTTA ATATTTTGTT T-CTTTAATG TAGATTGGGC
CP000730.1   GTTTATTCTA ---------- -----GTTAA TATATAGTTA ATGTCTTTTA ATATTTTGTT T-CTTTAATG TAGATTGGGC
CP000255.1   GTTTATTCTA ---------- -----GTTAA TATATAGTTA ATGTCTTTTA ATATTTTGTT T-CTTTAATG TAGATTGGGC
CP007539.1   GTTTATTCTA ---------- -----GTTAA TATATAGTTA ATGTCTTTTA ATATTTTGTT T-CTTTAATG TAGATTGGGC
CP007657.1   GTTTATTCTA ---------- -----GTTAA TATATAGTTA ATGTCTTTTA ATATTTTGTT TTCTTTAATG TAGATTGGGC
AP014921.1   GTTTATTCTA ---------- -----GTTAA TATATAGTTA ATGTCTTTTA ATATTTTGTT T-CTTTAATG TAGATTGGGC
AB436955.1   ---------- ---------- ---------- ---------- ---------- ---------- ---------- ----------
AB489885.1   ---------- ---------- ---------- ---------- ---------- ---------- ---------- ----------
AB489873.1   ---------- ---------- ---------- ---------- ---------- ---------- ---------- ----------
AB489883.1   ---------- ---------- ---------- ---------- ---------- ---------- ---------- ----------
AB489874.1   ---------- ---------- ---------- ---------- ---------- ---------- ---------- ----------
LT615218.1   GTTTATTCTA ---------- -----GTTAA TATATAGTTA ATGTCTTTTA ATATTTTGTT T-CTTTAATG TAGATTGGGC
AB436976.1   GTTTATTCTA ---------- -----GTTAA TATATAGTTA ATGTCTTTTA ATATTTTGTT T-CTTTAATG TAGATTGGGC
AB489892.1   ---------- ---------- ---------- ---------- ---------- ---------- ---------- ----------
AJ306908.1   GTTTATTCTA ---------- -----GTTAA TATATAGTTA ATGTCTTTTA ATATTTTGTT T-CTTTAATG TAGATTGGGC
CP015646.1   GTTTATTCTA ---------- -----GTTAA TATATAGTTA ATGTCTTTTA ATATTTTGTT T-CTTTAATG TAGATTGGGC
AB488510.1   ---------- ---------- ---------- ---------- ---------- ---------- ---------- ----------
EU105387.1   G--------- ---------- ---------- ---------- ---------- ---------- ---------- -------GGC
AB436975.1   GTTTATTCTA ---------- -----GTTAA TATATAGTTA ATGTCTTTTA ATATTTTGTT T-CTTTAATG TAGATTGGGC
AB488499.1   ---------- ---------- ---------- ---------- ---------- ---------- ---------- ----------
AJ309189.1   ---------- ---------- ---------- ---------- ---------- ---------- ---------- ----------
FR821779.1   GTTTATTCTA ---------- -----GTTAA TATATAGTTA ATGTCTTTTA ATATTTTGTT T-CTTTAATG TAGATTGGGC
CP012593.1   ---------- ---------- ---------- ---------- ---------- ---------- ---------- ----------
CP012692.1   ---------- ---------- ---------- ---------- ---------- ---------- ---------- ----------
CP013955.1   ---------- ---------- ---------- ---------- ---------- ---------- ---------- ----------
CP013953.1   ---------- ---------- ---------- ---------- ---------- ---------- ---------- ----------
CP014064.1   ---------- ---------- ---------- ---------- ---------- ---------- ---------- ----------
LT009690.1   ---------- ---------- ---------- ---------- ---------- ---------- ---------- ----------
AP017320.1   ---------- ---------- ---------- ---------- ---------- ---------- ---------- ----------
CP010890.1   ---------- ---------- ---------- ---------- ---------- ---------- ---------- ----------
CP001844.2   ---------- ---------- ---------- ---------- ---------- ---------- ---------- ----------
CP001781.1   ---------- ---------- ---------- ---------- ---------- ---------- ---------- ----------
AB488501.1   ---------- ---------- ---------- ---------- ---------- ---------- ---------- ----------
AB489898.1   ---------- ---------- ---------- ---------- ---------- ---------- ---------- ----------
HE579073.1   ---------- ---------- ---------- ---------- ---------- ---------- ---------- ----------
HE579071.1   ---------- ---------- ---------- ---------- ---------- ---------- ---------- ----------
HE579069.1   ---------- ---------- ---------- ---------- ---------- ---------- ---------- ----------
HE579065.1   ---------- ---------- ---------- ---------- ---------- ---------- ---------- ----------
HE579063.1   ---------- ---------- ---------- ---------- ---------- ---------- ---------- ----------
```

		100	
CP007670.1	AATTACATTT	TGGAGGAATT	84
CP003033.1	AATTACATTT	TGGAGGAATT	84
CP018205.1	AATTACATTT	TGGAGGAATT	84
AP017377.1	AATTACATTT	TGGAGGAATT	84
LT598688.1	AATTACATTT	TGGAGGAATT	84
CP007676.1	AATTACATTT	TGGAGGAATT	84
CP007672.1	AATTACATTT	TGGAGGAATT	84
CP007674.1	AATTACATTT	TGGAGGAATT	84
CP011526.1	AATTACATTT	TGGAGGAATT	84
HF937103.1	AATTACATTT	TGGAGGAATT	84
AP009351.1	AATTACATTT	TGGAGGAATT	84
CP000046.1	AATTACATTT	TGGAGGAATT	84
CP000253.1	AATTACATTT	TGGAGGAATT	84
AC025591.8	AATTACATTT	TGGAGGAATT	84
LT671859.1	AATTACATTT	TGGAGGAATT	84
CP007499.1	AATTACATTT	TGGAGGAATT	84
X17679.1	AATTACATTT	TGGAGGAATT	84
CP007657.1	AATTACATTT	TGGAGGAATT	84
CP014444.1	AATTACATTT	TGGAGGAATT	84
CP014441.1	AATTACATTT	TGGAGGAATT	84
CP014438.1	AATTACATTT	TGGAGGAATT	84
CP014435.1	AATTACATTT	TGGAGGAATT	84
CP014432.1	AATTACATTT	TGGAGGAATT	84
CP014429.1	AATTACATTT	TGGAGGAATT	84
CP014426.1	AATTACATTT	TGGAGGAATT	84
CP014423.1	AATTACATTT	TGGAGGAATT	84
CP014420.1	AATTACATTT	TGGAGGAATT	84
CP014415.1	AATTACATTT	TGGAGGAATT	84
CP014412.1	AATTACATTT	TGGAGGAATT	84
CP014409.1	AATTACATTT	TGGAGGAATT	84
CP014407.1	AATTACATTT	TGGAGGAATT	84
CP014402.1	AATTACATTT	TGGAGGAATT	84
CP014397.1	AATTACATTT	TGGAGGAATT	84
CP014392.1	AATTACATTT	TGGAGGAATT	84
CP014387.1	AATTACATTT	TGGAGGAATT	84
CP014384.1	AATTACATTT	TGGAGGAATT	84
CP014381.1	AATTACATTT	TGGAGGAATT	84
CP014371.1	AATTACATTT	TGGAGGAATT	84
CP014368.1	AATTACATTT	TGGAGGAATT	84
CP014365.1	AATTACATTT	TGGAGGAATT	84
CP014362.1	AATTACATTT	TGGAGGAATT	84
CP014376.1	AATTACATTT	TGGAGGAATT	84
CP009423.1	AATTACATTT	TGGAGGAATT	84
CP016855.1	AATTACATTT	TGGAGGAATT	84
CP013231.1	AATTACATTT	TGGAGGAATT	84
CP010300.1	AATTACATTT	TGGAGGAATT	84
CP010299.1	AATTACATTT	TGGAGGAATT	84
CP010298.1	AATTACATTT	TGGAGGAATT	84
CP010297.1	AATTACATTT	TGGAGGAATT	84
CP010296.1	AATTACATTT	TGGAGGAATT	84
CP010295.1	AATTACATTT	TGGAGGAATT	84
CP007690.1	AATTACATTT	TGGAGGAATT	84
CP007176.1	AATTACATTT	TGGAGGAATT	84
CP000730.1	AATTACATTT	TGGAGGAATT	84
CP000255.1	AATTACATTT	TGGAGGAATT	84
CP007539.1	AATTACATTT	TGGAGGAATT	84
CP007657.1	AATTACATTT	TGGAGGAATT	85
AP014921.1	AATTACATTT	TGGAGGAATT	84
AB436955.1	----------	----------	-
AB489885.1	----------	----------	-
AB489873.1	----------	----------	-
AB489883.1	----------	----------	-
AB489874.1	----------	----------	-
LT615218.1	AATTACATTT	TGGAGGAATT	84
AB436976.1	AATTACATTT	TGGAGGAATT	84
AB489892.1	----------	----------	-
AJ306908.1	AATTACATTT	TGGAGGAATT	84
CP015646.1	AATTACATTT	TGGAGGAATT	84
AB488510.1	----------	----------	-
EU105387.1	AATTACATTT	TGGAGGAATT	24
AB436975.1	AATTACATTT	TGGAGGAATT	84
AB488499.1	----------	----------	-
AJ309189.1	----------	----------	-
FR821779.1	AATTACATTT	TGGAGGAATT	84
CP012593.1	----------	----------	-
CP012692.1	----------	----------	-
CP013955.1	----------	----------	-
CP013953.1	----------	----------	-
CP014064.1	----------	----------	-
LT009690.1	----------	----------	-
AP017320.1	----------	----------	-
CP010890.1	----------	----------	-
CP001844.2	----------	----------	-
CP001781.1	----------	----------	-
AB488501.1	----------	----------	-
AB489898.1	----------	----------	-
HE579073.1	----------	----------	-
HE579071.1	----------	----------	-
HE579069.1	----------	----------	-
HE579065.1	----------	----------	-
HE579063.1	----------	----------	-

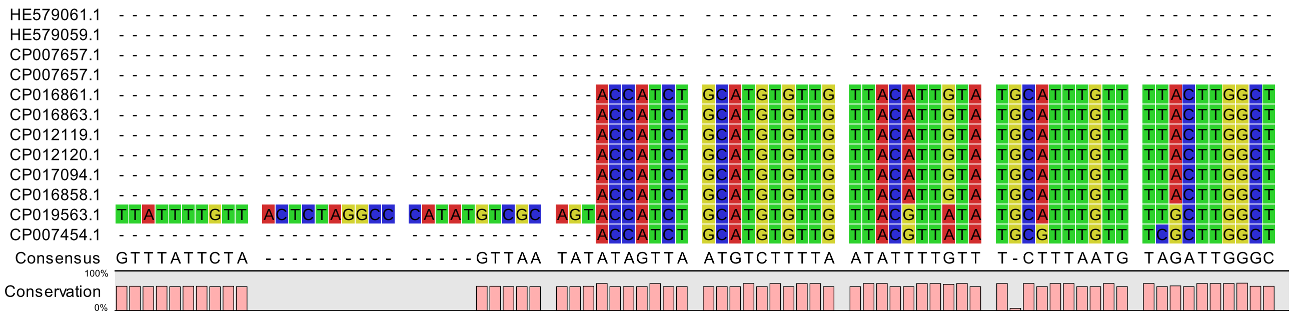
HE579061.1 ---------- ---------- ---------- ---------- ---------- ---------- ---------- ----------
HE579059.1 ---------- ---------- ---------- ---------- ---------- ---------- ---------- ----------
CP007657.1 ---------- ---------- ---------- ---------- ---------- ---------- ---------- ----------
CP007657.1 ---------- ---------- ---------- ---------- ---------- ---------- ---------- ----------
CP016861.1 ---------- ---------- ---------- ---ACCATCT GCATGTGTTG TTACATTGTA TGCATTTGTT TTACTTGGCT
CP016863.1 ---------- ---------- ---------- ---ACCATCT GCATGTGTTG TTACATTGTA TGCATTTGTT TTACTTGGCT
CP012119.1 ---------- ---------- ---------- ---ACCATCT GCATGTGTTG TTACATTGTA TGCATTTGTT TTACTTGGCT
CP012120.1 ---------- ---------- ---------- ---ACCATCT GCATGTGTTG TTACATTGTA TGCATTTGTT TTACTTGGCT
CP017094.1 ---------- ---------- ---------- ---ACCATCT GCATGTGTTG TTACATTGTA TGCATTTGTT TTACTTGGCT
CP016858.1 ---------- ---------- ---------- ---ACCATCT GCATGTGTTG TTACATTGTA TGCATTTGTT TTACTTGGCT
CP019563.1 TTATTTTGTT ACTCTAGGCC CATATGTCGC AGTACCATCT GCATGTGTTG TTACGTTATA TGCATTTGTT TTGCTTGGCT
CP007454.1 ---------- ---------- ---------- ---ACCATCT GCATGTGTTG TTACGTTATA TGCGTTTGTT TCGCTTGGCT
Consensus GTTTATTCTA ---------- -----GTTAA TATATAGTTA ATGTCTTTTA ATATTTTGTT T-CTTTAATG TAGATTGGGC
100%
Conservation
0%

HE579061.1	- - - - - - - - - -	- - - - - - - - - -	-
HE579059.1	- - - - - - - - - -	- - - - - - - - - -	-
CP007657.1	- - - - - - - - - -	- - - - - - - - - -	-
CP007657.1	- - - - - - - - - -	- - - - - - - - - -	-
CP016861.1	TCTTGTATGT	CGGGCGAGCT	67
CP016863.1	TCTTGTATGT	CGGGCGAGCT	67
CP012119.1	TCTTGTATGT	CGGGCGAGCT	67
CP012120.1	TCTTGTATGT	CGGGCGAGCT	67
CP017094.1	TCTTGTATGT	CGGGCGAGCT	67
CP016858.1	TCTTGTATGT	CGGGCGAGCT	67
CP019563.1	TTTTTTGTGT	CGGGCGAGCG	100
CP007454.1	TTTTTTGTGT	CGGGCGAGCG	67
Consensus	AATTACATTT	TGGAGGAATT	

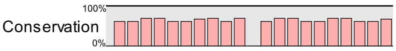

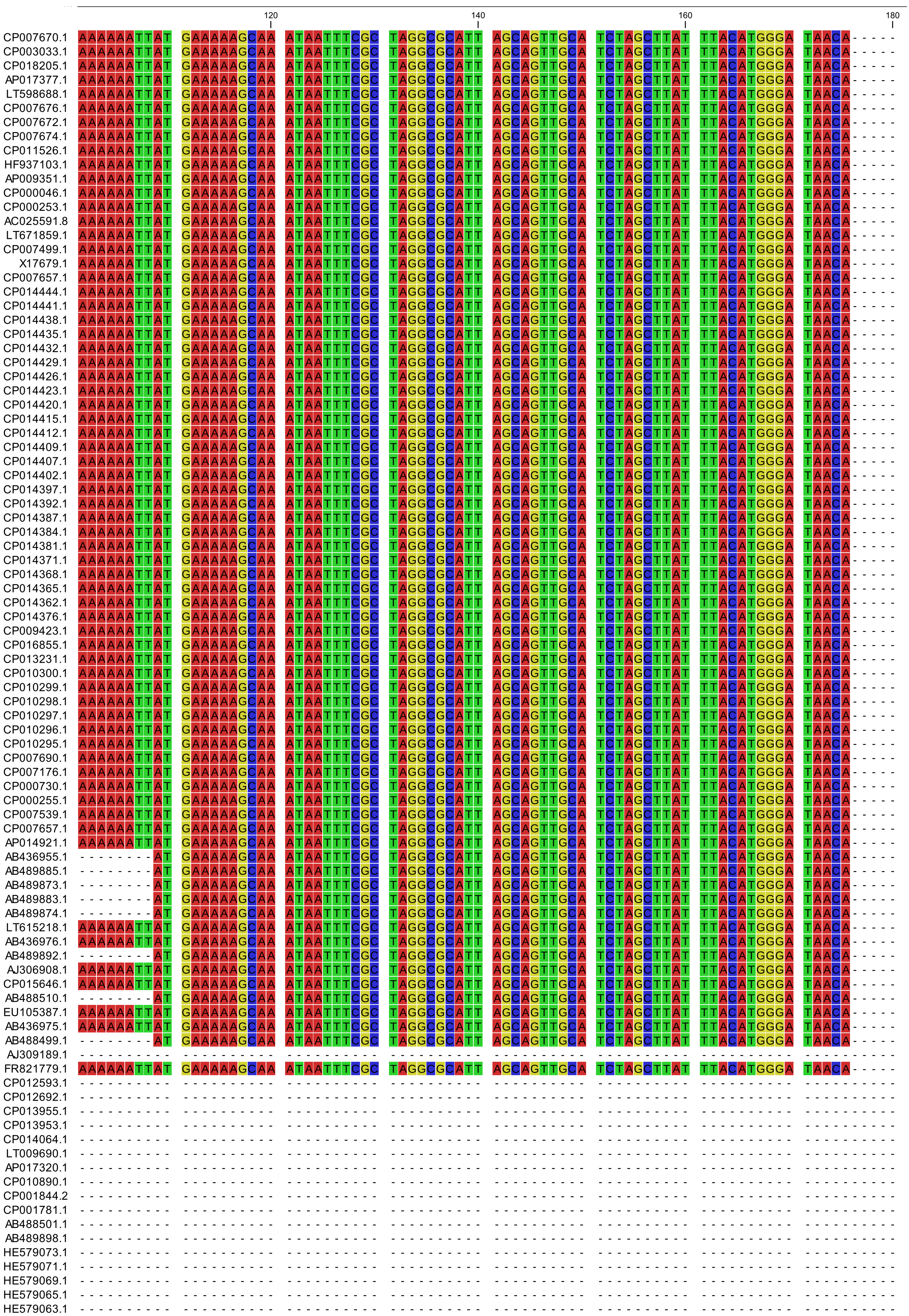
120
140
160
180
CP007670.1 AAAAAATTAT GAAAAAGCAA ATAATTTCGC TAGGCGCATT AGCAGTTGCA TCTAGCTTAT TTACATGGGA TAACA-----
CP003033.1 AAAAAATTAT GAAAAAGCAA ATAATTTCGC TAGGCGCATT AGCAGTTGCA TCTAGCTTAT TTACATGGGA TAACA-----
CP018205.1 AAAAAATTAT GAAAAAGCAA ATAATTTCGC TAGGCGCATT AGCAGTTGCA TCTAGCTTAT TTACATGGGA TAACA-----
AP017377.1 AAAAAATTAT GAAAAAGCAA ATAATTTCGC TAGGCGCATT AGCAGTTGCA TCTAGCTTAT TTACATGGGA TAACA-----
LT598688.1 AAAAAATTAT GAAAAAGCAA ATAATTTCGC TAGGCGCATT AGCAGTTGCA TCTAGCTTAT TTACATGGGA TAACA-----
CP007676.1 AAAAAATTAT GAAAAAGCAA ATAATTTCGC TAGGCGCATT AGCAGTTGCA TCTAGCTTAT TTACATGGGA TAACA-----
CP007672.1 AAAAAATTAT GAAAAAGCAA ATAATTTCGC TAGGCGCATT AGCAGTTGCA TCTAGCTTAT TTACATGGGA TAACA-----
CP007674.1 AAAAAATTAT GAAAAAGCAA ATAATTTCGC TAGGCGCATT AGCAGTTGCA TCTAGCTTAT TTACATGGGA TAACA-----
CP011526.1 AAAAAATTAT GAAAAAGCAA ATAATTTCGC TAGGCGCATT AGCAGTTGCA TCTAGCTTAT TTACATGGGA TAACA-----
HF937103.1 AAAAAATTAT GAAAAAGCAA ATAATTTCGC TAGGCGCATT AGCAGTTGCA TCTAGCTTAT TTACATGGGA TAACA-----
AP009351.1 AAAAAATTAT GAAAAAGCAA ATAATTTCGC TAGGCGCATT AGCAGTTGCA TCTAGCTTAT TTACATGGGA TAACA-----
CP000046.1 AAAAAATTAT GAAAAAGCAA ATAATTTCGC TAGGCGCATT AGCAGTTGCA TCTAGCTTAT TTACATGGGA TAACA-----
CP000253.1 AAAAAATTAT GAAAAAGCAA ATAATTTCGC TAGGCGCATT AGCAGTTGCA TCTAGCTTAT TTACATGGGA TAACA-----
AC025591.8 AAAAAATTAT GAAAAAGCAA ATAATTTCGC TAGGCGCATT AGCAGTTGCA TCTAGCTTAT TTACATGGGA TAACA-----
LT671859.1 AAAAAATTAT GAAAAAGCAA ATAATTTCGC TAGGCGCATT AGCAGTTGCA TCTAGCTTAT TTACATGGGA TAACA-----
CP007499.1 AAAAAATTAT GAAAAAGCAA ATAATTTCGC TAGGCGCATT AGCAGTTGCA TCTAGCTTAT TTACATGGGA TAACA-----
X17679.1 AAAAAATTAT GAAAAAGCAA ATAATTTCGC TAGGCGCATT AGCAGTTGCA TCTAGCTTAT TTACATGGGA TAACA-----
CP007657.1 AAAAAATTAT GAAAAAGCAA ATAATTTCGC TAGGCGCATT AGCAGTTGCA TCTAGCTTAT TTACATGGGA TAACA-----
CP014444.1 AAAAAATTAT GAAAAAGCAA ATAATTTCGC TAGGCGCATT AGCAGTTGCA TCTAGCTTAT TTACATGGGA TAACA-----
CP014441.1 AAAAAATTAT GAAAAAGCAA ATAATTTCGC TAGGCGCATT AGCAGTTGCA TCTAGCTTAT TTACATGGGA TAACA-----
CP014438.1 AAAAAATTAT GAAAAAGCAA ATAATTTCGC TAGGCGCATT AGCAGTTGCA TCTAGCTTAT TTACATGGGA TAACA-----
CP014435.1 AAAAAATTAT GAAAAAGCAA ATAATTTCGC TAGGCGCATT AGCAGTTGCA TCTAGCTTAT TTACATGGGA TAACA-----
CP014432.1 AAAAAATTAT GAAAAAGCAA ATAATTTCGC TAGGCGCATT AGCAGTTGCA TCTAGCTTAT TTACATGGGA TAACA-----
CP014429.1 AAAAAATTAT GAAAAAGCAA ATAATTTCGC TAGGCGCATT AGCAGTTGCA TCTAGCTTAT TTACATGGGA TAACA-----
CP014426.1 AAAAAATTAT GAAAAAGCAA ATAATTTCGC TAGGCGCATT AGCAGTTGCA TCTAGCTTAT TTACATGGGA TAACA-----
CP014423.1 AAAAAATTAT GAAAAAGCAA ATAATTTCGC TAGGCGCATT AGCAGTTGCA TCTAGCTTAT TTACATGGGA TAACA-----
CP014420.1 AAAAAATTAT GAAAAAGCAA ATAATTTCGC TAGGCGCATT AGCAGTTGCA TCTAGCTTAT TTACATGGGA TAACA-----
CP014415.1 AAAAAATTAT GAAAAAGCAA ATAATTTCGC TAGGCGCATT AGCAGTTGCA TCTAGCTTAT TTACATGGGA TAACA-----
CP014412.1 AAAAAATTAT GAAAAAGCAA ATAATTTCGC TAGGCGCATT AGCAGTTGCA TCTAGCTTAT TTACATGGGA TAACA-----
CP014409.1 AAAAAATTAT GAAAAAGCAA ATAATTTCGC TAGGCGCATT AGCAGTTGCA TCTAGCTTAT TTACATGGGA TAACA-----
CP014407.1 AAAAAATTAT GAAAAAGCAA ATAATTTCGC TAGGCGCATT AGCAGTTGCA TCTAGCTTAT TTACATGGGA TAACA-----
CP014402.1 AAAAAATTAT GAAAAAGCAA ATAATTTCGC TAGGCGCATT AGCAGTTGCA TCTAGCTTAT TTACATGGGA TAACA-----
CP014397.1 AAAAAATTAT GAAAAAGCAA ATAATTTCGC TAGGCGCATT AGCAGTTGCA TCTAGCTTAT TTACATGGGA TAACA-----
CP014392.1 AAAAAATTAT GAAAAAGCAA ATAATTTCGC TAGGCGCATT AGCAGTTGCA TCTAGCTTAT TTACATGGGA TAACA-----
CP014387.1 AAAAAATTAT GAAAAAGCAA ATAATTTCGC TAGGCGCATT AGCAGTTGCA TCTAGCTTAT TTACATGGGA TAACA-----
CP014384.1 AAAAAATTAT GAAAAAGCAA ATAATTTCGC TAGGCGCATT AGCAGTTGCA TCTAGCTTAT TTACATGGGA TAACA-----
CP014381.1 AAAAAATTAT GAAAAAGCAA ATAATTTCGC TAGGCGCATT AGCAGTTGCA TCTAGCTTAT TTACATGGGA TAACA-----
CP014371.1 AAAAAATTAT GAAAAAGCAA ATAATTTCGC TAGGCGCATT AGCAGTTGCA TCTAGCTTAT TTACATGGGA TAACA-----
CP014368.1 AAAAAATTAT GAAAAAGCAA ATAATTTCGC TAGGCGCATT AGCAGTTGCA TCTAGCTTAT TTACATGGGA TAACA-----
CP014365.1 AAAAAATTAT GAAAAAGCAA ATAATTTCGC TAGGCGCATT AGCAGTTGCA TCTAGCTTAT TTACATGGGA TAACA-----
CP014362.1 AAAAAATTAT GAAAAAGCAA ATAATTTCGC TAGGCGCATT AGCAGTTGCA TCTAGCTTAT TTACATGGGA TAACA-----
CP014376.1 AAAAAATTAT GAAAAAGCAA ATAATTTCGC TAGGCGCATT AGCAGTTGCA TCTAGCTTAT TTACATGGGA TAACA-----
CP009423.1 AAAAAATTAT GAAAAAGCAA ATAATTTCGC TAGGCGCATT AGCAGTTGCA TCTAGCTTAT TTACATGGGA TAACA-----
CP016855.1 AAAAAATTAT GAAAAAGCAA ATAATTTCGC TAGGCGCATT AGCAGTTGCA TCTAGCTTAT TTACATGGGA TAACA-----
CP013231.1 AAAAAATTAT GAAAAAGCAA ATAATTTCGC TAGGCGCATT AGCAGTTGCA TCTAGCTTAT TTACATGGGA TAACA-----
CP010300.1 AAAAAATTAT GAAAAAGCAA ATAATTTCGC TAGGCGCATT AGCAGTTGCA TCTAGCTTAT TTACATGGGA TAACA-----
CP010299.1 AAAAAATTAT GAAAAAGCAA ATAATTTCGC TAGGCGCATT AGCAGTTGCA TCTAGCTTAT TTACATGGGA TAACA-----
CP010298.1 AAAAAATTAT GAAAAAGCAA ATAATTTCGC TAGGCGCATT AGCAGTTGCA TCTAGCTTAT TTACATGGGA TAACA-----
CP010297.1 AAAAAATTAT GAAAAAGCAA ATAATTTCGC TAGGCGCATT AGCAGTTGCA TCTAGCTTAT TTACATGGGA TAACA-----
CP010296.1 AAAAAATTAT GAAAAAGCAA ATAATTTCGC TAGGCGCATT AGCAGTTGCA TCTAGCTTAT TTACATGGGA TAACA-----
CP010295.1 AAAAAATTAT GAAAAAGCAA ATAATTTCGC TAGGCGCATT AGCAGTTGCA TCTAGCTTAT TTACATGGGA TAACA-----
CP007690.1 AAAAAATTAT GAAAAAGCAA ATAATTTCGC TAGGCGCATT AGCAGTTGCA TCTAGCTTAT TTACATGGGA TAACA-----
CP007176.1 AAAAAATTAT GAAAAAGCAA ATAATTTCGC TAGGCGCATT AGCAGTTGCA TCTAGCTTAT TTACATGGGA TAACA-----
CP000730.1 AAAAAATTAT GAAAAAGCAA ATAATTTCGC TAGGCGCATT AGCAGTTGCA TCTAGCTTAT TTACATGGGA TAACA-----
CP000255.1 AAAAAATTAT GAAAAAGCAA ATAATTTCGC TAGGCGCATT AGCAGTTGCA TCTAGCTTAT TTACATGGGA TAACA-----
CP007539.1 AAAAAATTAT GAAAAAGCAA ATAATTTCGC TAGGCGCATT AGCAGTTGCA TCTAGCTTAT TTACATGGGA TAACA-----
CP007657.1 AAAAAATTAT GAAAAAGCAA ATAATTTCGC TAGGCGCATT AGCAGTTGCA TCTAGCTTAT TTACATGGGA TAACA-----
AP014921.1 AAAAAATTAT GAAAAAGCAA ATAATTTCGC TAGGCGCATT AGCAGTTGCA TCTAGCTTAT TTACATGGGA TAACA-----
AB436955.1 --------AT GAAAAAGCAA ATAATTTCGC TAGGCGCATT AGCAGTTGCA TCTAGCTTAT TTACATGGGA TAACA-----
AB489885.1 --------AT GAAAAAGCAA ATAATTTCGC TAGGCGCATT AGCAGTTGCA TCTAGCTTAT TTACATGGGA TAACA-----
AB489873.1 --------AT GAAAAAGCAA ATAATTTCGC TAGGCGCATT AGCAGTTGCA TCTAGCTTAT TTACATGGGA TAACA-----
AB489883.1 --------AT GAAAAAGCAA ATAATTTCGC TAGGCGCATT AGCAGTTGCA TCTAGCTTAT TTACATGGGA TAACA-----
AB489874.1 --------AT GAAAAAGCAA ATAATTTCGC TAGGCGCATT AGCAGTTGCA TCTAGCTTAT TTACATGGGA TAACA-----
LT615218.1 AAAAAATTAT GAAAAAGCAA ATAATTTCGC TAGGCGCATT AGCAGTTGCA TCTAGCTTAT TTACATGGGA TAACA-----
AB436976.1 AAAAAATTAT GAAAAAGCAA ATAATTTCGC TAGGCGCATT AGCAGTTGCA TCTAGCTTAT TTACATGGGA TAACA-----
AB489892.1 --------AT GAAAAAGCAA ATAATTTCGC TAGGCGCATT AGCAGTTGCA TCTAGCTTAT TTACATGGGA TAACA-----
AJ306908.1 AAAAAATTAT GAAAAAGCAA ATAATTTCGC TAGGCGCATT AGCAGTTGCA TCTAGCTTAT TTACATGGGA TAACA-----
CP015646.1 AAAAAATTAT GAAAAAGCAA ATAATTTCGC TAGGCGCATT AGCAGTTGCA TCTAGCTTAT TTACATGGGA TAACA-----
AB488510.1 --------AT GAAAAAGCAA ATAATTTCGC TAGGCGCATT AGCAGTTGCA TCTAGCTTAT TTACATGGGA TAACA-----
EU105387.1 AAAAAATTAT GAAAAAGCAA ATAATTTCGC TAGGCGCATT AGCAGTTGCA TCTAGCTTAT TTACATGGGA TAACA-----
AB436975.1 AAAAAATTAT GAAAAAGCAA ATAATTTCGC TAGGCGCATT AGCAGTTGCA TCTAGCTTAT TTACATGGGA TAACA-----
AB488499.1 --------AT GAAAAAGCAA ATAATTTCGC TAGGCGCATT AGCAGTTGCA TCTAGCTTAT TTACATGGGA TAACA-----
AJ309189.1 ---------- ---------- ---------- ---------- ---------- ---------- ---------- ----------
FR821779.1 AAAAAATTAT GAAAAAGCAA ATAATTTCGC TAGGCGCATT AGCAGTTGCA TCTAGCTTAT TTACATGGGA TAACA-----
CP012593.1 ---------- ---------- ---------- ---------- ---------- ---------- ---------- ----------
CP012692.1 ---------- ---------- ---------- ---------- ---------- ---------- ---------- ----------
CP013955.1 ---------- ---------- ---------- ---------- ---------- ---------- ---------- ----------
CP013953.1 ---------- ---------- ---------- ---------- ---------- ---------- ---------- ----------
CP014064.1 ---------- ---------- ---------- ---------- ---------- ---------- ---------- ----------
LT009690.1 ---------- ---------- ---------- ---------- ---------- ---------- ---------- ----------
AP017320.1 ---------- ---------- ---------- ---------- ---------- ---------- ---------- ----------
CP010890.1 ---------- ---------- ---------- ---------- ---------- ---------- ---------- ----------
CP001844.2 ---------- ---------- ---------- ---------- ---------- ---------- ---------- ----------
CP001781.1 ---------- ---------- ---------- ---------- ---------- ---------- ---------- ----------
AB488501.1 ---------- ---------- ---------- ---------- ---------- ---------- ---------- ----------
AB489898.1 ---------- ---------- ---------- ---------- ---------- ---------- ---------- ----------
HE579073.1 ---------- ---------- ---------- ---------- ---------- ---------- ---------- ----------
HE579071.1 ---------- ---------- ---------- ---------- ---------- ---------- ---------- ----------
HE579069.1 ---------- ---------- ---------- ---------- ---------- ---------- ---------- ----------
HE579065.1 ---------- ---------- ---------- ---------- ---------- ---------- ---------- ----------
HE579063.1 ---------- ---------- ---------- ---------- ---------- ---------- ---------- ----------

		200	
CP007670.1	----------	----------	159
CP003033.1	----------	----------	159
CP018205.1	----------	----------	159
AP017377.1	----------	----------	159
LT598688.1	----------	----------	159
CP007676.1	----------	----------	159
CP007672.1	----------	----------	159
CP007674.1	----------	----------	159
CP011526.1	----------	----------	159
HF937103.1	----------	----------	159
AP009351.1	----------	----------	159
CP000046.1	----------	----------	159
CP000253.1	----------	----------	159
AC025591.8	----------	----------	159
LT671859.1	----------	----------	159
CP007499.1	----------	----------	159
X17679.1	----------	----------	159
CP007657.1	----------	----------	159
CP014444.1	----------	----------	159
CP014441.1	----------	----------	159
CP014438.1	----------	----------	159
CP014435.1	----------	----------	159
CP014432.1	----------	----------	159
CP014429.1	----------	----------	159
CP014426.1	----------	----------	159
CP014423.1	----------	----------	159
CP014420.1	----------	----------	159
CP014415.1	----------	----------	159
CP014412.1	----------	----------	159
CP014409.1	----------	----------	159
CP014407.1	----------	----------	159
CP014402.1	----------	----------	159
CP014397.1	----------	----------	159
CP014392.1	----------	----------	159
CP014387.1	----------	----------	159
CP014384.1	----------	----------	159
CP014381.1	----------	----------	159
CP014371.1	----------	----------	159
CP014368.1	----------	----------	159
CP014365.1	----------	----------	159
CP014362.1	----------	----------	159
CP014376.1	----------	----------	159
CP009423.1	----------	----------	159
CP016855.1	----------	----------	159
CP013231.1	----------	----------	159
CP010300.1	----------	----------	159
CP010299.1	----------	----------	159
CP010298.1	----------	----------	159
CP010297.1	----------	----------	159
CP010296.1	----------	----------	159
CP010295.1	----------	----------	159
CP007690.1	----------	----------	159
CP007176.1	----------	----------	159
CP000730.1	----------	----------	159
CP000255.1	----------	----------	159
CP007539.1	----------	----------	159
CP007657.1	----------	----------	160
AP014921.1	----------	----------	159
AB436955.1	----------	----------	67
AB489885.1	----------	----------	67
AB489873.1	----------	----------	67
AB489883.1	----------	----------	67
AB489874.1	----------	----------	67
LT615218.1	----------	----------	159
AB436976.1	----------	----------	159
AB489892.1	----------	----------	67
AJ306908.1	----------	----------	159
CP015646.1	----------	----------	159
AB488510.1	----------	----------	67
EU105387.1	----------	----------	99
AB436975.1	----------	----------	159
AB488499.1	----------	----------	67
AJ309189.1	----------	----------	-
FR821779.1	----------	----------	159
CP012593.1	----------	----------	-
CP012692.1	----------	----------	-
CP013955.1	----------	----------	-
CP013953.1	----------	----------	-
CP014064.1	----------	----------	-
LT009690.1	----------	----------	-
AP017320.1	----------	----------	-
CP010890.1	----------	----------	-
CP001844.2	----------	----------	-
CP001781.1	----------	----------	-
AB488501.1	----------	----------	-
AB489898.1	----------	----------	-
HE579073.1	----------	----------	-
HE579071.1	----------	----------	-
HE579069.1	----------	----------	-
HE579065.1	----------	----------	-
HE579063.1	----------	----------	-

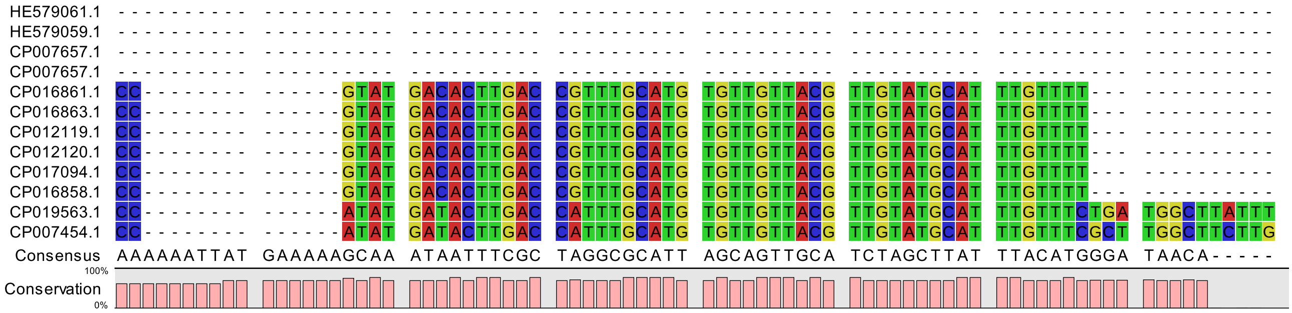
HE579061.1
HE579059.1
CP007657.1
CP007657.1
CP016861.1
CP016863.1
CP012119.1
CP012120.1
CP017094.1
CP016858.1
CP019563.1
CP007454.1
Consensus AAAAAATTAT GAAAAAGCAA ATAATTTCGC TAGGCGCATT AGCAGTTGCA TCTAGCTTAT TTACATGGGA TAACA-----
100%
Conservation
0%

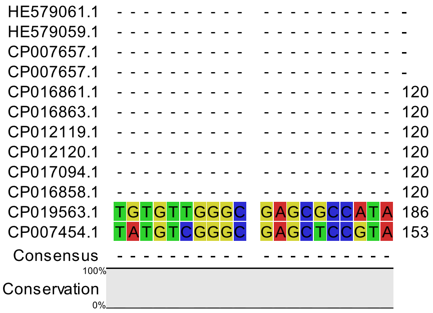
HE579061.1 -
HE579059.1 -
CP007657.1 -
CP007657.1 -
CP016861.1 - 120
CP016863.1 - 120
CP012119.1 - 120
CP012120.1 - 120
CP017094.1 - 120
CP016858.1 - 120
CP019563.1 TGTGTTGGGC GAGCGCCATA 186
CP007454.1 TATGTCGGGC GAGCTCCGTA 153
Consensus -
100%
Conservation
0%

```
                                  220                   240                   260                   280
CP007670.1   ---------- ---------- ---------- -----AAGCA GATGCGATAG TAACAAAGGA TTATAGTGGG AAATCACAAG
CP003033.1   ---------- ---------- ---------- -----AAGCA GATGCGATAG TAACAAAGGA TTATAGTGGG AAATCACAAG
CP018205.1   ---------- ---------- ---------- -----AAGCA GATGCGATAG TAACAAAGGA TTATAGTGGG AAATCACAAG
AP017377.1   ---------- ---------- ---------- -----AAGCA GATGCGATAG TAACAAAGGA TTATAGTGGG AAATCACAAG
LT598688.1   ---------- ---------- ---------- -----AAGCA GATGCGATAG TAACAAAGGA TTATAGTGGG AAATCACAAG
CP007676.1   ---------- ---------- ---------- -----AAGCA GATGCGATAG TAACAAAGGA TTATAGTGGG AAATCACAAG
CP007672.1   ---------- ---------- ---------- -----AAGCA GATGCGATAG TAACAAAGGA TTATAGTGGG AAATCACAAG
CP007674.1   ---------- ---------- ---------- -----AAGCA GATGCGATAG TAACAAAGGA TTATAGTGGG AAATCACAAG
CP011526.1   ---------- ---------- ---------- -----AAGCA GATGCGATAG TAACAAAGGA TTATAGTGGG AAATCACAAG
HF937103.1   ---------- ---------- ---------- -----AAGCA GATGCGATAG TAACAAAGGA TTATAGTGGG AAATCACAAG
AP009351.1   ---------- ---------- ---------- -----AAGCA GATGCGATAG TAACAAAGGA TTATAGTGGG AAATCACAAG
CP000046.1   ---------- ---------- ---------- -----AAGCA GATGCGATAG TAACAAAGGA TTATAGTGGG AAATCACAAG
CP000253.1   ---------- ---------- ---------- -----AAGCA GATGCGATAG TAACAAAGGA TTATAGTGGG AAATCACAAG
AC025591.8   ---------- ---------- ---------- -----AAGCA GATGCGATAG TAACAAAGGA TTATAGTGGG AAATCACAAG
LT671859.1   ---------- ---------- ---------- -----AAGCA GATGCGATAG TAACAAAGGA TTATAGTGGG AAATCACAAG
CP007499.1   ---------- ---------- ---------- -----AAGCA GATGCGATAG TAACAAAGGA TTATAGTGGG AAATCACAAG
X17679.1     ---------- ---------- ---------- -----AAGCA GATGCGATAG TAACAAAGGA TTATAGTGGG AAATCACAAG
CP007657.1   ---------- ---------- ---------- -----AAGCA GATGCGATAG TAACAAAGGA TTATAGTGGG AAATCACAAG
CP014444.1   ---------- ---------- ---------- -----AAGCA GATGCGATAG TAACAAAGGA TTATAGTGGG AAATCACAAG
CP014441.1   ---------- ---------- ---------- -----AAGCA GATGCGATAG TAACAAAGGA TTATAGTGGG AAATCACAAG
CP014438.1   ---------- ---------- ---------- -----AAGCA GATGCGATAG TAACAAAGGA TTATAGTGGG AAATCACAAG
CP014435.1   ---------- ---------- ---------- -----AAGCA GATGCGATAG TAACAAAGGA TTATAGTGGG AAATCACAAG
CP014432.1   ---------- ---------- ---------- -----AAGCA GATGCGATAG TAACAAAGGA TTATAGTGGG AAATCACAAG
CP014429.1   ---------- ---------- ---------- -----AAGCA GATGCGATAG TAACAAAGGA TTATAGTGGG AAATCACAAG
CP014426.1   ---------- ---------- ---------- -----AAGCA GATGCGATAG TAACAAAGGA TTATAGTGGG AAATCACAAG
CP014423.1   ---------- ---------- ---------- -----AAGCA GATGCGATAG TAACAAAGGA TTATAGTGGG AAATCACAAG
CP014420.1   ---------- ---------- ---------- -----AAGCA GATGCGATAG TAACAAAGGA TTATAGTGGG AAATCACAAG
CP014415.1   ---------- ---------- ---------- -----AAGCA GATGCGATAG TAACAAAGGA TTATAGTGGG AAATCACAAG
CP014412.1   ---------- ---------- ---------- -----AAGCA GATGCGATAG TAACAAAGGA TTATAGTGGG AAATCACAAG
CP014409.1   ---------- ---------- ---------- -----AAGCA GATGCGATAG TAACAAAGGA TTATAGTGGG AAATCACAAG
CP014407.1   ---------- ---------- ---------- -----AAGCA GATGCGATAG TAACAAAGGA TTATAGTGGG AAATCACAAG
CP014402.1   ---------- ---------- ---------- -----AAGCA GATGCGATAG TAACAAAGGA TTATAGTGGG AAATCACAAG
CP014397.1   ---------- ---------- ---------- -----AAGCA GATGCGATAG TAACAAAGGA TTATAGTGGG AAATCACAAG
CP014392.1   ---------- ---------- ---------- -----AAGCA GATGCGATAG TAACAAAGGA TTATAGTGGG AAATCACAAG
CP014387.1   ---------- ---------- ---------- -----AAGCA GATGCGATAG TAACAAAGGA TTATAGTGGG AAATCACAAG
CP014384.1   ---------- ---------- ---------- -----AAGCA GATGCGATAG TAACAAAGGA TTATAGTGGG AAATCACAAG
CP014381.1   ---------- ---------- ---------- -----AAGCA GATGCGATAG TAACAAAGGA TTATAGTGGG AAATCACAAG
CP014371.1   ---------- ---------- ---------- -----AAGCA GATGCGATAG TAACAAAGGA TTATAGTGGG AAATCACAAG
CP014368.1   ---------- ---------- ---------- -----AAGCA GATGCGATAG TAACAAAGGA TTATAGTGGG AAATCACAAG
CP014365.1   ---------- ---------- ---------- -----AAGCA GATGCGATAG TAACAAAGGA TTATAGTGGG AAATCACAAG
CP014362.1   ---------- ---------- ---------- -----AAGCA GATGCGATAG TAACAAAGGA TTATAGTGGG AAATCACAAG
CP014376.1   ---------- ---------- ---------- -----AAGCA GATGCGATAG TAACAAAGGA TTATAGTGGG AAATCACAAG
CP009423.1   ---------- ---------- ---------- -----AAGCA GATGCGATAG TAACAAAGGA TTATAGTGGG AAATCACAAG
CP016855.1   ---------- ---------- ---------- -----AAGCA GATGCGATAG TAACAAAGGA TTATAGTGGG AAATCACAAG
CP013231.1   ---------- ---------- ---------- -----AAGCA GATGCGATAG TAACAAAGGA TTATAGTGGG AAATCACAAG
CP010300.1   ---------- ---------- ---------- -----AAGCA GATGCGATAG TAACAAAGGA TTATAGTGGG AAATCACAAG
CP010299.1   ---------- ---------- ---------- -----AAGCA GATGCGATAG TAACAAAGGA TTATAGTGGG AAATCACAAG
CP010298.1   ---------- ---------- ---------- -----AAGCA GATGCGATAG TAACAAAGGA TTATAGTGGG AAATCACAAG
CP010297.1   ---------- ---------- ---------- -----AAGCA GATGCGATAG TAACAAAGGA TTATAGTGGG AAATCACAAG
CP010296.1   ---------- ---------- ---------- -----AAGCA GATGCGATAG TAACAAAGGA TTATAGTGGG AAATCACAAG
CP010295.1   ---------- ---------- ---------- -----AAGCA GATGCGATAG TAACAAAGGA TTATAGTGGG AAATCACAAG
CP007690.1   ---------- ---------- ---------- -----AAGCA GATGCGATAG TAACAAAGGA TTATAGTGGG AAATCACAAG
CP007176.1   ---------- ---------- ---------- -----AAGCA GATGCGATAG TAACAAAGGA TTATAGTGGG AAATCACAAG
CP000730.1   ---------- ---------- ---------- -----AAGCA GATGCGATAG TAACAAAGGA TTATAGTGGG AAATCACAAG
CP000255.1   ---------- ---------- ---------- -----AAGCA GATGCGATAG TAACAAAGGA TTATAGTGGG AAATCACAAG
CP007539.1   ---------- ---------- ---------- -----AAGCA GATGCGATAG TAACAAAGGA TTATAGTGGG AAATCACAAG
CP007657.1   ---------- ---------- ---------- -----AAGCA GATGCGATAG TAACAAAGGA TTATAGTGGG AAATCACAAG
AP014921.1   ---------- ---------- ---------- -----AAGCA GATGCGATAG TAACAAAGGA TTATAGTGGG AAATCACAAG
AB436955.1   ---------- ---------- ---------- -----AAGCA GATGCGATAG TAACAAAGGA TTATAGTGGG AAATCACAAG
AB489885.1   ---------- ---------- ---------- -----AAGCA GATGCGATAG TAACAAAGGA TTATAGTGGG AAATCACAAG
AB489873.1   ---------- ---------- ---------- -----AAGCA GATGCGATAG TAACAAAGGA TTATAGTGGG AAATCACAAG
AB489883.1   ---------- ---------- ---------- -----AAGCA GATGCGATAG TAACAAAGGA TTATAGTGGG AAATCACAAG
AB489874.1   ---------- ---------- ---------- -----AAGCA GATGCGATAG TAACAAAGGA TTATAGTGGG AAATCACAAG
LT615218.1   ---------- ---------- ---------- -----AAGCA GATGCGATAG TAACAAAGGA TTATAGTGGG AAATCACAAG
AB436976.1   ---------- ---------- ---------- -----AAGCA GATGCGATAG TAACAAAGGA TTATAGTGGG AAATCACAAG
AB489892.1   ---------- ---------- ---------- -----AAGCA GATGCGATAG TAACAAAGGA TTATAGTGGG AAATCACAAG
AJ306908.1   ---------- ---------- ---------- -----AAGCA GATGCGATAG TAACAAAGGA TTATAATGGG AAATCACAAG
CP015646.1   ---------- ---------- ---------- -----AAGCA GATGCGATAG TAACAAAGGA TTATAATGGG AAATCACAAG
AB488510.1   ---------- ---------- ---------- -----AAGCA GATGCGATAG TAACAAAGGA TTATAGTGGG AAATCACAAG
EU105387.1   ---------- ---------- ---------- -----AAGCA GATGCGATAG TGACAAAGGA TTATAGTGGG AAATCACAAG
AB436975.1   ---------- ---------- ---------- -----AAGCA GATGCGATAG TAACAAAGGA TTATAGTGGG AAATCACAAG
AB488499.1   ---------- ---------- ---------- -----AAGCA GATGCGATAG TAACAAAGGA TTATAGTGGG AAATCACAAG
AJ309189.1   ---------- ---------- ---------- ---------- ---------- --ACAAAGGA TTATAGTGGG AAATCACAAG
FR821779.1   ---------- ---------- ---------- -----AAGCA GATGCGATAG TAACAAAGGA TTATAGCAAA GAGTCAAGAG
CP012593.1   ---------- ---------- ---------- ---------- ---------- ---------- ---------- ----------
CP012692.1   ---------- ---------- ---------- ---------- ---------- ---------- ---------- ----------
CP013955.1   ---------- ---------- ---------- ---------- ---------- ---------- ---------- ----------
CP013953.1   ---------- ---------- ---------- ---------- ---------- ---------- ---------- ----------
CP014064.1   ---------- ---------- ---------- ---------- ---------- ---------- ---------- ----------
LT009690.1   ---------- ---------- ---------- ---------- ---------- ---------- ---------- ----------
AP017320.1   ---------- ---------- ---------- ---------- ---------- ---------- ---------- ----------
CP010890.1   ---------- ---------- ---------- ---------- ---------- ---------- ---------- ----------
CP001844.2   ---------- ---------- ---------- ---------- ---------- ---------- ---------- ----------
CP001781.1   ---------- ---------- ---------- ---------- ---------- ---------- ---------- ----------
AB488501.1   ---------- ---------- ---------- ---------- ---------- ---------- ---------- ----------
AB489898.1   ---------- ---------- ---------- ---------- ---------- ---------- ---------- ----------
HE579073.1   ---------- ---------- ---------- ---------- ---------- ---------- ---------- ----------
HE579071.1   ---------- ---------- ---------- ---------- ---------- ---------- ---------- ----------
HE579069.1   ---------- ---------- ---------- ---------- ---------- ---------- ---------- ----------
HE579065.1   ---------- ---------- ---------- ---------- ---------- ---------- ---------- ----------
HE579063.1   ---------- ---------- ---------- ---------- ---------- ---------- ---------- ----------
```

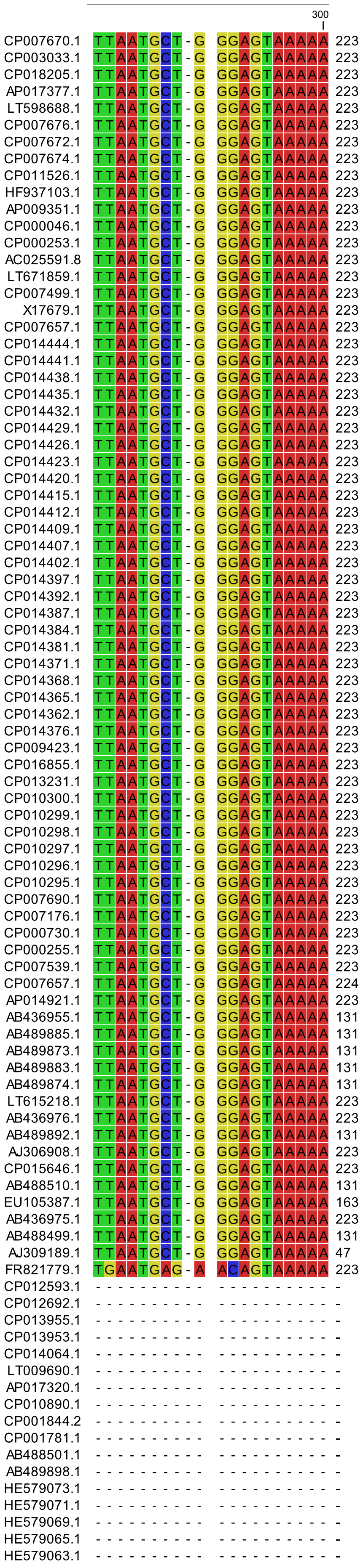
300
CP007670.1 TTAATGCT-G GGAGTAAAAA 223
CP003033.1 TTAATGCT-G GGAGTAAAAA 223
CP018205.1 TTAATGCT-G GGAGTAAAAA 223
AP017377.1 TTAATGCT-G GGAGTAAAAA 223
LT598688.1 TTAATGCT-G GGAGTAAAAA 223
CP007676.1 TTAATGCT-G GGAGTAAAAA 223
CP007672.1 TTAATGCT-G GGAGTAAAAA 223
CP007674.1 TTAATGCT-G GGAGTAAAAA 223
CP011526.1 TTAATGCT-G GGAGTAAAAA 223
HF937103.1 TTAATGCT-G GGAGTAAAAA 223
AP009351.1 TTAATGCT-G GGAGTAAAAA 223
CP000046.1 TTAATGCT-G GGAGTAAAAA 223
CP000253.1 TTAATGCT-G GGAGTAAAAA 223
AC025591.8 TTAATGCT-G GGAGTAAAAA 223
LT671859.1 TTAATGCT-G GGAGTAAAAA 223
CP007499.1 TTAATGCT-G GGAGTAAAAA 223
X17679.1 TTAATGCT-G GGAGTAAAAA 223
CP007657.1 TTAATGCT-G GGAGTAAAAA 223
CP014444.1 TTAATGCT-G GGAGTAAAAA 223
CP014441.1 TTAATGCT-G GGAGTAAAAA 223
CP014438.1 TTAATGCT-G GGAGTAAAAA 223
CP014435.1 TTAATGCT-G GGAGTAAAAA 223
CP014432.1 TTAATGCT-G GGAGTAAAAA 223
CP014429.1 TTAATGCT-G GGAGTAAAAA 223
CP014426.1 TTAATGCT-G GGAGTAAAAA 223
CP014423.1 TTAATGCT-G GGAGTAAAAA 223
CP014420.1 TTAATGCT-G GGAGTAAAAA 223
CP014415.1 TTAATGCT-G GGAGTAAAAA 223
CP014412.1 TTAATGCT-G GGAGTAAAAA 223
CP014409.1 TTAATGCT-G GGAGTAAAAA 223
CP014407.1 TTAATGCT-G GGAGTAAAAA 223
CP014402.1 TTAATGCT-G GGAGTAAAAA 223
CP014397.1 TTAATGCT-G GGAGTAAAAA 223
CP014392.1 TTAATGCT-G GGAGTAAAAA 223
CP014387.1 TTAATGCT-G GGAGTAAAAA 223
CP014384.1 TTAATGCT-G GGAGTAAAAA 223
CP014381.1 TTAATGCT-G GGAGTAAAAA 223
CP014371.1 TTAATGCT-G GGAGTAAAAA 223
CP014368.1 TTAATGCT-G GGAGTAAAAA 223
CP014365.1 TTAATGCT-G GGAGTAAAAA 223
CP014362.1 TTAATGCT-G GGAGTAAAAA 223
CP014376.1 TTAATGCT-G GGAGTAAAAA 223
CP009423.1 TTAATGCT-G GGAGTAAAAA 223
CP016855.1 TTAATGCT-G GGAGTAAAAA 223
CP013231.1 TTAATGCT-G GGAGTAAAAA 223
CP010300.1 TTAATGCT-G GGAGTAAAAA 223
CP010299.1 TTAATGCT-G GGAGTAAAAA 223
CP010298.1 TTAATGCT-G GGAGTAAAAA 223
CP010297.1 TTAATGCT-G GGAGTAAAAA 223
CP010296.1 TTAATGCT-G GGAGTAAAAA 223
CP010295.1 TTAATGCT-G GGAGTAAAAA 223
CP007690.1 TTAATGCT-G GGAGTAAAAA 223
CP007176.1 TTAATGCT-G GGAGTAAAAA 223
CP000730.1 TTAATGCT-G GGAGTAAAAA 223
CP000255.1 TTAATGCT-G GGAGTAAAAA 223
CP007539.1 TTAATGCT-G GGAGTAAAAA 223
CP007657.1 TTAATGCT-G GGAGTAAAAA 224
AP014921.1 TTAATGCT-G GGAGTAAAAA 223
AB436955.1 TTAATGCT-G GGAGTAAAAA 131
AB489885.1 TTAATGCT-G GGAGTAAAAA 131
AB489873.1 TTAATGCT-G GGAGTAAAAA 131
AB489883.1 TTAATGCT-G GGAGTAAAAA 131
AB489874.1 TTAATGCT-G GGAGTAAAAA 131
LT615218.1 TTAATGCT-G GGAGTAAAAA 223
AB436976.1 TTAATGCT-G GGAGTAAAAA 223
AB489892.1 TTAATGCT-G GGAGTAAAAA 131
AJ306908.1 TTAATGCT-G GGAGTAAAAA 223
CP015646.1 TTAATGCT-G GGAGTAAAAA 223
AB488510.1 TTAATGCT-G GGAGTAAAAA 131
EU105387.1 TTAATGCT-G GGAGTAAAAA 163
AB436975.1 TTAATGCT-G GGAGTAAAAA 223
AB488499.1 TTAATGCT-G GGAGTAAAAA 131
AJ309189.1 TTAATGCT-G GGAGTAAAAA 47
FR821779.1 TGAATGAG-A ACAGTAAAAA 223
CP012593.1 ---------- ---------- -
CP012692.1 ---------- ---------- -
CP013955.1 ---------- ---------- -
CP013953.1 ---------- ---------- -
CP014064.1 ---------- ---------- -
LT009690.1 ---------- ---------- -
AP017320.1 ---------- ---------- -
CP010890.1 ---------- ---------- -
CP001844.2 ---------- ---------- -
CP001781.1 ---------- ---------- -
AB488501.1 ---------- ---------- -
AB489898.1 ---------- ---------- -
HE579073.1 ---------- ---------- -
HE579071.1 ---------- ---------- -
HE579069.1 ---------- ---------- -
HE579065.1 ---------- ---------- -
HE579063.1 ---------- ---------- -

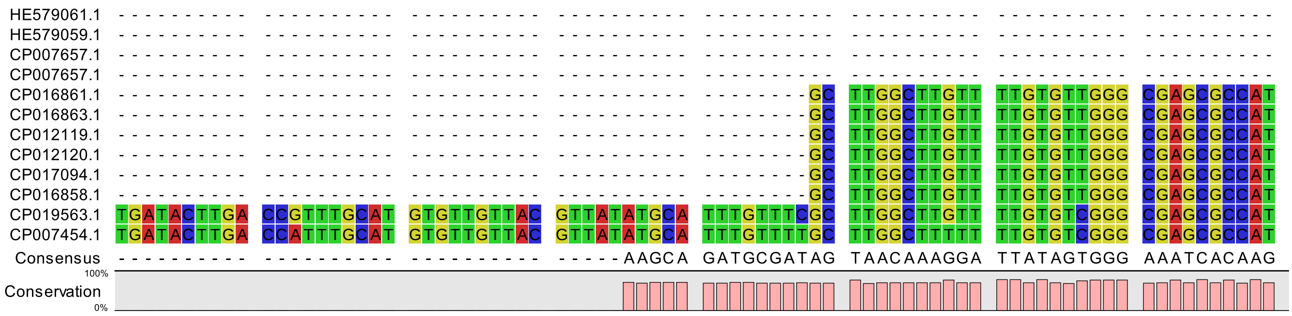
HE579061.1 ---------- ---------- ---------- ---------- ---------- ---------- ---------- ----------
HE579059.1 ---------- ---------- ---------- ---------- ---------- ---------- ---------- ----------
CP007657.1 ---------- ---------- ---------- ---------- ---------- ---------- ---------- ----------
CP007657.1 ---------- ---------- ---------- ---------- ---------- ---------- ---------- ----------
CP016861.1 ---------- ---------- ---------- ---------- --------GC TTGGCTTGTT TTGTGTTGGG CGAGCGCCAT
CP016863.1 ---------- ---------- ---------- ---------- --------GC TTGGCTTGTT TTGTGTTGGG CGAGCGCCAT
CP012119.1 ---------- ---------- ---------- ---------- --------GC TTGGCTTGTT TTGTGTTGGG CGAGCGCCAT
CP012120.1 ---------- ---------- ---------- ---------- --------GC TTGGCTTGTT TTGTGTTGGG CGAGCGCCAT
CP017094.1 ---------- ---------- ---------- ---------- --------GC TTGGCTTGTT TTGTGTTGGG CGAGCGCCAT
CP016858.1 ---------- ---------- ---------- ---------- --------GC TTGGCTTGTT TTGTGTTGGG CGAGCGCCAT
CP019563.1 TGATACTTGA CCGTTTGCAT GTGTTGTTAC GTTATATGCA TTTGTTTCGC TTGGCTTGTT TTGTGTCGGG CGAGCGCCAT
CP007454.1 TGATACTTGA CCATTTGCAT GTGTTGTTAC GTTATATGCA TTTGTTTTGC TTGGCTTTTT TTGTGTCGGG CGAGCGCCAT
Consensus ---------- ---------- ---------- -----AAGCA GATGCGATAG TAACAAAGGA TTATAGTGGG AAATCACAAG
100%
Conservation
0%

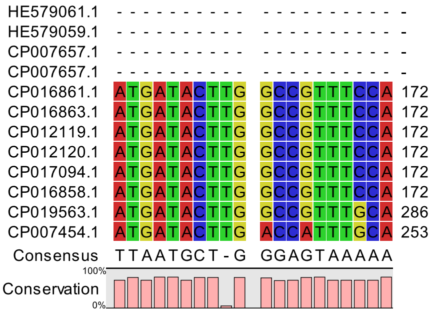
HE579061.1 -
HE579059.1 -
CP007657.1 -
CP007657.1 -
CP016861.1 ATGATACTTG GCCGTTTCCA 172
CP016863.1 ATGATACTTG GCCGTTTCCA 172
CP012119.1 ATGATACTTG GCCGTTTCCA 172
CP012120.1 ATGATACTTG GCCGTTTCCA 172
CP017094.1 ATGATACTTG GCCGTTTCCA 172
CP016858.1 ATGATACTTG GCCGTTTCCA 172
CP019563.1 ATGATACTTG GCCGTTTGCA 286
CP007454.1 ATGATACTTG ACCATTTGCA 253
Consensus TTAATGCT-G GGAGTAAAAA
100%
Conservation
0%

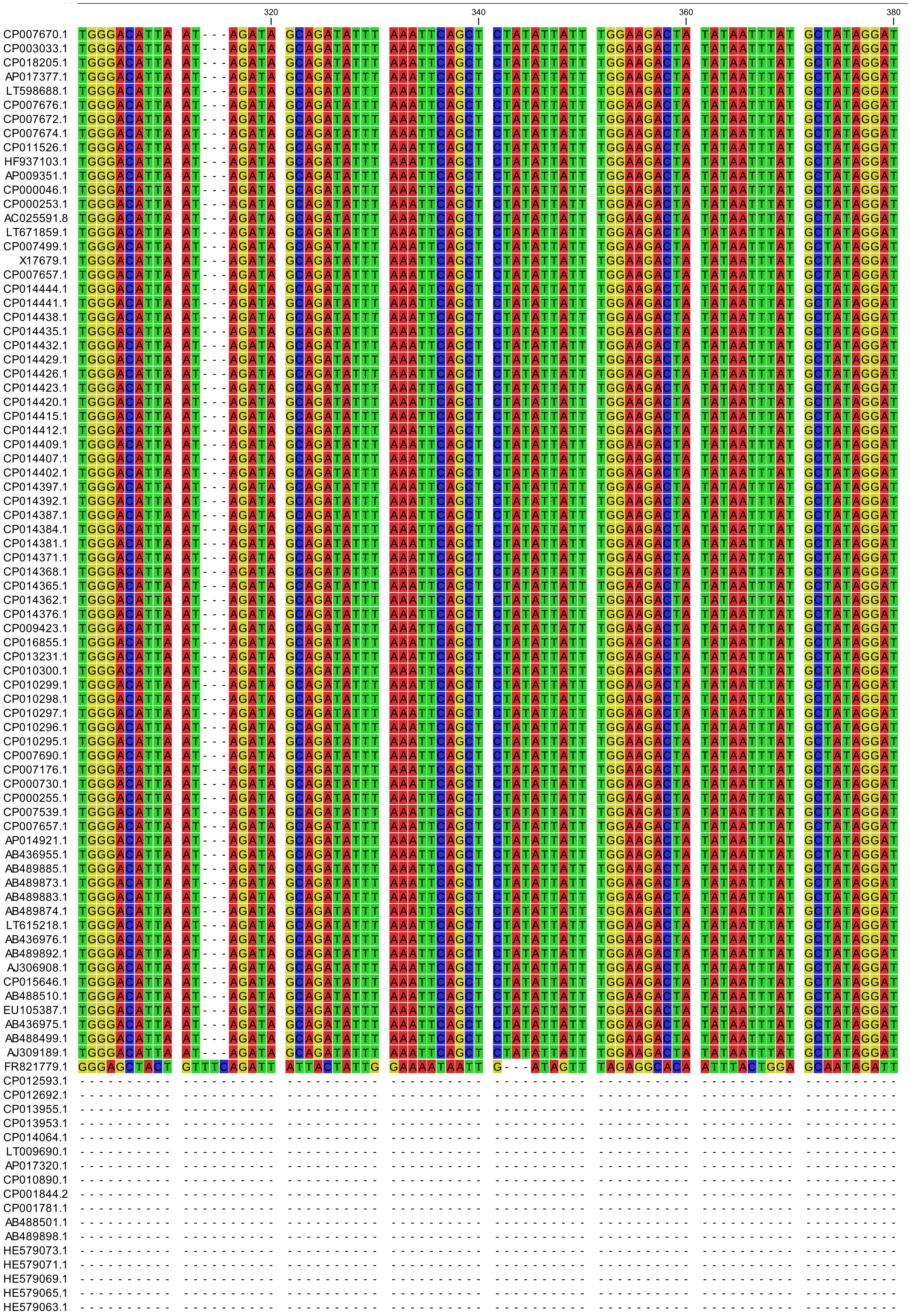
320
340
360
380
CP007670.1 TGGGACATTA AT---AGATA GCAGATATTT AAATTCAGCT CTATATTATT TGGAAGACTA TATAATTTAT GCTATAGGAT
CP003033.1 TGGGACATTA AT---AGATA GCAGATATTT AAATTCAGCT CTATATTATT TGGAAGACTA TATAATTTAT GCTATAGGAT
CP018205.1 TGGGACATTA AT---AGATA GCAGATATTT AAATTCAGCT CTATATTATT TGGAAGACTA TATAATTTAT GCTATAGGAT
AP017377.1 TGGGACATTA AT---AGATA GCAGATATTT AAATTCAGCT CTATATTATT TGGAAGACTA TATAATTTAT GCTATAGGAT
LT598688.1 TGGGACATTA AT---AGATA GCAGATATTT AAATTCAGCT CTATATTATT TGGAAGACTA TATAATTTAT GCTATAGGAT
CP007676.1 TGGGACATTA AT---AGATA GCAGATATTT AAATTCAGCT CTATATTATT TGGAAGACTA TATAATTTAT GCTATAGGAT
CP007672.1 TGGGACATTA AT---AGATA GCAGATATTT AAATTCAGCT CTATATTATT TGGAAGACTA TATAATTTAT GCTATAGGAT
CP007674.1 TGGGACATTA AT---AGATA GCAGATATTT AAATTCAGCT CTATATTATT TGGAAGACTA TATAATTTAT GCTATAGGAT
CP011526.1 TGGGACATTA AT---AGATA GCAGATATTT AAATTCAGCT CTATATTATT TGGAAGACTA TATAATTTAT GCTATAGGAT
HF937103.1 TGGGACATTA AT---AGATA GCAGATATTT AAATTCAGCT CTATATTATT TGGAAGACTA TATAATTTAT GCTATAGGAT
AP009351.1 TGGGACATTA AT---AGATA GCAGATATTT AAATTCAGCT CTATATTATT TGGAAGACTA TATAATTTAT GCTATAGGAT
CP000046.1 TGGGACATTA AT---AGATA GCAGATATTT AAATTCAGCT CTATATTATT TGGAAGACTA TATAATTTAT GCTATAGGAT
CP000253.1 TGGGACATTA AT---AGATA GCAGATATTT AAATTCAGCT CTATATTATT TGGAAGACTA TATAATTTAT GCTATAGGAT
AC025591.8 TGGGACATTA AT---AGATA GCAGATATTT AAATTCAGCT CTATATTATT TGGAAGACTA TATAATTTAT GCTATAGGAT
LT671859.1 TGGGACATTA AT---AGATA GCAGATATTT AAATTCAGCT CTATATTATT TGGAAGACTA TATAATTTAT GCTATAGGAT
CP007499.1 TGGGACATTA AT---AGATA GCAGATATTT AAATTCAGCT CTATATTATT TGGAAGACTA TATAATTTAT GCTATAGGAT
X17679.1 TGGGACATTA AT---AGATA GCAGATATTT AAATTCAGCT CTATATTATT TGGAAGACTA TATAATTTAT GCTATAGGAT
CP007657.1 TGGGACATTA AT---AGATA GCAGATATTT AAATTCAGCT CTATATTATT TGGAAGACTA TATAATTTAT GCTATAGGAT
CP014444.1 TGGGACATTA AT---AGATA GCAGATATTT AAATTCAGCT CTATATTATT TGGAAGACTA TATAATTTAT GCTATAGGAT
CP014441.1 TGGGACATTA AT---AGATA GCAGATATTT AAATTCAGCT CTATATTATT TGGAAGACTA TATAATTTAT GCTATAGGAT
CP014438.1 TGGGACATTA AT---AGATA GCAGATATTT AAATTCAGCT CTATATTATT TGGAAGACTA TATAATTTAT GCTATAGGAT
CP014435.1 TGGGACATTA AT---AGATA GCAGATATTT AAATTCAGCT CTATATTATT TGGAAGACTA TATAATTTAT GCTATAGGAT
CP014432.1 TGGGACATTA AT---AGATA GCAGATATTT AAATTCAGCT CTATATTATT TGGAAGACTA TATAATTTAT GCTATAGGAT
CP014429.1 TGGGACATTA AT---AGATA GCAGATATTT AAATTCAGCT CTATATTATT TGGAAGACTA TATAATTTAT GCTATAGGAT
CP014426.1 TGGGACATTA AT---AGATA GCAGATATTT AAATTCAGCT CTATATTATT TGGAAGACTA TATAATTTAT GCTATAGGAT
CP014423.1 TGGGACATTA AT---AGATA GCAGATATTT AAATTCAGCT CTATATTATT TGGAAGACTA TATAATTTAT GCTATAGGAT
CP014420.1 TGGGACATTA AT---AGATA GCAGATATTT AAATTCAGCT CTATATTATT TGGAAGACTA TATAATTTAT GCTATAGGAT
CP014415.1 TGGGACATTA AT---AGATA GCAGATATTT AAATTCAGCT CTATATTATT TGGAAGACTA TATAATTTAT GCTATAGGAT
CP014412.1 TGGGACATTA AT---AGATA GCAGATATTT AAATTCAGCT CTATATTATT TGGAAGACTA TATAATTTAT GCTATAGGAT
CP014409.1 TGGGACATTA AT---AGATA GCAGATATTT AAATTCAGCT CTATATTATT TGGAAGACTA TATAATTTAT GCTATAGGAT
CP014407.1 TGGGACATTA AT---AGATA GCAGATATTT AAATTCAGCT CTATATTATT TGGAAGACTA TATAATTTAT GCTATAGGAT
CP014402.1 TGGGACATTA AT---AGATA GCAGATATTT AAATTCAGCT CTATATTATT TGGAAGACTA TATAATTTAT GCTATAGGAT
CP014397.1 TGGGACATTA AT---AGATA GCAGATATTT AAATTCAGCT CTATATTATT TGGAAGACTA TATAATTTAT GCTATAGGAT
CP014392.1 TGGGACATTA AT---AGATA GCAGATATTT AAATTCAGCT CTATATTATT TGGAAGACTA TATAATTTAT GCTATAGGAT
CP014387.1 TGGGACATTA AT---AGATA GCAGATATTT AAATTCAGCT CTATATTATT TGGAAGACTA TATAATTTAT GCTATAGGAT
CP014384.1 TGGGACATTA AT---AGATA GCAGATATTT AAATTCAGCT CTATATTATT TGGAAGACTA TATAATTTAT GCTATAGGAT
CP014381.1 TGGGACATTA AT---AGATA GCAGATATTT AAATTCAGCT CTATATTATT TGGAAGACTA TATAATTTAT GCTATAGGAT
CP014371.1 TGGGACATTA AT---AGATA GCAGATATTT AAATTCAGCT CTATATTATT TGGAAGACTA TATAATTTAT GCTATAGGAT
CP014368.1 TGGGACATTA AT---AGATA GCAGATATTT AAATTCAGCT CTATATTATT TGGAAGACTA TATAATTTAT GCTATAGGAT
CP014365.1 TGGGACATTA AT---AGATA GCAGATATTT AAATTCAGCT CTATATTATT TGGAAGACTA TATAATTTAT GCTATAGGAT
CP014362.1 TGGGACATTA AT---AGATA GCAGATATTT AAATTCAGCT CTATATTATT TGGAAGACTA TATAATTTAT GCTATAGGAT
CP014376.1 TGGGACATTA AT---AGATA GCAGATATTT AAATTCAGCT CTATATTATT TGGAAGACTA TATAATTTAT GCTATAGGAT
CP009423.1 TGGGACATTA AT---AGATA GCAGATATTT AAATTCAGCT CTATATTATT TGGAAGACTA TATAATTTAT GCTATAGGAT
CP016855.1 TGGGACATTA AT---AGATA GCAGATATTT AAATTCAGCT CTATATTATT TGGAAGACTA TATAATTTAT GCTATAGGAT
CP013231.1 TGGGACATTA AT---AGATA GCAGATATTT AAATTCAGCT CTATATTATT TGGAAGACTA TATAATTTAT GCTATAGGAT
CP010300.1 TGGGACATTA AT---AGATA GCAGATATTT AAATTCAGCT CTATATTATT TGGAAGACTA TATAATTTAT GCTATAGGAT
CP010299.1 TGGGACATTA AT---AGATA GCAGATATTT AAATTCAGCT CTATATTATT TGGAAGACTA TATAATTTAT GCTATAGGAT
CP010298.1 TGGGACATTA AT---AGATA GCAGATATTT AAATTCAGCT CTATATTATT TGGAAGACTA TATAATTTAT GCTATAGGAT
CP010297.1 TGGGACATTA AT---AGATA GCAGATATTT AAATTCAGCT CTATATTATT TGGAAGACTA TATAATTTAT GCTATAGGAT
CP010296.1 TGGGACATTA AT---AGATA GCAGATATTT AAATTCAGCT CTATATTATT TGGAAGACTA TATAATTTAT GCTATAGGAT
CP010295.1 TGGGACATTA AT---AGATA GCAGATATTT AAATTCAGCT CTATATTATT TGGAAGACTA TATAATTTAT GCTATAGGAT
CP007690.1 TGGGACATTA AT---AGATA GCAGATATTT AAATTCAGCT CTATATTATT TGGAAGACTA TATAATTTAT GCTATAGGAT
CP007176.1 TGGGACATTA AT---AGATA GCAGATATTT AAATTCAGCT CTATATTATT TGGAAGACTA TATAATTTAT GCTATAGGAT
CP000730.1 TGGGACATTA AT---AGATA GCAGATATTT AAATTCAGCT CTATATTATT TGGAAGACTA TATAATTTAT GCTATAGGAT
CP000255.1 TGGGACATTA AT---AGATA GCAGATATTT AAATTCAGCT CTATATTATT TGGAAGACTA TATAATTTAT GCTATAGGAT
CP007539.1 TGGGACATTA AT---AGATA GCAGATATTT AAATTCAGCT CTATATTATT TGGAAGACTA TATAATTTAT GCTATAGGAT
CP007657.1 TGGGACATTA AT---AGATA GCAGATATTT AAATTCAGCT CTATATTATT TGGAAGACTA TATAATTTAT GCTATAGGAT
AP014921.1 TGGGACATTA AT---AGATA GCAGATATTT AAATTCAGCT CTATATTATT TGGAAGACTA TATAATTTAT GCTATAGGAT
AB436955.1 TGGGACATTA AT---AGATA GCAGATATTT AAATTCAGCT CTATATTATT TGGAAGACTA TATAATTTAT GCTATAGGAT
AB489885.1 TGGGACATTA AT---AGATA GCAGATATTT AAATTCAGCT CTATATTATT TGGAAGACTA TATAATTTAT GCTATAGGAT
AB489873.1 TGGGACATTA AT---AGATA GCAGATATTT AAATTCAGCT CTATATTATT TGGAAGACTA TATAATTTAT GCTATAGGAT
AB489883.1 TGGGACATTA AT---AGATA GCAGATATTT AAATTCAGCT CTATATTATT TGGAAGACTA TATAATTTAT GCTATAGGAT
AB489874.1 TGGGACATTA AT---AGATA GCAGATATTT AAATTCAGCT CTATATTATT TGGAAGACTA TATAATTTAT GCTATAGGAT
LT615218.1 TGGGACATTA AT---AGATA GCAGATATTT AAATTCAGCT CTATATTATT TGGAAGACTA TATAATTTAT GCTATAGGAT
AB436976.1 TGGGACATTA AT---AGATA GCAGATATTT AAATTCAGCT CTATATTATT TGGAAGACTA TATAATTTAT GCTATAGGAT
AB489892.1 TGGGACATTA AT---AGATA GCAGATATTT AAATTCAGCT CTATATTATT TGGAAGACTA TATAATTTAT GCTATAGGAT
AJ306908.1 TGGGACATTA AT---AGATA GCAGATATTT AAATTCAGCT CTATATTATT TGGAAGACTA TATAATTTAT GCTATAGGAT
CP015646.1 TGGGACATTA AT---AGATA GCAGATATTT AAATTCAGCT CTATATTATT TGGAAGACTA TATAATTTAT GCTATAGGAT
AB488510.1 TGGGACATTA AT---AGATA GCAGATATTT AAATTCAGCT CTATATTATT TGGAAGACTA TATAATTTAT GCTATAGGAT
EU105387.1 TGGGACATTA AT---AGATA GCAGATATTT AAATTCAGCT CTATATTATT TGGAAGACTA TATAATTTAT GCTATAGGAT
AB436975.1 TGGGACATTA AT---AGATA GCAGATATTT AAATTCAGCT CTATATTATT TGGAAGACTA TATAATTTAT GCTATAGGAT
AB488499.1 TGGGACATTA AT---AGATA GCAGATATTT AAATTCAGCT CTATATTATT TGGAAGACTA TATAATTTAT GCTATAGGAT
AJ309189.1 TGGGACATTA AT---AGATA GCAGATATTT AAATTCAGCT CTATATTATT TGGAAGACTA TATAATTTAT GCTATAGGAT
FR821779.1 GGGAGCTACT GTTTCAGATT ATTACTATTG GAAAATAATT G---ATAGTT TAGAGGCACA ATTTACTGGA GCAATAGATT
CP012593.1 ---------- ---------- ---------- ---------- ---------- ---------- ---------- ----------
CP012692.1 ---------- ---------- ---------- ---------- ---------- ---------- ---------- ----------
CP013955.1 ---------- ---------- ---------- ---------- ---------- ---------- ---------- ----------
CP013953.1 ---------- ---------- ---------- ---------- ---------- ---------- ---------- ----------
CP014064.1 ---------- ---------- ---------- ---------- ---------- ---------- ---------- ----------
LT009690.1 ---------- ---------- ---------- ---------- ---------- ---------- ---------- ----------
AP017320.1 ---------- ---------- ---------- ---------- ---------- ---------- ---------- ----------
CP010890.1 ---------- ---------- ---------- ---------- ---------- ---------- ---------- ----------
CP001844.2 ---------- ---------- ---------- ---------- ---------- ---------- ---------- ----------
CP001781.1 ---------- ---------- ---------- ---------- ---------- ---------- ---------- ----------
AB488501.1 ---------- ---------- ---------- ---------- ---------- ---------- ---------- ----------
AB489898.1 ---------- ---------- ---------- ---------- ---------- ---------- ---------- ----------
HE579073.1 ---------- ---------- ---------- ---------- ---------- ---------- ---------- ----------
HE579071.1 ---------- ---------- ---------- ---------- ---------- ---------- ---------- ----------
HE579069.1 ---------- ---------- ---------- ---------- ---------- ---------- ---------- ----------
HE579065.1 ---------- ---------- ---------- ---------- ---------- ---------- ---------- ----------
HE579063.1 ---------- ---------- ---------- ---------- ---------- ---------- ---------- ----------

		400	
CP007670.1	TAACTAATAA	ATATGAATAT	320
CP003033.1	TAACTAATAA	ATATGAATAT	320
CP018205.1	TAACTAATAA	ATATGAATAT	320
AP017377.1	TAACTAATAA	ATATGAATAT	320
LT598688.1	TAACTAATAA	ATATGAATAT	320
CP007676.1	TAACTAATAA	ATATGAATAT	320
CP007672.1	TAACTAATAA	ATATGAATAT	320
CP007674.1	TAACTAATAA	ATATGAATAT	320
CP011526.1	TAACTAATAA	ATATGAATAT	320
HF937103.1	TAACTAATAA	ATATGAATAT	320
AP009351.1	TAACTAATAA	ATATGAATAT	320
CP000046.1	TAACTAATAA	ATATGAATAT	320
CP000253.1	TAACTAATAA	ATATGAATAT	320
AC025591.8	TAACTAATAA	ATATGAATAT	320
LT671859.1	TAACTAATAA	ATATGAATAT	320
CP007499.1	TAACTAATAA	ATATGAATAT	320
X17679.1	TAACTAATAA	ATATGAATAT	320
CP007657.1	TAACTAATAA	ATATGAATAT	320
CP014444.1	TAACTAATAA	ATATGAATAT	320
CP014441.1	TAACTAATAA	ATATGAATAT	320
CP014438.1	TAACTAATAA	ATATGAATAT	320
CP014435.1	TAACTAATAA	ATATGAATAT	320
CP014432.1	TAACTAATAA	ATATGAATAT	320
CP014429.1	TAACTAATAA	ATATGAATAT	320
CP014426.1	TAACTAATAA	ATATGAATAT	320
CP014423.1	TAACTAATAA	ATATGAATAT	320
CP014420.1	TAACTAATAA	ATATGAATAT	320
CP014415.1	TAACTAATAA	ATATGAATAT	320
CP014412.1	TAACTAATAA	ATATGAATAT	320
CP014409.1	TAACTAATAA	ATATGAATAT	320
CP014407.1	TAACTAATAA	ATATGAATAT	320
CP014402.1	TAACTAATAA	ATATGAATAT	320
CP014397.1	TAACTAATAA	ATATGAATAT	320
CP014392.1	TAACTAATAA	ATATGAATAT	320
CP014387.1	TAACTAATAA	ATATGAATAT	320
CP014384.1	TAACTAATAA	ATATGAATAT	320
CP014381.1	TAACTAATAA	ATATGAATAT	320
CP014371.1	TAACTAATAA	ATATGAATAT	320
CP014368.1	TAACTAATAA	ATATGAATAT	320
CP014365.1	TAACTAATAA	ATATGAATAT	320
CP014362.1	TAACTAATAA	ATATGAATAT	320
CP014376.1	TAACTAATAA	ATATGAATAT	320
CP009423.1	TAACTAATAA	ATATGAATAT	320
CP016855.1	TAACTAATAA	ATATGAATAT	320
CP013231.1	TAACTAATAA	ATATGAATAT	320
CP010300.1	TAACTAATAA	ATATGAATAT	320
CP010299.1	TAACTAATAA	ATATGAATAT	320
CP010298.1	TAACTAATAA	ATATGAATAT	320
CP010297.1	TAACTAATAA	ATATGAATAT	320
CP010296.1	TAACTAATAA	ATATGAATAT	320
CP010295.1	TAACTAATAA	ATATGAATAT	320
CP007690.1	TAACTAATAA	ATATGAATAT	320
CP007176.1	TAACTAATAA	ATATGAATAT	320
CP000730.1	TAACTAATAA	ATATGAATAT	320
CP000255.1	TAACTAATAA	ATATGAATAT	320
CP007539.1	TAACTAATAA	ATATGAATAT	320
CP007657.1	TAACTAATAA	ATATGAATAT	321
AP014921.1	TAACTAATAA	ATATGAATAT	320
AB436955.1	TAACTAATAA	ATATGAATAT	228
AB489885.1	TAACTAATAA	ATATGAATAT	228
AB489873.1	TAACTAATAA	ATATGAATAT	228
AB489883.1	TAACTAATAA	ATATGAATAT	228
AB489874.1	TAACTAATAA	ATATGAATAT	228
LT615218.1	TAACTAATAA	ATATGAATAT	320
AB436976.1	TAACTAATAA	ATATGAATAT	320
AB489892.1	TAACTAATAA	ATATGAATAT	228
AJ306908.1	TAACTAATAA	ATATGAATAT	320
CP015646.1	TAACTAATAA	ATATGAATAT	320
AB488510.1	TAACTAATAA	ATATGAATAT	228
EU105387.1	TAACTAATAA	ATATGAATAT	260
AB436975.1	TAACTAATAA	ATATGAATAT	320
AB488499.1	TAACTAATAA	ATATGAATAT	228
AJ309189.1	TAACTAATAA	ATATGAATAT	144
FR821779.1	TATTGGAAGA	TTATAAATAT	320
CP012593.1	- - - - - - - - - -	- - - - - - - - - -	-
CP012692.1	- - - - - - - - - -	- - - - - - - - - -	-
CP013955.1	- - - - - - - - - -	- - - - - - - - - -	-
CP013953.1	- - - - - - - - - -	- - - - - - - - - -	-
CP014064.1	- - - - - - - - - -	- - - - - - - - - -	-
LT009690.1	- - - - - - - - - -	- - - - - - - - - -	-
AP017320.1	- - - - - - - - - -	- - - - - - - - - -	-
CP010890.1	- - - - - - - - - -	- - - - - - - - - -	-
CP001844.2	- - - - - - - - - -	- - - - - - - - - -	-
CP001781.1	- - - - - - - - - -	- - - - - - - - - -	-
AB488501.1	- - - - - - - - - -	- - - - - - - - - -	-
AB489898.1	- - - - - - - - - -	- - - - - - - - - -	-
HE579073.1	- - - - - - - - - -	- - - - - - - - - -	-
HE579071.1	- - - - - - - - - -	- - - - - - - - - -	-
HE579069.1	- - - - - - - - - -	- - - - - - - - - -	-
HE579065.1	- - - - - - - - - -	- - - - - - - - - -	-
HE579063.1	- - - - - - - - - -	- - - - - - - - - -	-

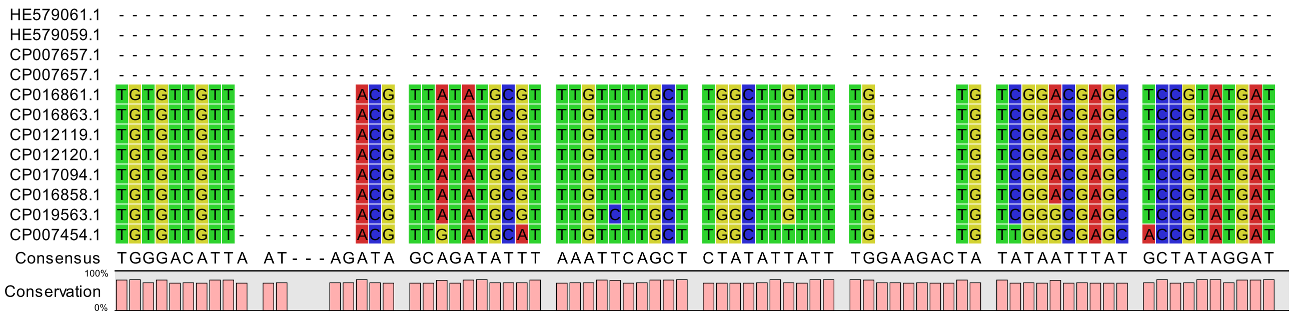
HE579061.1 ---------- ---------- ---------- ---------- ---------- ---------- ---------- ----------
HE579059.1 ---------- ---------- ---------- ---------- ---------- ---------- ---------- ----------
CP007657.1 ---------- ---------- ---------- ---------- ---------- ---------- ---------- ----------
CP007657.1 ---------- ---------- ---------- ---------- ---------- ---------- ---------- ----------
CP016861.1 TGTGTTGTT- -------ACG TTATATGCGT TTGTTTTGCT TGGCTTGTTT TG------TG TCGGACGAGC TCCGTATGAT
CP016863.1 TGTGTTGTT- -------ACG TTATATGCGT TTGTTTTGCT TGGCTTGTTT TG------TG TCGGACGAGC TCCGTATGAT
CP012119.1 TGTGTTGTT- -------ACG TTATATGCGT TTGTTTTGCT TGGCTTGTTT TG------TG TCGGACGAGC TCCGTATGAT
CP012120.1 TGTGTTGTT- -------ACG TTATATGCGT TTGTTTTGCT TGGCTTGTTT TG------TG TCGGACGAGC TCCGTATGAT
CP017094.1 TGTGTTGTT- -------ACG TTATATGCGT TTGTTTTGCT TGGCTTGTTT TG------TG TCGGACGAGC TCCGTATGAT
CP016858.1 TGTGTTGTT- -------ACG TTATATGCGT TTGTTTTGCT TGGCTTGTTT TG------TG TCGGACGAGC TCCGTATGAT
CP019563.1 TGTGTTGTT- -------ACG TTATATGCGT TTGTCTTGCT TGGCTTGTTT TG------TG TCGGGCGAGC TCCGTATGAT
CP007454.1 TGTGTTGTT- -------ACG TTGTATGCAT TTGTTTTGCT TGGCTTTTTT TG------TG TTGGGCGAGC ACCGTATGAT
Consensus TGGGACATTA AT---AGATA GCAGATATTT AAATTCAGCT CTATATTATT TGGAAGACTA TATAATTTAT GCTATAGGAT
100%
Conservation
0%

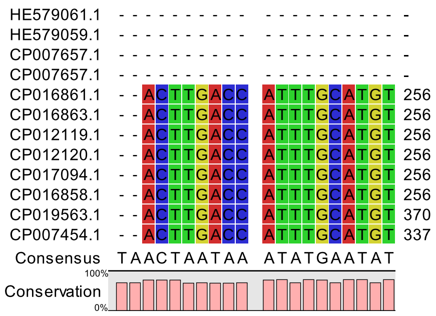
HE579061.1 -
HE579059.1 -
CP007657.1 -
CP007657.1 -
CP016861.1 - - ACTTGACC ATTTGCATGT 256
CP016863.1 - - ACTTGACC ATTTGCATGT 256
CP012119.1 - - ACTTGACC ATTTGCATGT 256
CP012120.1 - - ACTTGACC ATTTGCATGT 256
CP017094.1 - - ACTTGACC ATTTGCATGT 256
CP016858.1 - - ACTTGACC ATTTGCATGT 256
CP019563.1 - - ACTTGACC ATTTGCATGT 370
CP007454.1 - - ACTTGACC ATTTGCATGT 337
Consensus TAACTAATAA ATATGAATAT
100%
Conservation
0%

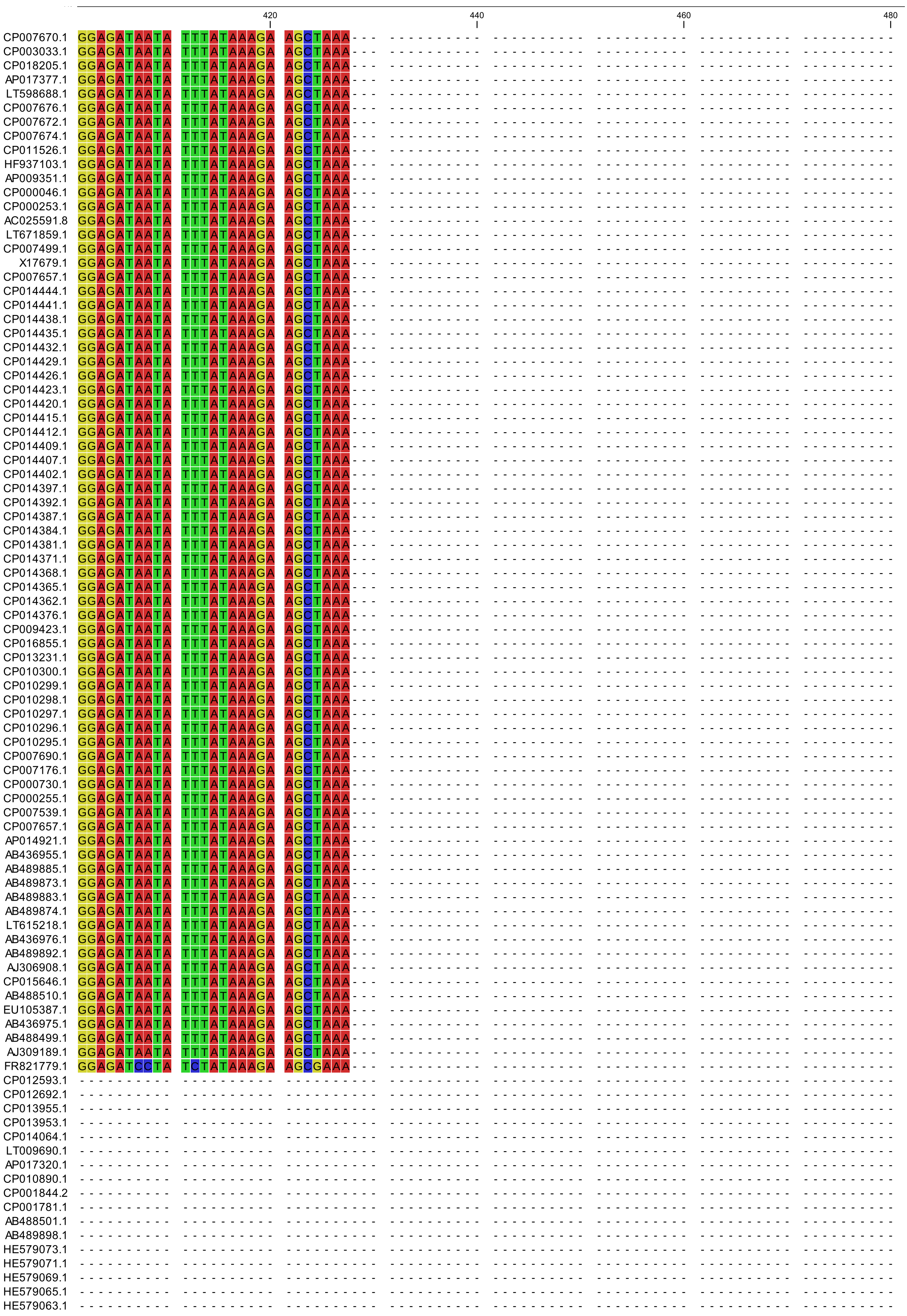

420
440
460
480
CP007670.1 GGAGATAATA TTTATAAAGA AGCTAAA--- ---------- ---------- ---------- ---------- ----------
CP003033.1 GGAGATAATA TTTATAAAGA AGCTAAA--- ---------- ---------- ---------- ---------- ----------
CP018205.1 GGAGATAATA TTTATAAAGA AGCTAAA--- ---------- ---------- ---------- ---------- ----------
AP017377.1 GGAGATAATA TTTATAAAGA AGCTAAA--- ---------- ---------- ---------- ---------- ----------
LT598688.1 GGAGATAATA TTTATAAAGA AGCTAAA--- ---------- ---------- ---------- ---------- ----------
CP007676.1 GGAGATAATA TTTATAAAGA AGCTAAA--- ---------- ---------- ---------- ---------- ----------
CP007672.1 GGAGATAATA TTTATAAAGA AGCTAAA--- ---------- ---------- ---------- ---------- ----------
CP007674.1 GGAGATAATA TTTATAAAGA AGCTAAA--- ---------- ---------- ---------- ---------- ----------
CP011526.1 GGAGATAATA TTTATAAAGA AGCTAAA--- ---------- ---------- ---------- ---------- ----------
HF937103.1 GGAGATAATA TTTATAAAGA AGCTAAA--- ---------- ---------- ---------- ---------- ----------
AP009351.1 GGAGATAATA TTTATAAAGA AGCTAAA--- ---------- ---------- ---------- ---------- ----------
CP000046.1 GGAGATAATA TTTATAAAGA AGCTAAA--- ---------- ---------- ---------- ---------- ----------
CP000253.1 GGAGATAATA TTTATAAAGA AGCTAAA--- ---------- ---------- ---------- ---------- ----------
AC025591.8 GGAGATAATA TTTATAAAGA AGCTAAA--- ---------- ---------- ---------- ---------- ----------
LT671859.1 GGAGATAATA TTTATAAAGA AGCTAAA--- ---------- ---------- ---------- ---------- ----------
CP007499.1 GGAGATAATA TTTATAAAGA AGCTAAA--- ---------- ---------- ---------- ---------- ----------
X17679.1 GGAGATAATA TTTATAAAGA AGCTAAA--- ---------- ---------- ---------- ---------- ----------
CP007657.1 GGAGATAATA TTTATAAAGA AGCTAAA--- ---------- ---------- ---------- ---------- ----------
CP014444.1 GGAGATAATA TTTATAAAGA AGCTAAA--- ---------- ---------- ---------- ---------- ----------
CP014441.1 GGAGATAATA TTTATAAAGA AGCTAAA--- ---------- ---------- ---------- ---------- ----------
CP014438.1 GGAGATAATA TTTATAAAGA AGCTAAA--- ---------- ---------- ---------- ---------- ----------
CP014435.1 GGAGATAATA TTTATAAAGA AGCTAAA--- ---------- ---------- ---------- ---------- ----------
CP014432.1 GGAGATAATA TTTATAAAGA AGCTAAA--- ---------- ---------- ---------- ---------- ----------
CP014429.1 GGAGATAATA TTTATAAAGA AGCTAAA--- ---------- ---------- ---------- ---------- ----------
CP014426.1 GGAGATAATA TTTATAAAGA AGCTAAA--- ---------- ---------- ---------- ---------- ----------
CP014423.1 GGAGATAATA TTTATAAAGA AGCTAAA--- ---------- ---------- ---------- ---------- ----------
CP014420.1 GGAGATAATA TTTATAAAGA AGCTAAA--- ---------- ---------- ---------- ---------- ----------
CP014415.1 GGAGATAATA TTTATAAAGA AGCTAAA--- ---------- ---------- ---------- ---------- ----------
CP014412.1 GGAGATAATA TTTATAAAGA AGCTAAA--- ---------- ---------- ---------- ---------- ----------
CP014409.1 GGAGATAATA TTTATAAAGA AGCTAAA--- ---------- ---------- ---------- ---------- ----------
CP014407.1 GGAGATAATA TTTATAAAGA AGCTAAA--- ---------- ---------- ---------- ---------- ----------
CP014402.1 GGAGATAATA TTTATAAAGA AGCTAAA--- ---------- ---------- ---------- ---------- ----------
CP014397.1 GGAGATAATA TTTATAAAGA AGCTAAA--- ---------- ---------- ---------- ---------- ----------
CP014392.1 GGAGATAATA TTTATAAAGA AGCTAAA--- ---------- ---------- ---------- ---------- ----------
CP014387.1 GGAGATAATA TTTATAAAGA AGCTAAA--- ---------- ---------- ---------- ---------- ----------
CP014384.1 GGAGATAATA TTTATAAAGA AGCTAAA--- ---------- ---------- ---------- ---------- ----------
CP014381.1 GGAGATAATA TTTATAAAGA AGCTAAA--- ---------- ---------- ---------- ---------- ----------
CP014371.1 GGAGATAATA TTTATAAAGA AGCTAAA--- ---------- ---------- ---------- ---------- ----------
CP014368.1 GGAGATAATA TTTATAAAGA AGCTAAA--- ---------- ---------- ---------- ---------- ----------
CP014365.1 GGAGATAATA TTTATAAAGA AGCTAAA--- ---------- ---------- ---------- ---------- ----------
CP014362.1 GGAGATAATA TTTATAAAGA AGCTAAA--- ---------- ---------- ---------- ---------- ----------
CP014376.1 GGAGATAATA TTTATAAAGA AGCTAAA--- ---------- ---------- ---------- ---------- ----------
CP009423.1 GGAGATAATA TTTATAAAGA AGCTAAA--- ---------- ---------- ---------- ---------- ----------
CP016855.1 GGAGATAATA TTTATAAAGA AGCTAAA--- ---------- ---------- ---------- ---------- ----------
CP013231.1 GGAGATAATA TTTATAAAGA AGCTAAA--- ---------- ---------- ---------- ---------- ----------
CP010300.1 GGAGATAATA TTTATAAAGA AGCTAAA--- ---------- ---------- ---------- ---------- ----------
CP010299.1 GGAGATAATA TTTATAAAGA AGCTAAA--- ---------- ---------- ---------- ---------- ----------
CP010298.1 GGAGATAATA TTTATAAAGA AGCTAAA--- ---------- ---------- ---------- ---------- ----------
CP010297.1 GGAGATAATA TTTATAAAGA AGCTAAA--- ---------- ---------- ---------- ---------- ----------
CP010296.1 GGAGATAATA TTTATAAAGA AGCTAAA--- ---------- ---------- ---------- ---------- ----------
CP010295.1 GGAGATAATA TTTATAAAGA AGCTAAA--- ---------- ---------- ---------- ---------- ----------
CP007690.1 GGAGATAATA TTTATAAAGA AGCTAAA--- ---------- ---------- ---------- ---------- ----------
CP007176.1 GGAGATAATA TTTATAAAGA AGCTAAA--- ---------- ---------- ---------- ---------- ----------
CP000730.1 GGAGATAATA TTTATAAAGA AGCTAAA--- ---------- ---------- ---------- ---------- ----------
CP000255.1 GGAGATAATA TTTATAAAGA AGCTAAA--- ---------- ---------- ---------- ---------- ----------
CP007539.1 GGAGATAATA TTTATAAAGA AGCTAAA--- ---------- ---------- ---------- ---------- ----------
CP007657.1 GGAGATAATA TTTATAAAGA AGCTAAA--- ---------- ---------- ---------- ---------- ----------
AP014921.1 GGAGATAATA TTTATAAAGA AGCTAAA--- ---------- ---------- ---------- ---------- ----------
AB436955.1 GGAGATAATA TTTATAAAGA AGCTAAA--- ---------- ---------- ---------- ---------- ----------
AB489885.1 GGAGATAATA TTTATAAAGA AGCTAAA--- ---------- ---------- ---------- ---------- ----------
AB489873.1 GGAGATAATA TTTATAAAGA AGCTAAA--- ---------- ---------- ---------- ---------- ----------
AB489883.1 GGAGATAATA TTTATAAAGA AGCTAAA--- ---------- ---------- ---------- ---------- ----------
AB489874.1 GGAGATAATA TTTATAAAGA AGCTAAA--- ---------- ---------- ---------- ---------- ----------
LT615218.1 GGAGATAATA TTTATAAAGA AGCTAAA--- ---------- ---------- ---------- ---------- ----------
AB436976.1 GGAGATAATA TTTATAAAGA AGCTAAA--- ---------- ---------- ---------- ---------- ----------
AB489892.1 GGAGATAATA TTTATAAAGA AGCTAAA--- ---------- ---------- ---------- ---------- ----------
AJ306908.1 GGAGATAATA TTTATAAAGA AGCTAAA--- ---------- ---------- ---------- ---------- ----------
CP015646.1 GGAGATAATA TTTATAAAGA AGCTAAA--- ---------- ---------- ---------- ---------- ----------
AB488510.1 GGAGATAATA TTTATAAAGA AGCTAAA--- ---------- ---------- ---------- ---------- ----------
EU105387.1 GGAGATAATA TTTATAAAGA AGCTAAA--- ---------- ---------- ---------- ---------- ----------
AB436975.1 GGAGATAATA TTTATAAAGA AGCTAAA--- ---------- ---------- ---------- ---------- ----------
AB488499.1 GGAGATAATA TTTATAAAGA AGCTAAA--- ---------- ---------- ---------- ---------- ----------
AJ309189.1 GGAGATAATA TTTATAAAGA AGCTAAA--- ---------- ---------- ---------- ---------- ----------
FR821779.1 GGAGATCCTA TCTATAAAGA AGCGAAA--- ---------- ---------- ---------- ---------- ----------
CP012593.1 ---------- ---------- ---------- ---------- ---------- ---------- ---------- ----------
CP012692.1 ---------- ---------- ---------- ---------- ---------- ---------- ---------- ----------
CP013955.1 ---------- ---------- ---------- ---------- ---------- ---------- ---------- ----------
CP013953.1 ---------- ---------- ---------- ---------- ---------- ---------- ---------- ----------
CP014064.1 ---------- ---------- ---------- ---------- ---------- ---------- ---------- ----------
LT009690.1 ---------- ---------- ---------- ---------- ---------- ---------- ---------- ----------
AP017320.1 ---------- ---------- ---------- ---------- ---------- ---------- ---------- ----------
CP010890.1 ---------- ---------- ---------- ---------- ---------- ---------- ---------- ----------
CP001844.2 ---------- ---------- ---------- ---------- ---------- ---------- ---------- ----------
CP001781.1 ---------- ---------- ---------- ---------- ---------- ---------- ---------- ----------
AB488501.1 ---------- ---------- ---------- ---------- ---------- ---------- ---------- ----------
AB489898.1 ---------- ---------- ---------- ---------- ---------- ---------- ---------- ----------
HE579073.1 ---------- ---------- ---------- ---------- ---------- ---------- ---------- ----------
HE579071.1 ---------- ---------- ---------- ---------- ---------- ---------- ---------- ----------
HE579069.1 ---------- ---------- ---------- ---------- ---------- ---------- ---------- ----------
HE579065.1 ---------- ---------- ---------- ---------- ---------- ---------- ---------- ----------
HE579063.1 ---------- ---------- ---------- ---------- ---------- ---------- ---------- ----------

	500	
CP007670.1	---------- ----------	347
CP003033.1	---------- ----------	347
CP018205.1	---------- ----------	347
AP017377.1	---------- ----------	347
LT598688.1	---------- ----------	347
CP007676.1	---------- ----------	347
CP007672.1	---------- ----------	347
CP007674.1	---------- ----------	347
CP011526.1	---------- ----------	347
HF937103.1	---------- ----------	347
AP009351.1	---------- ----------	347
CP000046.1	---------- ----------	347
CP000253.1	---------- ----------	347
AC025591.8	---------- ----------	347
LT671859.1	---------- ----------	347
CP007499.1	---------- ----------	347
X17679.1	---------- ----------	347
CP007657.1	---------- ----------	347
CP014444.1	---------- ----------	347
CP014441.1	---------- ----------	347
CP014438.1	---------- ----------	347
CP014435.1	---------- ----------	347
CP014432.1	---------- ----------	347
CP014429.1	---------- ----------	347
CP014426.1	---------- ----------	347
CP014423.1	---------- ----------	347
CP014420.1	---------- ----------	347
CP014415.1	---------- ----------	347
CP014412.1	---------- ----------	347
CP014409.1	---------- ----------	347
CP014407.1	---------- ----------	347
CP014402.1	---------- ----------	347
CP014397.1	---------- ----------	347
CP014392.1	---------- ----------	347
CP014387.1	---------- ----------	347
CP014384.1	---------- ----------	347
CP014381.1	---------- ----------	347
CP014371.1	---------- ----------	347
CP014368.1	---------- ----------	347
CP014365.1	---------- ----------	347
CP014362.1	---------- ----------	347
CP014376.1	---------- ----------	347
CP009423.1	---------- ----------	347
CP016855.1	---------- ----------	347
CP013231.1	---------- ----------	347
CP010300.1	---------- ----------	347
CP010299.1	---------- ----------	347
CP010298.1	---------- ----------	347
CP010297.1	---------- ----------	347
CP010296.1	---------- ----------	347
CP010295.1	---------- ----------	347
CP007690.1	---------- ----------	347
CP007176.1	---------- ----------	347
CP000730.1	---------- ----------	347
CP000255.1	---------- ----------	347
CP007539.1	---------- ----------	347
CP007657.1	---------- ----------	348
AP014921.1	---------- ----------	347
AB436955.1	---------- ----------	255
AB489885.1	---------- ----------	255
AB489873.1	---------- ----------	255
AB489883.1	---------- ----------	255
AB489874.1	---------- ----------	255
LT615218.1	---------- ----------	347
AB436976.1	---------- ----------	347
AB489892.1	---------- ----------	255
AJ306908.1	---------- ----------	347
CP015646.1	---------- ----------	347
AB488510.1	---------- ----------	255
EU105387.1	---------- ----------	287
AB436975.1	---------- ----------	347
AB488499.1	---------- ----------	255
AJ309189.1	---------- ----------	171
FR821779.1	---------- ----------	347
CP012593.1	---------- ----------	-
CP012692.1	---------- ----------	-
CP013955.1	---------- ----------	-
CP013953.1	---------- ----------	-
CP014064.1	---------- ----------	-
LT009690.1	---------- ----------	-
AP017320.1	---------- ----------	-
CP010890.1	---------- ----------	-
CP001844.2	---------- ----------	-
CP001781.1	---------- ----------	-
AB488501.1	---------- ----------	-
AB489898.1	---------- ----------	-
HE579073.1	---------- ----------	-
HE579071.1	---------- ----------	-
HE579069.1	---------- ----------	-
HE579065.1	---------- ----------	-
HE579063.1	---------- ----------	-

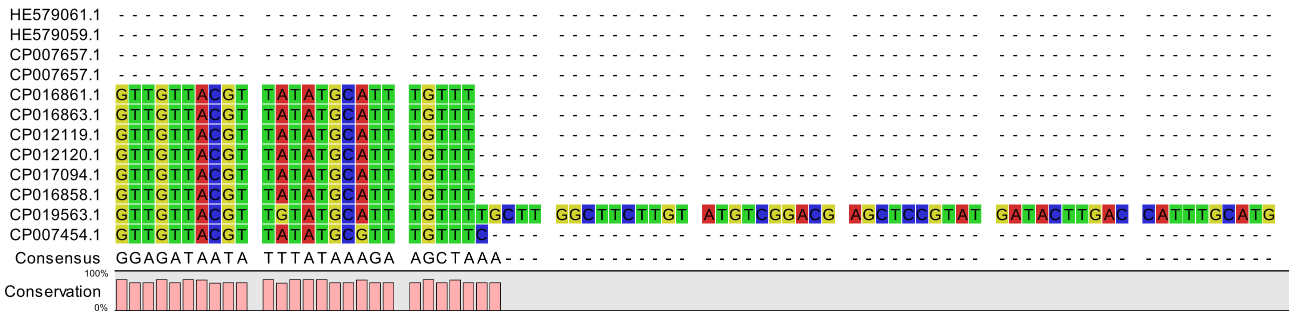
HE579061.1 ---------- ---------- ---------- ---------- ---------- ---------- ---------- ----------
HE579059.1 ---------- ---------- ---------- ---------- ---------- ---------- ---------- ----------
CP007657.1 ---------- ---------- ---------- ---------- ---------- ---------- ---------- ----------
CP007657.1 ---------- ---------- ---------- ---------- ---------- ---------- ---------- ----------
CP016861.1 GTTGTTACGT TATATGCATT TGTTT----- ---------- ---------- ---------- ---------- ----------
CP016863.1 GTTGTTACGT TATATGCATT TGTTT----- ---------- ---------- ---------- ---------- ----------
CP012119.1 GTTGTTACGT TATATGCATT TGTTT----- ---------- ---------- ---------- ---------- ----------
CP012120.1 GTTGTTACGT TATATGCATT TGTTT----- ---------- ---------- ---------- ---------- ----------
CP017094.1 GTTGTTACGT TATATGCATT TGTTT----- ---------- ---------- ---------- ---------- ----------
CP016858.1 GTTGTTACGT TATATGCATT TGTTT----- ---------- ---------- ---------- ---------- ----------
CP019563.1 GTTGTTACGT TGTATGCATT TGTTTTGCTT GGCTTCTTGT ATGTCGGACG AGCTCCGTAT GATACTTGAC CATTTGCATG
CP007454.1 GTTGTTACGT TATATGCGTT TGTTTC---- ---------- ---------- ---------- ---------- ----------
Consensus GGAGATAATA TTTATAAAGA AGCTAAA--- ---------- ---------- ---------- ---------- ----------
100%
Conservation
0%

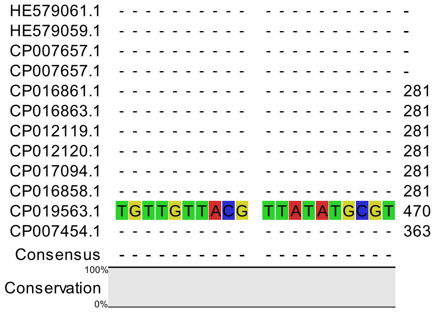
HE579061.1 -
HE579059.1 -
CP007657.1 -
CP007657.1 -
CP016861.1 - 281
CP016863.1 - 281
CP012119.1 - 281
CP012120.1 - 281
CP017094.1 - 281
CP016858.1 - 281
CP019563.1 TGTTGTTACG TTATATGCGT 470
CP007454.1 - 363
Consensus -
100%
Conservation
0%

```
                        520                  540                  560                  580
                          |                    |                    |                    |
CP007670.1  ---------- ---------- ---------- ---------- ---------- ---------- ---------- ----------
CP003033.1  ---------- ---------- ---------- ---------- ---------- ---------- ---------- ----------
CP018205.1  ---------- ---------- ---------- ---------- ---------- ---------- ---------- ----------
AP017377.1  ---------- ---------- ---------- ---------- ---------- ---------- ---------- ----------
LT598688.1  ---------- ---------- ---------- ---------- ---------- ---------- ---------- ----------
CP007676.1  ---------- ---------- ---------- ---------- ---------- ---------- ---------- ----------
CP007672.1  ---------- ---------- ---------- ---------- ---------- ---------- ---------- ----------
CP007674.1  ---------- ---------- ---------- ---------- ---------- ---------- ---------- ----------
CP011526.1  ---------- ---------- ---------- ---------- ---------- ---------- ---------- ----------
HF937103.1  ---------- ---------- ---------- ---------- ---------- ---------- ---------- ----------
AP009351.1  ---------- ---------- ---------- ---------- ---------- ---------- ---------- ----------
CP000046.1  ---------- ---------- ---------- ---------- ---------- ---------- ---------- ----------
CP000253.1  ---------- ---------- ---------- ---------- ---------- ---------- ---------- ----------
AC025591.8  ---------- ---------- ---------- ---------- ---------- ---------- ---------- ----------
LT671859.1  ---------- ---------- ---------- ---------- ---------- ---------- ---------- ----------
CP007499.1  ---------- ---------- ---------- ---------- ---------- ---------- ---------- ----------
X17679.1    ---------- ---------- ---------- ---------- ---------- ---------- ---------- ----------
CP007657.1  ---------- ---------- ---------- ---------- ---------- ---------- ---------- ----------
CP014444.1  ---------- ---------- ---------- ---------- ---------- ---------- ---------- ----------
CP014441.1  ---------- ---------- ---------- ---------- ---------- ---------- ---------- ----------
CP014438.1  ---------- ---------- ---------- ---------- ---------- ---------- ---------- ----------
CP014435.1  ---------- ---------- ---------- ---------- ---------- ---------- ---------- ----------
CP014432.1  ---------- ---------- ---------- ---------- ---------- ---------- ---------- ----------
CP014429.1  ---------- ---------- ---------- ---------- ---------- ---------- ---------- ----------
CP014426.1  ---------- ---------- ---------- ---------- ---------- ---------- ---------- ----------
CP014423.1  ---------- ---------- ---------- ---------- ---------- ---------- ---------- ----------
CP014420.1  ---------- ---------- ---------- ---------- ---------- ---------- ---------- ----------
CP014415.1  ---------- ---------- ---------- ---------- ---------- ---------- ---------- ----------
CP014412.1  ---------- ---------- ---------- ---------- ---------- ---------- ---------- ----------
CP014409.1  ---------- ---------- ---------- ---------- ---------- ---------- ---------- ----------
CP014407.1  ---------- ---------- ---------- ---------- ---------- ---------- ---------- ----------
CP014402.1  ---------- ---------- ---------- ---------- ---------- ---------- ---------- ----------
CP014397.1  ---------- ---------- ---------- ---------- ---------- ---------- ---------- ----------
CP014392.1  ---------- ---------- ---------- ---------- ---------- ---------- ---------- ----------
CP014387.1  ---------- ---------- ---------- ---------- ---------- ---------- ---------- ----------
CP014384.1  ---------- ---------- ---------- ---------- ---------- ---------- ---------- ----------
CP014381.1  ---------- ---------- ---------- ---------- ---------- ---------- ---------- ----------
CP014371.1  ---------- ---------- ---------- ---------- ---------- ---------- ---------- ----------
CP014368.1  ---------- ---------- ---------- ---------- ---------- ---------- ---------- ----------
CP014365.1  ---------- ---------- ---------- ---------- ---------- ---------- ---------- ----------
CP014362.1  ---------- ---------- ---------- ---------- ---------- ---------- ---------- ----------
CP014376.1  ---------- ---------- ---------- ---------- ---------- ---------- ---------- ----------
CP009423.1  ---------- ---------- ---------- ---------- ---------- ---------- ---------- ----------
CP016855.1  ---------- ---------- ---------- ---------- ---------- ---------- ---------- ----------
CP013231.1  ---------- ---------- ---------- ---------- ---------- ---------- ---------- ----------
CP010300.1  ---------- ---------- ---------- ---------- ---------- ---------- ---------- ----------
CP010299.1  ---------- ---------- ---------- ---------- ---------- ---------- ---------- ----------
CP010298.1  ---------- ---------- ---------- ---------- ---------- ---------- ---------- ----------
CP010297.1  ---------- ---------- ---------- ---------- ---------- ---------- ---------- ----------
CP010296.1  ---------- ---------- ---------- ---------- ---------- ---------- ---------- ----------
CP010295.1  ---------- ---------- ---------- ---------- ---------- ---------- ---------- ----------
CP007690.1  ---------- ---------- ---------- ---------- ---------- ---------- ---------- ----------
CP007176.1  ---------- ---------- ---------- ---------- ---------- ---------- ---------- ----------
CP000730.1  ---------- ---------- ---------- ---------- ---------- ---------- ---------- ----------
CP000255.1  ---------- ---------- ---------- ---------- ---------- ---------- ---------- ----------
CP007539.1  ---------- ---------- ---------- ---------- ---------- ---------- ---------- ----------
CP007657.1  ---------- ---------- ---------- ---------- ---------- ---------- ---------- ----------
AP014921.1  ---------- ---------- ---------- ---------- ---------- ---------- ---------- ----------
AB436955.1  ---------- ---------- ---------- ---------- ---------- ---------- ---------- ----------
AB489885.1  ---------- ---------- ---------- ---------- ---------- ---------- ---------- ----------
AB489873.1  ---------- ---------- ---------- ---------- ---------- ---------- ---------- ----------
AB489883.1  ---------- ---------- ---------- ---------- ---------- ---------- ---------- ----------
AB489874.1  ---------- ---------- ---------- ---------- ---------- ---------- ---------- ----------
LT615218.1  ---------- ---------- ---------- ---------- ---------- ---------- ---------- ----------
AB436976.1  ---------- ---------- ---------- ---------- ---------- ---------- ---------- ----------
AB489892.1  ---------- ---------- ---------- ---------- ---------- ---------- ---------- ----------
AJ306908.1  ---------- ---------- ---------- ---------- ---------- ---------- ---------- ----------
CP015646.1  ---------- ---------- ---------- ---------- ---------- ---------- ---------- ----------
AB488510.1  ---------- ---------- ---------- ---------- ---------- ---------- ---------- ----------
EU105387.1  ---------- ---------- ---------- ---------- ---------- ---------- ---------- ----------
AB436975.1  ---------- ---------- ---------- ---------- ---------- ---------- ---------- ----------
AB488499.1  ---------- ---------- ---------- ---------- ---------- ---------- ---------- ----------
AJ309189.1  ---------- ---------- ---------- ---------- ---------- ---------- ---------- ----------
FR821779.1  ---------- ---------- ---------- ---------- ---------- ---------- ---------- ----------
CP012593.1  ---------- ---------- ---------- ---------- ---------- ---------- ---------- ----------
CP012692.1  ---------- ---------- ---------- ---------- ---------- ---------- ---------- ----------
CP013955.1  ---------- ---------- ---------- ---------- ---------- ---------- ---------- ----------
CP013953.1  ---------- ---------- ---------- ---------- ---------- ---------- ---------- ----------
CP014064.1  ---------- ---------- ---------- ---------- ---------- ---------- ---------- ----------
LT009690.1  ---------- ---------- ---------- ---------- ---------- ---------- ---------- ----------
AP017320.1  ---------- ---------- ---------- ---------- ---------- ---------- ---------- ----------
CP010890.1  ---------- ---------- ---------- ---------- ---------- ---------- ---------- ----------
CP001844.2  ---------- ---------- ---------- ---------- ---------- ---------- ---------- ----------
CP001781.1  ---------- ---------- ---------- ---------- ---------- ---------- ---------- ----------
AB488501.1  ---------- ---------- ---------- ---------- ---------- ---------- ---------- ----------
AB489898.1  ---------- ---------- ---------- ---------- ---------- ---------- ---------- ----------
HE579073.1  ---------- ---------- ---------- ---------- ---------- ---------- ---------- ----------
HE579071.1  ---------- ---------- ---------- ---------- ---------- ---------- ---------- ----------
HE579069.1  ---------- ---------- ---------- ---------- ---------- ---------- ---------- ----------
HE579065.1  ---------- ---------- ---------- ---------- ---------- ---------- ---------- ----------
HE579063.1  ---------- ---------- ---------- ---------- ---------- ---------- ---------- ----------
```

		600	
CP007670.1	---------G	ATAGGTTGTT	358
CP003033.1	---------G	ATAGGTTGTT	358
CP018205.1	---------G	ATAGGTTGTT	358
AP017377.1	---------G	ATAGGTTGTT	358
LT598688.1	---------G	ATAGGTTGTT	358
CP007676.1	---------G	ATAGGTTGTT	358
CP007672.1	---------G	ATAGGTTGTT	358
CP007674.1	---------G	ATAGGTTGTT	358
CP011526.1	---------G	ATAGGTTGTT	358
HF937103.1	---------G	ATAGGTTGTT	358
AP009351.1	---------G	ATAGGTTGTT	358
CP000046.1	---------G	ATAGGTTGTT	358
CP000253.1	---------G	ATAGGTTGTT	358
AC025591.8	---------G	ATAGGTTGTT	358
LT671859.1	---------G	ATAGGTTGTT	358
CP007499.1	---------G	ATAGGTTGTT	358
X17679.1	---------G	ATAGGTTGTT	358
CP007657.1	---------G	ATAGGTTGTT	358
CP014444.1	---------G	ATAGGTTGTT	358
CP014441.1	---------G	ATAGGTTGTT	358
CP014438.1	---------G	ATAGGTTGTT	358
CP014435.1	---------G	ATAGGTTGTT	358
CP014432.1	---------G	ATAGGTTGTT	358
CP014429.1	---------G	ATAGGTTGTT	358
CP014426.1	---------G	ATAGGTTGTT	358
CP014423.1	---------G	ATAGGTTGTT	358
CP014420.1	---------G	ATAGGTTGTT	358
CP014415.1	---------G	ATAGGTTGTT	358
CP014412.1	---------G	ATAGGTTGTT	358
CP014409.1	---------G	ATAGGTTGTT	358
CP014407.1	---------G	ATAGGTTGTT	358
CP014402.1	---------G	ATAGGTTGTT	358
CP014397.1	---------G	ATAGGTTGTT	358
CP014392.1	---------G	ATAGGTTGTT	358
CP014387.1	---------G	ATAGGTTGTT	358
CP014384.1	---------G	ATAGGTTGTT	358
CP014381.1	---------G	ATAGGTTGTT	358
CP014371.1	---------G	ATAGGTTGTT	358
CP014368.1	---------G	ATAGGTTGTT	358
CP014365.1	---------G	ATAGGTTGTT	358
CP014362.1	---------G	ATAGGTTGTT	358
CP014376.1	---------G	ATAGGTTGTT	358
CP009423.1	---------G	ATAGGTTGTT	358
CP016855.1	---------G	ATAGGTTGTT	358
CP013231.1	---------G	ATAGGTTGTT	358
CP010300.1	---------G	ATAGGTTGTT	358
CP010299.1	---------G	ATAGGTTGTT	358
CP010298.1	---------G	ATAGGTTGTT	358
CP010297.1	---------G	ATAGGTTGTT	358
CP010296.1	---------G	ATAGGTTGTT	358
CP010295.1	---------G	ATAGGTTGTT	358
CP007690.1	---------G	ATAGGTTGTT	358
CP007176.1	---------G	ATAGGTTGTT	358
CP000730.1	---------G	ATAGGTTGTT	358
CP000255.1	---------G	ATAGGTTGTT	358
CP007539.1	---------G	ATAGGTTGTT	358
CP007657.1	---------G	ATAGGTTGTT	359
AP014921.1	---------G	ATAGGTTGTT	358
AB436955.1	---------G	ATAGGTTGTT	266
AB489885.1	---------G	ATAGGTTGTT	266
AB489873.1	---------G	ATAGGTTGTT	266
AB489883.1	---------G	ATAGGTTGTT	266
AB489874.1	---------G	ATAGGTTGTT	266
LT615218.1	---------G	ATAGGTTGTT	358
AB436976.1	---------G	ATAGGTTGTT	358
AB489892.1	---------G	ATAGGTTGTT	266
AJ306908.1	---------G	ATAGGTTGTT	358
CP015646.1	---------G	ATAGGTTGTT	358
AB488510.1	---------G	ATAGGTTGTT	266
EU105387.1	---------G	ATAGGTTGTT	298
AB436975.1	---------G	ATAGGTTGTT	358
AB488499.1	---------G	ATAGGTTGTT	266
AJ309189.1	---------G	ATAGGTTGTT	182
FR821779.1	---------G	ATAGATTGAT	358
CP012593.1	----------	----------	-
CP012692.1	----------	----------	-
CP013955.1	----------	----------	-
CP013953.1	----------	----------	-
CP014064.1	----------	----------	-
LT009690.1	----------	----------	-
AP017320.1	----------	----------	-
CP010890.1	----------	----------	-
CP001844.2	----------	----------	-
CP001781.1	----------	----------	-
AB488501.1	----------	----------	-
AB489898.1	----------	----------	-
HE579073.1	----------	----------	-
HE579071.1	----------	----------	-
HE579069.1	----------	----------	-
HE579065.1	----------	----------	-
HE579063.1	----------	----------	-

```
HE579061.1   ----------  ----------  ----------  ----------  ----------  ----------  ----------  ----------
HE579059.1   ----------  ----------  ----------  ----------  ----------  ----------  ----------  ----------
CP007657.1   ----------  ----------  ----------  ----------  ----------  ----------  ----------  ----------
CP007657.1   ----------  ----------  ----------  ----------  ----------  ----------  ----------  ----------
CP016861.1   ----------  ----------  ----------  ----------  ----------  ----------  ----------  ----------
CP016863.1   ----------  ----------  ----------  ----------  ----------  ----------  ----------  ----------
CP012119.1   ----------  ----------  ----------  ----------  ----------  ----------  ----------  ----------
CP012120.1   ----------  ----------  ----------  ----------  ----------  ----------  ----------  ----------
CP017094.1   ----------  ----------  ----------  ----------  ----------  ----------  ----------  ----------
CP016858.1   ----------  ----------  ----------  ----------  ----------  ----------  ----------  ----------
CP019563.1   TCGTTTTACT  TGGCTTGTTT  TGTGTTGGAC  GAGCGCCATA  TGTTACTGTG  CCATCTTGAT  TTGTCGTTAC  GTTGTATGCG
CP007454.1   -------ACT  TGGCTTGTTT  TGTGTTGGGC  GAGCTCCGTA  TGATACTGTG  CCATCTTGAT  TTGTCGTTAC  GTTGTATGCA
Consensus    ----------  ----------  ----------  ----------  ----------  ----------  ----------  ----------
100%
Conservation
0%
```

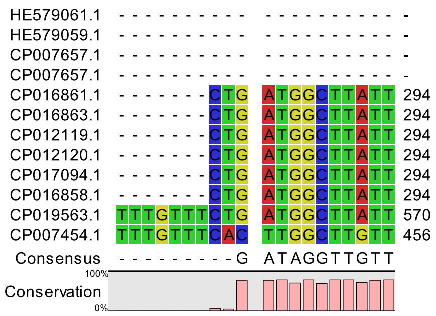
HE579061.1 ---------- ----------- -
HE579059.1 ---------- ----------- -
CP007657.1 ---------- ----------- -
CP007657.1 ---------- ----------- -
CP016861.1 -------CTG ATGGCTTATT 294
CP016863.1 -------CTG ATGGCTTATT 294
CP012119.1 -------CTG ATGGCTTATT 294
CP012120.1 -------CTG ATGGCTTATT 294
CP017094.1 -------CTG ATGGCTTATT 294
CP016858.1 -------CTG ATGGCTTATT 294
CP019563.1 TTTGTTTCTG ATGGCTTATT 570
CP007454.1 TTTGTTTCAC TTGGCTTGTT 456
Consensus ---------G ATAGGTTGTT
100%
Conservation
0%

		620		640		660		680
CP007670.1	GGAAAAGGTA	TTAAGGGAAG	ATCAATATCT	TTTGGAGAGA	AAGAAATCTC	AATATGAAGA	TTATAAACAA	TGGTATGCAA
CP003033.1	GGAAAAGGTA	TTAAGGGAAG	ATCAATATCT	TTTGGAGAGA	AAGAAATCTC	AATATGAAGA	TTATAAACAA	TGGTATGCAA
CP018205.1	GGAAAAGGTA	TTAAGGGAAG	ATCAATATCT	TTTGGAGAGA	AAGAAATCTC	AATATGAAGA	TTATAAACAA	TGGTATGCAA
AP017377.1	GGAAAAGGTA	TTAAGGGAAG	ATCAATATCT	TTTGGAGAGA	AAGAAATCTC	AATATGAAGA	TTATAAACAA	TGGTATGCAA
LT598688.1	GGAAAAGGTA	TTAAGGGAAG	ATCAATATCT	TTTGGAGAGA	AAGAAATCTC	AATATGAAGA	TTATAAACAA	TGGTATGCAA
CP007676.1	GGAAAAGGTA	TTAAGGGAAG	ATCAATATCT	TTTGGAGAGA	AAGAAATCTC	AATATGAAGA	TTATAAACAA	TGGTATGCAA
CP007672.1	GGAAAAGGTA	TTAAGGGAAG	ATCAATATCT	TTTGGAGAGA	AAGAAATCTC	AATATGAAGA	TTATAAACAA	TGGTATGCAA
CP007674.1	GGAAAAGGTA	TTAAGGGAAG	ATCAATATCT	TTTGGAGAGA	AAGAAATCTC	AATATGAAGA	TTATAAACAA	TGGTATGCAA
CP011526.1	GGAAAAGGTA	TTAAGGGAAG	ATCAATATCT	TTTGGAGAGA	AAGAAATCTC	AATATGAAGA	TTATAAACAA	TGGTATGCAA
HF937103.1	GGAAAAGGTA	TTAAGGGAAG	ATCAATATCT	TTTGGAGAGA	AAGAAATCTC	AATATGAAGA	TTATAAACAA	TGGTATGCAA
AP009351.1	GGAAAAGGTA	TTAAGGGAAG	ATCAATATCT	TTTGGAGAGA	AAGAAATCTC	AATATGAAGA	TTATAAACAA	TGGTATGCAA
CP000046.1	GGAAAAGGTA	TTAAGGGAAG	ATCAATATCT	TTTGGAGAGA	AAGAAATCTC	AATATGAAGA	TTATAAACAA	TGGTATGCAA
CP000253.1	GGAAAAGGTA	TTAAGGGAAG	ATCAATATCT	TTTGGAGAGA	AAGAAATCTC	AATATGAAGA	TTATAAACAA	TGGTATGCAA
AC025591.8	GGAAAAGGTA	TTAAGGGAAG	ATCAATATCT	TTTGGAGAGA	AAGAAATCTC	AATATGAAGA	TTATAAACAA	TGGTATGCAA
LT671859.1	GGAAAAGGTA	TTAAGGGAAG	ATCAATATCT	TTTGGAGAGA	AAGAAATCTC	AATATGAAGA	TTATAAACAA	TGGTATGCAA
CP007499.1	GGAAAAGGTA	TTAAGGGAAG	ATCAATATCT	TTTGGAGAGA	AAGAAATCTC	AATATGAAGA	TTATAAACAA	TGGTATGCAA
X17679.1	GGAAAAGGTA	TTAAGGGAAG	ATCAATATCT	TTTGGAGAGA	AAGAAATCTC	AATATGAAGA	TTATAAACAA	TGGTATGCAA
CP007657.1	GGAAAAGGTA	TTAAGGGAAG	ATCAATATCT	TTTGGAGAGA	AAGAAATCTC	AATATGAAGA	TTATAAACAA	TGGTATGCAA
CP014444.1	GGAAAAGGTA	TTAAGGGAAG	ATCAATATCT	TTTGGAGAGA	AAGAAATCTC	AATATGAAGA	TTATAAACAA	TGGTATGCAA
CP014441.1	GGAAAAGGTA	TTAAGGGAAG	ATCAATATCT	TTTGGAGAGA	AAGAAATCTC	AATATGAAGA	TTATAAACAA	TGGTATGCAA
CP014438.1	GGAAAAGGTA	TTAAGGGAAG	ATCAATATCT	TTTGGAGAGA	AAGAAATCTC	AATATGAAGA	TTATAAACAA	TGGTATGCAA
CP014435.1	GGAAAAGGTA	TTAAGGGAAG	ATCAATATCT	TTTGGAGAGA	AAGAAATCTC	AATATGAAGA	TTATAAACAA	TGGTATGCAA
CP014432.1	GGAAAAGGTA	TTAAGGGAAG	ATCAATATCT	TTTGGAGAGA	AAGAAATCTC	AATATGAAGA	TTATAAACAA	TGGTATGCAA
CP014429.1	GGAAAAGGTA	TTAAGGGAAG	ATCAATATCT	TTTGGAGAGA	AAGAAATCTC	AATATGAAGA	TTATAAACAA	TGGTATGCAA
CP014426.1	GGAAAAGGTA	TTAAGGGAAG	ATCAATATCT	TTTGGAGAGA	AAGAAATCTC	AATATGAAGA	TTATAAACAA	TGGTATGCAA
CP014423.1	GGAAAAGGTA	TTAAGGGAAG	ATCAATATCT	TTTGGAGAGA	AAGAAATCTC	AATATGAAGA	TTATAAACAA	TGGTATGCAA
CP014420.1	GGAAAAGGTA	TTAAGGGAAG	ATCAATATCT	TTTGGAGAGA	AAGAAATCTC	AATATGAAGA	TTATAAACAA	TGGTATGCAA
CP014415.1	GGAAAAGGTA	TTAAGGGAAG	ATCAATATCT	TTTGGAGAGA	AAGAAATCTC	AATATGAAGA	TTATAAACAA	TGGTATGCAA
CP014412.1	GGAAAAGGTA	TTAAGGGAAG	ATCAATATCT	TTTGGAGAGA	AAGAAATCTC	AATATGAAGA	TTATAAACAA	TGGTATGCAA
CP014409.1	GGAAAAGGTA	TTAAGGGAAG	ATCAATATCT	TTTGGAGAGA	AAGAAATCTC	AATATGAAGA	TTATAAACAA	TGGTATGCAA
CP014407.1	GGAAAAGGTA	TTAAGGGAAG	ATCAATATCT	TTTGGAGAGA	AAGAAATCTC	AATATGAAGA	TTATAAACAA	TGGTATGCAA
CP014402.1	GGAAAAGGTA	TTAAGGGAAG	ATCAATATCT	TTTGGAGAGA	AAGAAATCTC	AATATGAAGA	TTATAAACAA	TGGTATGCAA
CP014397.1	GGAAAAGGTA	TTAAGGGAAG	ATCAATATCT	TTTGGAGAGA	AAGAAATCTC	AATATGAAGA	TTATAAACAA	TGGTATGCAA
CP014392.1	GGAAAAGGTA	TTAAGGGAAG	ATCAATATCT	TTTGGAGAGA	AAGAAATCTC	AATATGAAGA	TTATAAACAA	TGGTATGCAA
CP014387.1	GGAAAAGGTA	TTAAGGGAAG	ATCAATATCT	TTTGGAGAGA	AAGAAATCTC	AATATGAAGA	TTATAAACAA	TGGTATGCAA
CP014384.1	GGAAAAGGTA	TTAAGGGAAG	ATCAATATCT	TTTGGAGAGA	AAGAAATCTC	AATATGAAGA	TTATAAACAA	TGGTATGCAA
CP014381.1	GGAAAAGGTA	TTAAGGGAAG	ATCAATATCT	TTTGGAGAGA	AAGAAATCTC	AATATGAAGA	TTATAAACAA	TGGTATGCAA
CP014371.1	GGAAAAGGTA	TTAAGGGAAG	ATCAATATCT	TTTGGAGAGA	AAGAAATCTC	AATATGAAGA	TTATAAACAA	TGGTATGCAA
CP014368.1	GGAAAAGGTA	TTAAGGGAAG	ATCAATATCT	TTTGGAGAGA	AAGAAATCTC	AATATGAAGA	TTATAAACAA	TGGTATGCAA
CP014365.1	GGAAAAGGTA	TTAAGGGAAG	ATCAATATCT	TTTGGAGAGA	AAGAAATCTC	AATATGAAGA	TTATAAACAA	TGGTATGCAA
CP014362.1	GGAAAAGGTA	TTAAGGGAAG	ATCAATATCT	TTTGGAGAGA	AAGAAATCTC	AATATGAAGA	TTATAAACAA	TGGTATGCAA
CP014376.1	GGAAAAGGTA	TTAAGGGAAG	ATCAATATCT	TTTGGAGAGA	AAGAAATCTC	AATATGAAGA	TTATAAACAA	TGGTATGCAA
CP009423.1	GGAAAAGGTA	TTAAGGGAAG	ATCAATATCT	TTTGGAGAGA	AAGAAATCTC	AATATGAAGA	TTATAAACAA	TGGTATGCAA
CP016855.1	GGAAAAGGTA	TTAAGGGAAG	ATCAATATCT	TTTGGAGAGA	AAGAAATCTC	AATATGAAGA	TTATAAACAA	TGGTATGCAA
CP013231.1	GGAAAAGGTA	TTAAGGGAAG	ATCAATATCT	TTTGGAGAGA	AAGAAATCTC	AATATGAAGA	TTATAAACAA	TGGTATGCAA
CP010300.1	GGAAAAGGTA	TTAAGGGAAG	ATCAATATCT	TTTGGAGAGA	AAGAAATCTC	AATATGAAGA	TTATAAACAA	TGGTATGCAA
CP010299.1	GGAAAAGGTA	TTAAGGGAAG	ATCAATATCT	TTTGGAGAGA	AAGAAATCTC	AATATGAAGA	TTATAAACAA	TGGTATGCAA
CP010298.1	GGAAAAGGTA	TTAAGGGAAG	ATCAATATCT	TTTGGAGAGA	AAGAAATCTC	AATATGAAGA	TTATAAACAA	TGGTATGCAA
CP010297.1	GGAAAAGGTA	TTAAGGGAAG	ATCAATATCT	TTTGGAGAGA	AAGAAATCTC	AATATGAAGA	TTATAAACAA	TGGTATGCAA
CP010296.1	GGAAAAGGTA	TTAAGGGAAG	ATCAATATCT	TTTGGAGAGA	AAGAAATCTC	AATATGAAGA	TTATAAACAA	TGGTATGCAA
CP010295.1	GGAAAAGGTA	TTAAGGGAAG	ATCAATATCT	TTTGGAGAGA	AAGAAATCTC	AATATGAAGA	TTATAAACAA	TGGTATGCAA
CP007690.1	GGAAAAGGTA	TTAAGGGAAG	ATCAATATCT	TTTGGAGAGA	AAGAAATCTC	AATATGAAGA	TTATAAACAA	TGGTATGCAA
CP007176.1	GGAAAAGGTA	TTAAGGGAAG	ATCAATATCT	TTTGGAGAGA	AAGAAATCTC	AATATGAAGA	TTATAAACAA	TGGTATGCAA
CP000730.1	GGAAAAGGTA	TTAAGGGAAG	ATCAATATCT	TTTGGAGAGA	AAGAAATCTC	AATATGAAGA	TTATAAACAA	TGGTATGCAA
CP000255.1	GGAAAAGGTA	TTAAGGGAAG	ATCAATATCT	TTTGGAGAGA	AAGAAATCTC	AATATGAAGA	TTATAAACAA	TGGTATGCAA
CP007539.1	GGAAAAGGTA	TTAAGGGAAG	ATCAATATCT	TTTGGAGAGA	AAGAAATCTC	AATATGAAGA	TTATAAACAA	TGGTATGCAA
CP007657.1	GGAAAAGGTA	TTAAGGGAAG	ATCAATATCT	TTTGGAGAGA	AAGAAATCTC	AATATGAAGA	TTATAAACAA	TGGTATGCAA
AP014921.1	GGAAAAGGTA	TTAAGGGAAG	ATCAATATCT	TTTGGAGAGA	AAGAAATCTC	AATATGAAGA	TTATAAACAA	TGGTATGCAA
AB436955.1	GGAAAAGGTA	TTAAGGGAAG	ATCAATATCT	TTTGGAGAGA	AAGAAATCTC	AATATGAAGA	TTATAAACAA	TGGTATGCAA
AB489885.1	GGAAAAGGTA	TTAAGGGAAG	ATCAATATCT	TTTGGAGAGA	AAGAAATCTC	AATATGAAGA	TTATAAACAA	TGGTATGCAA
AB489873.1	GGAAAAGGTA	TTAAGGGAAG	ATCAATATCT	TTTGGAGAGA	AAGAAATCTC	AATATGAAGA	TTATAAACAA	TGGTATGCAA
AB489883.1	GGAAAAGGTA	TTAAGGGAAG	ATCAATATCT	TTTGGAGAGA	AAGAAATCTC	AATATGAAGA	TTATAAACAA	TGGTATGCAA
AB489874.1	GGAAAAGGTA	TTAAGGGAAG	ATCAATATCT	TTTGGAGAGA	AAGAAATCTC	AATATGAAGA	TTATAAACAA	TGGTATGCAA
LT615218.1	GGAAAAGGTA	TTAAGGGAAG	ATCAATATCT	TTTGGAGAGA	AAGAAATCTC	AATATGAAGA	TTATAAACAA	TGGTATGCAA
AB436976.1	GGAAAAGGTA	TTAAGGGAAG	ATCAATATCT	TTTGGAGAGA	AAGAAATCTC	AATATGAAGA	TTATAAACAA	TGGTATGCAA
AB489892.1	GGAAAAGGTA	TTAAGGGAAG	ATCAATATCT	TTTGGAGAGA	AAGAAATCTC	AATATGAAGA	TTATAAACAA	TGGTATGCAA
AJ306908.1	GGAAAAGGTA	TTAAGGGAAG	ATCAATATCT	TTTGGAGAGA	AAGAAATCTC	AATATGAAGA	TTATAAACAA	TGGTATGCAA
CP015646.1	GGAAAAGGTA	TTAAGGGAAG	ATCAATATCT	TTTGGAGAGA	AAGAAATCTC	AATATGAAGA	TTATAAACAA	TGGTATGCAA
AB488510.1	GGAAAAGGTA	TTAAGGGAAG	ATCAATATCT	TTTGGAGAGA	AAGAAATCTC	AATATGAAGA	TTATAAACAA	TGGTATGCAA
EU105387.1	GGAAAAGGTA	TTAAGGGAAG	ATCAATATCT	TTTGGAGAGA	AAGAAATCTC	AATATGAAGA	TTATAAACAA	TGGTATGCAA
AB436975.1	GGAAAAGGTA	TTAAGGGAAG	ATCAATATCT	TTTGGAGAGA	AAGAAATCTC	AATATGAAGA	TTATAAACAA	TGGTATGCAA
AB488499.1	GGAAAAGGTA	TTAAGGGAAG	ATCAATATCT	TTTGGAGAGA	AAGAAATCTC	AATATGAAGA	TTATAAACAA	TGGTATGCAA
AJ309189.1	GGAAAAGGTA	TTAAGGGAAG	ATCAATATCT	TTTGGAGAGA	AAGAAATCTC	AATATGAAGA	TTATAAACAA	TGGTATGCAA
FR821779.1	GACAAGAGTA	TTAGGAGAAG	ACCAGTATTT	ATTAAAGAAA	AAGATTGATG	AATATGAGCT	TTATAAAAAG	TGGTATAAAA
CP012593.1	----------	----------	----------	----------	----------	----------	----------	----------
CP012692.1	----------	----------	----------	----------	----------	----------	----------	----------
CP013955.1	----------	----------	----------	----------	----------	----------	----------	----------
CP013953.1	----------	----------	----------	----------	----------	----------	----------	----------
CP014064.1	----------	----------	----------	----------	----------	----------	----------	----------
LT009690.1	----------	----------	----------	----------	----------	----------	----------	----------
AP017320.1	----------	----------	----------	----------	----------	----------	----------	----------
CP010890.1	----------	----------	----------	----------	----------	----------	----------	----------
CP001844.2	----------	----------	----------	----------	----------	----------	----------	----------
CP001781.1	----------	----------	----------	----------	----------	----------	----------	----------
AB488501.1	----------	----------	----------	----------	----------	----------	----------	----------
AB489898.1	----------	----------	----------	----------	----------	----------	----------	----------
HE579073.1	----------	----------	----------	----------	----------	----------	----------	----------
HE579071.1	----------	----------	----------	----------	----------	----------	----------	----------
HE579069.1	----------	----------	----------	----------	----------	----------	----------	----------
HE579065.1	----------	----------	----------	----------	----------	----------	----------	----------
HE579063.1	----------	----------	----------	----------	----------	----------	----------	----------

		700	
CP007670.1	ATTATAAAAA	AGAAAATCCT	458
CP003033.1	ATTATAAAAA	AGAAAATCCT	458
CP018205.1	ATTATAAAAA	AGAAAATCCT	458
AP017377.1	ATTATAAAAA	AGAAAATCCT	458
LT598688.1	ATTATAAAAA	AGAAAATCCT	458
CP007676.1	ATTATAAAAA	AGAAAATCCT	458
CP007672.1	ATTATAAAAA	AGAAAATCCT	458
CP007674.1	ATTATAAAAA	AGAAAATCCT	458
CP011526.1	ATTATAAAAA	AGAAAATCCT	458
HF937103.1	ATTATAAAAA	AGAAAATCCT	458
AP009351.1	ATTATAAAAA	AGAAAATCCT	458
CP000046.1	ATTATAAAAA	AGAAAATCCT	458
CP000253.1	ATTATAAAAA	AGAAAATCCT	458
AC025591.8	ATTATAAAAA	AGAAAATCCT	458
LT671859.1	ATTATAAAAA	AGAAAATCCT	458
CP007499.1	ATTATAAAAA	AGAAAATCCT	458
X17679.1	ATTATAAAAA	AGAAAATCCT	458
CP007657.1	ATTATAAAAA	AGAAAATCCT	458
CP014444.1	ATTATAAAAA	AGAAAATCCT	458
CP014441.1	ATTATAAAAA	AGAAAATCCT	458
CP014438.1	ATTATAAAAA	AGAAAATCCT	458
CP014435.1	ATTATAAAAA	AGAAAATCCT	458
CP014432.1	ATTATAAAAA	AGAAAATCCT	458
CP014429.1	ATTATAAAAA	AGAAAATCCT	458
CP014426.1	ATTATAAAAA	AGAAAATCCT	458
CP014423.1	ATTATAAAAA	AGAAAATCCT	458
CP014420.1	ATTATAAAAA	AGAAAATCCT	458
CP014415.1	ATTATAAAAA	AGAAAATCCT	458
CP014412.1	ATTATAAAAA	AGAAAATCCT	458
CP014409.1	ATTATAAAAA	AGAAAATCCT	458
CP014407.1	ATTATAAAAA	AGAAAATCCT	458
CP014402.1	ATTATAAAAA	AGAAAATCCT	458
CP014397.1	ATTATAAAAA	AGAAAATCCT	458
CP014392.1	ATTATAAAAA	AGAAAATCCT	458
CP014387.1	ATTATAAAAA	AGAAAATCCT	458
CP014384.1	ATTATAAAAA	AGAAAATCCT	458
CP014381.1	ATTATAAAAA	AGAAAATCCT	458
CP014371.1	ATTATAAAAA	AGAAAATCCT	458
CP014368.1	ATTATAAAAA	AGAAAATCCT	458
CP014365.1	ATTATAAAAA	AGAAAATCCT	458
CP014362.1	ATTATAAAAA	AGAAAATCCT	458
CP014376.1	ATTATAAAAA	AGAAAATCCT	458
CP009423.1	ATTATAAAAA	AGAAAATCCT	458
CP016855.1	ATTATAAAAA	AGAAAATCCT	458
CP013231.1	ATTATAAAAA	AGAAAATCCT	458
CP010300.1	ATTATAAAAA	AGAAAATCCT	458
CP010299.1	ATTATAAAAA	AGAAAATCCT	458
CP010298.1	ATTATAAAAA	AGAAAATCCT	458
CP010297.1	ATTATAAAAA	AGAAAATCCT	458
CP010296.1	ATTATAAAAA	AGAAAATCCT	458
CP010295.1	ATTATAAAAA	AGAAAATCCT	458
CP007690.1	ATTATAAAAA	AGAAAATCCT	458
CP007176.1	ATTATAAAAA	AGAAAATCCT	458
CP000730.1	ATTATAAAAA	AGAAAATCCT	458
CP000255.1	ATTATAAAAA	AGAAAATCCT	458
CP007539.1	ATTATAAAAA	AGAAAATCCT	458
CP007657.1	ATTATAAAAA	AGAAAATCCT	459
AP014921.1	ATTATAAAAA	AGAAAATCCT	458
AB436955.1	ATTATAAAAA	AGAAAATCCT	366
AB489885.1	ATTATAAAAA	AGAAAATCCT	366
AB489873.1	ATTATAAAAA	AGAAAATCCT	366
AB489883.1	ATTATAAAAA	AGAAAATCCT	366
AB489874.1	ATTATAAAAA	AGAAAATCCT	366
LT615218.1	ATTATAAAAA	AGAAAATCCT	458
AB436976.1	ATTATAAAAA	AGAAAATCCT	458
AB489892.1	ATTATAAAAA	AGAAAATCCT	366
AJ306908.1	ATTATAAAAA	AGAAAATCCT	458
CP015646.1	ATTATAAAAA	AGAAAATCCT	458
AB488510.1	ATTATAAAAA	AGAAAATCCT	366
EU105387.1	ATTATAAAAA	AGAAAATCCT	398
AB436975.1	ATTATAAAAA	AGAAAATCCT	458
AB488499.1	ATTATAAAAA	AGAAAATCCT	366
AJ309189.1	ATTATAAAAA	AGAAAATCCT	282
FR821779.1	GTT-CAAATA	AGAACACT--	455
CP012593.1	----------	----------	-
CP012692.1	----------	----------	-
CP013955.1	----------	----------	-
CP013953.1	----------	----------	-
CP014064.1	----------	----------	-
LT009690.1	----------	----------	-
AP017320.1	----------	----------	-
CP010890.1	----------	----------	-
CP001844.2	----------	----------	-
CP001781.1	----------	----------	-
AB488501.1	----------	----------	-
AB489898.1	----------	----------	-
HE579073.1	----------	----------	-
HE579071.1	----------	----------	-
HE579069.1	----------	----------	-
HE579065.1	----------	----------	-
HE579063.1	----------	----------	-

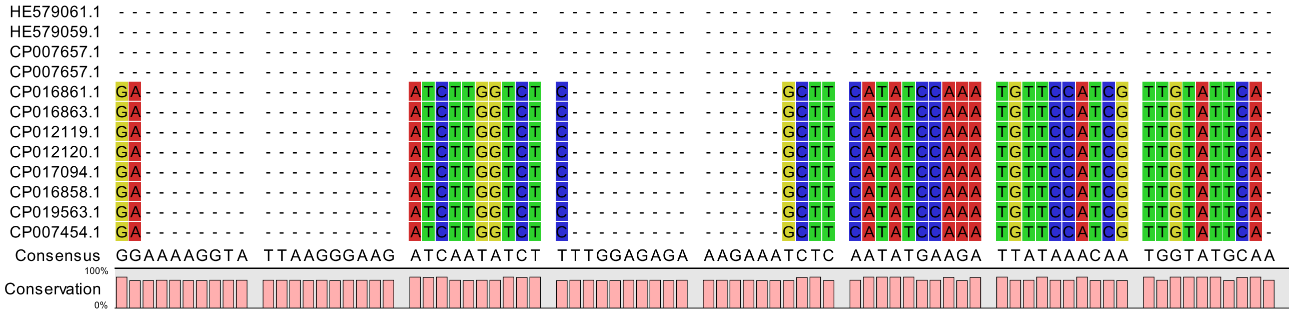
HE579061.1
HE579059.1
CP007657.1
CP007657.1
CP016861.1 GA ATCTTGGTCT C GCTT CATATCCAAA TGTTCCATCG TTGTATTCA
CP016863.1 GA ATCTTGGTCT C GCTT CATATCCAAA TGTTCCATCG TTGTATTCA
CP012119.1 GA ATCTTGGTCT C GCTT CATATCCAAA TGTTCCATCG TTGTATTCA
CP012120.1 GA ATCTTGGTCT C GCTT CATATCCAAA TGTTCCATCG TTGTATTCA
CP017094.1 GA ATCTTGGTCT C GCTT CATATCCAAA TGTTCCATCG TTGTATTCA
CP016858.1 GA ATCTTGGTCT C GCTT CATATCCAAA TGTTCCATCG TTGTATTCA
CP019563.1 GA ATCTTGGTCT C GCTT CATATCCAAA TGTTCCATCG TTGTATTCA
CP007454.1 GA ATCTTGGTCT C GCTT CATATCCAAA TGTTCCATCG TTGTATTCA
Consensus GGAAAAGGTA TTAAGGGAAG ATCAATATCT TTTGGAGAGA AAGAAATCTC AATATGAAGA TTATAAACAA TGGTATGCAA
100%
Conservation
0%

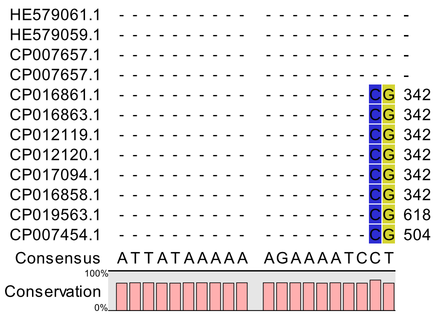
HE579061.1 -
HE579059.1 -
CP007657.1 -
CP007657.1 -
CP016861.1 - - - - - - - - - - - - - - - - - - - C G 342
CP016863.1 - - - - - - - - - - - - - - - - - - - C G 342
CP012119.1 - - - - - - - - - - - - - - - - - - - C G 342
CP012120.1 - - - - - - - - - - - - - - - - - - - C G 342
CP017094.1 - - - - - - - - - - - - - - - - - - - C G 342
CP016858.1 - - - - - - - - - - - - - - - - - - - C G 342
CP019563.1 - - - - - - - - - - - - - - - - - - - C G 618
CP007454.1 - - - - - - - - - - - - - - - - - - - C G 504
Consensus A T T A T A A A A A A G A A A A T C C T
100%
Conservation
0%

```
                                      720                                     740                                     760                                     780
                                        |                                       |                                       |                                       |
CP007670.1   CGTACAGATT TAAAAATGGC TAATTTTCAT AAATATAATT TAGAAGAACT TTCGATGAAA GAATACAATG AACTACAGGA
CP003033.1   CGTACAGATT TAAAAATGGC TAATTTTCAT AAATATAATT TAGAAGAACT TTCGATGAAA GAATACAATG AACTACAGGA
CP018205.1   CGTACAGATT TAAAAATGGC TAATTTTCAT AAATATAATT TAGAAGAACT TTCGATGAAA GAATACAATG AACTACAGGA
AP017377.1   CGTACAGATT TAAAAATGGC TAATTTTCAT AAATATAATT TAGAAGAACT TTCGATGAAA GAATACAATG AACTACAGGA
LT598688.1   CGTACAGATT TAAAAATGGC TAATTTTCAT AAATATAATT TAGAAGAACT TTCGATGAAA GAATACAATG AACTACAGGA
CP007676.1   CGTACAGATT TAAAAATGGC TAATTTTCAT AAATATAATT TAGAAGAACT TTCGATGAAA GAATACAATG AACTACAGGA
CP007672.1   CGTACAGATT TAAAAATGGC TAATTTTCAT AAATATAATT TAGAAGAACT TTCGATGAAA GAATACAATG AACTACAGGA
CP007674.1   CGTACAGATT TAAAAATGGC TAATTTTCAT AAATATAATT TAGAAGAACT TTCGATGAAA GAATACAATG AACTACAGGA
CP011526.1   CGTACAGATT TAAAAATGGC TAATTTTCAT AAATATAATT TAGAAGAACT TTCGATGAAA GAATACAATG AACTACAGGA
HF937103.1   CGTACAGATT TAAAAATGGC TAATTTTCAT AAATATAATT TAGAAGAACT TTCGATGAAA GAATACAATG AACTACAGGA
AP009351.1   CGTACAGATT TAAAAATGGC TAATTTTCAT AAATATAATT TAGAAGAACT TTCGATGAAA GAATACAATG AACTACAGGA
CP000046.1   CGTACAGATT TAAAAATGGC TAATTTTCAT AAATATAATT TAGAAGAACT TTCGATGAAA GAATACAATG AACTACAGGA
CP000253.1   CGTACAGATT TAAAAATGGC TAATTTTCAT AAATATAATT TAGAAGAACT TTCGATGAAA GAATACAATG AACTACAGGA
AC025591.8   CGTACAGATT TAAAAATGGC TAATTTTCAT AAATATAATT TAGAAGAACT TTCGATGAAA GAATACAATG AACTACAGGA
LT671859.1   CGTACAGATT TAAAAATGGC TAATTTTCAT AAATATAATT TAGAAGAACT TTCGATGAAA GAATACAATG AACTACAGGA
CP007499.1   CGTACAGATT TAAAAATGGC TAATTTTCAT AAATATAATT TAGAAGAACT TTCGATGAAA GAATACAATG AACTACAGGA
X17679.1     CGTACAGATT TAAAAATGGC TAATTTTCAT AAATATAATT TAGAAGAACT TTCGATGAAA GAATACAATG AACTACAGGA
CP007657.1   CGTACAGATT TAAAAATGGC TAATTTTCAT AAATATAATT TAGAAGAACT TTCGATGAAA GAATACAATG AACTACAGGA
CP014444.1   CGTACAGATT TAAAAATGGC TAATTTTCAT AAATATAATT TAGAAGAACT TTCGATGAAA GAATACAATG AACTACAGGA
CP014441.1   CGTACAGATT TAAAAATGGC TAATTTTCAT AAATATAATT TAGAAGAACT TTCGATGAAA GAATACAATG AACTACAGGA
CP014438.1   CGTACAGATT TAAAAATGGC TAATTTTCAT AAATATAATT TAGAAGAACT TTCGATGAAA GAATACAATG AACTACAGGA
CP014435.1   CGTACAGATT TAAAAATGGC TAATTTTCAT AAATATAATT TAGAAGAACT TTCGATGAAA GAATACAATG AACTACAGGA
CP014432.1   CGTACAGATT TAAAAATGGC TAATTTTCAT AAATATAATT TAGAAGAACT TTCGATGAAA GAATACAATG AACTACAGGA
CP014429.1   CGTACAGATT TAAAAATGGC TAATTTTCAT AAATATAATT TAGAAGAACT TTCGATGAAA GAATACAATG AACTACAGGA
CP014426.1   CGTACAGATT TAAAAATGGC TAATTTTCAT AAATATAATT TAGAAGAACT TTCGATGAAA GAATACAATG AACTACAGGA
CP014423.1   CGTACAGATT TAAAAATGGC TAATTTTCAT AAATATAATT TAGAAGAACT TTCGATGAAA GAATACAATG AACTACAGGA
CP014420.1   CGTACAGATT TAAAAATGGC TAATTTTCAT AAATATAATT TAGAAGAACT TTCGATGAAA GAATACAATG AACTACAGGA
CP014415.1   CGTACAGATT TAAAAATGGC TAATTTTCAT AAATATAATT TAGAAGAACT TTCGATGAAA GAATACAATG AACTACAGGA
CP014412.1   CGTACAGATT TAAAAATGGC TAATTTTCAT AAATATAATT TAGAAGAACT TTCGATGAAA GAATACAATG AACTACAGGA
CP014409.1   CGTACAGATT TAAAAATGGC TAATTTTCAT AAATATAATT TAGAAGAACT TTCGATGAAA GAATACAATG AACTACAGGA
CP014407.1   CGTACAGATT TAAAAATGGC TAATTTTCAT AAATATAATT TAGAAGAACT TTCGATGAAA GAATACAATG AACTACAGGA
CP014402.1   CGTACAGATT TAAAAATGGC TAATTTTCAT AAATATAATT TAGAAGAACT TTCGATGAAA GAATACAATG AACTACAGGA
CP014397.1   CGTACAGATT TAAAAATGGC TAATTTTCAT AAATATAATT TAGAAGAACT TTCGATGAAA GAATACAATG AACTACAGGA
CP014392.1   CGTACAGATT TAAAAATGGC TAATTTTCAT AAATATAATT TAGAAGAACT TTCGATGAAA GAATACAATG AACTACAGGA
CP014387.1   CGTACAGATT TAAAAATGGC TAATTTTCAT AAATATAATT TAGAAGAACT TTCGATGAAA GAATACAATG AACTACAGGA
CP014384.1   CGTACAGATT TAAAAATGGC TAATTTTCAT AAATATAATT TAGAAGAACT TTCGATGAAA GAATACAATG AACTACAGGA
CP014381.1   CGTACAGATT TAAAAATGGC TAATTTTCAT AAATATAATT TAGAAGAACT TTCGATGAAA GAATACAATG AACTACAGGA
CP014371.1   CGTACAGATT TAAAAATGGC TAATTTTCAT AAATATAATT TAGAAGAACT TTCGATGAAA GAATACAATG AACTACAGGA
CP014368.1   CGTACAGATT TAAAAATGGC TAATTTTCAT AAATATAATT TAGAAGAACT TTCGATGAAA GAATACAATG AACTACAGGA
CP014365.1   CGTACAGATT TAAAAATGGC TAATTTTCAT AAATATAATT TAGAAGAACT TTCGATGAAA GAATACAATG AACTACAGGA
CP014362.1   CGTACAGATT TAAAAATGGC TAATTTTCAT AAATATAATT TAGAAGAACT TTCGATGAAA GAATACAATG AACTACAGGA
CP014376.1   CGTACAGATT TAAAAATGGC TAATTTTCAT AAATATAATT TAGAAGAACT TTCGATGAAA GAATACAATG AACTACAGGA
CP009423.1   CGTACAGATT TAAAAATGGC TAATTTTCAT AAATATAATT TAGAAGAACT TTCGATGAAA GAATACAATG AACTACAGGA
CP016855.1   CGTACAGATT TAAAAATGGC TAATTTTCAT AAATATAATT TAGAAGAACT TTCGATGAAA GAATACAATG AACTACAGGA
CP013231.1   CGTACAGATT TAAAAATGGC TAATTTTCAT AAATATAATT TAGAAGAACT TTCGATGAAA GAATACAATG AACTACAGGA
CP010300.1   CGTACAGATT TAAAAATGGC TAATTTTCAT AAATATAATT TAGAAGAACT TTCGATGAAA GAATACAATG AACTACAGGA
CP010299.1   CGTACAGATT TAAAAATGGC TAATTTTCAT AAATATAATT TAGAAGAACT TTCGATGAAA GAATACAATG AACTACAGGA
CP010298.1   CGTACAGATT TAAAAATGGC TAATTTTCAT AAATATAATT TAGAAGAACT TTCGATGAAA GAATACAATG AACTACAGGA
CP010297.1   CGTACAGATT TAAAAATGGC TAATTTTCAT AAATATAATT TAGAAGAACT TTCGATGAAA GAATACAATG AACTACAGGA
CP010296.1   CGTACAGATT TAAAAATGGC TAATTTTCAT AAATATAATT TAGAAGAACT TTCGATGAAA GAATACAATG AACTACAGGA
CP010295.1   CGTACAGATT TAAAAATGGC TAATTTTCAT AAATATAATT TAGAAGAACT TTCGATGAAA GAATACAATG AACTACAGGA
CP007690.1   CGTACAGATT TAAAAATGGC TAATTTTCAT AAATATAATT TAGAAGAACT TTCGATGAAA GAATACAATG AACTACAGGA
CP007176.1   CGTACAGATT TAAAAATGGC TAATTTTCAT AAATATAATT TAGAAGAACT TTCGATGAAA GAATACAATG AACTACAGGA
CP000730.1   CGTACAGATT TAAAAATGGC TAATTTTCAT AAATATAATT TAGAAGAACT TTCGATGAAA GAATACAATG AACTACAGGA
CP000255.1   CGTACAGATT TAAAAATGGC TAATTTTCAT AAATATAATT TAGAAGAACT TTCGATGAAA GAATACAATG AACTACAGGA
CP007539.1   CGTACAGATT TAAAAATGGC TAATTTTCAT AAATATAATT TAGAAGAACT TTCGATGAAA GAATACAATG AACTACAGGA
CP007657.1   CGTACAGATT TAAAAATGGC TAATTTTCAT AAATATAATT TAGAAGAACT TTCGATGAAA GAATACAATG AACTACAGGA
AP014921.1   CGTACAGATT TAAAAATGGC TAATTTTCAT AAATATAATT TAGAAGAACT TTCGATGAAA GAATACAATG AACTACAGGA
AB436955.1   CGTACAGATT TAAAAATGGC TAATTTTCAT AAATATAATT TAGAAGAACT TTCGATGAAA GAATACAATG AACTACAGGA
AB489885.1   CGTACAGATT TAAAAATGGC TAATTTTCAT AAATATAATT TAGAAGAACT TTCGATGAAA GAATACAATG AACTACAGGA
AB489873.1   CGTACAGATT TAAAAATGGC TAATTTTCAT AAATATAATT TAGAAGAACT TTCGATGAAA GAATACAATG AACTACAGGA
AB489883.1   CGTACAGATT TAAAAATGGC TAATTTTCAT AAATATAATT TAGAAGAACT TTCGATGAAA GAATACAATG AACTACAGGA
AB489874.1   CGTACAGATT TAAAAATGGC TAATTTTCAT AAATATAATT TAGAAGAACT TTCGATGAAA GAATACAATG AACTACAGGA
LT615218.1   CGTACAGATT TAAAAATGGC TAATTTTCAT AAATATAATT TAGAAGAACT TTCGATGAAA GAATACAATG AACTACAGGA
AB436976.1   CGTACAGATT TAAAAATGGC TAATTTTCAT AAATATAATT TAGAAGAACT TTCGATGAAA GAATACAATG AACTACAGGA
AB489892.1   CGTACAGATT TAAAAATGGC TAATTTTCAT AAATATAATT TAGAAGAACT TTCGATGAAA GAATACAATG AACTACAGGA
AJ306908.1   CGTACAGATT TAAAAATGGC TAATTTTCAT AAATATAATT TAGAAGAACT TTCGATGAAA GAATACAATG AACTACAGGA
CP015646.1   CGTACAGATT TAAAAATGGC TAATTTTCAT AAATATAATT TAGAAGAACT TTCGATGAAA GAATACAATG AACTACAGGA
AB488510.1   CGTACAGATT TAAAAATGGC TAATTTTCAT AAATATAATT TAGAAGAACT TTCGATGAAA GAATACAATG AACTACAGGA
EU105387.1   CGTACAGATT TAAAAATGGC TAATTTTCAT AAATATAATT TAGAAGAACT TTCGATGAAA GAATACAATG AACTACAGGA
AB436975.1   CGTACAGATT TAAAAATGGC TAATTTTCAT AAATATAATT TAGAAGAACT TTCGATGAAA GAATACAATG AACTACAGAA
AB488499.1   CGTACAGATT TAAAAATGGC TAATTTTCAT AAATATAATT TAGAAGAACT TTCGATGAAA GAATACAATG AACTACAGAA
AJ309189.1   CGTACAGATT TAAAAATGGC TAATTTTCAT AAATATAATT TAGAAGAACT TTCGATGAAA GAATACAATG AACTACAGGA
FR821779.1   ---------- --AATATGCT TACTTTCCAT AAATATAATC TTTACAATTT AACAATGAAT GAATATAACG ATATTTTTAA
CP012593.1   ---------- ---------- ---------- ---------- ---------- ---------- ---------- ----------
CP012692.1   ---------- ---------- ---------- ---------- ---------- ---------- ---------- ----------
CP013955.1   ---------- ---------- ---------- ---------- ---------- ---------- ---------- ----------
CP013953.1   ---------- ---------- ---------- ---------- ---------- ---------- ---------- ----------
CP014064.1   ---------- ---------- ---------- ---------- ---------- ---------- ---------- ----------
LT009690.1   ---------- ---------- ---------- ---------- ---------- ---------- ---------- ----------
AP017320.1   ---------- ---------- ---------- ---------- ---------- ---------- ---------- ----------
CP010890.1   ---------- ---------- ---------- ---------- ---------- ---------- ---------- ----------
CP001844.2   ---------- ---------- ---------- ---------- ---------- ---------- ---------- ----------
CP001781.1   ---------- ---------- ---------- ---------- ---------- ---------- ---------- ----------
AB488501.1   ---------- ---------- ---------- ---------- ---------- ---------- ---------- ----------
AB489898.1   ---------- ---------- ---------- ---------- ---------- ---------- ---------- ----------
HE579073.1   ---------- ---------- ---------- ---------- ---------- ---------- ---------- ----------
HE579071.1   ---------- ---------- ---------- ---------- ---------- ---------- ---------- ----------
HE579069.1   ---------- ---------- ---------- ---------- ---------- ---------- ---------- ----------
HE579065.1   ---------- ---------- ---------- ---------- ---------- ---------- ---------- ----------
HE579063.1   ---------- ---------- ---------- ---------- ---------- ---------- ---------- ----------
```

		800	
CP007670.1	TGCATTAAAG	AGAGCACTGG	558
CP003033.1	TGCATTAAAG	AGAGCACTGG	558
CP018205.1	TGCATTAAAG	AGAGCACTGG	558
AP017377.1	TGCATTAAAG	AGAGCACTGG	558
LT598688.1	TGCATTAAAG	AGAGCACTGG	558
CP007676.1	TGCATTAAAG	AGAGCACTGG	558
CP007672.1	TGCATTAAAG	AGAGCACTGG	558
CP007674.1	TGCATTAAAG	AGAGCACTGG	558
CP011526.1	TGCATTAAAG	AGAGCACTGG	558
HF937103.1	TGCATTAAAG	AGAGCACTGG	558
AP009351.1	TGCATTAAAG	AGAGCACTGG	558
CP000046.1	TGCATTAAAG	AGAGCACTGG	558
CP000253.1	TGCATTAAAG	AGAGCACTGG	558
AC025591.8	TGCATTAAAG	AGAGCACTGG	558
LT671859.1	TGCATTAAAG	AGAGCACTGG	558
CP007499.1	TGCATTAAAG	AGAGCACTGG	558
X17679.1	TGCATTAAAG	AGAGCACTGG	558
CP007657.1	TGCATTAAAG	AGAGCACTGG	558
CP014444.1	TGCATTAAAG	AGAGCACTGG	558
CP014441.1	TGCATTAAAG	AGAGCACTGG	558
CP014438.1	TGCATTAAAG	AGAGCACTGG	558
CP014435.1	TGCATTAAAG	AGAGCACTGG	558
CP014432.1	TGCATTAAAG	AGAGCACTGG	558
CP014429.1	TGCATTAAAG	AGAGCACTGG	558
CP014426.1	TGCATTAAAG	AGAGCACTGG	558
CP014423.1	TGCATTAAAG	AGAGCACTGG	558
CP014420.1	TGCATTAAAG	AGAGCACTGG	558
CP014415.1	TGCATTAAAG	AGAGCACTGG	558
CP014412.1	TGCATTAAAG	AGAGCACTGG	558
CP014409.1	TGCATTAAAG	AGAGCACTGG	558
CP014407.1	TGCATTAAAG	AGAGCACTGG	558
CP014402.1	TGCATTAAAG	AGAGCACTGG	558
CP014397.1	TGCATTAAAG	AGAGCACTGG	558
CP014392.1	TGCATTAAAG	AGAGCACTGG	558
CP014387.1	TGCATTAAAG	AGAGCACTGG	558
CP014384.1	TGCATTAAAG	AGAGCACTGG	558
CP014381.1	TGCATTAAAG	AGAGCACTGG	558
CP014371.1	TGCATTAAAG	AGAGCACTGG	558
CP014368.1	TGCATTAAAG	AGAGCACTGG	558
CP014365.1	TGCATTAAAG	AGAGCACTGG	558
CP014362.1	TGCATTAAAG	AGAGCACTGG	558
CP014376.1	TGCATTAAAG	AGAGCACTGG	558
CP009423.1	TGCATTAAAG	AGAGCACTGG	558
CP016855.1	TGCATTAAAG	AGAGCACTGG	558
CP013231.1	TGCATTAAAG	AGAGCACTGG	558
CP010300.1	TGCATTAAAG	AGAGCACTGG	558
CP010299.1	TGCATTAAAG	AGAGCACTGG	558
CP010298.1	TGCATTAAAG	AGAGCACTGG	558
CP010297.1	TGCATTAAAG	AGAGCACTGG	558
CP010296.1	TGCATTAAAG	AGAGCACTGG	558
CP010295.1	TGCATTAAAG	AGAGCACTGG	558
CP007690.1	TGCATTAAAG	AGAGCACTGG	558
CP007176.1	TGCATTAAAG	AGAGCACTGG	558
CP000730.1	TGCATTAAAG	AGAGCACTGG	558
CP000255.1	TGCATTAAAG	AGAGCACTGG	558
CP007539.1	TGCATTAAAG	AGAGCACTGG	558
CP007657.1	TGCATTAAAG	AGAGCACTGG	559
AP014921.1	TGCATTAAAG	AGAGCACTGG	558
AB436955.1	TGCATTAAAG	AGAGCACTGG	466
AB489885.1	TGCATTAAAG	AGAGCACTGG	466
AB489873.1	TGCATTAAAG	AGAGCACTGG	466
AB489883.1	TGCATTAAAG	AGAGCACTGG	466
AB489874.1	TGCATTAAAG	AGAGCACTGG	466
LT615218.1	TGCATTAAAG	AGAGCACTGG	558
AB436976.1	TGCATTAAAG	AGAGCACTGG	558
AB489892.1	TGCATTAAAG	AGAGCACTGG	466
AJ306908.1	TGCATTAAAG	AGAGCACTGG	558
CP015646.1	TGCATTAAAG	AGAGCACTGG	558
AB488510.1	TGCATTAAAG	AGAGCACTGG	466
EU105387.1	TGCATTAAAG	AGAGCACTGG	498
AB436975.1	TGCATTAAAG	GGAGCACTGG	558
AB488499.1	TGCATTAAAG	GGAGCACTGG	466
AJ309189.1	TGCATTAAAG	AGAGCACTGG	382
FR821779.1	CTCTTTGAAA	GATGCAGTTT	543
CP012593.1	----------	----------	-
CP012692.1	----------	----------	-
CP013955.1	----------	----------	-
CP013953.1	----------	----------	-
CP014064.1	----------	----------	-
LT009690.1	----------	----------	-
AP017320.1	----------	----------	-
CP010890.1	----------	----------	-
CP001844.2	----------	----------	-
CP001781.1	----------	----------	-
AB488501.1	----------	----------	-
AB489898.1	----------	----------	-
HE579073.1	----------	----------	-
HE579071.1	----------	----------	-
HE579069.1	----------	----------	-
HE579065.1	----------	----------	-
HE579063.1	----------	----------	-

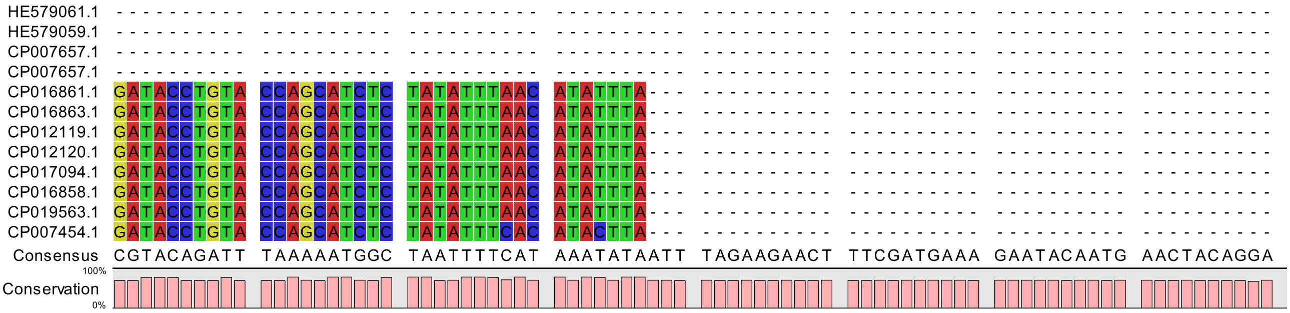
HE579061.1 ---------- ---------- ---------- ---------- ---------- ---------- ---------- ----------
HE579059.1 ---------- ---------- ---------- ---------- ---------- ---------- ---------- ----------
CP007657.1 ---------- ---------- ---------- ---------- ---------- ---------- ---------- ----------
CP007657.1 ---------- ---------- ---------- ---------- ---------- ---------- ---------- ----------
CP016861.1 GATACCTGTA CCAGCATCTC TATATTTAAC ATATTTA--- ---------- ---------- ---------- ----------
CP016863.1 GATACCTGTA CCAGCATCTC TATATTTAAC ATATTTA--- ---------- ---------- ---------- ----------
CP012119.1 GATACCTGTA CCAGCATCTC TATATTTAAC ATATTTA--- ---------- ---------- ---------- ----------
CP012120.1 GATACCTGTA CCAGCATCTC TATATTTAAC ATATTTA--- ---------- ---------- ---------- ----------
CP017094.1 GATACCTGTA CCAGCATCTC TATATTTAAC ATATTTA--- ---------- ---------- ---------- ----------
CP016858.1 GATACCTGTA CCAGCATCTC TATATTTAAC ATATTTA--- ---------- ---------- ---------- ----------
CP019563.1 GATACCTGTA CCAGCATCTC TATATTTAAC ATATTTA--- ---------- ---------- ---------- ----------
CP007454.1 GATACCTGTA CCAGCATCTC TATATTTCAC ATACTTA--- ---------- ---------- ---------- ----------
Consensus CGTACAGATT TAAAAATGGC TAATTTTCAT AAATATAATT TAGAAGAACT TTCGATGAAA GAATACAATG AACTACAGGA
100%
Conservation
0%

HE579061.1	- - - - - - - - - -	- - - - - - - - - -	-
HE579059.1	- - - - - - - - - -	- - - - - - - - - -	-
CP007657.1	- - - - - - - - - -	- - - - - - - - - -	-
CP007657.1	- - - - - - - - - -	- - - - - - - - - -	-
CP016861.1	- - - - - - - - - -	- - - - - - - - - -	379
CP016863.1	- - - - - - - - - -	- - - - - - - - - -	379
CP012119.1	- - - - - - - - - -	- - - - - - - - - -	379
CP012120.1	- - - - - - - - - -	- - - - - - - - - -	379
CP017094.1	- - - - - - - - - -	- - - - - - - - - -	379
CP016858.1	- - - - - - - - - -	- - - - - - - - - -	379
CP019563.1	- - - - - - - - - -	- - - - - - - - - -	655
CP007454.1	- - - - - - - - - -	- - - - - - - - - -	541
Consensus	TGCATTAAAG	AGAGCACTGG	

Conservation

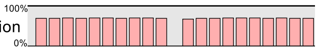

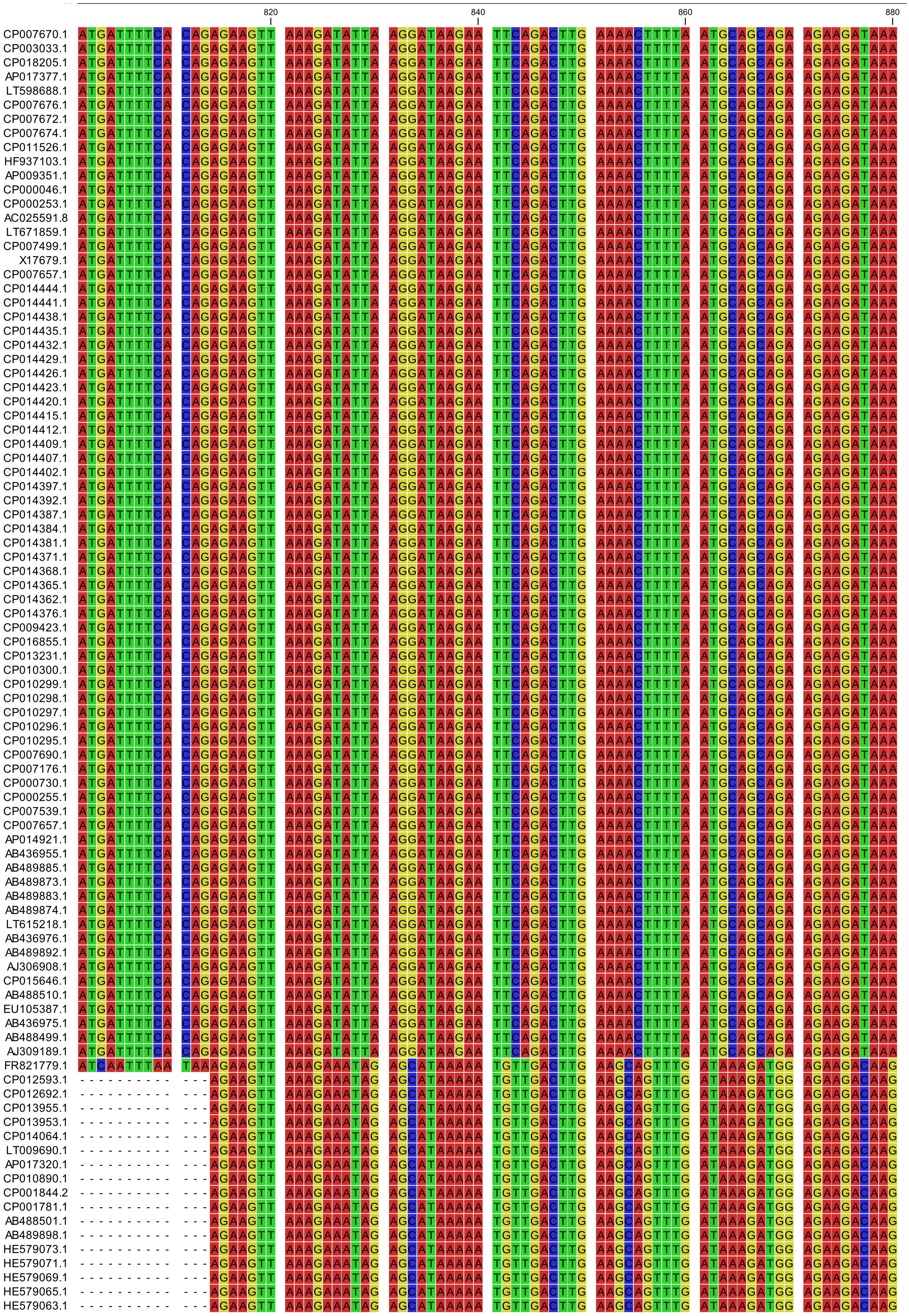
820
840
860
880
CP007670.1 ATGATTTTCA CAGAGAAGTT AAAGATATTA AGGATAAGAA TTCAGACTTG AAAACTTTTA ATGCAGCAGA AGAAGATAAA
CP003033.1 ATGATTTTCA CAGAGAAGTT AAAGATATTA AGGATAAGAA TTCAGACTTG AAAACTTTTA ATGCAGCAGA AGAAGATAAA
CP018205.1 ATGATTTTCA CAGAGAAGTT AAAGATATTA AGGATAAGAA TTCAGACTTG AAAACTTTTA ATGCAGCAGA AGAAGATAAA
AP017377.1 ATGATTTTCA CAGAGAAGTT AAAGATATTA AGGATAAGAA TTCAGACTTG AAAACTTTTA ATGCAGCAGA AGAAGATAAA
LT598688.1 ATGATTTTCA CAGAGAAGTT AAAGATATTA AGGATAAGAA TTCAGACTTG AAAACTTTTA ATGCAGCAGA AGAAGATAAA
CP007676.1 ATGATTTTCA CAGAGAAGTT AAAGATATTA AGGATAAGAA TTCAGACTTG AAAACTTTTA ATGCAGCAGA AGAAGATAAA
CP007672.1 ATGATTTTCA CAGAGAAGTT AAAGATATTA AGGATAAGAA TTCAGACTTG AAAACTTTTA ATGCAGCAGA AGAAGATAAA
CP007674.1 ATGATTTTCA CAGAGAAGTT AAAGATATTA AGGATAAGAA TTCAGACTTG AAAACTTTTA ATGCAGCAGA AGAAGATAAA
CP011526.1 ATGATTTTCA CAGAGAAGTT AAAGATATTA AGGATAAGAA TTCAGACTTG AAAACTTTTA ATGCAGCAGA AGAAGATAAA
HF937103.1 ATGATTTTCA CAGAGAAGTT AAAGATATTA AGGATAAGAA TTCAGACTTG AAAACTTTTA ATGCAGCAGA AGAAGATAAA
AP009351.1 ATGATTTTCA CAGAGAAGTT AAAGATATTA AGGATAAGAA TTCAGACTTG AAAACTTTTA ATGCAGCAGA AGAAGATAAA
CP000046.1 ATGATTTTCA CAGAGAAGTT AAAGATATTA AGGATAAGAA TTCAGACTTG AAAACTTTTA ATGCAGCAGA AGAAGATAAA
CP000253.1 ATGATTTTCA CAGAGAAGTT AAAGATATTA AGGATAAGAA TTCAGACTTG AAAACTTTTA ATGCAGCAGA AGAAGATAAA
AC025591.8 ATGATTTTCA CAGAGAAGTT AAAGATATTA AGGATAAGAA TTCAGACTTG AAAACTTTTA ATGCAGCAGA AGAAGATAAA
LT671859.1 ATGATTTTCA CAGAGAAGTT AAAGATATTA AGGATAAGAA TTCAGACTTG AAAACTTTTA ATGCAGCAGA AGAAGATAAA
CP007499.1 ATGATTTTCA CAGAGAAGTT AAAGATATTA AGGATAAGAA TTCAGACTTG AAAACTTTTA ATGCAGCAGA AGAAGATAAA
X17679.1 ATGATTTTCA CAGAGAAGTT AAAGATATTA AGGATAAGAA TTCAGACTTG AAAACTTTTA ATGCAGCAGA AGAAGATAAA
CP007657.1 ATGATTTTCA CAGAGAAGTT AAAGATATTA AGGATAAGAA TTCAGACTTG AAAACTTTTA ATGCAGCAGA AGAAGATAAA
CP014444.1 ATGATTTTCA CAGAGAAGTT AAAGATATTA AGGATAAGAA TTCAGACTTG AAAACTTTTA ATGCAGCAGA AGAAGATAAA
CP014441.1 ATGATTTTCA CAGAGAAGTT AAAGATATTA AGGATAAGAA TTCAGACTTG AAAACTTTTA ATGCAGCAGA AGAAGATAAA
CP014438.1 ATGATTTTCA CAGAGAAGTT AAAGATATTA AGGATAAGAA TTCAGACTTG AAAACTTTTA ATGCAGCAGA AGAAGATAAA
CP014435.1 ATGATTTTCA CAGAGAAGTT AAAGATATTA AGGATAAGAA TTCAGACTTG AAAACTTTTA ATGCAGCAGA AGAAGATAAA
CP014432.1 ATGATTTTCA CAGAGAAGTT AAAGATATTA AGGATAAGAA TTCAGACTTG AAAACTTTTA ATGCAGCAGA AGAAGATAAA
CP014429.1 ATGATTTTCA CAGAGAAGTT AAAGATATTA AGGATAAGAA TTCAGACTTG AAAACTTTTA ATGCAGCAGA AGAAGATAAA
CP014426.1 ATGATTTTCA CAGAGAAGTT AAAGATATTA AGGATAAGAA TTCAGACTTG AAAACTTTTA ATGCAGCAGA AGAAGATAAA
CP014423.1 ATGATTTTCA CAGAGAAGTT AAAGATATTA AGGATAAGAA TTCAGACTTG AAAACTTTTA ATGCAGCAGA AGAAGATAAA
CP014420.1 ATGATTTTCA CAGAGAAGTT AAAGATATTA AGGATAAGAA TTCAGACTTG AAAACTTTTA ATGCAGCAGA AGAAGATAAA
CP014415.1 ATGATTTTCA CAGAGAAGTT AAAGATATTA AGGATAAGAA TTCAGACTTG AAAACTTTTA ATGCAGCAGA AGAAGATAAA
CP014412.1 ATGATTTTCA CAGAGAAGTT AAAGATATTA AGGATAAGAA TTCAGACTTG AAAACTTTTA ATGCAGCAGA AGAAGATAAA
CP014409.1 ATGATTTTCA CAGAGAAGTT AAAGATATTA AGGATAAGAA TTCAGACTTG AAAACTTTTA ATGCAGCAGA AGAAGATAAA
CP014407.1 ATGATTTTCA CAGAGAAGTT AAAGATATTA AGGATAAGAA TTCAGACTTG AAAACTTTTA ATGCAGCAGA AGAAGATAAA
CP014402.1 ATGATTTTCA CAGAGAAGTT AAAGATATTA AGGATAAGAA TTCAGACTTG AAAACTTTTA ATGCAGCAGA AGAAGATAAA
CP014397.1 ATGATTTTCA CAGAGAAGTT AAAGATATTA AGGATAAGAA TTCAGACTTG AAAACTTTTA ATGCAGCAGA AGAAGATAAA
CP014392.1 ATGATTTTCA CAGAGAAGTT AAAGATATTA AGGATAAGAA TTCAGACTTG AAAACTTTTA ATGCAGCAGA AGAAGATAAA
CP014387.1 ATGATTTTCA CAGAGAAGTT AAAGATATTA AGGATAAGAA TTCAGACTTG AAAACTTTTA ATGCAGCAGA AGAAGATAAA
CP014384.1 ATGATTTTCA CAGAGAAGTT AAAGATATTA AGGATAAGAA TTCAGACTTG AAAACTTTTA ATGCAGCAGA AGAAGATAAA
CP014381.1 ATGATTTTCA CAGAGAAGTT AAAGATATTA AGGATAAGAA TTCAGACTTG AAAACTTTTA ATGCAGCAGA AGAAGATAAA
CP014371.1 ATGATTTTCA CAGAGAAGTT AAAGATATTA AGGATAAGAA TTCAGACTTG AAAACTTTTA ATGCAGCAGA AGAAGATAAA
CP014368.1 ATGATTTTCA CAGAGAAGTT AAAGATATTA AGGATAAGAA TTCAGACTTG AAAACTTTTA ATGCAGCAGA AGAAGATAAA
CP014365.1 ATGATTTTCA CAGAGAAGTT AAAGATATTA AGGATAAGAA TTCAGACTTG AAAACTTTTA ATGCAGCAGA AGAAGATAAA
CP014362.1 ATGATTTTCA CAGAGAAGTT AAAGATATTA AGGATAAGAA TTCAGACTTG AAAACTTTTA ATGCAGCAGA AGAAGATAAA
CP014376.1 ATGATTTTCA CAGAGAAGTT AAAGATATTA AGGATAAGAA TTCAGACTTG AAAACTTTTA ATGCAGCAGA AGAAGATAAA
CP009423.1 ATGATTTTCA CAGAGAAGTT AAAGATATTA AGGATAAGAA TTCAGACTTG AAAACTTTTA ATGCAGCAGA AGAAGATAAA
CP016855.1 ATGATTTTCA CAGAGAAGTT AAAGATATTA AGGATAAGAA TTCAGACTTG AAAACTTTTA ATGCAGCAGA AGAAGATAAA
CP013231.1 ATGATTTTCA CAGAGAAGTT AAAGATATTA AGGATAAGAA TTCAGACTTG AAAACTTTTA ATGCAGCAGA AGAAGATAAA
CP010300.1 ATGATTTTCA CAGAGAAGTT AAAGATATTA AGGATAAGAA TTCAGACTTG AAAACTTTTA ATGCAGCAGA AGAAGATAAA
CP010299.1 ATGATTTTCA CAGAGAAGTT AAAGATATTA AGGATAAGAA TTCAGACTTG AAAACTTTTA ATGCAGCAGA AGAAGATAAA
CP010298.1 ATGATTTTCA CAGAGAAGTT AAAGATATTA AGGATAAGAA TTCAGACTTG AAAACTTTTA ATGCAGCAGA AGAAGATAAA
CP010297.1 ATGATTTTCA CAGAGAAGTT AAAGATATTA AGGATAAGAA TTCAGACTTG AAAACTTTTA ATGCAGCAGA AGAAGATAAA
CP010296.1 ATGATTTTCA CAGAGAAGTT AAAGATATTA AGGATAAGAA TTCAGACTTG AAAACTTTTA ATGCAGCAGA AGAAGATAAA
CP010295.1 ATGATTTTCA CAGAGAAGTT AAAGATATTA AGGATAAGAA TTCAGACTTG AAAACTTTTA ATGCAGCAGA AGAAGATAAA
CP007690.1 ATGATTTTCA CAGAGAAGTT AAAGATATTA AGGATAAGAA TTCAGACTTG AAAACTTTTA ATGCAGCAGA AGAAGATAAA
CP007176.1 ATGATTTTCA CAGAGAAGTT AAAGATATTA AGGATAAGAA TTCAGACTTG AAAACTTTTA ATGCAGCAGA AGAAGATAAA
CP000730.1 ATGATTTTCA CAGAGAAGTT AAAGATATTA AGGATAAGAA TTCAGACTTG AAAACTTTTA ATGCAGCAGA AGAAGATAAA
CP000255.1 ATGATTTTCA CAGAGAAGTT AAAGATATTA AGGATAAGAA TTCAGACTTG AAAACTTTTA ATGCAGCAGA AGAAGATAAA
CP007539.1 ATGATTTTCA CAGAGAAGTT AAAGATATTA AGGATAAGAA TTCAGACTTG AAAACTTTTA ATGCAGCAGA AGAAGATAAA
CP007657.1 ATGATTTTCA CAGAGAAGTT AAAGATATTA AGGATAAGAA TTCAGACTTG AAAACTTTTA ATGCAGCAGA AGAAGATAAA
AP014921.1 ATGATTTTCA CAGAGAAGTT AAAGATATTA AGGATAAGAA TTCAGACTTG AAAACTTTTA ATGCAGCAGA AGAAGATAAA
AB436955.1 ATGATTTTCA CAGAGAAGTT AAAGATATTA AGGATAAGAA TTCAGACTTG AAAACTTTTA ATGCAGCAGA AGAAGATAAA
AB489885.1 ATGATTTTCA CAGAGAAGTT AAAGATATTA AGGATAAGAA TTCAGACTTG AAAACTTTTA ATGCAGCAGA AGAAGATAAA
AB489873.1 ATGATTTTCA CAGAGAAGTT AAAGATATTA AGGATAAGAA TTCAGACTTG AAAACTTTTA ATGCAGCAGA AGAAGATAAA
AB489883.1 ATGATTTTCA CAGAGAAGTT AAAGATATTA AGGATAAGAA TTCAGACTTG AAAACTTTTA ATGCAGCAGA AGAAGATAAA
AB489874.1 ATGATTTTCA CAGAGAAGTT AAAGATATTA AGGATAAGAA TTCAGACTTG AAAACTTTTA ATGCAGCAGA AGAAGATAAA
LT615218.1 ATGATTTTCA CAGAGAAGTT AAAGATATTA AGGATAAGAA TTCAGACTTG AAAACTTTTA ATGCAGCAGA AGAAGATAAA
AB436976.1 ATGATTTTCA CAGAGAAGTT AAAGATATTA AGGATAAGAA TTCAGACTTG AAAACTTTTA ATGCAGCAGA AGAAGATAAA
AB489892.1 ATGATTTTCA CAGAGAAGTT AAAGATATTA AGGATAAGAA TTCAGACTTG AAAACTTTTA ATGCAGCAGA AGAAGATAAA
AJ306908.1 ATGATTTTCA CAGAGAAGTT AAAGATATTA AGGATAAGAA TTCAGACTTG AAAACTTTTA ATGCAGCAGA AGAAGATAAA
CP015646.1 ATGATTTTCA CAGAGAAGTT AAAGATATTA AGGATAAGAA TTCAGACTTG AAAACTTTTA ATGCAGCAGA AGAAGATAAA
AB488510.1 ATGATTTTCA CAGAGAAGTT AAAGATATTA AGGATAAGAA TTCAGACTTG AAAACTTTTA ATGCAGCAGA AGAAGATAAA
EU105387.1 ATGATTTTCA CAGAGAAGTT AAAGATATTA AGGATAAGAA TTCAGACTTG AAAACTTTTA ATGCAGCAGA AGAAGATAAA
AB436975.1 ATGATTTTCA CAGAGAAGTT AAAGATATTA AGGATAAGAA TTCAGACTTG AAAACTTTTA ATGCAGCAGA AGAAGATAAA
AB488499.1 ATGATTTTCA CAGAGAAGTT AAAGATATTA AGGATAAGAA TTCAGACTTG AAAACTTTTA ATGCAGCAGA AGAAGATAAA
AJ309189.1 ATGATTTTCA CAGAGAAGTT AAAGATATTA AGGATAAGAA TTCAGACTTG AAAACTTTTA ATGCAGCAGA AGAAGATAAA
FR821779.1 ATCAATTTAA TAAAGAAGTT AAAGAAATAG AGCATAAAAA TGTTGACTTG AAGCAGTTTG ATAAAGATGG AGAAGACAAG
CP012593.1 ---------- ---AGAAGTT AAAGAAATAG AGCATAAAAA TGTTGACTTG AAGCAGTTTG ATAAAGATGG AGAAGACAAG
CP012692.1 ---------- ---AGAAGTT AAAGAAATAG AGCATAAAAA TGTTGACTTG AAGCAGTTTG ATAAAGATGG AGAAGACAAG
CP013955.1 ---------- ---AGAAGTT AAAGAAATAG AGCATAAAAA TGTTGACTTG AAGCAGTTTG ATAAAGATGG AGAAGACAAG
CP013953.1 ---------- ---AGAAGTT AAAGAAATAG AGCATAAAAA TGTTGACTTG AAGCAGTTTG ATAAAGATGG AGAAGACAAG
CP014064.1 ---------- ---AGAAGTT AAAGAAATAG AGCATAAAAA TGTTGACTTG AAGCAGTTTG ATAAAGATGG AGAAGACAAG
LT009690.1 ---------- ---AGAAGTT AAAGAAATAG AGCATAAAAA TGTTGACTTG AAGCAGTTTG ATAAAGATGG AGAAGACAAG
AP017320.1 ---------- ---AGAAGTT AAAGAAATAG AGCATAAAAA TGTTGACTTG AAGCAGTTTG ATAAAGATGG AGAAGACAAG
CP010890.1 ---------- ---AGAAGTT AAAGAAATAG AGCATAAAAA TGTTGACTTG AAGCAGTTTG ATAAAGATGG AGAAGACAAG
CP001844.2 ---------- ---AGAAGTT AAAGAAATAG AGCATAAAAA TGTTGACTTG AAGCAGTTTG ATAAAGATGG AGAAGACAAG
CP001781.1 ---------- ---AGAAGTT AAAGAAATAG AGCATAAAAA TGTTGACTTG AAGCAGTTTG ATAAAGATGG AGAAGACAAG
AB488501.1 ---------- ---AGAAGTT AAAGAAATAG AGCATAAAAA TGTTGACTTG AAGCAGTTTG ATAAAGATGG AGAAGACAAG
AB489898.1 ---------- ---AGAAGTT AAAGAAATAG AGCATAAAAA TGTTGACTTG AAGCAGTTTG ATAAAGATGG AGAAGACAAG
HE579073.1 ---------- ---AGAAGTT AAAGAAATAG AGCATAAAAA TGTTGACTTG AAGCAGTTTG ATAAAGATGG AGAAGACAAG
HE579071.1 ---------- ---AGAAGTT AAAGAAATAG AGCATAAAAA TGTTGACTTG AAGCAGTTTG ATAAAGATGG AGAAGACAAG
HE579069.1 ---------- ---AGAAGTT AAAGAAATAG AGCATAAAAA TGTTGACTTG AAGCAGTTTG ATAAAGATGG AGAAGACAAG
HE579065.1 ---------- ---AGAAGTT AAAGAAATAG AGCATAAAAA TGTTGACTTG AAGCAGTTTG ATAAAGATGG AGAAGACAAG
HE579063.1 ---------- ---AGAAGTT AAAGAAATAG AGCATAAAAA TGTTGACTTG AAGCAGTTTG ATAAAGATGG AGAAGACAAG

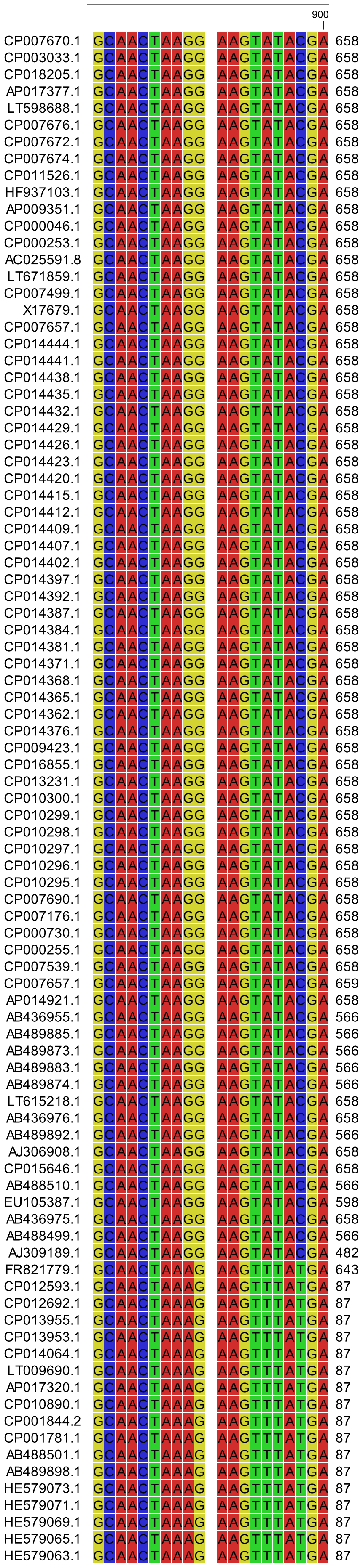
900
CP007670.1 GCAACTAAGG AAGTATACGA 658
CP003033.1 GCAACTAAGG AAGTATACGA 658
CP018205.1 GCAACTAAGG AAGTATACGA 658
AP017377.1 GCAACTAAGG AAGTATACGA 658
LT598688.1 GCAACTAAGG AAGTATACGA 658
CP007676.1 GCAACTAAGG AAGTATACGA 658
CP007672.1 GCAACTAAGG AAGTATACGA 658
CP007674.1 GCAACTAAGG AAGTATACGA 658
CP011526.1 GCAACTAAGG AAGTATACGA 658
HF937103.1 GCAACTAAGG AAGTATACGA 658
AP009351.1 GCAACTAAGG AAGTATACGA 658
CP000046.1 GCAACTAAGG AAGTATACGA 658
CP000253.1 GCAACTAAGG AAGTATACGA 658
AC025591.8 GCAACTAAGG AAGTATACGA 658
LT671859.1 GCAACTAAGG AAGTATACGA 658
CP007499.1 GCAACTAAGG AAGTATACGA 658
X17679.1 GCAACTAAGG AAGTATACGA 658
CP007657.1 GCAACTAAGG AAGTATACGA 658
CP014444.1 GCAACTAAGG AAGTATACGA 658
CP014441.1 GCAACTAAGG AAGTATACGA 658
CP014438.1 GCAACTAAGG AAGTATACGA 658
CP014435.1 GCAACTAAGG AAGTATACGA 658
CP014432.1 GCAACTAAGG AAGTATACGA 658
CP014429.1 GCAACTAAGG AAGTATACGA 658
CP014426.1 GCAACTAAGG AAGTATACGA 658
CP014423.1 GCAACTAAGG AAGTATACGA 658
CP014420.1 GCAACTAAGG AAGTATACGA 658
CP014415.1 GCAACTAAGG AAGTATACGA 658
CP014412.1 GCAACTAAGG AAGTATACGA 658
CP014409.1 GCAACTAAGG AAGTATACGA 658
CP014407.1 GCAACTAAGG AAGTATACGA 658
CP014402.1 GCAACTAAGG AAGTATACGA 658
CP014397.1 GCAACTAAGG AAGTATACGA 658
CP014392.1 GCAACTAAGG AAGTATACGA 658
CP014387.1 GCAACTAAGG AAGTATACGA 658
CP014384.1 GCAACTAAGG AAGTATACGA 658
CP014381.1 GCAACTAAGG AAGTATACGA 658
CP014371.1 GCAACTAAGG AAGTATACGA 658
CP014368.1 GCAACTAAGG AAGTATACGA 658
CP014365.1 GCAACTAAGG AAGTATACGA 658
CP014362.1 GCAACTAAGG AAGTATACGA 658
CP014376.1 GCAACTAAGG AAGTATACGA 658
CP009423.1 GCAACTAAGG AAGTATACGA 658
CP016855.1 GCAACTAAGG AAGTATACGA 658
CP013231.1 GCAACTAAGG AAGTATACGA 658
CP010300.1 GCAACTAAGG AAGTATACGA 658
CP010299.1 GCAACTAAGG AAGTATACGA 658
CP010298.1 GCAACTAAGG AAGTATACGA 658
CP010297.1 GCAACTAAGG AAGTATACGA 658
CP010296.1 GCAACTAAGG AAGTATACGA 658
CP010295.1 GCAACTAAGG AAGTATACGA 658
CP007690.1 GCAACTAAGG AAGTATACGA 658
CP007176.1 GCAACTAAGG AAGTATACGA 658
CP000730.1 GCAACTAAGG AAGTATACGA 658
CP000255.1 GCAACTAAGG AAGTATACGA 658
CP007539.1 GCAACTAAGG AAGTATACGA 658
CP007657.1 GCAACTAAGG AAGTATACGA 659
AP014921.1 GCAACTAAGG AAGTATACGA 658
AB436955.1 GCAACTAAGG AAGTATACGA 566
AB489885.1 GCAACTAAGG AAGTATACGA 566
AB489873.1 GCAACTAAGG AAGTATACGA 566
AB489883.1 GCAACTAAGG AAGTATACGA 566
AB489874.1 GCAACTAAGG AAGTATACGA 566
LT615218.1 GCAACTAAGG AAGTATACGA 658
AB436976.1 GCAACTAAGG AAGTATACGA 658
AB489892.1 GCAACTAAGG AAGTATACGA 566
AJ306908.1 GCAACTAAGG AAGTATACGA 658
CP015646.1 GCAACTAAGG AAGTATACGA 658
AB488510.1 GCAACTAAGG AAGTATACGA 566
EU105387.1 GCAACTAAGG AAGTATACGA 598
AB436975.1 GCAACTAAGG AAGTATACGA 658
AB488499.1 GCAACTAAGG AAGTATACGA 566
AJ309189.1 GCAACTAAGG AAGTATACGA 482
FR821779.1 GCAACTAAAG AAGTTTATGA 643
CP012593.1 GCAACTAAAG AAGTTTATGA 87
CP012692.1 GCAACTAAAG AAGTTTATGA 87
CP013955.1 GCAACTAAAG AAGTTTATGA 87
CP013953.1 GCAACTAAAG AAGTTTATGA 87
CP014064.1 GCAACTAAAG AAGTTTATGA 87
LT009690.1 GCAACTAAAG AAGTTTATGA 87
AP017320.1 GCAACTAAAG AAGTTTATGA 87
CP010890.1 GCAACTAAAG AAGTTTATGA 87
CP001844.2 GCAACTAAAG AAGTTTATGA 87
CP001781.1 GCAACTAAAG AAGTTTATGA 87
AB488501.1 GCAACTAAAG AAGTTTATGA 87
AB489898.1 GCAACTAAAG AAGTTTATGA 87
HE579073.1 GCAACTAAAG AAGTTTATGA 87
HE579071.1 GCAACTAAAG AAGTTTATGA 87
HE579069.1 GCAACTAAAG AAGTTTATGA 87
HE579065.1 GCAACTAAAG AAGTTTATGA 87
HE579063.1 GCAACTAAAG AAGTTTATGA 87

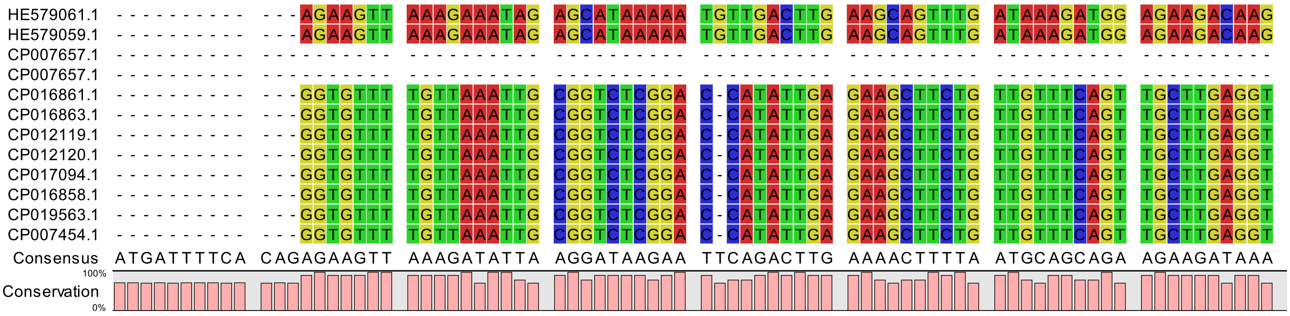
HE579061.1 ---------- ---AGAAGTT AAAGAAATAG AGCATAAAAA TGTTGACTTG AAGCAGTTTG ATAAAGATGG AGAAGACAAG
HE579059.1 ---------- ---AGAAGTT AAAGAAATAG AGCATAAAAA TGTTGACTTG AAGCAGTTTG ATAAAGATGG AGAAGACAAG
CP007657.1 ---------- ---------- ---------- ---------- ---------- ---------- ---------- ----------
CP007657.1 ---------- ---------- ---------- ---------- ---------- ---------- ---------- ----------
CP016861.1 ---------- ---GGTGTTT TGTTAAATTG CGGTCTCGGA C-CATATTGA GAAGCTTCTG TTGTTTCAGT TGCTTGAGGT
CP016863.1 ---------- ---GGTGTTT TGTTAAATTG CGGTCTCGGA C-CATATTGA GAAGCTTCTG TTGTTTCAGT TGCTTGAGGT
CP012119.1 ---------- ---GGTGTTT TGTTAAATTG CGGTCTCGGA C-CATATTGA GAAGCTTCTG TTGTTTCAGT TGCTTGAGGT
CP012120.1 ---------- ---GGTGTTT TGTTAAATTG CGGTCTCGGA C-CATATTGA GAAGCTTCTG TTGTTTCAGT TGCTTGAGGT
CP017094.1 ---------- ---GGTGTTT TGTTAAATTG CGGTCTCGGA C-CATATTGA GAAGCTTCTG TTGTTTCAGT TGCTTGAGGT
CP016858.1 ---------- ---GGTGTTT TGTTAAATTG CGGTCTCGGA C-CATATTGA GAAGCTTCTG TTGTTTCAGT TGCTTGAGGT
CP019563.1 ---------- ---GGTGTTT TGTTAAATTG CGGTCTCGGA C-CATATTGA GAAGCTTCTG TTGTTTCAGT TGCTTGAGGT
CP007454.1 ---------- ---GGTGTTT TGTTAAATTG CGGTCTCGGA C-CATATTGA GAAGCTTCTG TTGTTTCAGT TGCTTGAGGT
Consensus ATGATTTTCA CAGAGAAGTT AAAGATATTA AGGATAAGAA TTCAGACTTG AAAACTTTTA ATGCAGCAGA AGAAGATAAA
Conservation
100%
0%

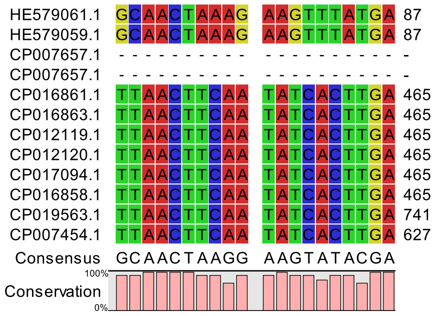
HE579061.1 GCAACTAAAG AAGTTTATGA 87
HE579059.1 GCAACTAAAG AAGTTTATGA 87
CP007657.1 -
CP007657.1 -
CP016861.1 TTAACTTCAA TATCACTTGA 465
CP016863.1 TTAACTTCAA TATCACTTGA 465
CP012119.1 TTAACTTCAA TATCACTTGA 465
CP012120.1 TTAACTTCAA TATCACTTGA 465
CP017094.1 TTAACTTCAA TATCACTTGA 465
CP016858.1 TTAACTTCAA TATCACTTGA 465
CP019563.1 TTAACTTCAA TATCACTTGA 741
CP007454.1 TTAACTTCAA TATCACTTGA 627
Consensus GCAACTAAGG AAGTATACGA
100%
Conservation
0%

```
                    920         940         960         980
CP007670.1  TCTCGTATCT GAAATTGATA CATTAGTTGT ATCATATTAT GGTGATA--- -AGGATTATG GGGAGCACGC GAAAGAGTTA
CP003033.1  TCTCGTATCT GAAATTGATA CATTAGTTGT ATCATATTAT GGTGATA--- -AGGATTATG GGGAGCACGC GAAAGAGTTA
CP018205.1  TCTCGTATCT GAAATTGATA CATTAGTTGT ATCATATTAT GGTGATA--- -AGGATTATG GGGAGCACGC GAAAGAGTTA
AP017377.1  TCTCGTATCT GAAATTGATA CATTAGTTGT ATCATATTAT GGTGATA--- -AGGATTATG GGGAGCACGC GAAAGAGTTA
LT598688.1  TCTCGTATCT GAAATTGATA CATTAGTTGT ATCATATTAT GGTGATA--- -AGGATTATG GGGAGCACGC GAAAGAGTTA
CP007676.1  TCTCGTATCT GAAATTGATA CATTAGTTGT ATCATATTAT GGTGATA--- -AGGATTATG GGGAGCACGC GAAAGAGTTA
CP007672.1  TCTCGTATCT GAAATTGATA CATTAGTTGT ATCATATTAT GGTGATA--- -AGGATTATG GGGAGCACGC GAAAGAGTTA
CP007674.1  TCTCGTATCT GAAATTGATA CATTAGTTGT ATCATATTAT GGTGATA--- -AGGATTATG GGGAGCACGC GAAAGAGTTA
CP011526.1  TCTCGTATCT GAAATTGATA CATTAGTTGT ATCATATTAT GGTGATA--- -AGGATTATG GGGAGCACGC GAAAGAGTTA
HF937103.1  TCTCGTATCT GAAATTGATA CATTAGTTGT ATCATATTAT GGTGATA--- -AGGATTATG GGGAGCACGC GAAAGAGTTA
AP009351.1  TCTCGTATCT GAAATTGATA CATTAGTTGT ATCATATTAT GGTGATA--- -AGGATTATG GGGAGCACGC GAAAGAGTTA
CP000046.1  TCTCGTATCT GAAATTGATA CATTAGTTGT ATCATATTAT GGTGATA--- -AGGATTATG GGGAGCACGC GAAAGAGTTA
CP000253.1  TCTCGTATCT GAAATTGATA CATTAGTTGT ATCATATTAT GGTGATA--- -AGGATTATG GGGAGCACGC GAAAGAGTTA
AC025591.8  TCTCGTATCT GAAATTGATA CATTAGTTGT ATCATATTAT GGTGATA--- -AGGATTATG GGGAGCACGC GAAAGAGTTA
LT671859.1  TCTCGTATCT GAAATTGATA CATTAGTTGT ATCATATTAT AGTGATA--- -AGGATTATG GGGAGCACGC GAAAGAGTTA
CP007499.1  TCTCGTATCT GAAATTGATA CATTAGTTGT ATCATATTAT GGTGATA--- -AGGATTATG GGGAGCACGC GAAAGAGTTA
X17679.1    TCTCGTATCT GAAATTGATA CATTAGTTGT ATCATATTAT GGTGATA--- -AGGATTATG GGGAGCACGC GAAAGAGTTA
CP007657.1  TCTCGTATCT GAAATTGATA CATTAGTTGT ATCATATTAT GGTGATA--- -AGGATTATG GGGAGCACGC GAAAGAGTTA
CP014444.1  TCTCGTATCT GAAATTGATA CATTAGTTGT ATCATATTAT GGTGATA--- -AGGATTATG GGGAGCACGC GAAAGAGTTA
CP014441.1  TCTCGTATCT GAAATTGATA CATTAGTTGT ATCATATTAT GGTGATA--- -AGGATTATG GGGAGCACGC GAAAGAGTTA
CP014438.1  TCTCGTATCT GAAATTGATA CATTAGTTGT ATCATATTAT GGTGATA--- -AGGATTATG GGGAGCACGC GAAAGAGTTA
CP014435.1  TCTCGTATCT GAAATTGATA CATTAGTTGT ATCATATTAT GGTGATA--- -AGGATTATG GGGAGCACGC GAAAGAGTTA
CP014432.1  TCTCGTATCT GAAATTGATA CATTAGTTGT ATCATATTAT GGTGATA--- -AGGATTATG GGGAGCACGC GAAAGAGTTA
CP014429.1  TCTCGTATCT GAAATTGATA CATTAGTTGT ATCATATTAT GGTGATA--- -AGGATTATG GGGAGCACGC GAAAGAGTTA
CP014426.1  TCTCGTATCT GAAATTGATA CATTAGTTGT ATCATATTAT GGTGATA--- -AGGATTATG GGGAGCACGC GAAAGAGTTA
CP014423.1  TCTCGTATCT GAAATTGATA CATTAGTTGT ATCATATTAT GGTGATA--- -AGGATTATG GGGAGCACGC GAAAGAGTTA
CP014420.1  TCTCGTATCT GAAATTGATA CATTAGTTGT ATCATATTAT GGTGATA--- -AGGATTATG GGGAGCACGC GAAAGAGTTA
CP014415.1  TCTCGTATCT GAAATTGATA CATTAGTTGT ATCATATTAT GGTGATA--- -AGGATTATG GGGAGCACGC GAAAGAGTTA
CP014412.1  TCTCGTATCT GAAATTGATA CATTAGTTGT ATCATATTAT GGTGATA--- -AGGATTATG GGGAGCACGC GAAAGAGTTA
CP014409.1  TCTCGTATCT GAAATTGATA CATTAGTTGT ATCATATTAT GGTGATA--- -AGGATTATG GGGAGCACGC GAAAGAGTTA
CP014407.1  TCTCGTATCT GAAATTGATA CATTAGTTGT ATCATATTAT GGTGATA--- -AGGATTATG GGGAGCACGC GAAAGAGTTA
CP014402.1  TCTCGTATCT GAAATTGATA CATTAGTTGT ATCATATTAT GGTGATA--- -AGGATTATG GGGAGCACGC GAAAGAGTTA
CP014397.1  TCTCGTATCT GAAATTGATA CATTAGTTGT ATCATATTAT GGTGATA--- -AGGATTATG GGGAGCACGC GAAAGAGTTA
CP014392.1  TCTCGTATCT GAAATTGATA CATTAGTTGT ATCATATTAT GGTGATA--- -AGGATTATG GGGAGCACGC GAAAGAGTTA
CP014387.1  TCTCGTATCT GAAATTGATA CATTAGTTGT ATCATATTAT GGTGATA--- -AGGATTATG GGGAGCACGC GAAAGAGTTA
CP014384.1  TCTCGTATCT GAAATTGATA CATTAGTTGT ATCATATTAT GGTGATA--- -AGGATTATG GGGAGCACGC GAAAGAGTTA
CP014381.1  TCTCGTATCT GAAATTGATA CATTAGTTGT ATCATATTAT GGTGATA--- -AGGATTATG GGGAGCACGC GAAAGAGTTA
CP014371.1  TCTCGTATCT GAAATTGATA CATTAGTTGT ATCATATTAT GGTGATA--- -AGGATTATG GGGAGCACGC GAAAGAGTTA
CP014368.1  TCTCGTATCT GAAATTGATA CATTAGTTGT ATCATATTAT GGTGATA--- -AGGATTATG GGGAGCACGC GAAAGAGTTA
CP014365.1  TCTCGTATCT GAAATTGATA CATTAGTTGT ATCATATTAT GGTGATA--- -AGGATTATG GGGAGCACGC GAAAGAGTTA
CP014362.1  TCTCGTATCT GAAATTGATA CATTAGTTGT ATCATATTAT GGTGATA--- -AGGATTATG GGGAGCACGC GAAAGAGTTA
CP014376.1  TCTCGTATCT GAAATTGATA CATTAGTTGT ATCATATTAT GGTGATA--- -AGGATTATG GGGAGCACGC GAAAGAGTTA
CP009423.1  TCTCGTATCT GAAATTGATA CATTAGTTGT ATCATATTAT GGTGATA--- -AGGATTATG GGGAGCACGC GAAAGAGTTA
CP016855.1  TCTCGTATCT GAAATTGATA CATTAGTTGT ATCATATTAT GGTGATA--- -AGGATTATG GGGAGCACGC GAAAGAGTTA
CP013231.1  TCTCGTATCT GAAATTGATA CATTAGTTGT ATCATATTAT GGTGATA--- -AGGATTATG GGGAGCACGC GAAAGAGTTA
CP010300.1  TCTCGTATCT GAAATTGATA CATTAGTTGT ATCATATTAT GGTGATA--- -AGGATTATG GGGAGCACGC GAAAGAGTTA
CP010299.1  TCTCGTATCT GAAATTGATA CATTAGTTGT ATCATATTAT GGTGATA--- -AGGATTATG GGGAGCACGC GAAAGAGTTA
CP010298.1  TCTCGTATCT GAAATTGATA CATTAGTTGT ATCATATTAT GGTGATA--- -AGGATTATG GGGAGCACGC GAAAGAGTTA
CP010297.1  TCTCGTATCT GAAATTGATA CATTAGTTGT ATCATATTAT GGTGATA--- -AGGATTATG GGGAGCACGC GAAAGAGTTA
CP010296.1  TCTCGTATCT GAAATTGATA CATTAGTTGT ATCATATTAT GGTGATA--- -AGGATTATG GGGAGCACGC GAAAGAGTTA
CP010295.1  TCTCGTATCT GAAATTGATA CATTAGTTGT ATCATATTAT GGTGATA--- -AGGATTATG GGGAGCACGC GAAAGAGTTA
CP007690.1  TCTCGTATCT GAAATTGATA CATTAGTTGT ATCATATTAT GGTGATA--- -AGGATTATG GGGAGCACGC GAAAGAGTTA
CP007176.1  TCTCGTATCT GAAATTGATA CATTAGTTGT ATCATATTAT GGTGATA--- -AGGATTATG GGGAGCACGC GAAAGAGTTA
CP000730.1  TCTCGTATCT GAAATTGATA CATTAGTTGT ATCATATTAT GGTGATA--- -AGGATTATG GGGAGCACGC GAAAGAGTTA
CP000255.1  TCTCGTATCT GAAATTGATA CATTAGTTGT ATCATATTAT GGTGATA--- -AGGATTATG GGGAGCACGC GAAAGAGTTA
CP007539.1  TCTCGTATCT GAAATTGATA CATTAGTTGT ATCATATTAT GGTGATA--- -AGGATTATG GGGAGCACGC GAAAGAGTTA
CP007657.1  TCTCGTATCT GAAATTGATA CATTAGTTGT ATCATATTAT GGTGATA--- -AGGATTATG GGGAGCACGC GAAAGAGTTA
AP014921.1  TCTCGTATCT GAAATTGATA CATTAGTTGT ATCATATTAT GGTGATA--- -AGGATTATG GGGAGCACGC GAAAGAGTTA
AB436955.1  TCTCGTATCT GAAATTGATA CATTAGTTGT ATCATATTAT GGTGATA--- -AGGATTATG GGGAGCACGC GAAAGAGTTA
AB489885.1  TCTCGTATCT GAAATTGATA CATTAGTTGT ATCATATTAT GGTGATA--- -AGGATTATG GGGAGCACGC GAAAGAGTTA
AB489873.1  TCTCGTATCT GAAATTGATA CATTAGTTGT ATCATATTAT GGTGATA--- -AGGATTATG GGGAGCACGC GAAAGAGTTA
AB489883.1  TCTCGTATCT GAAATTGATA CATTAGTTGT ATCATATTAT GGTGATA--- -AGGATTATG GGGAGCACGC GAAAGAGTTA
AB489874.1  TCTCGTATCT GAAATTGATA CATTAGTTGT ATCATATTAT GGTGATA--- -AGGATTATG GGGAGCACGC GAAAGAGTTA
LT615218.1  TCTCGTATCT GAAATTGATA CATTAGTTGT ATCATATTAT GGTGATA--- -AGGATTATG GGGAGCACGC GAAAGAGTTA
AB436976.1  TCTCGTATCT GAAATTGATA CATTAGTTGT ATCATATTAT GGTGATA--- -AGGATTATG GGGAGCACGC GAAAGAGTTA
AB489892.1  TCTCGTATCT GAAATTGATA CATTAGTTGT ATCATATTAT GGTGATA--- -AGGATTATG GGGAGCACGC GAAAGAGTTA
AJ306908.1  TCTCGTATCT GAAATTGATA CATTAGTTGT ATCATATTAT GGTGATA--- -AGGATTATG GGGAGCACGC GAAAGAGTTA
CP015646.1  TCTCGTATCT GAAATTGATA CATTAGTTGT ATCATATTAT GGTGATA--- -AGGATTATG GGGAGCACGC GAAAGAGTTA
AB488510.1  TCTCGTATCT GAAATTGATA CATTAGTTGT ATCATATTAT GGTGATA--- -AGGATTATG GGGAGCACGC GAAAGAGTTA
EU105387.1  TCTCGTATCT GAAATTGATA CATTAGTTGT ATCATATTAT GGTGATA--- -AGGATTATG GGGAGCACGC GAAAGAGTTA
AB436975.1  TCTCGTATCT GAAATTGATA CATTAGTTGT ATCATATTAT GGTGATA--- -AGGATTATG GGGAGCACGC GAAAGAGTTA
AB488499.1  TCTCGTATCT GAAATTGATA CATTAGTTGT ATCATATTAT GGTGATA--- -AGGATTATG GGGAGCACGC GAAAGAGTTA
AJ309189.1  TCTCGTATCT GAAATTGATA CATTAGTTGT ATCATATTAT GGTGATA--- -AGGATTATG GGGAGCACGC GAAAGAGTTA
FR821779.1  CCTTGTTTCT GAAATTGATA CATTAGTTGT AACTTATTAT GGTGATA--- -AGGATTATG GGGAGCACGC GAAAGAGTTA
CP012593.1  CCTTGTTTCT GAAATTGATA CATTAGTTGT AACTTATTAT GCTGATA--- -AGGATTATG GGGAGCATGC GAAAGAGTTA
CP012692.1  CCTTGTTTCT GAAATTGATA CATTAGTTGT AACTTATTAT GCTGATA--- -AGGATTATG GGGAGCATGC GAAAGAGTTA
CP013955.1  CCTTGTTTCT GAAATTGATA CATTAGTTGT AACTTATTAT GCTGATA--- -AGGATTATG GGGAGCATGC GAAAGAGTTA
CP013953.1  CCTTGTTTCT GAAATTGATA CATTAGTTGT AACTTATTAT GCTGATA--- -AGGATTATG GGGAGCATGC GAAAGAGTTA
CP014064.1  CCTTGTTTCT GAAATTGATA CATTAGTTGT AACTTATTAT GCTGATA--- -AGGATTATG GGGAGCATGC GAAAGAGTTA
LT009690.1  CCTTGTTTCT GAAATTGATA CATTAGTTGT AACTTATTAT GCTGATA--- -AGGATTATG GGGAGCATGC GAAAGAGTTA
AP017320.1  CCTTGTTTCT GAAATTGATA CATTAGTTGT AACTTATTAT GCTGATA--- -AGGATTATG GGGAGCATGC GAAAGAGTTA
CP010890.1  CCTTGTTTCT GAAATTGATA CATTAGTTGT AACTTATTAT GCTGATA--- -AGGATTATG GGGAGCATGC GAAAGAGTTA
CP001844.2  CCTTGTTTCT GAAATTGATA CATTAGTTGT AACTTATTAT GCTGATA--- -AGGATTATG GGGAGCATGC GAAAGAGTTA
CP001781.1  CCTTGTTTCT GAAATTGATA CATTAGTTGT AACTTATTAT GCTGATA--- -AGGATTATG GGGAGCATGC GAAAGAGTTA
AB488501.1  CCTTGTTTCT GAAATTGATA CATTAGTTGT AACTTATTAT GCTGATA--- -AGGATTATG GGGAGCATGC GAAAGAGTTA
AB489898.1  CCTTGTTTCT GAAATTGATA CATTAGTTGT AACTTATTAT GCTGATA--- -AGGATTATG GGGAGCATGC GAAAGAGTTA
HE579073.1  CCTTGTTTCT GAAATTGATA CATTAGTTGT AACTTATTAT GCTGATA--- -AGGATTATG GGGAGCATGC GAAAGAGTTA
HE579071.1  CCTTGTTTCT GAAATTGATA CATTAGTTGT AACTTATTAT GCTGATA--- -AGGATTATG GGGAGCATGC GAAAGAGTTA
HE579069.1  CCTTGTTTCT GAAATTGATA CATTAGTTGT AACTTATTAT GCTGATA--- -AGGATTATG GGGAGCATGC GAAAGAGTTA
HE579065.1  CCTTGTTTCT GAAATTGATA CATTAGTTGT AACTTATTAT GCTGATA--- -AGGATTATG GGGAGCATGC GAAAGAGTTA
HE579063.1  CCTTGTTTCT GAAATTGATA CATTAGTTGT AACTTATTAT GCTGATA--- -AGGATTATG GGGAGCATGC GAAAGAGTTA
```

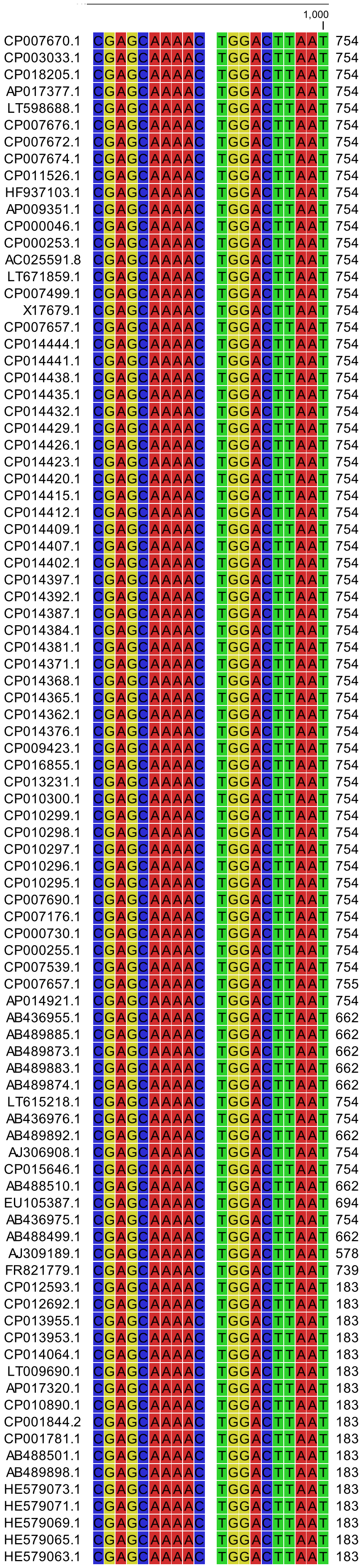

1,000
CP007670.1 CGAGCAAAAC TGGACTTAAT 754
CP003033.1 CGAGCAAAAC TGGACTTAAT 754
CP018205.1 CGAGCAAAAC TGGACTTAAT 754
AP017377.1 CGAGCAAAAC TGGACTTAAT 754
LT598688.1 CGAGCAAAAC TGGACTTAAT 754
CP007676.1 CGAGCAAAAC TGGACTTAAT 754
CP007672.1 CGAGCAAAAC TGGACTTAAT 754
CP007674.1 CGAGCAAAAC TGGACTTAAT 754
CP011526.1 CGAGCAAAAC TGGACTTAAT 754
HF937103.1 CGAGCAAAAC TGGACTTAAT 754
AP009351.1 CGAGCAAAAC TGGACTTAAT 754
CP000046.1 CGAGCAAAAC TGGACTTAAT 754
CP000253.1 CGAGCAAAAC TGGACTTAAT 754
AC025591.8 CGAGCAAAAC TGGACTTAAT 754
LT671859.1 CGAGCAAAAC TGGACTTAAT 754
CP007499.1 CGAGCAAAAC TGGACTTAAT 754
X17679.1 CGAGCAAAAC TGGACTTAAT 754
CP007657.1 CGAGCAAAAC TGGACTTAAT 754
CP014444.1 CGAGCAAAAC TGGACTTAAT 754
CP014441.1 CGAGCAAAAC TGGACTTAAT 754
CP014438.1 CGAGCAAAAC TGGACTTAAT 754
CP014435.1 CGAGCAAAAC TGGACTTAAT 754
CP014432.1 CGAGCAAAAC TGGACTTAAT 754
CP014429.1 CGAGCAAAAC TGGACTTAAT 754
CP014426.1 CGAGCAAAAC TGGACTTAAT 754
CP014423.1 CGAGCAAAAC TGGACTTAAT 754
CP014420.1 CGAGCAAAAC TGGACTTAAT 754
CP014415.1 CGAGCAAAAC TGGACTTAAT 754
CP014412.1 CGAGCAAAAC TGGACTTAAT 754
CP014409.1 CGAGCAAAAC TGGACTTAAT 754
CP014407.1 CGAGCAAAAC TGGACTTAAT 754
CP014402.1 CGAGCAAAAC TGGACTTAAT 754
CP014397.1 CGAGCAAAAC TGGACTTAAT 754
CP014392.1 CGAGCAAAAC TGGACTTAAT 754
CP014387.1 CGAGCAAAAC TGGACTTAAT 754
CP014384.1 CGAGCAAAAC TGGACTTAAT 754
CP014381.1 CGAGCAAAAC TGGACTTAAT 754
CP014371.1 CGAGCAAAAC TGGACTTAAT 754
CP014368.1 CGAGCAAAAC TGGACTTAAT 754
CP014365.1 CGAGCAAAAC TGGACTTAAT 754
CP014362.1 CGAGCAAAAC TGGACTTAAT 754
CP014376.1 CGAGCAAAAC TGGACTTAAT 754
CP009423.1 CGAGCAAAAC TGGACTTAAT 754
CP016855.1 CGAGCAAAAC TGGACTTAAT 754
CP013231.1 CGAGCAAAAC TGGACTTAAT 754
CP010300.1 CGAGCAAAAC TGGACTTAAT 754
CP010299.1 CGAGCAAAAC TGGACTTAAT 754
CP010298.1 CGAGCAAAAC TGGACTTAAT 754
CP010297.1 CGAGCAAAAC TGGACTTAAT 754
CP010296.1 CGAGCAAAAC TGGACTTAAT 754
CP010295.1 CGAGCAAAAC TGGACTTAAT 754
CP007690.1 CGAGCAAAAC TGGACTTAAT 754
CP007176.1 CGAGCAAAAC TGGACTTAAT 754
CP000730.1 CGAGCAAAAC TGGACTTAAT 754
CP000255.1 CGAGCAAAAC TGGACTTAAT 754
CP007539.1 CGAGCAAAAC TGGACTTAAT 754
CP007657.1 CGAGCAAAAC TGGACTTAAT 755
AP014921.1 CGAGCAAAAC TGGACTTAAT 754
AB436955.1 CGAGCAAAAC TGGACTTAAT 662
AB489885.1 CGAGCAAAAC TGGACTTAAT 662
AB489873.1 CGAGCAAAAC TGGACTTAAT 662
AB489883.1 CGAGCAAAAC TGGACTTAAT 662
AB489874.1 CGAGCAAAAC TGGACTTAAT 662
LT615218.1 CGAGCAAAAC TGGACTTAAT 754
AB436976.1 CGAGCAAAAC TGGACTTAAT 754
AB489892.1 CGAGCAAAAC TGGACTTAAT 662
AJ306908.1 CGAGCAAAAC TGGACTTAAT 754
CP015646.1 CGAGCAAAAC TGGACTTAAT 754
AB488510.1 CGAGCAAAAC TGGACTTAAT 662
EU105387.1 CGAGCAAAAC TGGACTTAAT 694
AB436975.1 CGAGCAAAAC TGGACTTAAT 754
AB488499.1 CGAGCAAAAC TGGACTTAAT 662
AJ309189.1 CGAGCAAAAC TGGACTTAAT 578
FR821779.1 CGAGCAAAAC TGGACTTAAT 739
CP012593.1 CGAGCAAAAC TGGACTTAAT 183
CP012692.1 CGAGCAAAAC TGGACTTAAT 183
CP013955.1 CGAGCAAAAC TGGACTTAAT 183
CP013953.1 CGAGCAAAAC TGGACTTAAT 183
CP014064.1 CGAGCAAAAC TGGACTTAAT 183
LT009690.1 CGAGCAAAAC TGGACTTAAT 183
AP017320.1 CGAGCAAAAC TGGACTTAAT 183
CP010890.1 CGAGCAAAAC TGGACTTAAT 183
CP001844.2 CGAGCAAAAC TGGACTTAAT 183
CP001781.1 CGAGCAAAAC TGGACTTAAT 183
AB488501.1 CGAGCAAAAC TGGACTTAAT 183
AB489898.1 CGAGCAAAAC TGGACTTAAT 183
HE579073.1 CGAGCAAAAC TGGACTTAAT 183
HE579071.1 CGAGCAAAAC TGGACTTAAT 183
HE579069.1 CGAGCAAAAC TGGACTTAAT 183
HE579065.1 CGAGCAAAAC TGGACTTAAT 183
HE579063.1 CGAGCAAAAC TGGACTTAAT 183

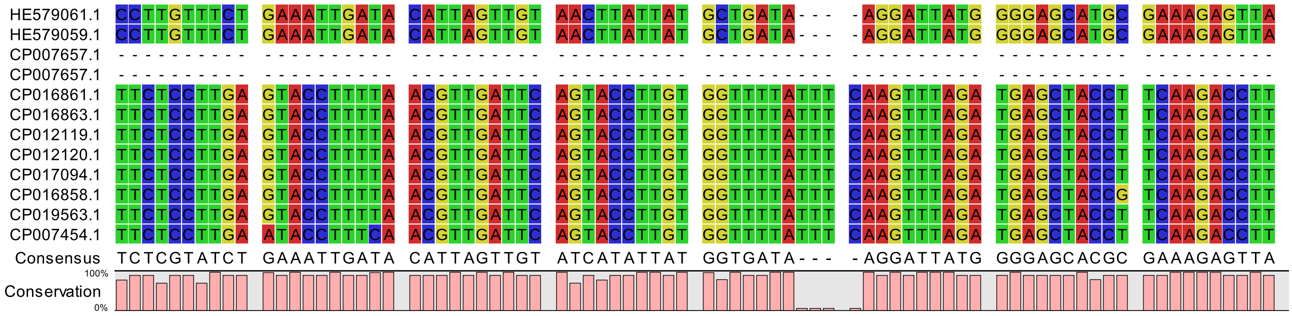
HE579061.1 CCTTGTTTCT GAAATTGATA CATTAGTTGT AACTTATTAT GCTGATA--- -AGGATTATG GGGAGCATGC GAAAGAGTTA
HE579059.1 CCTTGTTTCT GAAATTGATA CATTAGTTGT AACTTATTAT GCTGATA--- -AGGATTATG GGGAGCATGC GAAAGAGTTA
CP007657.1 ---------- ---------- ---------- ---------- ---------- ---------- ---------- ----------
CP007657.1 ---------- ---------- ---------- ---------- ---------- ---------- ---------- ----------
CP016861.1 TTCTCCTTGA GTACCTTTTA ACGTTGATTC AGTACCTTGT GGTTTTATTT CAAGTTTAGA TGAGCTACCT TCAAGACCTT
CP016863.1 TTCTCCTTGA GTACCTTTTA ACGTTGATTC AGTACCTTGT GGTTTTATTT CAAGTTTAGA TGAGCTACCT TCAAGACCTT
CP012119.1 TTCTCCTTGA GTACCTTTTA ACGTTGATTC AGTACCTTGT GGTTTTATTT CAAGTTTAGA TGAGCTACCT TCAAGACCTT
CP012120.1 TTCTCCTTGA GTACCTTTTA ACGTTGATTC AGTACCTTGT GGTTTTATTT CAAGTTTAGA TGAGCTACCT TCAAGACCTT
CP017094.1 TTCTCCTTGA GTACCTTTTA ACGTTGATTC AGTACCTTGT GGTTTTATTT CAAGTTTAGA TGAGCTACCT TCAAGACCTT
CP016858.1 TTCTCCTTGA GTACCTTTTA ACGTTGATTC AGTACCTTGT GGTTTTATTT CAAGTTTAGA TGAGCTACCG TCAAGACCTT
CP019563.1 TTCTCCTTGA GTACCTTTTA ACGTTGATTC AGTACCTTGT GGTTTTATTT CAAGTTTAGA TGAGCTACCT TCAAGACCTT
CP007454.1 TTCTCCTTGA ATACCTTTCA ACGTTGATTC AGTACCTTGT GGTTTTATTT CAAGTTTAGA TGAGCTACCT TCAAGACCTT
Consensus TCTCGTATCT GAAATTGATA CATTAGTTGT ATCATATTAT GGTGATA--- -AGGATTATG GGGAGCACGC GAAAGAGTTA
100%
Conservation
0%

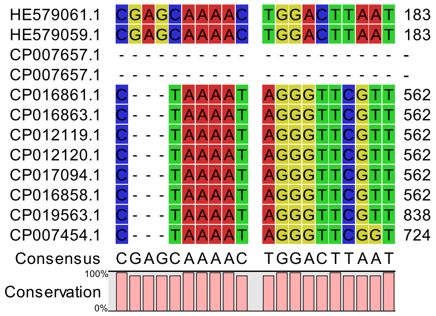

HE579061.1 CGAGCAAAAC TGGACTTAAT 183
HE579059.1 CGAGCAAAAC TGGACTTAAT 183
CP007657.1 -
CP007657.1 -
CP016861.1 C - - - TAAAAT AGGGTTCGTT 562
CP016863.1 C - - - TAAAAT AGGGTTCGTT 562
CP012119.1 C - - - TAAAAT AGGGTTCGTT 562
CP012120.1 C - - - TAAAAT AGGGTTCGTT 562
CP017094.1 C - - - TAAAAT AGGGTTCGTT 562
CP016858.1 C - - - TAAAAT AGGGTTCGTT 562
CP019563.1 C - - - TAAAAT AGGGTTCGTT 838
CP007454.1 C - - - TAAAAT AGGGTTCGGT 724
Consensus CGAGCAAAAC TGGACTTAAT
100%
Conservation
0%

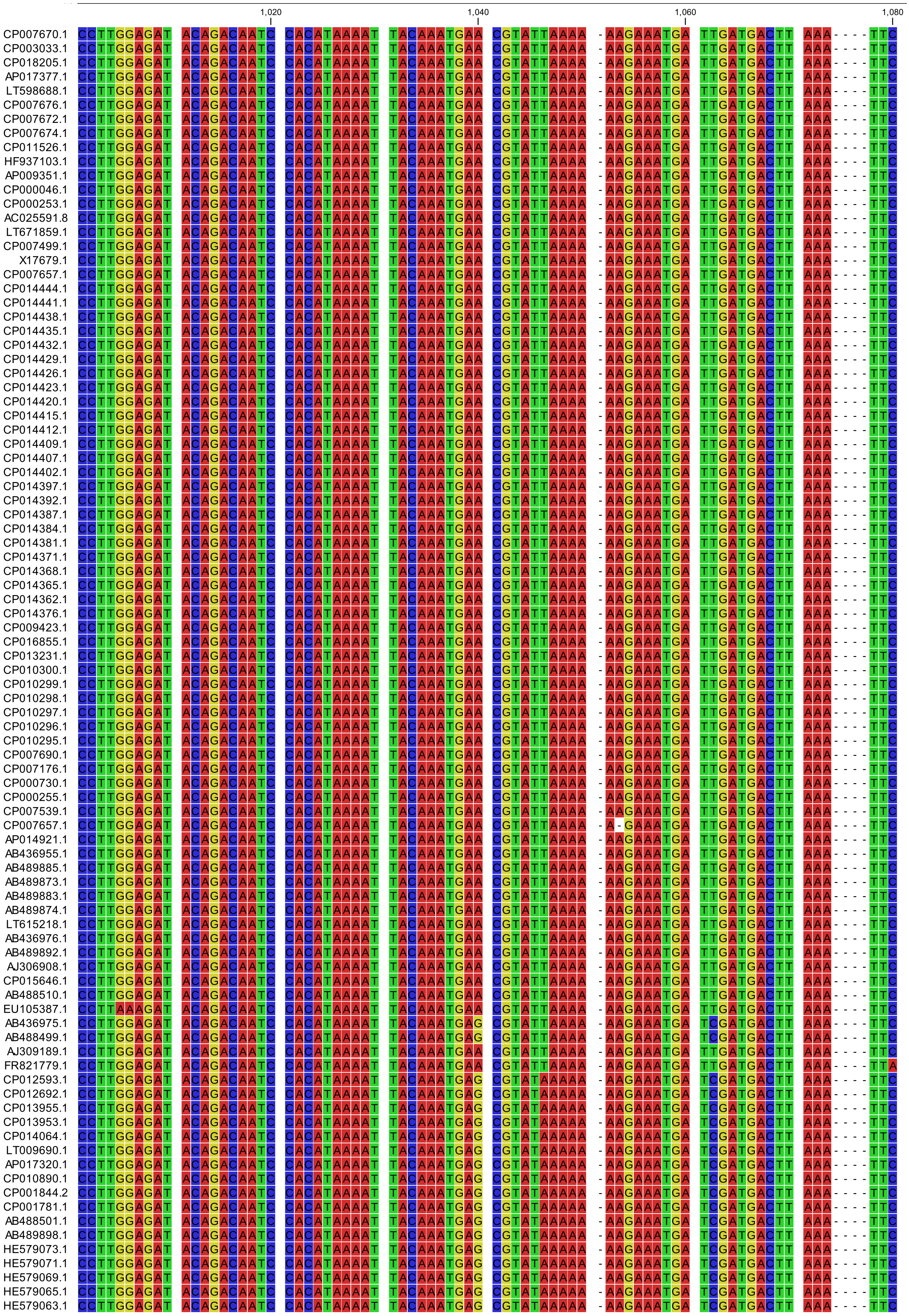

1,020
1,040
1,060
1,080
CP007670.1 CCTTGGAGAT ACAGACAATC CACATAAAAT TACAAATGAA CGTATTAAAA -AAGAAATGA TTGATGACTT AAA----TTC
CP003033.1 CCTTGGAGAT ACAGACAATC CACATAAAAT TACAAATGAA CGTATTAAAA -AAGAAATGA TTGATGACTT AAA----TTC
CP018205.1 CCTTGGAGAT ACAGACAATC CACATAAAAT TACAAATGAA CGTATTAAAA -AAGAAATGA TTGATGACTT AAA----TTC
AP017377.1 CCTTGGAGAT ACAGACAATC CACATAAAAT TACAAATGAA CGTATTAAAA -AAGAAATGA TTGATGACTT AAA----TTC
LT598688.1 CCTTGGAGAT ACAGACAATC CACATAAAAT TACAAATGAA CGTATTAAAA -AAGAAATGA TTGATGACTT AAA----TTC
CP007676.1 CCTTGGAGAT ACAGACAATC CACATAAAAT TACAAATGAA CGTATTAAAA -AAGAAATGA TTGATGACTT AAA----TTC
CP007672.1 CCTTGGAGAT ACAGACAATC CACATAAAAT TACAAATGAA CGTATTAAAA -AAGAAATGA TTGATGACTT AAA----TTC
CP007674.1 CCTTGGAGAT ACAGACAATC CACATAAAAT TACAAATGAA CGTATTAAAA -AAGAAATGA TTGATGACTT AAA----TTC
CP011526.1 CCTTGGAGAT ACAGACAATC CACATAAAAT TACAAATGAA CGTATTAAAA -AAGAAATGA TTGATGACTT AAA----TTC
HF937103.1 CCTTGGAGAT ACAGACAATC CACATAAAAT TACAAATGAA CGTATTAAAA -AAGAAATGA TTGATGACTT AAA----TTC
AP009351.1 CCTTGGAGAT ACAGACAATC CACATAAAAT TACAAATGAA CGTATTAAAA -AAGAAATGA TTGATGACTT AAA----TTC
CP000046.1 CCTTGGAGAT ACAGACAATC CACATAAAAT TACAAATGAA CGTATTAAAA -AAGAAATGA TTGATGACTT AAA----TTC
CP000253.1 CCTTGGAGAT ACAGACAATC CACATAAAAT TACAAATGAA CGTATTAAAA -AAGAAATGA TTGATGACTT AAA----TTC
AC025591.8 CCTTGGAGAT ACAGACAATC CACATAAAAT TACAAATGAA CGTATTAAAA -AAGAAATGA TTGATGACTT AAA----TTC
LT671859.1 CCTTGGAGAT ACAGACAATC CACATAAAAT TACAAATGAA CGTATTAAAA -AAGAAATGA TTGATGACTT AAA----TTC
CP007499.1 CCTTGGAGAT ACAGACAATC CACATAAAAT TACAAATGAA CGTATTAAAA -AAGAAATGA TTGATGACTT AAA----TTC
X17679.1 CCTTGGAGAT ACAGACAATC CACATAAAAT TACAAATGAA CGTATTAAAA -AAGAAATGA TTGATGACTT AAA----TTC
CP007657.1 CCTTGGAGAT ACAGACAATC CACATAAAAT TACAAATGAA CGTATTAAAA -AAGAAATGA TTGATGACTT AAA----TTC
CP014444.1 CCTTGGAGAT ACAGACAATC CACATAAAAT TACAAATGAA CGTATTAAAA -AAGAAATGA TTGATGACTT AAA----TTC
CP014441.1 CCTTGGAGAT ACAGACAATC CACATAAAAT TACAAATGAA CGTATTAAAA -AAGAAATGA TTGATGACTT AAA----TTC
CP014438.1 CCTTGGAGAT ACAGACAATC CACATAAAAT TACAAATGAA CGTATTAAAA -AAGAAATGA TTGATGACTT AAA----TTC
CP014435.1 CCTTGGAGAT ACAGACAATC CACATAAAAT TACAAATGAA CGTATTAAAA -AAGAAATGA TTGATGACTT AAA----TTC
CP014432.1 CCTTGGAGAT ACAGACAATC CACATAAAAT TACAAATGAA CGTATTAAAA -AAGAAATGA TTGATGACTT AAA----TTC
CP014429.1 CCTTGGAGAT ACAGACAATC CACATAAAAT TACAAATGAA CGTATTAAAA -AAGAAATGA TTGATGACTT AAA----TTC
CP014426.1 CCTTGGAGAT ACAGACAATC CACATAAAAT TACAAATGAA CGTATTAAAA -AAGAAATGA TTGATGACTT AAA----TTC
CP014423.1 CCTTGGAGAT ACAGACAATC CACATAAAAT TACAAATGAA CGTATTAAAA -AAGAAATGA TTGATGACTT AAA----TTC
CP014420.1 CCTTGGAGAT ACAGACAATC CACATAAAAT TACAAATGAA CGTATTAAAA -AAGAAATGA TTGATGACTT AAA----TTC
CP014415.1 CCTTGGAGAT ACAGACAATC CACATAAAAT TACAAATGAA CGTATTAAAA -AAGAAATGA TTGATGACTT AAA----TTC
CP014412.1 CCTTGGAGAT ACAGACAATC CACATAAAAT TACAAATGAA CGTATTAAAA -AAGAAATGA TTGATGACTT AAA----TTC
CP014409.1 CCTTGGAGAT ACAGACAATC CACATAAAAT TACAAATGAA CGTATTAAAA -AAGAAATGA TTGATGACTT AAA----TTC
CP014407.1 CCTTGGAGAT ACAGACAATC CACATAAAAT TACAAATGAA CGTATTAAAA -AAGAAATGA TTGATGACTT AAA----TTC
CP014402.1 CCTTGGAGAT ACAGACAATC CACATAAAAT TACAAATGAA CGTATTAAAA -AAGAAATGA TTGATGACTT AAA----TTC
CP014397.1 CCTTGGAGAT ACAGACAATC CACATAAAAT TACAAATGAA CGTATTAAAA -AAGAAATGA TTGATGACTT AAA----TTC
CP014392.1 CCTTGGAGAT ACAGACAATC CACATAAAAT TACAAATGAA CGTATTAAAA -AAGAAATGA TTGATGACTT AAA----TTC
CP014387.1 CCTTGGAGAT ACAGACAATC CACATAAAAT TACAAATGAA CGTATTAAAA -AAGAAATGA TTGATGACTT AAA----TTC
CP014384.1 CCTTGGAGAT ACAGACAATC CACATAAAAT TACAAATGAA CGTATTAAAA -AAGAAATGA TTGATGACTT AAA----TTC
CP014381.1 CCTTGGAGAT ACAGACAATC CACATAAAAT TACAAATGAA CGTATTAAAA -AAGAAATGA TTGATGACTT AAA----TTC
CP014371.1 CCTTGGAGAT ACAGACAATC CACATAAAAT TACAAATGAA CGTATTAAAA -AAGAAATGA TTGATGACTT AAA----TTC
CP014368.1 CCTTGGAGAT ACAGACAATC CACATAAAAT TACAAATGAA CGTATTAAAA -AAGAAATGA TTGATGACTT AAA----TTC
CP014365.1 CCTTGGAGAT ACAGACAATC CACATAAAAT TACAAATGAA CGTATTAAAA -AAGAAATGA TTGATGACTT AAA----TTC
CP014362.1 CCTTGGAGAT ACAGACAATC CACATAAAAT TACAAATGAA CGTATTAAAA -AAGAAATGA TTGATGACTT AAA----TTC
CP014376.1 CCTTGGAGAT ACAGACAATC CACATAAAAT TACAAATGAA CGTATTAAAA -AAGAAATGA TTGATGACTT AAA----TTC
CP009423.1 CCTTGGAGAT ACAGACAATC CACATAAAAT TACAAATGAA CGTATTAAAA -AAGAAATGA TTGATGACTT AAA----TTC
CP016855.1 CCTTGGAGAT ACAGACAATC CACATAAAAT TACAAATGAA CGTATTAAAA -AAGAAATGA TTGATGACTT AAA----TTC
CP013231.1 CCTTGGAGAT ACAGACAATC CACATAAAAT TACAAATGAA CGTATTAAAA -AAGAAATGA TTGATGACTT AAA----TTC
CP010300.1 CCTTGGAGAT ACAGACAATC CACATAAAAT TACAAATGAA CGTATTAAAA -AAGAAATGA TTGATGACTT AAA----TTC
CP010299.1 CCTTGGAGAT ACAGACAATC CACATAAAAT TACAAATGAA CGTATTAAAA -AAGAAATGA TTGATGACTT AAA----TTC
CP010298.1 CCTTGGAGAT ACAGACAATC CACATAAAAT TACAAATGAA CGTATTAAAA -AAGAAATGA TTGATGACTT AAA----TTC
CP010297.1 CCTTGGAGAT ACAGACAATC CACATAAAAT TACAAATGAA CGTATTAAAA -AAGAAATGA TTGATGACTT AAA----TTC
CP010296.1 CCTTGGAGAT ACAGACAATC CACATAAAAT TACAAATGAA CGTATTAAAA -AAGAAATGA TTGATGACTT AAA----TTC
CP010295.1 CCTTGGAGAT ACAGACAATC CACATAAAAT TACAAATGAA CGTATTAAAA -AAGAAATGA TTGATGACTT AAA----TTC
CP007690.1 CCTTGGAGAT ACAGACAATC CACATAAAAT TACAAATGAA CGTATTAAAA -AAGAAATGA TTGATGACTT AAA----TTC
CP007176.1 CCTTGGAGAT ACAGACAATC CACATAAAAT TACAAATGAA CGTATTAAAA -AAGAAATGA TTGATGACTT AAA----TTC
CP000730.1 CCTTGGAGAT ACAGACAATC CACATAAAAT TACAAATGAA CGTATTAAAA -AAGAAATGA TTGATGACTT AAA----TTC
CP000255.1 CCTTGGAGAT ACAGACAATC CACATAAAAT TACAAATGAA CGTATTAAAA -AAGAAATGA TTGATGACTT AAA----TTC
CP007539.1 CCTTGGAGAT ACAGACAATC CACATAAAAT TACAAATGAA CGTATTAAAA -AAGAAATGA TTGATGACTT AAA----TTC
CP007657.1 CCTTGGAGAT ACAGACAATC CACATAAAAT TACAAATGAA CGTATTAAAA -A-GAAATGA TTGATGACTT AAA----TTC
AP014921.1 CCTTGGAGAT ACAGACAATC CACATAAAAT TACAAATGAA CGTATTAAAA -AAGAAATGA TTGATGACTT AAA----TTC
AB436955.1 CCTTGGAGAT ACAGACAATC CACATAAAAT TACAAATGAA CGTATTAAAA -AAGAAATGA TTGATGACTT AAA----TTC
AB489885.1 CCTTGGAGAT ACAGACAATC CACATAAAAT TACAAATGAA CGTATTAAAA -AAGAAATGA TTGATGACTT AAA----TTC
AB489873.1 CCTTGGAGAT ACAGACAATC CACATAAAAT TACAAATGAA CGTATTAAAA -AAGAAATGA TTGATGACTT AAA----TTC
AB489883.1 CCTTGGAGAT ACAGACAATC CACATAAAAT TACAAATGAA CGTATTAAAA -AAGAAATGA TTGATGACTT AAA----TTC
AB489874.1 CCTTGGAGAT ACAGACAATC CACATAAAAT TACAAATGAA CGTATTAAAA -AAGAAATGA TTGATGACTT AAA----TTC
LT615218.1 CCTTGGAGAT ACAGACAATC CACATAAAAT TACAAATGAA CGTATTAAAA -AAGAAATGA TTGATGACTT AAA----TTC
AB436976.1 CCTTGGAGAT ACAGACAATC CACATAAAAT TACAAATGAA CGTATTAAAA -AAGAAATGA TTGATGACTT AAA----TTC
AB489892.1 CCTTGGAGAT ACAGACAATC CACATAAAAT TACAAATGAA CGTATTAAAA -AAGAAATGA TTGATGACTT AAA----TTC
AJ306908.1 CCTTGGAGAT ACAGACAATC CACATAAAAT TACAAATGAA CGTATTAAAA -AAGAAATGA TTGATGACTT AAA----TTC
CP015646.1 CCTTGGAGAT ACAGACAATC CACATAAAAT TACAAATGAA CGTATTAAAA -AAGAAATGA TTGATGACTT AAA----TTC
AB488510.1 CCTTGGAGAT ACAGACAATC CACATAAAAT TACAAATGAA CGTATTAAAA -AAGAAATGA TTGATGACTT AAA----TTC
EU105387.1 CCTTAAAGAT ACAGACAATC CACATAAAAT TACAAATGAA CGTATTAAAA -AAGAAATGA TTGATGACTT AAA----TTC
AB436975.1 CCTTGGAGAT ACAGACAATC CACATAAAAT TACAAATGAG CGTATTAAAA -AAGAAATGA TCGATGACTT AAA----TTC
AB488499.1 CCTTGGAGAT ACAGACAATC CACATAAAAT TACAAATGAG CGTATTAAAA -AAGAAATGA TCGATGACTT AAA----TTC
AJ309189.1 CCTTGGAGAT ACAGACAATC CACATAAAAT TACAAATGAA CGTATTAAAA -AAGAAATGA TTGATGACTT AAA----TTC
FR821779.1 CCTTGGAGAT ACAGACAATC CACATAAAAT TACAAATGAA CGTATTAAAA -AAGAAATGA TTGATGACTT AAA----TTA
CP012593.1 CCTTGGAGAT ACAGACAATC CACATAAAAT TACAAATGAG CGTATAAAAA -AAGAAATGA TCGATGACTT AAA----TTC
CP012692.1 CCTTGGAGAT ACAGACAATC CACATAAAAT TACAAATGAG CGTATAAAAA -AAGAAATGA TCGATGACTT AAA----TTC
CP013955.1 CCTTGGAGAT ACAGACAATC CACATAAAAT TACAAATGAG CGTATAAAAA -AAGAAATGA TCGATGACTT AAA----TTC
CP013953.1 CCTTGGAGAT ACAGACAATC CACATAAAAT TACAAATGAG CGTATAAAAA -AAGAAATGA TCGATGACTT AAA----TTC
CP014064.1 CCTTGGAGAT ACAGACAATC CACATAAAAT TACAAATGAG CGTATAAAAA -AAGAAATGA TCGATGACTT AAA----TTC
LT009690.1 CCTTGGAGAT ACAGACAATC CACATAAAAT TACAAATGAG CGTATAAAAA -AAGAAATGA TCGATGACTT AAA----TTC
AP017320.1 CCTTGGAGAT ACAGACAATC CACATAAAAT TACAAATGAG CGTATAAAAA -AAGAAATGA TCGATGACTT AAA----TTC
CP010890.1 CCTTGGAGAT ACAGACAATC CACATAAAAT TACAAATGAG CGTATAAAAA -AAGAAATGA TCGATGACTT AAA----TTC
CP001844.2 CCTTGGAGAT ACAGACAATC CACATAAAAT TACAAATGAG CGTATAAAAA -AAGAAATGA TCGATGACTT AAA----TTC
CP001781.1 CCTTGGAGAT ACAGACAATC CACATAAAAT TACAAATGAG CGTATAAAAA -AAGAAATGA TCGATGACTT AAA----TTC
AB488501.1 CCTTGGAGAT ACAGACAATC CACATAAAAT TACAAATGAG CGTATAAAAA -AAGAAATGA TCGATGACTT AAA----TTC
AB489898.1 CCTTGGAGAT ACAGACAATC CACATAAAAT TACAAATGAG CGTATAAAAA -AAGAAATGA TCGATGACTT AAA----TTC
HE579073.1 CCTTGGAGAT ACAGACAATC CACATAAAAT TACAAATGAG CGTATAAAAA -AAGAAATGA TCGATGACTT AAA----TTC
HE579071.1 CCTTGGAGAT ACAGACAATC CACATAAAAT TACAAATGAG CGTATAAAAA -AAGAAATGA TCGATGACTT AAA----TTC
HE579069.1 CCTTGGAGAT ACAGACAATC CACATAAAAT TACAAATGAG CGTATAAAAA -AAGAAATGA TCGATGACTT AAA----TTC
HE579065.1 CCTTGGAGAT ACAGACAATC CACATAAAAT TACAAATGAG CGTATAAAAA -AAGAAATGA TCGATGACTT AAA----TTC
HE579063.1 CCTTGGAGAT ACAGACAATC CACATAAAAT TACAAATGAG CGTATAAAAA -AAGAAATGA TCGATGACTT AAA----TTC

1,100

CP007670.1	AATTATTGAT	GATTTCTTTA	849
CP003033.1	AATTATTGAT	GATTTCTTTA	849
CP018205.1	AATTATTGAT	GATTTCTTTA	849
AP017377.1	AATTATTGAT	GATTTCTTTA	849
LT598688.1	AATTATTGAT	GATTTCTTTA	849
CP007676.1	AATTATTGAT	GATTTCTTTA	849
CP007672.1	AATTATTGAT	GATTTCTTTA	849
CP007674.1	AATTATTGAT	GATTTCTTTA	849
CP011526.1	AATTATTGAT	GATTTCTTTA	849
HF937103.1	AATTATTGAT	GATTTCTTTA	849
AP009351.1	AATTATTGAT	GATTTCTTTA	849
CP000046.1	AATTATTGAT	GATTTCTTTA	849
CP000253.1	AATTATTGAT	GATTTCTTTA	849
AC025591.8	AATTATTGAT	GATTTCTTTA	849
LT671859.1	AATTATTGAT	GATTTCTTTA	849
CP007499.1	AATTATTGAT	GATTTCTTTA	849
X17679.1	AATTATTGAT	GATTTCTTTA	849
CP007657.1	AATTATTGAT	GATTTCTTTA	849
CP014444.1	AATTATTGAT	GATTTCTTTA	849
CP014441.1	AATTATTGAT	GATTTCTTTA	849
CP014438.1	AATTATTGAT	GATTTCTTTA	849
CP014435.1	AATTATTGAT	GATTTCTTTA	849
CP014432.1	AATTATTGAT	GATTTCTTTA	849
CP014429.1	AATTATTGAT	GATTTCTTTA	849
CP014426.1	AATTATTGAT	GATTTCTTTA	849
CP014423.1	AATTATTGAT	GATTTCTTTA	849
CP014420.1	AATTATTGAT	GATTTCTTTA	849
CP014415.1	AATTATTGAT	GATTTCTTTA	849
CP014412.1	AATTATTGAT	GATTTCTTTA	849
CP014409.1	AATTATTGAT	GATTTCTTTA	849
CP014407.1	AATTATTGAT	GATTTCTTTA	849
CP014402.1	AATTATTGAT	GATTTCTTTA	849
CP014397.1	AATTATTGAT	GATTTCTTTA	849
CP014392.1	AATTATTGAT	GATTTCTTTA	849
CP014387.1	AATTATTGAT	GATTTCTTTA	849
CP014384.1	AATTATTGAT	GATTTCTTTA	849
CP014381.1	AATTATTGAT	GATTTCTTTA	849
CP014371.1	AATTATTGAT	GATTTCTTTA	849
CP014368.1	AATTATTGAT	GATTTCTTTA	849
CP014365.1	AATTATTGAT	GATTTCTTTA	849
CP014362.1	AATTATTGAT	GATTTCTTTA	849
CP014376.1	AATTATTGAT	GATTTCTTTA	849
CP009423.1	AATTATTGAT	GATTTCTTTA	849
CP016855.1	AATTATTGAT	GATTTCTTTA	849
CP013231.1	AATTATTGAT	GATTTCTTTA	849
CP010300.1	AATTATTGAT	GATTTCTTTA	849
CP010299.1	AATTATTGAT	GATTTCTTTA	849
CP010298.1	AATTATTGAT	GATTTCTTTA	849
CP010297.1	AATTATTGAT	GATTTCTTTA	849
CP010296.1	AATTATTGAT	GATTTCTTTA	849
CP010295.1	AATTATTGAT	GATTTCTTTA	849
CP007690.1	AATTATTGAT	GATTTCTTTA	849
CP007176.1	AATTATTGAT	GATTTCTTTA	849
CP000730.1	AATTATTGAT	GATTTCTTTA	849
CP000255.1	AATTATTGAT	GATTTCTTTA	849
CP007539.1	AATTATTGAT	GATTTCTTTA	849
CP007657.1	AATTATTGAT	GATTTCTTTA	849
AP014921.1	AATTATTGAT	GATTTCTTTA	849
AB436955.1	AATTATTGAT	GATTTCTTTA	757
AB489885.1	AATTATTGAT	GATTTCTTTA	757
AB489873.1	AATTATTGAT	GATTTCTTTA	757
AB489883.1	AATTATTGAT	GATTTCTTTA	757
AB489874.1	AATTATTGAT	GATTTCTTTA	757
LT615218.1	AATTATTGAT	GATTTCTTTA	849
AB436976.1	AATTATTGAT	GATTTCTTTA	849
AB489892.1	AATTATTGAT	GATTTCTTTA	757
AJ306908.1	AATTATTGAT	GATTTCTTTA	849
CP015646.1	AATTATTGAT	GATTTCTTTA	849
AB488510.1	AATTATTGAT	GATTTCTTTA	757
EU105387.1	AATTATTGAT	GATTTCTTTA	789
AB436975.1	AATTATTGAT	GATTTCTTTA	849
AB488499.1	AATTATTGAT	GATTTCTTTA	757
AJ309189.1	AATTATTGAT	GATTTCTTTA	673
FR821779.1	AATTATTGAT	GATTTCTTTA	834
CP012593.1	AATTATAGAT	GATTTCTTTA	278
CP012692.1	AATTATAGAT	GATTTCTTTA	278
CP013955.1	AATTATAGAT	GATTTCTTTA	278
CP013953.1	AATTATAGAT	GATTTCTTTA	278
CP014064.1	AATTATAGAT	GATTTCTTTA	278
LT009690.1	AATTATAGAT	GATTTCTTTA	278
AP017320.1	AATTATAGAT	GATTTCTTTA	278
CP010890.1	AATTATAGAT	GATTTCTTTA	278
CP001844.2	AATTATAGAT	GATTTCTTTA	278
CP001781.1	AATTATAGAT	GATTTCTTTA	278
AB488501.1	AATTATAGAT	GATTTCTTTA	278
AB489898.1	AATTATAGAT	GATTTCTTTA	278
HE579073.1	AATTATAGAT	GATTTCTTTA	278
HE579071.1	AATTATAGAT	GATTTCTTTA	278
HE579069.1	AATTATAGAT	GATTTCTTTA	278
HE579065.1	AATTATAGAT	GATTTCTTTA	278
HE579063.1	AATTATAGAT	GATTTCTTTA	278

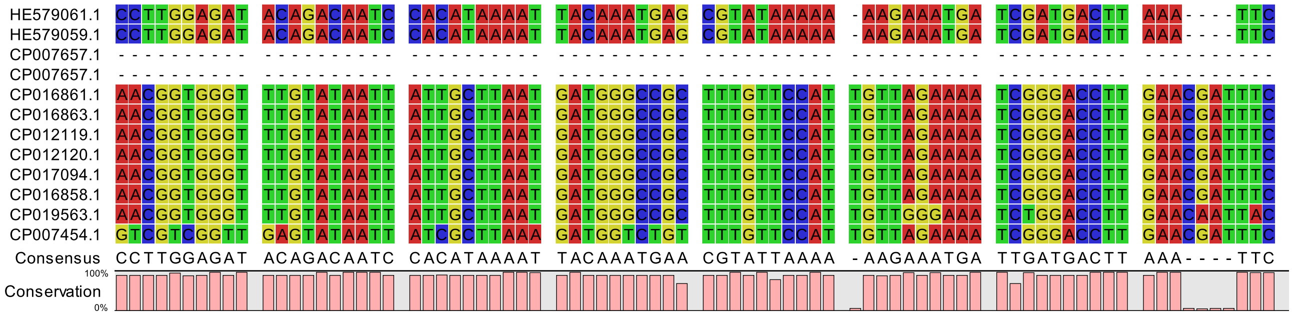
HE579061.1 CCTTGGAGAT ACAGACAATC CACATAAAAT TACAAATGAG CGTATAAAAA -AAGAAATGA TCGATGACTT AAA----TTC
HE579059.1 CCTTGGAGAT ACAGACAATC CACATAAAAT TACAAATGAG CGTATAAAAA -AAGAAATGA TCGATGACTT AAA----TTC
CP007657.1 ---------- ---------- ---------- ---------- ---------- ---------- ---------- ----------
CP007657.1 ---------- ---------- ---------- ---------- ---------- ---------- ---------- ----------
CP016861.1 AACGGTGGGT TTGTATAATT ATTGCTTAAT GATGGGCCGC TTTGTTCCAT TGTTAGAAAA TCGGGACCTT GAACGATTTC
CP016863.1 AACGGTGGGT TTGTATAATT ATTGCTTAAT GATGGGCCGC TTTGTTCCAT TGTTAGAAAA TCGGGACCTT GAACGATTTC
CP012119.1 AACGGTGGGT TTGTATAATT ATTGCTTAAT GATGGGCCGC TTTGTTCCAT TGTTAGAAAA TCGGGACCTT GAACGATTTC
CP012120.1 AACGGTGGGT TTGTATAATT ATTGCTTAAT GATGGGCCGC TTTGTTCCAT TGTTAGAAAA TCGGGACCTT GAACGATTTC
CP017094.1 AACGGTGGGT TTGTATAATT ATTGCTTAAT GATGGGCCGC TTTGTTCCAT TGTTAGAAAA TCGGGACCTT GAACGATTTC
CP016858.1 AACGGTGGGT TTGTATAATT ATTGCTTAAT GATGGGCCGC TTTGTTCCAT TGTTAGAAAA TCGGGACCTT GAACGATTTC
CP019563.1 AACGGTGGGT TTGTATAATT ATTGCTTAAT GATGGGCCGC TTTGTTCCAT TGTTGGGAAA TCTGGACCTT GAACAATTAC
CP007454.1 GTCGTCGGTT GAGTATAATT ATCGCTTAAA GATGGTCTGT TTTGTTCCAT TGTTAGAAAA TCGGGACCTT GAACGATTTC
Consensus CCTTGGAGAT ACAGACAATC CACATAAAAT TACAAATGAA CGTATTAAAA -AAGAAATGA TTGATGACTT AAA----TTC
100%
Conservation
0%

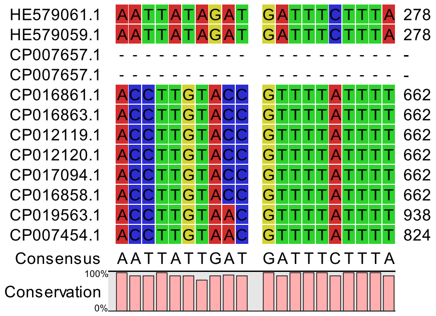
HE579061.1 AATTATAGAT GATTTCTTTA 278
HE579059.1 AATTATAGAT GATTTCTTTA 278
CP007657.1 -
CP007657.1 -
CP016861.1 ACCTTGTACC GTTTTATTTT 662
CP016863.1 ACCTTGTACC GTTTTATTTT 662
CP012119.1 ACCTTGTACC GTTTTATTTT 662
CP012120.1 ACCTTGTACC GTTTTATTTT 662
CP017094.1 ACCTTGTACC GTTTTATTTT 662
CP016858.1 ACCTTGTACC GTTTTATTTT 662
CP019563.1 ACCTTGTAAC GTTTTATTTT 938
CP007454.1 ACCTTGTAAC GTTTTATTTT 824
Consensus AATTATTGAT GATTTCTTTA
Conservation
100%
0%

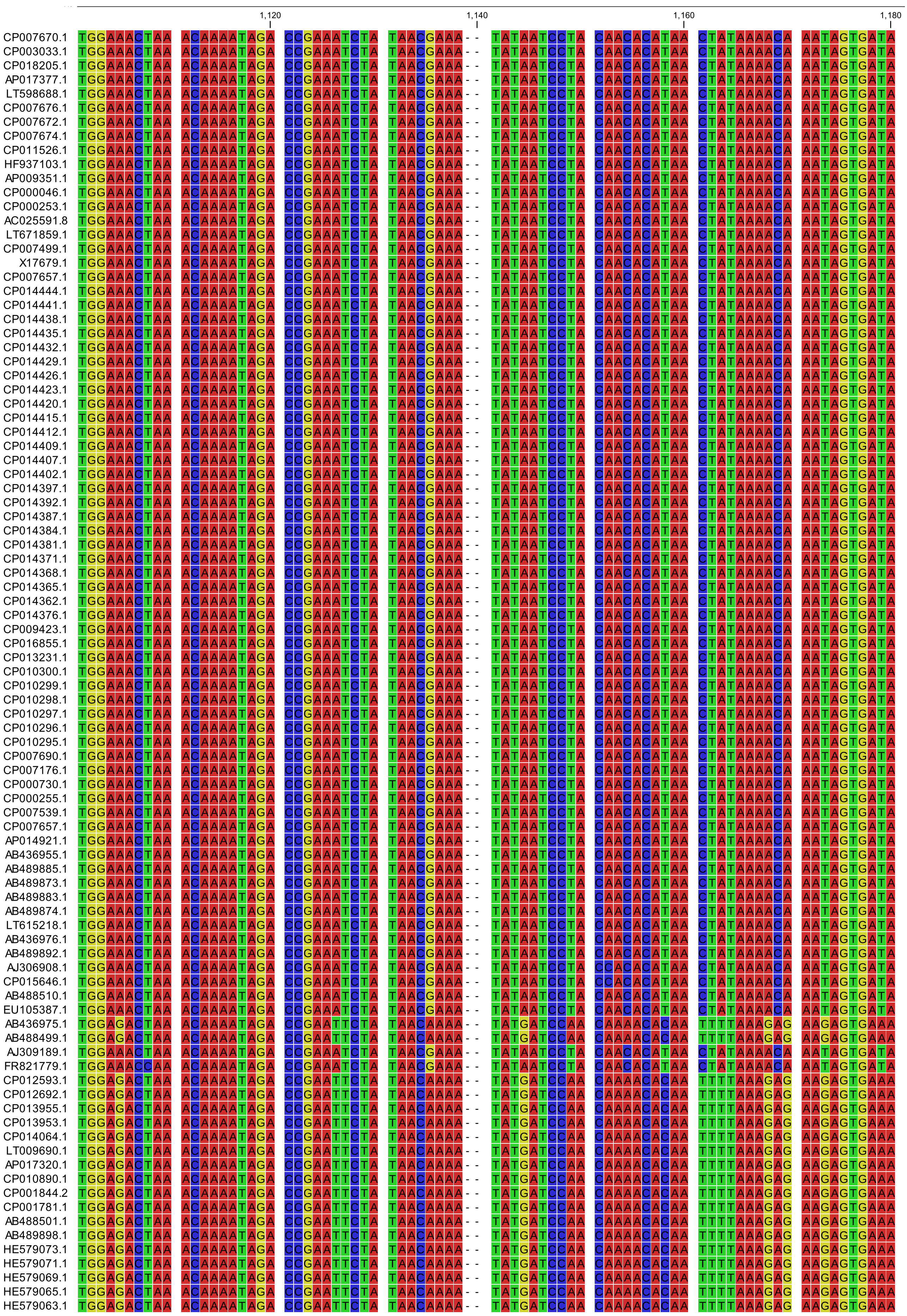
1,120
1,140
1,160
1,180
CP007670.1
CP003033.1
CP018205.1
AP017377.1
LT598688.1
CP007676.1
CP007672.1
CP007674.1
CP011526.1
HF937103.1
AP009351.1
CP000046.1
CP000253.1
AC025591.8
LT671859.1
CP007499.1
X17679.1
CP007657.1
CP014444.1
CP014441.1
CP014438.1
CP014435.1
CP014432.1
CP014429.1
CP014426.1
CP014423.1
CP014420.1
CP014415.1
CP014412.1
CP014409.1
CP014407.1
CP014402.1
CP014397.1
CP014392.1
CP014387.1
CP014384.1
CP014381.1
CP014371.1
CP014368.1
CP014365.1
CP014362.1
CP014376.1
CP009423.1
CP016855.1
CP013231.1
CP010300.1
CP010299.1
CP010298.1
CP010297.1
CP010296.1
CP010295.1
CP007690.1
CP007176.1
CP000730.1
CP000255.1
CP007539.1
CP007657.1
AP014921.1
AB436955.1
AB489885.1
AB489873.1
AB489883.1
AB489874.1
LT615218.1
AB436976.1
AB489892.1
AJ306908.1
CP015646.1
AB488510.1
EU105387.1
AB436975.1
AB488499.1
AJ309189.1
FR821779.1
CP012593.1
CP012692.1
CP013955.1
CP013953.1
CP014064.1
LT009690.1
AP017320.1
CP010890.1
CP001844.2
CP001781.1
AB488501.1
AB489898.1
HE579073.1
HE579071.1
HE579069.1
HE579065.1
HE579063.1

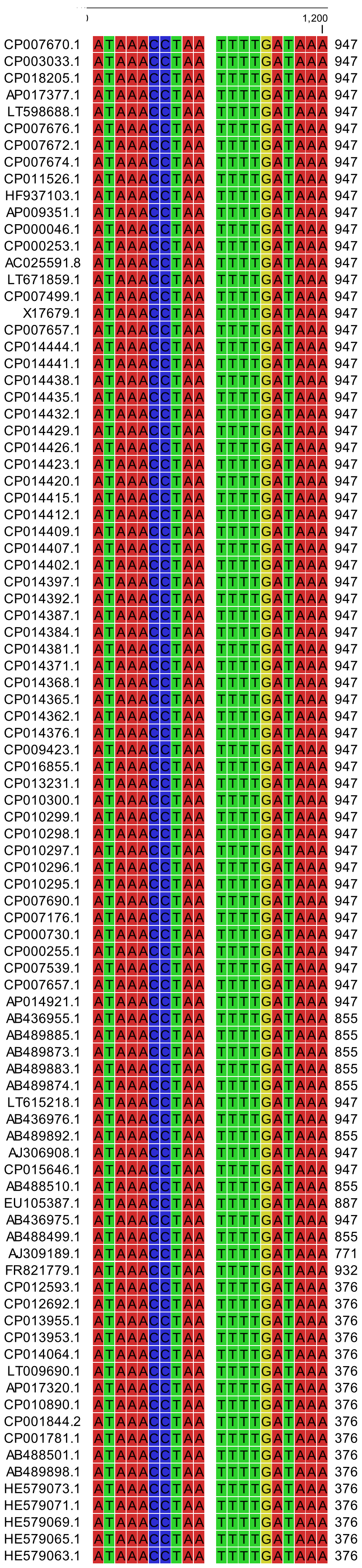
1,200
CP007670.1 ATAAACCTAA TTTTGATAAA 947
CP003033.1 ATAAACCTAA TTTTGATAAA 947
CP018205.1 ATAAACCTAA TTTTGATAAA 947
AP017377.1 ATAAACCTAA TTTTGATAAA 947
LT598688.1 ATAAACCTAA TTTTGATAAA 947
CP007676.1 ATAAACCTAA TTTTGATAAA 947
CP007672.1 ATAAACCTAA TTTTGATAAA 947
CP007674.1 ATAAACCTAA TTTTGATAAA 947
CP011526.1 ATAAACCTAA TTTTGATAAA 947
HF937103.1 ATAAACCTAA TTTTGATAAA 947
AP009351.1 ATAAACCTAA TTTTGATAAA 947
CP000046.1 ATAAACCTAA TTTTGATAAA 947
CP000253.1 ATAAACCTAA TTTTGATAAA 947
AC025591.8 ATAAACCTAA TTTTGATAAA 947
LT671859.1 ATAAACCTAA TTTTGATAAA 947
CP007499.1 ATAAACCTAA TTTTGATAAA 947
X17679.1 ATAAACCTAA TTTTGATAAA 947
CP007657.1 ATAAACCTAA TTTTGATAAA 947
CP014444.1 ATAAACCTAA TTTTGATAAA 947
CP014441.1 ATAAACCTAA TTTTGATAAA 947
CP014438.1 ATAAACCTAA TTTTGATAAA 947
CP014435.1 ATAAACCTAA TTTTGATAAA 947
CP014432.1 ATAAACCTAA TTTTGATAAA 947
CP014429.1 ATAAACCTAA TTTTGATAAA 947
CP014426.1 ATAAACCTAA TTTTGATAAA 947
CP014423.1 ATAAACCTAA TTTTGATAAA 947
CP014420.1 ATAAACCTAA TTTTGATAAA 947
CP014415.1 ATAAACCTAA TTTTGATAAA 947
CP014412.1 ATAAACCTAA TTTTGATAAA 947
CP014409.1 ATAAACCTAA TTTTGATAAA 947
CP014407.1 ATAAACCTAA TTTTGATAAA 947
CP014402.1 ATAAACCTAA TTTTGATAAA 947
CP014397.1 ATAAACCTAA TTTTGATAAA 947
CP014392.1 ATAAACCTAA TTTTGATAAA 947
CP014387.1 ATAAACCTAA TTTTGATAAA 947
CP014384.1 ATAAACCTAA TTTTGATAAA 947
CP014381.1 ATAAACCTAA TTTTGATAAA 947
CP014371.1 ATAAACCTAA TTTTGATAAA 947
CP014368.1 ATAAACCTAA TTTTGATAAA 947
CP014365.1 ATAAACCTAA TTTTGATAAA 947
CP014362.1 ATAAACCTAA TTTTGATAAA 947
CP014376.1 ATAAACCTAA TTTTGATAAA 947
CP009423.1 ATAAACCTAA TTTTGATAAA 947
CP016855.1 ATAAACCTAA TTTTGATAAA 947
CP013231.1 ATAAACCTAA TTTTGATAAA 947
CP010300.1 ATAAACCTAA TTTTGATAAA 947
CP010299.1 ATAAACCTAA TTTTGATAAA 947
CP010298.1 ATAAACCTAA TTTTGATAAA 947
CP010297.1 ATAAACCTAA TTTTGATAAA 947
CP010296.1 ATAAACCTAA TTTTGATAAA 947
CP010295.1 ATAAACCTAA TTTTGATAAA 947
CP007690.1 ATAAACCTAA TTTTGATAAA 947
CP007176.1 ATAAACCTAA TTTTGATAAA 947
CP000730.1 ATAAACCTAA TTTTGATAAA 947
CP000255.1 ATAAACCTAA TTTTGATAAA 947
CP007539.1 ATAAACCTAA TTTTGATAAA 947
CP007657.1 ATAAACCTAA TTTTGATAAA 947
AP014921.1 ATAAACCTAA TTTTGATAAA 947
AB436955.1 ATAAACCTAA TTTTGATAAA 855
AB489885.1 ATAAACCTAA TTTTGATAAA 855
AB489873.1 ATAAACCTAA TTTTGATAAA 855
AB489883.1 ATAAACCTAA TTTTGATAAA 855
AB489874.1 ATAAACCTAA TTTTGATAAA 855
LT615218.1 ATAAACCTAA TTTTGATAAA 947
AB436976.1 ATAAACCTAA TTTTGATAAA 947
AB489892.1 ATAAACCTAA TTTTGATAAA 855
AJ306908.1 ATAAACCTAA TTTTGATAAA 947
CP015646.1 ATAAACCTAA TTTTGATAAA 947
AB488510.1 ATAAACCTAA TTTTGATAAA 855
EU105387.1 ATAAACCTAA TTTTGATAAA 887
AB436975.1 ATAAACCTAA TTTTGATAAA 947
AB488499.1 ATAAACCTAA TTTTGATAAA 855
AJ309189.1 ATAAACCTAA TTTTGATAAA 771
FR821779.1 ATAAACCTAA TTTTGATAAA 932
CP012593.1 ATAAACCTAA TTTTGATAAA 376
CP012692.1 ATAAACCTAA TTTTGATAAA 376
CP013955.1 ATAAACCTAA TTTTGATAAA 376
CP013953.1 ATAAACCTAA TTTTGATAAA 376
CP014064.1 ATAAACCTAA TTTTGATAAA 376
LT009690.1 ATAAACCTAA TTTTGATAAA 376
AP017320.1 ATAAACCTAA TTTTGATAAA 376
CP010890.1 ATAAACCTAA TTTTGATAAA 376
CP001844.2 ATAAACCTAA TTTTGATAAA 376
CP001781.1 ATAAACCTAA TTTTGATAAA 376
AB488501.1 ATAAACCTAA TTTTGATAAA 376
AB489898.1 ATAAACCTAA TTTTGATAAA 376
HE579073.1 ATAAACCTAA TTTTGATAAA 376
HE579071.1 ATAAACCTAA TTTTGATAAA 376
HE579069.1 ATAAACCTAA TTTTGATAAA 376
HE579065.1 ATAAACCTAA TTTTGATAAA 376
HE579063.1 ATAAACCTAA TTTTGATAAA 376

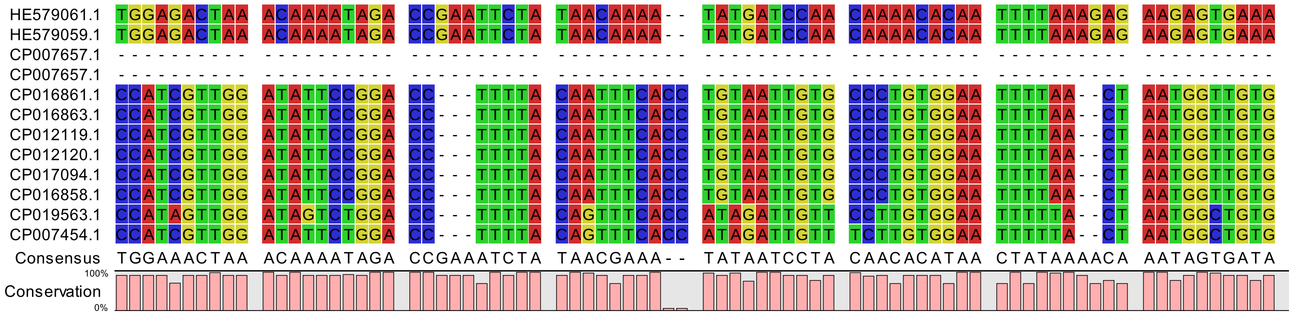
HE579061.1 TGGAGACTAA ACAAAATAGA CCGAATTCTA TAACAAAA-- TATGATCCAA CAAAACACAA TTTTAAAGAG AAGAGTGAAA
HE579059.1 TGGAGACTAA ACAAAATAGA CCGAATTCTA TAACAAAA-- TATGATCCAA CAAAACACAA TTTTAAAGAG AAGAGTGAAA
CP007657.1 ---------- ---------- ---------- ---------- ---------- ---------- ---------- ----------
CP007657.1 ---------- ---------- ---------- ---------- ---------- ---------- ---------- ----------
CP016861.1 CCATCGTTGG ATATTCCGGA CC---TTTTA CAATTTCACC TGTAATTGTG CCCTGTGGAA TTTTAA--CT AATGGTTGTG
CP016863.1 CCATCGTTGG ATATTCCGGA CC---TTTTA CAATTTCACC TGTAATTGTG CCCTGTGGAA TTTTAA--CT AATGGTTGTG
CP012119.1 CCATCGTTGG ATATTCCGGA CC---TTTTA CAATTTCACC TGTAATTGTG CCCTGTGGAA TTTTAA--CT AATGGTTGTG
CP012120.1 CCATCGTTGG ATATTCCGGA CC---TTTTA CAATTTCACC TGTAATTGTG CCCTGTGGAA TTTTAA--CT AATGGTTGTG
CP017094.1 CCATCGTTGG ATATTCCGGA CC---TTTTA CAATTTCACC TGTAATTGTG CCCTGTGGAA TTTTAA--CT AATGGTTGTG
CP016858.1 CCATCGTTGG ATATTCCGGA CC---TTTTA CAATTTCACC TGTAATTGTG CCCTGTGGAA TTTTAA--CT AATGGTTGTG
CP019563.1 CCATAGTTGG ATAGTCTGGA CC---TTTTA CAGTTTCACC ATAGATTGTT CCTTGTGGAA TTTTTA--CT AATGGCTGTG
CP007454.1 CCATCGTTGG ATATTCTGGA CC---TTTTA CAGTTTCACC ATAGATTGTT TCTTGTGGAA TTTTTA--CT AATGGCTGTG
Consensus TGGAAACTAA ACAAAATAGA CCGAAATCTA TAACGAAA-- TATAATCCTA CAACACATAA CTATAAAACA AATAGTGATA
Conservation
100%
0%

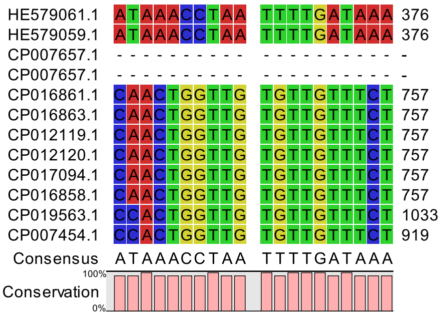

HE579061.1 ATAAACCTAA TTTTGATAAA 376
HE579059.1 ATAAACCTAA TTTTGATAAA 376
CP007657.1 -
CP007657.1 -
CP016861.1 CAACTGGTTG TGTTGTTTCT 757
CP016863.1 CAACTGGTTG TGTTGTTTCT 757
CP012119.1 CAACTGGTTG TGTTGTTTCT 757
CP012120.1 CAACTGGTTG TGTTGTTTCT 757
CP017094.1 CAACTGGTTG TGTTGTTTCT 757
CP016858.1 CAACTGGTTG TGTTGTTTCT 757
CP019563.1 CCACTGGTTG TGTTGTTTCT 1033
CP007454.1 CCACTGGTTG TGTTGTTTCT 919
Consensus ATAAACCTAA TTTTGATAAA
100%
Conservation
0%

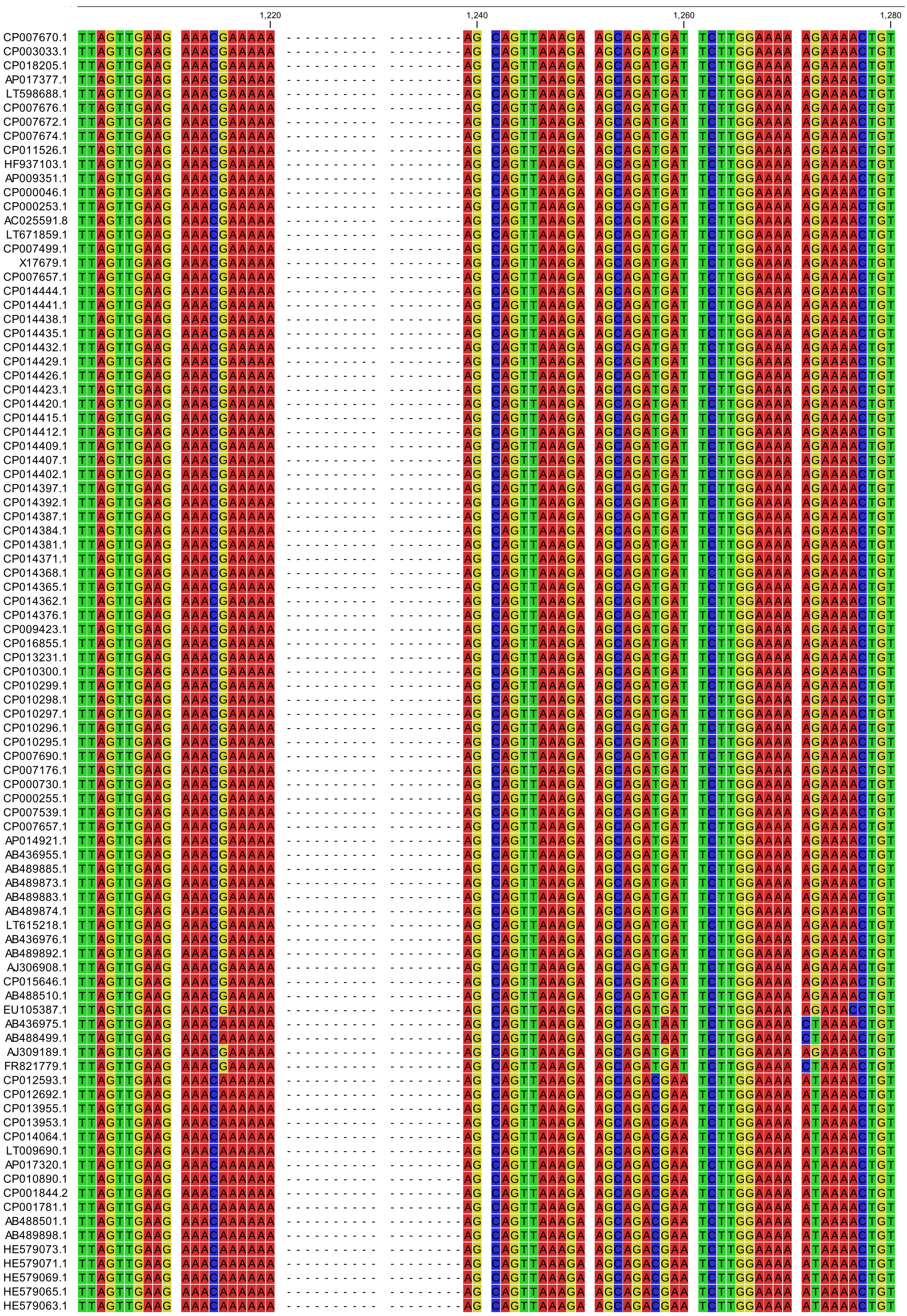
1,220
1,240
1,260
1,280
CP007670.1 TTAGTTGAAG AAACGAAAAA ---------- --------AG CAGTTAAAGA AGCAGATGAT TCTTGGAAAA AGAAAACTGT
CP003033.1 TTAGTTGAAG AAACGAAAAA ---------- --------AG CAGTTAAAGA AGCAGATGAT TCTTGGAAAA AGAAAACTGT
CP018205.1 TTAGTTGAAG AAACGAAAAA ---------- --------AG CAGTTAAAGA AGCAGATGAT TCTTGGAAAA AGAAAACTGT
AP017377.1 TTAGTTGAAG AAACGAAAAA ---------- --------AG CAGTTAAAGA AGCAGATGAT TCTTGGAAAA AGAAAACTGT
LT598688.1 TTAGTTGAAG AAACGAAAAA ---------- --------AG CAGTTAAAGA AGCAGATGAT TCTTGGAAAA AGAAAACTGT
CP007676.1 TTAGTTGAAG AAACGAAAAA ---------- --------AG CAGTTAAAGA AGCAGATGAT TCTTGGAAAA AGAAAACTGT
CP007672.1 TTAGTTGAAG AAACGAAAAA ---------- --------AG CAGTTAAAGA AGCAGATGAT TCTTGGAAAA AGAAAACTGT
CP007674.1 TTAGTTGAAG AAACGAAAAA ---------- --------AG CAGTTAAAGA AGCAGATGAT TCTTGGAAAA AGAAAACTGT
CP011526.1 TTAGTTGAAG AAACGAAAAA ---------- --------AG CAGTTAAAGA AGCAGATGAT TCTTGGAAAA AGAAAACTGT
HF937103.1 TTAGTTGAAG AAACGAAAAA ---------- --------AG CAGTTAAAGA AGCAGATGAT TCTTGGAAAA AGAAAACTGT
AP009351.1 TTAGTTGAAG AAACGAAAAA ---------- --------AG CAGTTAAAGA AGCAGATGAT TCTTGGAAAA AGAAAACTGT
CP000046.1 TTAGTTGAAG AAACGAAAAA ---------- --------AG CAGTTAAAGA AGCAGATGAT TCTTGGAAAA AGAAAACTGT
CP000253.1 TTAGTTGAAG AAACGAAAAA ---------- --------AG CAGTTAAAGA AGCAGATGAT TCTTGGAAAA AGAAAACTGT
AC025591.8 TTAGTTGAAG AAACGAAAAA ---------- --------AG CAGTTAAAGA AGCAGATGAT TCTTGGAAAA AGAAAACTGT
LT671859.1 TTAGTTGAAG AAACGAAAAA ---------- --------AG CAGTTAAAGA AGCAGATGAT TCTTGGAAAA AGAAAACTGT
CP007499.1 TTAGTTGAAG AAACGAAAAA ---------- --------AG CAGTTAAAGA AGCAGATGAT TCTTGGAAAA AGAAAACTGT
X17679.1 TTAGTTGAAG AAACGAAAAA ---------- --------AG CAGTTAAAGA AGCAGATGAT TCTTGGAAAA AGAAAACTGT
CP007657.1 TTAGTTGAAG AAACGAAAAA ---------- --------AG CAGTTAAAGA AGCAGATGAT TCTTGGAAAA AGAAAACTGT
CP014444.1 TTAGTTGAAG AAACGAAAAA ---------- --------AG CAGTTAAAGA AGCAGATGAT TCTTGGAAAA AGAAAACTGT
CP014441.1 TTAGTTGAAG AAACGAAAAA ---------- --------AG CAGTTAAAGA AGCAGATGAT TCTTGGAAAA AGAAAACTGT
CP014438.1 TTAGTTGAAG AAACGAAAAA ---------- --------AG CAGTTAAAGA AGCAGATGAT TCTTGGAAAA AGAAAACTGT
CP014435.1 TTAGTTGAAG AAACGAAAAA ---------- --------AG CAGTTAAAGA AGCAGATGAT TCTTGGAAAA AGAAAACTGT
CP014432.1 TTAGTTGAAG AAACGAAAAA ---------- --------AG CAGTTAAAGA AGCAGATGAT TCTTGGAAAA AGAAAACTGT
CP014429.1 TTAGTTGAAG AAACGAAAAA ---------- --------AG CAGTTAAAGA AGCAGATGAT TCTTGGAAAA AGAAAACTGT
CP014426.1 TTAGTTGAAG AAACGAAAAA ---------- --------AG CAGTTAAAGA AGCAGATGAT TCTTGGAAAA AGAAAACTGT
CP014423.1 TTAGTTGAAG AAACGAAAAA ---------- --------AG CAGTTAAAGA AGCAGATGAT TCTTGGAAAA AGAAAACTGT
CP014420.1 TTAGTTGAAG AAACGAAAAA ---------- --------AG CAGTTAAAGA AGCAGATGAT TCTTGGAAAA AGAAAACTGT
CP014415.1 TTAGTTGAAG AAACGAAAAA ---------- --------AG CAGTTAAAGA AGCAGATGAT TCTTGGAAAA AGAAAACTGT
CP014412.1 TTAGTTGAAG AAACGAAAAA ---------- --------AG CAGTTAAAGA AGCAGATGAT TCTTGGAAAA AGAAAACTGT
CP014409.1 TTAGTTGAAG AAACGAAAAA ---------- --------AG CAGTTAAAGA AGCAGATGAT TCTTGGAAAA AGAAAACTGT
CP014407.1 TTAGTTGAAG AAACGAAAAA ---------- --------AG CAGTTAAAGA AGCAGATGAT TCTTGGAAAA AGAAAACTGT
CP014402.1 TTAGTTGAAG AAACGAAAAA ---------- --------AG CAGTTAAAGA AGCAGATGAT TCTTGGAAAA AGAAAACTGT
CP014397.1 TTAGTTGAAG AAACGAAAAA ---------- --------AG CAGTTAAAGA AGCAGATGAT TCTTGGAAAA AGAAAACTGT
CP014392.1 TTAGTTGAAG AAACGAAAAA ---------- --------AG CAGTTAAAGA AGCAGATGAT TCTTGGAAAA AGAAAACTGT
CP014387.1 TTAGTTGAAG AAACGAAAAA ---------- --------AG CAGTTAAAGA AGCAGATGAT TCTTGGAAAA AGAAAACTGT
CP014384.1 TTAGTTGAAG AAACGAAAAA ---------- --------AG CAGTTAAAGA AGCAGATGAT TCTTGGAAAA AGAAAACTGT
CP014381.1 TTAGTTGAAG AAACGAAAAA ---------- --------AG CAGTTAAAGA AGCAGATGAT TCTTGGAAAA AGAAAACTGT
CP014371.1 TTAGTTGAAG AAACGAAAAA ---------- --------AG CAGTTAAAGA AGCAGATGAT TCTTGGAAAA AGAAAACTGT
CP014368.1 TTAGTTGAAG AAACGAAAAA ---------- --------AG CAGTTAAAGA AGCAGATGAT TCTTGGAAAA AGAAAACTGT
CP014365.1 TTAGTTGAAG AAACGAAAAA ---------- --------AG CAGTTAAAGA AGCAGATGAT TCTTGGAAAA AGAAAACTGT
CP014362.1 TTAGTTGAAG AAACGAAAAA ---------- --------AG CAGTTAAAGA AGCAGATGAT TCTTGGAAAA AGAAAACTGT
CP014376.1 TTAGTTGAAG AAACGAAAAA ---------- --------AG CAGTTAAAGA AGCAGATGAT TCTTGGAAAA AGAAAACTGT
CP009423.1 TTAGTTGAAG AAACGAAAAA ---------- --------AG CAGTTAAAGA AGCAGATGAT TCTTGGAAAA AGAAAACTGT
CP016855.1 TTAGTTGAAG AAACGAAAAA ---------- --------AG CAGTTAAAGA AGCAGATGAT TCTTGGAAAA AGAAAACTGT
CP013231.1 TTAGTTGAAG AAACGAAAAA ---------- --------AG CAGTTAAAGA AGCAGATGAT TCTTGGAAAA AGAAAACTGT
CP010300.1 TTAGTTGAAG AAACGAAAAA ---------- --------AG CAGTTAAAGA AGCAGATGAT TCTTGGAAAA AGAAAACTGT
CP010299.1 TTAGTTGAAG AAACGAAAAA ---------- --------AG CAGTTAAAGA AGCAGATGAT TCTTGGAAAA AGAAAACTGT
CP010298.1 TTAGTTGAAG AAACGAAAAA ---------- --------AG CAGTTAAAGA AGCAGATGAT TCTTGGAAAA AGAAAACTGT
CP010297.1 TTAGTTGAAG AAACGAAAAA ---------- --------AG CAGTTAAAGA AGCAGATGAT TCTTGGAAAA AGAAAACTGT
CP010296.1 TTAGTTGAAG AAACGAAAAA ---------- --------AG CAGTTAAAGA AGCAGATGAT TCTTGGAAAA AGAAAACTGT
CP010295.1 TTAGTTGAAG AAACGAAAAA ---------- --------AG CAGTTAAAGA AGCAGATGAT TCTTGGAAAA AGAAAACTGT
CP007690.1 TTAGTTGAAG AAACGAAAAA ---------- --------AG CAGTTAAAGA AGCAGATGAT TCTTGGAAAA AGAAAACTGT
CP007176.1 TTAGTTGAAG AAACGAAAAA ---------- --------AG CAGTTAAAGA AGCAGATGAT TCTTGGAAAA AGAAAACTGT
CP000730.1 TTAGTTGAAG AAACGAAAAA ---------- --------AG CAGTTAAAGA AGCAGATGAT TCTTGGAAAA AGAAAACTGT
CP000255.1 TTAGTTGAAG AAACGAAAAA ---------- --------AG CAGTTAAAGA AGCAGATGAT TCTTGGAAAA AGAAAACTGT
CP007539.1 TTAGTTGAAG AAACGAAAAA ---------- --------AG CAGTTAAAGA AGCAGATGAT TCTTGGAAAA AGAAAACTGT
CP007657.1 TTAGTTGAAG AAACGAAAAA ---------- --------AG CAGTTAAAGA AGCAGATGAT TCTTGGAAAA AGAAAACTGT
AP014921.1 TTAGTTGAAG AAACGAAAAA ---------- --------AG CAGTTAAAGA AGCAGATGAT TCTTGGAAAA AGAAAACTGT
AB436955.1 TTAGTTGAAG AAACGAAAAA ---------- --------AG CAGTTAAAGA AGCAGATGAT TCTTGGAAAA AGAAAACTGT
AB489885.1 TTAGTTGAAG AAACGAAAAA ---------- --------AG CAGTTAAAGA AGCAGATGAT TCTTGGAAAA AGAAAACTGT
AB489873.1 TTAGTTGAAG AAACGAAAAA ---------- --------AG CAGTTAAAGA AGCAGATGAT TCTTGGAAAA AGAAAACTGT
AB489883.1 TTAGTTGAAG AAACGAAAAA ---------- --------AG CAGTTAAAGA AGCAGATGAT TCTTGGAAAA AGAAAACTGT
AB489874.1 TTAGTTGAAG AAACGAAAAA ---------- --------AG CAGTTAAAGA AGCAGATGAT TCTTGGAAAA AGAAAACTGT
LT615218.1 TTAGTTGAAG AAACGAAAAA ---------- --------AG CAGTTAAAGA AGCAGATGAT TCTTGGAAAA AGAAAACTGT
AB436976.1 TTAGTTGAAG AAACGAAAAA ---------- --------AG CAGTTAAAGA AGCAGATGAT TCTTGGAAAA AGAAAACTGT
AB489892.1 TTAGTTGAAG AAACGAAAAA ---------- --------AG CAGTTAAAGA AGCAGATGAT TCTTGGAAAA AGAAAACTGT
AJ306908.1 TTAGTTGAAG AAACGAAAAA ---------- --------AG CAGTTAAAGA AGCAGATGAT TCTTGGAAAA AGAAAACTGT
CP015646.1 TTAGTTGAAG AAACGAAAAA ---------- --------AG CAGTTAAAGA AGCAGATGAT TCTTGGAAAA AGAAAACTGT
AB488510.1 TTAGTTGAAG AAACGAAAAA ---------- --------AG CAGTTAAAGA AGCAGATGAT TCTTGGAAAA AGAAAACTGT
EU105387.1 TTAGTTGAAG AAACGAAAAA ---------- --------AG CAGTTAAAGA AGCAGATGAT TCTTGGAAAA AGAAACCTGT
AB436975.1 TTAGTTGAAG AAACAAAAAA ---------- --------AG CAGTTAAAGA AGCAGATAAT TCTTGGAAAA CTAAAACTGT
AB488499.1 TTAGTTGAAG AAACAAAAAA ---------- --------AG CAGTTAAAGA AGCAGATAAT TCTTGGAAAA CTAAAACTGT
AJ309189.1 TTAGTTGAAG AAACGAAAAA ---------- --------AG CAGTTAAAGA AGCAGATGAT TCTTGGAAAA AGAAAACTGT
FR821779.1 TTAGTTGAAG AAACGAAAAA ---------- --------AG CAGTTAAAGA AGCAGATGAT TCTTGGAAAA CTAAAACTGT
CP012593.1 TTAGTTGAAG AAACAAAAAA ---------- --------AG CAGTTAAAGA AGCAGACGAA TCTTGGAAAA ATAAAACTGT
CP012692.1 TTAGTTGAAG AAACAAAAAA ---------- --------AG CAGTTAAAGA AGCAGACGAA TCTTGGAAAA ATAAAACTGT
CP013955.1 TTAGTTGAAG AAACAAAAAA ---------- --------AG CAGTTAAAGA AGCAGACGAA TCTTGGAAAA ATAAAACTGT
CP013953.1 TTAGTTGAAG AAACAAAAAA ---------- --------AG CAGTTAAAGA AGCAGACGAA TCTTGGAAAA ATAAAACTGT
CP014064.1 TTAGTTGAAG AAACAAAAAA ---------- --------AG CAGTTAAAGA AGCAGACGAA TCTTGGAAAA ATAAAACTGT
LT009690.1 TTAGTTGAAG AAACAAAAAA ---------- --------AG CAGTTAAAGA AGCAGACGAA TCTTGGAAAA ATAAAACTGT
AP017320.1 TTAGTTGAAG AAACAAAAAA ---------- --------AG CAGTTAAAGA AGCAGACGAA TCTTGGAAAA ATAAAACTGT
CP010890.1 TTAGTTGAAG AAACAAAAAA ---------- --------AG CAGTTAAAGA AGCAGACGAA TCTTGGAAAA ATAAAACTGT
CP001844.2 TTAGTTGAAG AAACAAAAAA ---------- --------AG CAGTTAAAGA AGCAGACGAA TCTTGGAAAA ATAAAACTGT
CP001781.1 TTAGTTGAAG AAACAAAAAA ---------- --------AG CAGTTAAAGA AGCAGACGAA TCTTGGAAAA ATAAAACTGT
AB488501.1 TTAGTTGAAG AAACAAAAAA ---------- --------AG CAGTTAAAGA AGCAGACGAA TCTTGGAAAA ATAAAACTGT
AB489898.1 TTAGTTGAAG AAACAAAAAA ---------- --------AG CAGTTAAAGA AGCAGACGAA TCTTGGAAAA ATAAAACTGT
HE579073.1 TTAGTTGAAG AAACAAAAAA ---------- --------AG CAGTTAAAGA AGCAGACGAA TCTTGGAAAA ATAAAACTGT
HE579071.1 TTAGTTGAAG AAACAAAAAA ---------- --------AG CAGTTAAAGA AGCAGACGAA TCTTGGAAAA ATAAAACTGT
HE579069.1 TTAGTTGAAG AAACAAAAAA ---------- --------AG CAGTTAAAGA AGCAGACGAA TCTTGGAAAA ATAAAACTGT
HE579065.1 TTAGTTGAAG AAACAAAAAA ---------- --------AG CAGTTAAAGA AGCAGACGAA TCTTGGAAAA ATAAAACTGT
HE579063.1 TTAGTTGAAG AAACAAAAAA ---------- --------AG CAGTTAAAGA AGCAGACGAA TCTTGGAAAA ATAAAACTGT

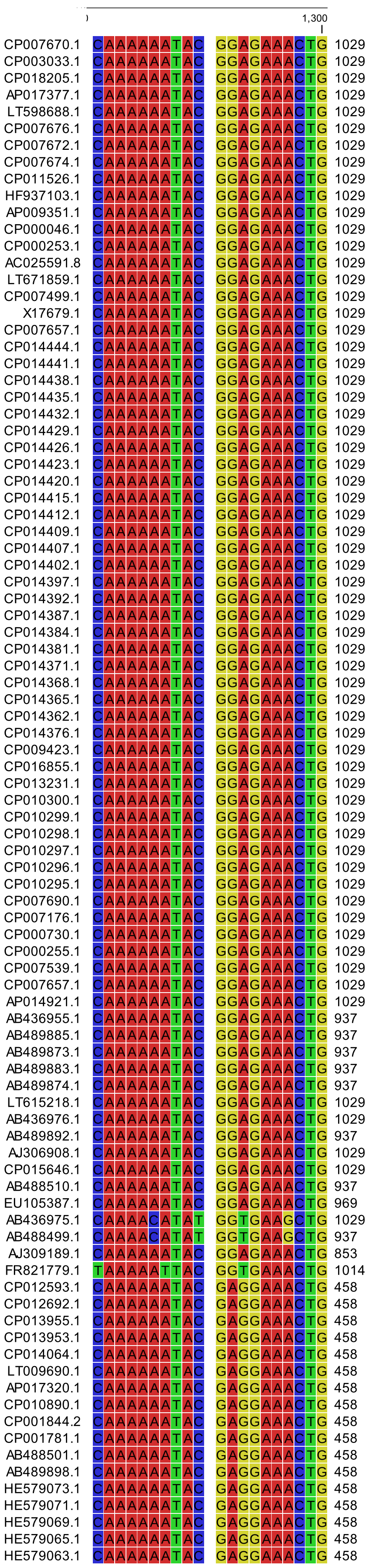
1,300
CP007670.1 CAAAAAATAC GGAGAAACTG 1029
CP003033.1 CAAAAAATAC GGAGAAACTG 1029
CP018205.1 CAAAAAATAC GGAGAAACTG 1029
AP017377.1 CAAAAAATAC GGAGAAACTG 1029
LT598688.1 CAAAAAATAC GGAGAAACTG 1029
CP007676.1 CAAAAAATAC GGAGAAACTG 1029
CP007672.1 CAAAAAATAC GGAGAAACTG 1029
CP007674.1 CAAAAAATAC GGAGAAACTG 1029
CP011526.1 CAAAAAATAC GGAGAAACTG 1029
HF937103.1 CAAAAAATAC GGAGAAACTG 1029
AP009351.1 CAAAAAATAC GGAGAAACTG 1029
CP000046.1 CAAAAAATAC GGAGAAACTG 1029
CP000253.1 CAAAAAATAC GGAGAAACTG 1029
AC025591.8 CAAAAAATAC GGAGAAACTG 1029
LT671859.1 CAAAAAATAC GGAGAAACTG 1029
CP007499.1 CAAAAAATAC GGAGAAACTG 1029
X17679.1 CAAAAAATAC GGAGAAACTG 1029
CP007657.1 CAAAAAATAC GGAGAAACTG 1029
CP014444.1 CAAAAAATAC GGAGAAACTG 1029
CP014441.1 CAAAAAATAC GGAGAAACTG 1029
CP014438.1 CAAAAAATAC GGAGAAACTG 1029
CP014435.1 CAAAAAATAC GGAGAAACTG 1029
CP014432.1 CAAAAAATAC GGAGAAACTG 1029
CP014429.1 CAAAAAATAC GGAGAAACTG 1029
CP014426.1 CAAAAAATAC GGAGAAACTG 1029
CP014423.1 CAAAAAATAC GGAGAAACTG 1029
CP014420.1 CAAAAAATAC GGAGAAACTG 1029
CP014415.1 CAAAAAATAC GGAGAAACTG 1029
CP014412.1 CAAAAAATAC GGAGAAACTG 1029
CP014409.1 CAAAAAATAC GGAGAAACTG 1029
CP014407.1 CAAAAAATAC GGAGAAACTG 1029
CP014402.1 CAAAAAATAC GGAGAAACTG 1029
CP014397.1 CAAAAAATAC GGAGAAACTG 1029
CP014392.1 CAAAAAATAC GGAGAAACTG 1029
CP014387.1 CAAAAAATAC GGAGAAACTG 1029
CP014384.1 CAAAAAATAC GGAGAAACTG 1029
CP014381.1 CAAAAAATAC GGAGAAACTG 1029
CP014371.1 CAAAAAATAC GGAGAAACTG 1029
CP014368.1 CAAAAAATAC GGAGAAACTG 1029
CP014365.1 CAAAAAATAC GGAGAAACTG 1029
CP014362.1 CAAAAAATAC GGAGAAACTG 1029
CP014376.1 CAAAAAATAC GGAGAAACTG 1029
CP009423.1 CAAAAAATAC GGAGAAACTG 1029
CP016855.1 CAAAAAATAC GGAGAAACTG 1029
CP013231.1 CAAAAAATAC GGAGAAACTG 1029
CP010300.1 CAAAAAATAC GGAGAAACTG 1029
CP010299.1 CAAAAAATAC GGAGAAACTG 1029
CP010298.1 CAAAAAATAC GGAGAAACTG 1029
CP010297.1 CAAAAAATAC GGAGAAACTG 1029
CP010296.1 CAAAAAATAC GGAGAAACTG 1029
CP010295.1 CAAAAAATAC GGAGAAACTG 1029
CP007690.1 CAAAAAATAC GGAGAAACTG 1029
CP007176.1 CAAAAAATAC GGAGAAACTG 1029
CP000730.1 CAAAAAATAC GGAGAAACTG 1029
CP000255.1 CAAAAAATAC GGAGAAACTG 1029
CP007539.1 CAAAAAATAC GGAGAAACTG 1029
CP007657.1 CAAAAAATAC GGAGAAACTG 1029
AP014921.1 CAAAAAATAC GGAGAAACTG 1029
AB436955.1 CAAAAAATAC GGAGAAACTG 937
AB489885.1 CAAAAAATAC GGAGAAACTG 937
AB489873.1 CAAAAAATAC GGAGAAACTG 937
AB489883.1 CAAAAAATAC GGAGAAACTG 937
AB489874.1 CAAAAAATAC GGAGAAACTG 937
LT615218.1 CAAAAAATAC GGAGAAACTG 1029
AB436976.1 CAAAAAATAC GGAGAAACTG 1029
AB489892.1 CAAAAAATAC GGAGAAACTG 937
AJ306908.1 CAAAAAATAC GGAGAAACTG 1029
CP015646.1 CAAAAAATAC GGAGAAACTG 1029
AB488510.1 CAAAAAATAC GGAGAAACTG 937
EU105387.1 CAAAAAATAC GGAGAAACTG 969
AB436975.1 CAAAACATAT GGTGAAGCTG 1029
AB488499.1 CAAAACATAT GGTGAAGCTG 937
AJ309189.1 CAAAAAATAC GGAGAAACTG 853
FR821779.1 TAAAAATTAC GGTGAAACTG 1014
CP012593.1 CAAAAAATAC GAGGAAACTG 458
CP012692.1 CAAAAAATAC GAGGAAACTG 458
CP013955.1 CAAAAAATAC GAGGAAACTG 458
CP013953.1 CAAAAAATAC GAGGAAACTG 458
CP014064.1 CAAAAAATAC GAGGAAACTG 458
LT009690.1 CAAAAAATAC GAGGAAACTG 458
AP017320.1 CAAAAAATAC GAGGAAACTG 458
CP010890.1 CAAAAAATAC GAGGAAACTG 458
CP001844.2 CAAAAAATAC GAGGAAACTG 458
CP001781.1 CAAAAAATAC GAGGAAACTG 458
AB488501.1 CAAAAAATAC GAGGAAACTG 458
AB489898.1 CAAAAAATAC GAGGAAACTG 458
HE579073.1 CAAAAAATAC GAGGAAACTG 458
HE579071.1 CAAAAAATAC GAGGAAACTG 458
HE579069.1 CAAAAAATAC GAGGAAACTG 458
HE579065.1 CAAAAAATAC GAGGAAACTG 458
HE579063.1 CAAAAAATAC GAGGAAACTG 458

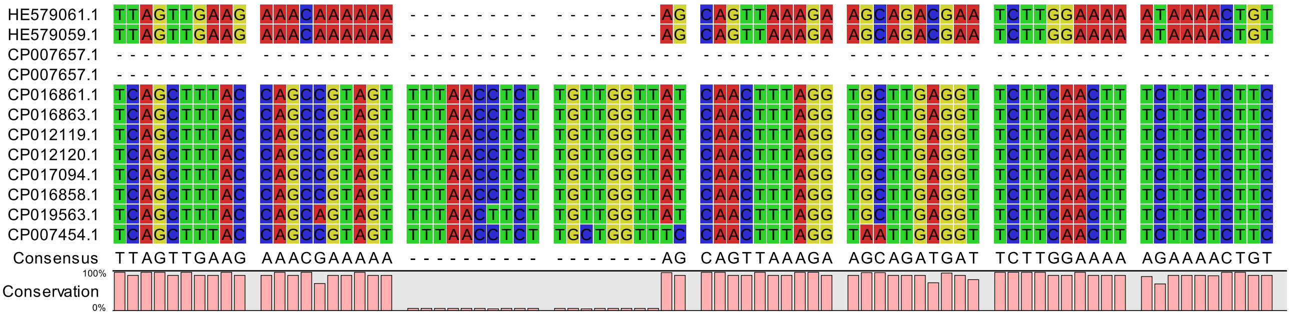
HE579061.1 TTAGTTGAAG AAACAAAAAA ---------- --------AG CAGTTAAAGA AGCAGACGAA TCTTGGAAAA ATAAAACTGT
HE579059.1 TTAGTTGAAG AAACAAAAAA ---------- --------AG CAGTTAAAGA AGCAGACGAA TCTTGGAAAA ATAAAACTGT
CP007657.1 ---------- ---------- ---------- ---------- ---------- ---------- ---------- ----------
CP007657.1 ---------- ---------- ---------- ---------- ---------- ---------- ---------- ----------
CP016861.1 TCAGCTTTAC CAGCCGTAGT TTTAACCTCT TGTTGGTTAT CAACTTTAGG TGCTTGAGGT TCTTCAACTT TCTTCTCTTC
CP016863.1 TCAGCTTTAC CAGCCGTAGT TTTAACCTCT TGTTGGTTAT CAACTTTAGG TGCTTGAGGT TCTTCAACTT TCTTCTCTTC
CP012119.1 TCAGCTTTAC CAGCCGTAGT TTTAACCTCT TGTTGGTTAT CAACTTTAGG TGCTTGAGGT TCTTCAACTT TCTTCTCTTC
CP012120.1 TCAGCTTTAC CAGCCGTAGT TTTAACCTCT TGTTGGTTAT CAACTTTAGG TGCTTGAGGT TCTTCAACTT TCTTCTCTTC
CP017094.1 TCAGCTTTAC CAGCCGTAGT TTTAACCTCT TGTTGGTTAT CAACTTTAGG TGCTTGAGGT TCTTCAACTT TCTTCTCTTC
CP016858.1 TCAGCTTTAC CAGCCGTAGT TTTAACCTCT TGTTGGTTAT CAACTTTAGG TGCTTGAGGT TCTTCAACTT TCTTCTCTTC
CP019563.1 TCAGCTTTAC CAGCAGTAGT TTTAACTTCT TGTTGGTTAT CAACTTTAGG TGCTTGAGGT TCTTCAACTT TCTTCTCTTC
CP007454.1 TCAGCTTTAC CAGCCGTAGT TTTAACCTCT TGCTGGTTTC CAACTTTAGG TAATTGAGGT TCTTCAACTT TCTTCTCTTC
Consensus TTAGTTGAAG AAACGAAAAA ---------- --------AG CAGTTAAAGA AGCAGATGAT TCTTGGAAAA AGAAAACTGT
100%
Conservation
0%

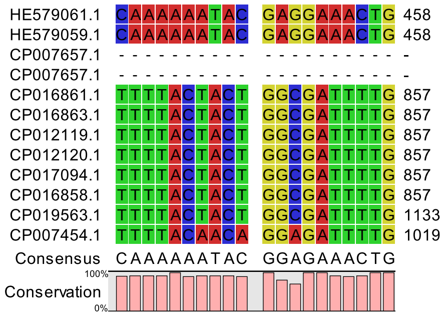
HE579061.1 CAAAAAATAC GAGGAAACTG 458
HE579059.1 CAAAAAATAC GAGGAAACTG 458
CP007657.1 -
CP007657.1 -
CP016861.1 TTTTACTACT GGCGATTTTG 857
CP016863.1 TTTTACTACT GGCGATTTTG 857
CP012119.1 TTTTACTACT GGCGATTTTG 857
CP012120.1 TTTTACTACT GGCGATTTTG 857
CP017094.1 TTTTACTACT GGCGATTTTG 857
CP016858.1 TTTTACTACT GGCGATTTTG 857
CP019563.1 TTTTACTACT GGCGATTTTG 1133
CP007454.1 TTTTACAACA GGAGATTTTG 1019
Consensus CAAAAAATAC GGAGAAACTG
Conservation
100%
0%

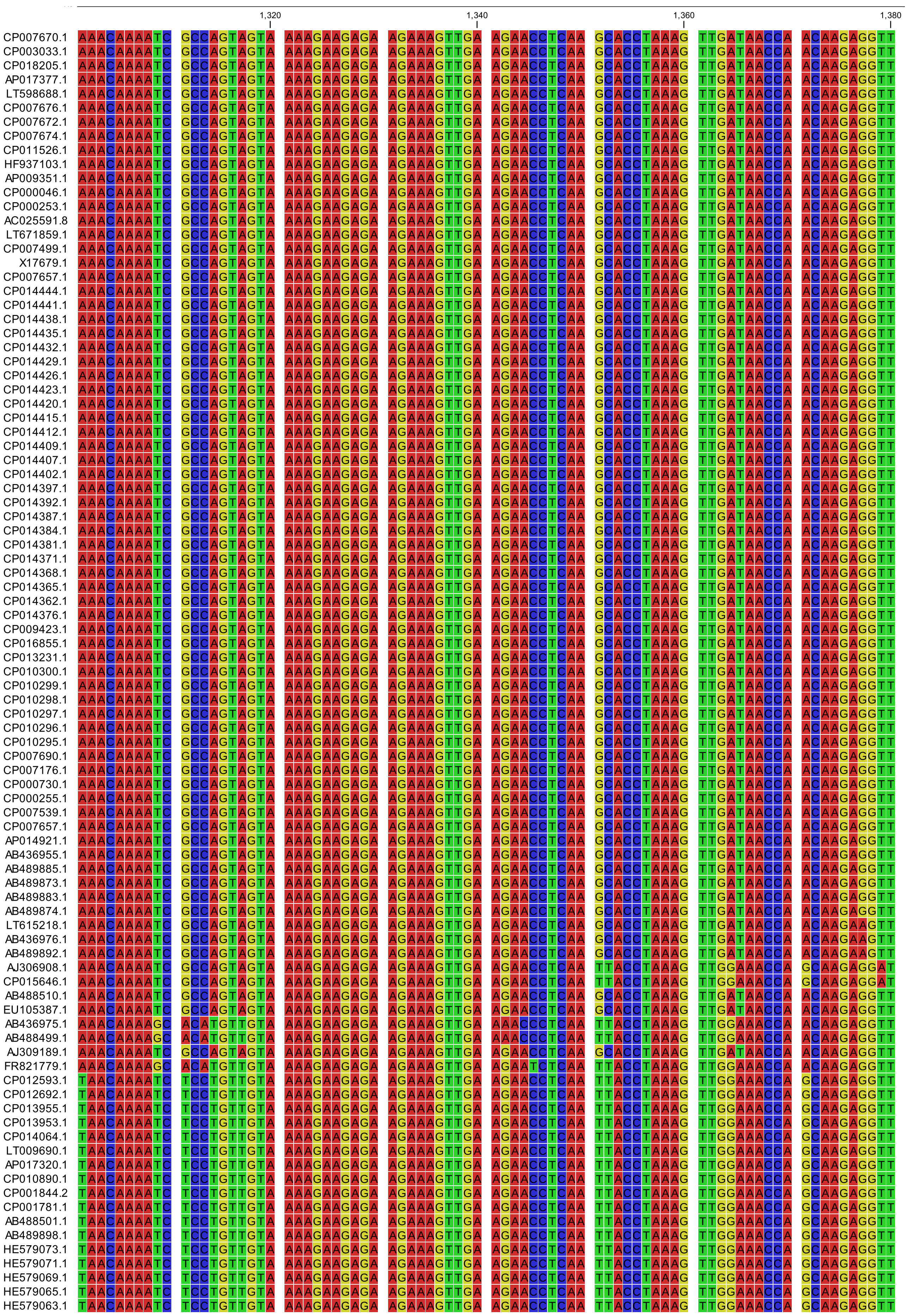
1,320
1,340
1,360
1,380
CP007670.1
CP003033.1
CP018205.1
AP017377.1
LT598688.1
CP007676.1
CP007672.1
CP007674.1
CP011526.1
HF937103.1
AP009351.1
CP000046.1
CP000253.1
AC025591.8
LT671859.1
CP007499.1
X17679.1
CP007657.1
CP014444.1
CP014441.1
CP014438.1
CP014435.1
CP014432.1
CP014429.1
CP014426.1
CP014423.1
CP014420.1
CP014415.1
CP014412.1
CP014409.1
CP014407.1
CP014402.1
CP014397.1
CP014392.1
CP014387.1
CP014384.1
CP014381.1
CP014371.1
CP014368.1
CP014365.1
CP014362.1
CP014376.1
CP009423.1
CP016855.1
CP013231.1
CP010300.1
CP010299.1
CP010298.1
CP010297.1
CP010296.1
CP010295.1
CP007690.1
CP007176.1
CP000730.1
CP000255.1
CP007539.1
CP007657.1
AP014921.1
AB436955.1
AB489885.1
AB489873.1
AB489883.1
AB489874.1
LT615218.1
AB436976.1
AB489892.1
AJ306908.1
CP015646.1
AB488510.1
EU105387.1
AB436975.1
AB488499.1
AJ309189.1
FR821779.1
CP012593.1
CP012692.1
CP013955.1
CP013953.1
CP014064.1
LT009690.1
AP017320.1
CP010890.1
CP001844.2
CP001781.1
AB488501.1
AB489898.1
HE579073.1
HE579071.1
HE579069.1
HE579065.1
HE579063.1

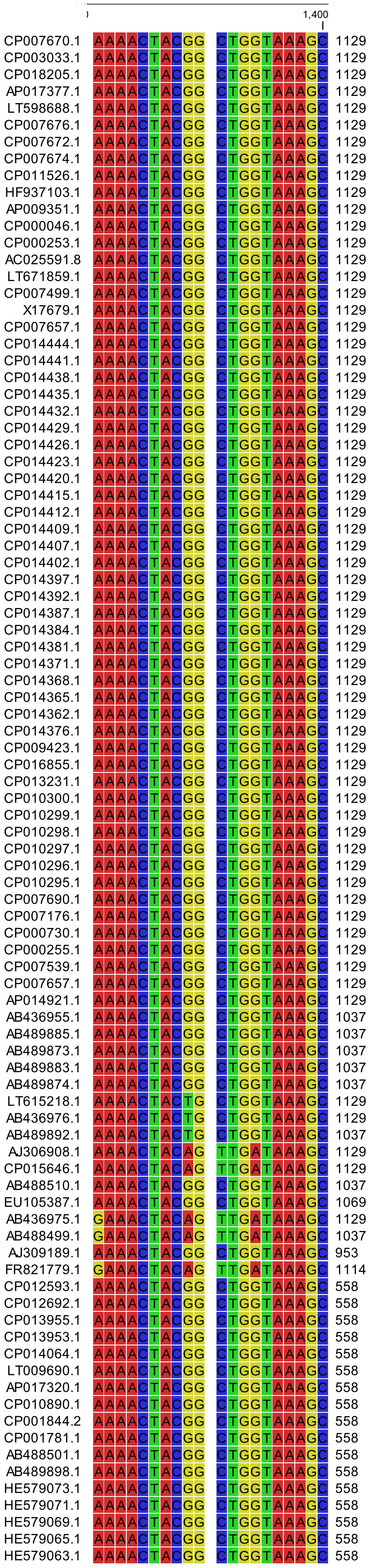
1,400
CP007670.1 AAAACTACGG CTGGTAAAGC 1129
CP003033.1 AAAACTACGG CTGGTAAAGC 1129
CP018205.1 AAAACTACGG CTGGTAAAGC 1129
AP017377.1 AAAACTACGG CTGGTAAAGC 1129
LT598688.1 AAAACTACGG CTGGTAAAGC 1129
CP007676.1 AAAACTACGG CTGGTAAAGC 1129
CP007672.1 AAAACTACGG CTGGTAAAGC 1129
CP007674.1 AAAACTACGG CTGGTAAAGC 1129
CP011526.1 AAAACTACGG CTGGTAAAGC 1129
HF937103.1 AAAACTACGG CTGGTAAAGC 1129
AP009351.1 AAAACTACGG CTGGTAAAGC 1129
CP000046.1 AAAACTACGG CTGGTAAAGC 1129
CP000253.1 AAAACTACGG CTGGTAAAGC 1129
AC025591.8 AAAACTACGG CTGGTAAAGC 1129
LT671859.1 AAAACTACGG CTGGTAAAGC 1129
CP007499.1 AAAACTACGG CTGGTAAAGC 1129
X17679.1 AAAACTACGG CTGGTAAAGC 1129
CP007657.1 AAAACTACGG CTGGTAAAGC 1129
CP014444.1 AAAACTACGG CTGGTAAAGC 1129
CP014441.1 AAAACTACGG CTGGTAAAGC 1129
CP014438.1 AAAACTACGG CTGGTAAAGC 1129
CP014435.1 AAAACTACGG CTGGTAAAGC 1129
CP014432.1 AAAACTACGG CTGGTAAAGC 1129
CP014429.1 AAAACTACGG CTGGTAAAGC 1129
CP014426.1 AAAACTACGG CTGGTAAAGC 1129
CP014423.1 AAAACTACGG CTGGTAAAGC 1129
CP014420.1 AAAACTACGG CTGGTAAAGC 1129
CP014415.1 AAAACTACGG CTGGTAAAGC 1129
CP014412.1 AAAACTACGG CTGGTAAAGC 1129
CP014409.1 AAAACTACGG CTGGTAAAGC 1129
CP014407.1 AAAACTACGG CTGGTAAAGC 1129
CP014402.1 AAAACTACGG CTGGTAAAGC 1129
CP014397.1 AAAACTACGG CTGGTAAAGC 1129
CP014392.1 AAAACTACGG CTGGTAAAGC 1129
CP014387.1 AAAACTACGG CTGGTAAAGC 1129
CP014384.1 AAAACTACGG CTGGTAAAGC 1129
CP014381.1 AAAACTACGG CTGGTAAAGC 1129
CP014371.1 AAAACTACGG CTGGTAAAGC 1129
CP014368.1 AAAACTACGG CTGGTAAAGC 1129
CP014365.1 AAAACTACGG CTGGTAAAGC 1129
CP014362.1 AAAACTACGG CTGGTAAAGC 1129
CP014376.1 AAAACTACGG CTGGTAAAGC 1129
CP009423.1 AAAACTACGG CTGGTAAAGC 1129
CP016855.1 AAAACTACGG CTGGTAAAGC 1129
CP013231.1 AAAACTACGG CTGGTAAAGC 1129
CP010300.1 AAAACTACGG CTGGTAAAGC 1129
CP010299.1 AAAACTACGG CTGGTAAAGC 1129
CP010298.1 AAAACTACGG CTGGTAAAGC 1129
CP010297.1 AAAACTACGG CTGGTAAAGC 1129
CP010296.1 AAAACTACGG CTGGTAAAGC 1129
CP010295.1 AAAACTACGG CTGGTAAAGC 1129
CP007690.1 AAAACTACGG CTGGTAAAGC 1129
CP007176.1 AAAACTACGG CTGGTAAAGC 1129
CP000730.1 AAAACTACGG CTGGTAAAGC 1129
CP000255.1 AAAACTACGG CTGGTAAAGC 1129
CP007539.1 AAAACTACGG CTGGTAAAGC 1129
CP007657.1 AAAACTACGG CTGGTAAAGC 1129
AP014921.1 AAAACTACGG CTGGTAAAGC 1129
AB436955.1 AAAACTACGG CTGGTAAAGC 1037
AB489885.1 AAAACTACGG CTGGTAAAGC 1037
AB489873.1 AAAACTACGG CTGGTAAAGC 1037
AB489883.1 AAAACTACGG CTGGTAAAGC 1037
AB489874.1 AAAACTACGG CTGGTAAAGC 1037
LT615218.1 AAAACTACTG CTGGTAAAGC 1129
AB436976.1 AAAACTACTG CTGGTAAAGC 1129
AB489892.1 AAAACTACTG CTGGTAAAGC 1037
AJ306908.1 AAAACTACAG TTGATAAAGC 1129
CP015646.1 AAAACTACAG TTGATAAAGC 1129
AB488510.1 AAAACTACGG CTGGTAAAGC 1037
EU105387.1 AAAACTACGG CTGGTAAAGC 1069
AB436975.1 GAAACTACAG TTGATAAAGC 1129
AB488499.1 GAAACTACAG TTGATAAAGC 1037
AJ309189.1 AAAACTACGG CTGGTAAAGC 953
FR821779.1 GAAACTACAG TTGATAAAGC 1114
CP012593.1 AAAACTACGG CTGGTAAAGC 558
CP012692.1 AAAACTACGG CTGGTAAAGC 558
CP013955.1 AAAACTACGG CTGGTAAAGC 558
CP013953.1 AAAACTACGG CTGGTAAAGC 558
CP014064.1 AAAACTACGG CTGGTAAAGC 558
LT009690.1 AAAACTACGG CTGGTAAAGC 558
AP017320.1 AAAACTACGG CTGGTAAAGC 558
CP010890.1 AAAACTACGG CTGGTAAAGC 558
CP001844.2 AAAACTACGG CTGGTAAAGC 558
CP001781.1 AAAACTACGG CTGGTAAAGC 558
AB488501.1 AAAACTACGG CTGGTAAAGC 558
AB489898.1 AAAACTACGG CTGGTAAAGC 558
HE579073.1 AAAACTACGG CTGGTAAAGC 558
HE579071.1 AAAACTACGG CTGGTAAAGC 558
HE579069.1 AAAACTACGG CTGGTAAAGC 558
HE579065.1 AAAACTACGG CTGGTAAAGC 558
HE579063.1 AAAACTACGG CTGGTAAAGC 558

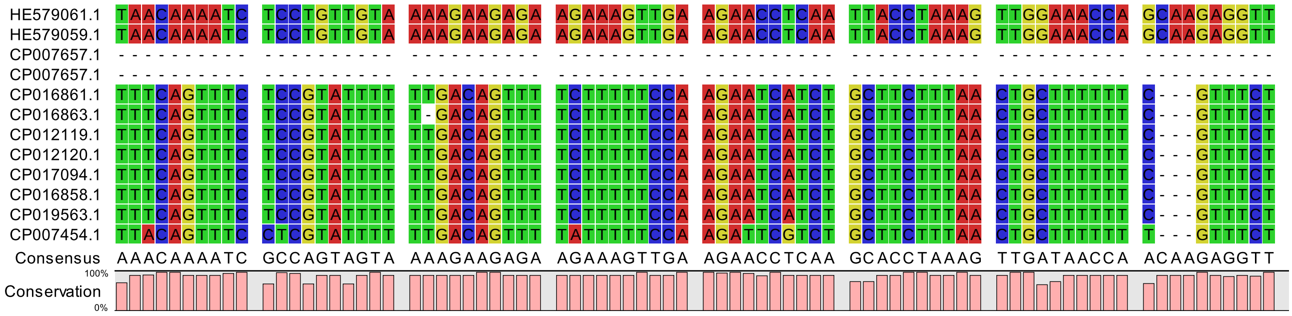
HE579061.1
HE579059.1
CP007657.1
CP007657.1
CP016861.1
CP016863.1
CP012119.1
CP012120.1
CP017094.1
CP016858.1
CP019563.1
CP007454.1
Consensus AAACAAAATC GCCAGTAGTA AAAGAAGAGA AGAAAGTTGA AGAACCTCAA GCACCTAAAG TTGATAACCA ACAAGAGGTT
100%
Conservation
0%

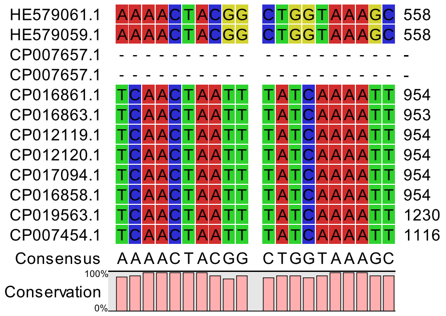
HE579061.1 AAAACTACGG CTGGTAAAGC 558
HE579059.1 AAAACTACGG CTGGTAAAGC 558
CP007657.1 -
CP007657.1 -
CP016861.1 TCAACTAATT TATCAAAATT 954
CP016863.1 TCAACTAATT TATCAAAATT 953
CP012119.1 TCAACTAATT TATCAAAATT 954
CP012120.1 TCAACTAATT TATCAAAATT 954
CP017094.1 TCAACTAATT TATCAAAATT 954
CP016858.1 TCAACTAATT TATCAAAATT 954
CP019563.1 TCAACTAATT TATCAAAATT 1230
CP007454.1 TCAACTAATT TATCAAAATT 1116
Consensus AAAACTACGG CTGGTAAAGC
100%
Conservation
0%

```
                 1,420                         1,440                         1,460                         1,480
CP007670.1  TGAAGAAACA ACACAACCAG TTGCACAACC ATTAGTTAAA ATTCCACAGG GCAC----AA TTACAGGTGA AATTGTAAAA
CP003033.1  TGAAGAAACA ACACAACCAG TTGCACAACC ATTAGTTAAA ATTCCACAGG GCAC----AA TTACAGGTGA AATTGTAAAA
CP018205.1  TGAAGAAACA ACACAACCAG TTGCACAACC ATTAGTTAAA ATTCCACAGG GCAC----AA TTACAGGTGA AATTGTAAAA
AP017377.1  TGAAGAAACA ACACAACCAG TTGCACAACC ATTAGTTAAA ATTCCACAGG GCAC----AA TTACAGGTGA AATTGTAAAA
LT598688.1  TGAAGAAACA ACACAACCAG TTGCACAACC ATTAGTTAAA ATTCCACAGG GCAC----AA TTACAGGTGA AATTGTAAAA
CP007676.1  TGAAGAAACA ACACAACCAG TTGCACAACC ATTAGTTAAA ATTCCACAGG GCAC----AA TTACAGGTGA AATTGTAAAA
CP007672.1  TGAAGAAACA ACACAACCAG TTGCACAACC ATTAGTTAAA ATTCCACAGG GCAC----AA TTACAGGTGA AATTGTAAAA
CP007674.1  TGAAGAAACA ACACAACCAG TTGCACAACC ATTAGTTAAA ATTCCACAGG GCAC----AA TTACAGGTGA AATTGTAAAA
CP011526.1  TGAAGAAACA ACACAACCAG TTGCACAACC ATTAGTTAAA ATTCCACAGG GCAC----AA TTACAGGTGA AATTGTAAAA
HF937103.1  TGAAGAAACA ACACAACCAG TTGCACAACC ATTAGTTAAA ATTCCACAGG GCAC----AA TTACAGGTGA AATTGTAAAA
AP009351.1  TGAAGAAACA ACACAACCAG TTGCACAACC ATTAGTTAAA ATTCCACAGG GCAC----AA TTACAGGTGA AATTGTAAAA
CP000046.1  TGAAGAAACA ACACAACCAG TTGCACAACC ATTAGTTAAA ATTCCACAGG GCAC----AA TTACAGGTGA AATTGTAAAA
CP000253.1  TGAAGAAACA ACACAACCAG TTGCACAACC ATTAGTTAAA ATTCCACAGG GCAC----AA TTACAGGTGA AATTGTAAAA
AC025591.8  TGAAGAAACA ACACAACCAG TTGCACAACC ATTAGTTAAA ATTCCACAGG GCAC----AA TTACAGGTGA AATTGTAAAA
LT671859.1  TGAAGAAACA ACACAACCAG TTGCACAACC ATTAGTTAAA ATTCCACAGG GCAC----AA TTACAGGTGA AATTGTAAAA
CP007499.1  TGAAGAAACA ACACAACCAG TTGCACAACC ATTAGTTAAA ATTCCACAGG GCAC----AA TTACAGGTGA AATTGTAAAA
X17679.1    TGAAGAAACA ACACAACCAG TTGCACAACC ATTAGTTAAA ATTCCACAGG GCAC----AA TTACAGGTGA AATTGTAAAA
CP007657.1  TGAAGAAACA ACACAACCAG TTGCACAACC ATTAGTTAAA ATTCCACAGG GCAC----AA TTACAGGTGA AATTGTAAAA
CP014444.1  TGAAGAAACA ACACAACCAG TTGCACAACC ATTAGTTAAA ATTCCACAGG GCAC----AA TTACAGGTGA AATTGTAAAA
CP014441.1  TGAAGAAACA ACACAACCAG TTGCACAACC ATTAGTTAAA ATTCCACAGG GCAC----AA TTACAGGTGA AATTGTAAAA
CP014438.1  TGAAGAAACA ACACAACCAG TTGCACAACC ATTAGTTAAA ATTCCACAGG GCAC----AA TTACAGGTGA AATTGTAAAA
CP014435.1  TGAAGAAACA ACACAACCAG TTGCACAACC ATTAGTTAAA ATTCCACAGG GCAC----AA TTACAGGTGA AATTGTAAAA
CP014432.1  TGAAGAAACA ACACAACCAG TTGCACAACC ATTAGTTAAA ATTCCACAGG GCAC----AA TTACAGGTGA AATTGTAAAA
CP014429.1  TGAAGAAACA ACACAACCAG TTGCACAACC ATTAGTTAAA ATTCCACAGG GCAC----AA TTACAGGTGA AATTGTAAAA
CP014426.1  TGAAGAAACA ACACAACCAG TTGCACAACC ATTAGTTAAA ATTCCACAGG GCAC----AA TTACAGGTGA AATTGTAAAA
CP014423.1  TGAAGAAACA ACACAACCAG TTGCACAACC ATTAGTTAAA ATTCCACAGG GCAC----AA TTACAGGTGA AATTGTAAAA
CP014420.1  TGAAGAAACA ACACAACCAG TTGCACAACC ATTAGTTAAA ATTCCACAGG GCAC----AA TTACAGGTGA AATTGTAAAA
CP014415.1  TGAAGAAACA ACACAACCAG TTGCACAACC ATTAGTTAAA ATTCCACAGG GCAC----AA TTACAGGTGA AATTGTAAAA
CP014412.1  TGAAGAAACA ACACAACCAG TTGCACAACC ATTAGTTAAA ATTCCACAGG GCAC----AA TTACAGGTGA AATTGTAAAA
CP014409.1  TGAAGAAACA ACACAACCAG TTGCACAACC ATTAGTTAAA ATTCCACAGG GCAC----AA TTACAGGTGA AATTGTAAAA
CP014407.1  TGAAGAAACA ACACAACCAG TTGCACAACC ATTAGTTAAA ATTCCACAGG GCAC----AA TTACAGGTGA AATTGTAAAA
CP014402.1  TGAAGAAACA ACACAACCAG TTGCACAACC ATTAGTTAAA ATTCCACAGG GCAC----AA TTACAGGTGA AATTGTAAAA
CP014397.1  TGAAGAAACA ACACAACCAG TTGCACAACC ATTAGTTAAA ATTCCACAGG GCAC----AA TTACAGGTGA AATTGTAAAA
CP014392.1  TGAAGAAACA ACACAACCAG TTGCACAACC ATTAGTTAAA ATTCCACAGG GCAC----AA TTACAGGTGA AATTGTAAAA
CP014387.1  TGAAGAAACA ACACAACCAG TTGCACAACC ATTAGTTAAA ATTCCACAGG GCAC----AA TTACAGGTGA AATTGTAAAA
CP014384.1  TGAAGAAACA ACACAACCAG TTGCACAACC ATTAGTTAAA ATTCCACAGG GCAC----AA TTACAGGTGA AATTGTAAAA
CP014381.1  TGAAGAAACA ACACAACCAG TTGCACAACC ATTAGTTAAA ATTCCACAGG GCAC----AA TTACAGGTGA AATTGTAAAA
CP014371.1  TGAAGAAACA ACACAACCAG TTGCACAACC ATTAGTTAAA ATTCCACAGG GCAC----AA TTACAGGTGA AATTGTAAAA
CP014368.1  TGAAGAAACA ACACAACCAG TTGCACAACC ATTAGTTAAA ATTCCACAGG GCAC----AA TTACAGGTGA AATTGTAAAA
CP014365.1  TGAAGAAACA ACACAACCAG TTGCACAACC ATTAGTTAAA ATTCCACAGG GCAC----AA TTACAGGTGA AATTGTAAAA
CP014362.1  TGAAGAAACA ACACAACCAG TTGCACAACC ATTAGTTAAA ATTCCACAGG GCAC----AA TTACAGGTGA AATTGTAAAA
CP014376.1  TGAAGAAACA ACACAACCAG TTGCACAACC ATTAGTTAAA ATTCCACAGG GCAC----AA TTACAGGTGA AATTGTAAAA
CP009423.1  TGAAGAAACA ACACAACCAG TTGCACAACC ATTAGTTAAA ATTCCACAGG GCAC----AA TTACAGGTGA AATTGTAAAA
CP016855.1  TGAAGAAACA ACACAACCAG TTGCACAACC ATTAGTTAAA ATTCCACAGG GCAC----AA TTACAGGTGA AATTGTAAAA
CP013231.1  TGAAGAAACA ACACAACCAG TTGCACAACC ATTAGTTAAA ATTCCACAGG GCAC----AA TTACAGGTGA AATTGTAAAA
CP010300.1  TGAAGAAACA ACACAACCAG TTGCACAACC ATTAGTTAAA ATTCCACAGG GCAC----AA TTACAGGTGA AATTGTAAAA
CP010299.1  TGAAGAAACA ACACAACCAG TTGCACAACC ATTAGTTAAA ATTCCACAGG GCAC----AA TTACAGGTGA AATTGTAAAA
CP010298.1  TGAAGAAACA ACACAACCAG TTGCACAACC ATTAGTTAAA ATTCCACAGG GCAC----AA TTACAGGTGA AATTGTAAAA
CP010297.1  TGAAGAAACA ACACAACCAG TTGCACAACC ATTAGTTAAA ATTCCACAGG GCAC----AA TTACAGGTGA AATTGTAAAA
CP010296.1  TGAAGAAACA ACACAACCAG TTGCACAACC ATTAGTTAAA ATTCCACAGG GCAC----AA TTACAGGTGA AATTGTAAAA
CP010295.1  TGAAGAAACA ACACAACCAG TTGCACAACC ATTAGTTAAA ATTCCACAGG GCAC----AA TTACAGGTGA AATTGTAAAA
CP007690.1  TGAAGAAACA ACACAACCAG TTGCACAACC ATTAGTTAAA ATTCCACAGG GCAC----AA TTACAGGTGA AATTGTAAAA
CP007176.1  TGAAGAAACA ACACAACCAG TTGCACAACC ATTAGTTAAA ATTCCACAGG GCAC----AA TTACAGGTGA AATTGTAAAA
CP000730.1  TGAAGAAACA ACACAACCAG TTGCACAACC ATTAGTTAAA ATTCCACAGG GCAC----AA TTACAGGTGA AATTGTAAAA
CP000255.1  TGAAGAAACA ACACAACCAG TTGCACAACC ATTAGTTAAA ATTCCACAGG GCAC----AA TTACAGGTGA AATTGTAAAA
CP007539.1  TGAAGAAACA ACACAACCAG TTGCACAACC ATTAGTTAAA ATTCCACAGG GCAC----AA TTACAGGTGA AATTGTAAAA
CP007657.1  TGAAGAAACA ACACAACCAG TTGCACAACC ATTAGTTAAA ATTCCACAGG GCAC----AA TTACAGGTGA AATTGTAAAA
AP014921.1  TGAAGAAACA ACACAACCAG TTGCACAACC ATTAGTTAAA ATTCCACAGG GCAC----AA TTACAGGTGA AATTGTAAAA
AB436955.1  TGAAGAAACA ACACAACCAG TTGCACAACC ATTAGTTAAA ATTCCACAGG GCAC----AA TTACAGGTGA AATTGTAAAA
AB489885.1  TGAAGAAACA ACACAACCAG TTGCACAACC ATTAGTTAAA ATTCCACAGG GCAC----AA TTACAGGTGA AATTGTAAAA
AB489873.1  TGAAGAAACA ACACAACCAG TTGCACAACC ATTAGTTAAA ATTCCACAGG GCAC----AA TTACAGGTGA AATTGTAAAA
AB489883.1  TGAAGAAACA ACACAACCAG TTGCACAACC ATTAGTTAAA ATTCCACAGG GCAC----AA TTACAGGTGA AATTGTAAAA
AB489874.1  TGAAGAAACA ACACAACCA- ---------- -TTAGTTAAA ATTCCACAGG GCAC----AA TTACAGGTGA AATTGTAAAA
LT615218.1  TGAAGAAACA ACACAACCAG TGGCACAGCC ATTAGTAAAA ATTCCACAAG GAAC----AA TCTATGGTGA AACTGTAAAA
AB436976.1  TGAAGAAACA ACACAACCAG TGGCACAGCC ATTAGTAAAA ATTCCACAAG GAAC----AA TCTATGGTGA AACTGTAAAA
AB489892.1  TGAAGAAACA ACACAACCAG TGGCACAGCC ATTAGTAAAA ATTCCACAAG GAAC----AA TCTATGGTGA AACTGTAAAA
AJ306908.1  TGAAGAAACA ACACAACCAG TGGCACAGCC ATTAGTTAAA ATTCCACAGG GCAC----AA TTACAGGTGA AATTGTAAAA
CP015646.1  TGAAGAAACA ACACAACCAG TGGCACAGCC ATTAGTTAAA ATTCCACAGG GCAC----AA TTACAGGTGA AATTGTAAAA
AB488510.1  TGAAGAAACA ACACAACCAG TTGCACAACC ATTAGTTAAA ATTCCACAGG GCAC----AA TTACAGGTGA AATTGTAAAA
EU105387.1  TGAAGAAACA ACACAACCAG TTGCACAACC ATTAGTTAAA ATTCCACAGG GCAC----AA TTACAGGTGA AATTGTAAAA
AB436975.1  TGAAGAAGCA ACACAACCAG TGGCACAGCC ATTAGTTAAA ATTCCACAGG GCAC----AA TTACAGGTGA AATTGTAAAA
AB488499.1  TGAAGAAGCA ACACAACCAG TGGCACAGCC ATTAGTTAAA ATTCCACAGG GCAC----AA TTACAGGTGA AATTGTAAAA
AJ309189.1  TGAAGAAACA ACACAACCAG TTGCACAACC ATTAGTTAAA ATTCCACAGG GCAC----AA TTACAGGTGA AATTGTAAAA
FR821779.1  TGAAGAAGCA ACACAACCAG TGGCACAGCC ATTAGTTAAA ATTCCACAGG GCAC----AA TTACAGGTGA AATTGTAAAA
CP012593.1  TGAAGAAACA ACACAACCAG TGGCACAGCC ATTAGTAAAA ATTCCACAAG AAAC----AA TCTATGGTGA AACTGTAAAA
CP012692.1  TGAAGAAACA ACACAACCAG TGGCACAGCC ATTAGTAAAA ATTCCACAAG AAAC----AA TCTATGGTGA AACTGTAAAA
CP013955.1  TGAAGAAACA ACACAACCAG TGGCACAGCC ATTAGTAAAA ATTCCACAAG AAAC----AA TCTATGGTGA AACTGTAAAA
CP013953.1  TGAAGAAACA ACACAACCAG TGGCACAGCC ATTAGTAAAA ATTCCACAAG AAAC----AA TCTATGGTGA AACTGTAAAA
CP014064.1  TGAAGAAACA ACACAACCAG TGGCACAGCC ATTAGTAAAA ATTCCACAAG AAAC----AA TCTATGGTGA AACTGTAAAA
LT009690.1  TGAAGAAACA ACACAACCAG TGGCACAGCC ATTAGTAAAA ATTCCACAAG AAAC----AA TCTATGGTGA AACTGTAAAA
AP017320.1  TGAAGAAACA ACACAACCAG TGGCACAGCC ATTAGTAAAA ATTCCACAAG AAAC----AA TCTATGGTGA AACTGTAAAA
CP010890.1  TGAAGAAACA ACACAACCAG TGGCACAGCC ATTAGTAAAA ATTCCACAAG AAAC----AA TCTATGGTGA AACTGTAAAA
CP001844.2  TGAAGAAACA ACACAACCAG TGGCACAGCC ATTAGTAAAA ATTCCACAAG AAAC----AA TCTATGGTGA AACTGTAAAA
CP001781.1  TGAAGAAACA ACACAACCAG TGGCACAGCC ATTAGTAAAA ATTCCACAAG AAAC----AA TCTATGGTGA AACTGTAAAA
AB488501.1  TGAAGAAACA ACACAACCAG TGGCACAGCC ATTAGTAAAA ATTCCACAAG AAAC----AA TCTATGGTGA AACTGTAAAA
AB489898.1  TGAAGAAACA ACACAACCAG TGGCACAGCC ATTAGTAAAA ATTCCACAAG AAAC----AA TCTATGGTGA AACTGTAAAA
HE579073.1  TGAAGAAACA ACACAACCAG TGGCACAGCC ATTAGTAAAA ATTCCACAAG AAAC----AA TCTATGGTGA AACTGTAAAA
HE579071.1  TGAAGAAACA ACACAACCAG TGGCACAGCC ATTAGTAAAA ATTCCACAAG AAAC----AA TCTATGGTGA AACTGTAAAA
HE579069.1  TGAAGAAACA ACACAACCAG TGGCACAGCC ATTAGTAAAA ATTCCACAAG AAAC----AA TCTATGGTGA AACTGTAAAA
HE579065.1  TGAAGAAACA ACACAACCAG TGGCACAGCC ATTAGTAAAA ATTCCACAAG AAAC----AA TCTATGGTGA AACTGTAAAA
HE579063.1  TGAAGAAACA ACACAACCAG TGGCACAGCC ATTAGTAAAA ATTCCACAAG AAAC----AA TCTATGGTGA AACTGTAAAA
```

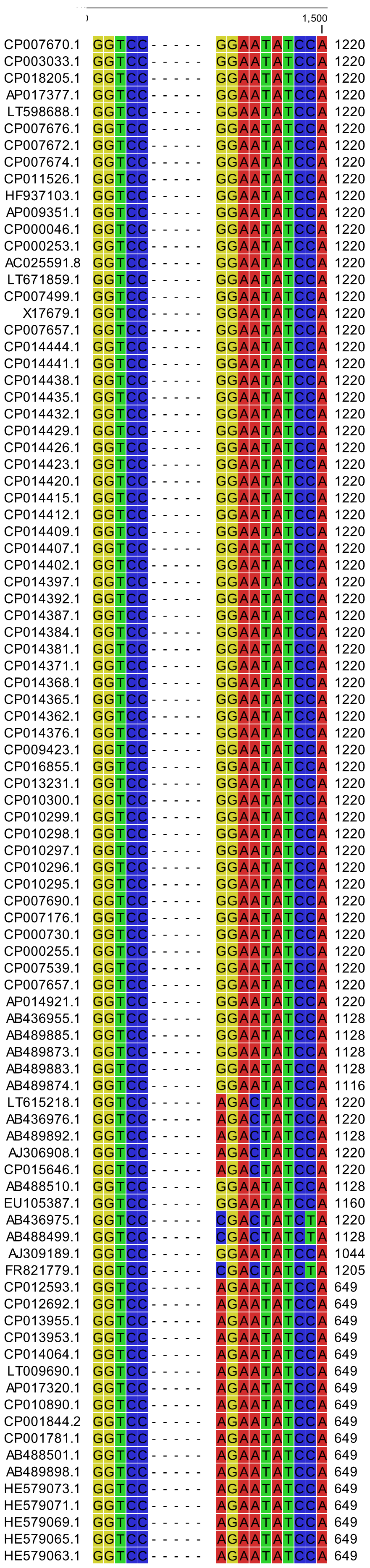
1,500
CP007670.1 GGTCC - - - - - GGAATATCCA 1220
CP003033.1 GGTCC - - - - - GGAATATCCA 1220
CP018205.1 GGTCC - - - - - GGAATATCCA 1220
AP017377.1 GGTCC - - - - - GGAATATCCA 1220
LT598688.1 GGTCC - - - - - GGAATATCCA 1220
CP007676.1 GGTCC - - - - - GGAATATCCA 1220
CP007672.1 GGTCC - - - - - GGAATATCCA 1220
CP007674.1 GGTCC - - - - - GGAATATCCA 1220
CP011526.1 GGTCC - - - - - GGAATATCCA 1220
HF937103.1 GGTCC - - - - - GGAATATCCA 1220
AP009351.1 GGTCC - - - - - GGAATATCCA 1220
CP000046.1 GGTCC - - - - - GGAATATCCA 1220
CP000253.1 GGTCC - - - - - GGAATATCCA 1220
AC025591.8 GGTCC - - - - - GGAATATCCA 1220
LT671859.1 GGTCC - - - - - GGAATATCCA 1220
CP007499.1 GGTCC - - - - - GGAATATCCA 1220
X17679.1 GGTCC - - - - - GGAATATCCA 1220
CP007657.1 GGTCC - - - - - GGAATATCCA 1220
CP014444.1 GGTCC - - - - - GGAATATCCA 1220
CP014441.1 GGTCC - - - - - GGAATATCCA 1220
CP014438.1 GGTCC - - - - - GGAATATCCA 1220
CP014435.1 GGTCC - - - - - GGAATATCCA 1220
CP014432.1 GGTCC - - - - - GGAATATCCA 1220
CP014429.1 GGTCC - - - - - GGAATATCCA 1220
CP014426.1 GGTCC - - - - - GGAATATCCA 1220
CP014423.1 GGTCC - - - - - GGAATATCCA 1220
CP014420.1 GGTCC - - - - - GGAATATCCA 1220
CP014415.1 GGTCC - - - - - GGAATATCCA 1220
CP014412.1 GGTCC - - - - - GGAATATCCA 1220
CP014409.1 GGTCC - - - - - GGAATATCCA 1220
CP014407.1 GGTCC - - - - - GGAATATCCA 1220
CP014402.1 GGTCC - - - - - GGAATATCCA 1220
CP014397.1 GGTCC - - - - - GGAATATCCA 1220
CP014392.1 GGTCC - - - - - GGAATATCCA 1220
CP014387.1 GGTCC - - - - - GGAATATCCA 1220
CP014384.1 GGTCC - - - - - GGAATATCCA 1220
CP014381.1 GGTCC - - - - - GGAATATCCA 1220
CP014371.1 GGTCC - - - - - GGAATATCCA 1220
CP014368.1 GGTCC - - - - - GGAATATCCA 1220
CP014365.1 GGTCC - - - - - GGAATATCCA 1220
CP014362.1 GGTCC - - - - - GGAATATCCA 1220
CP014376.1 GGTCC - - - - - GGAATATCCA 1220
CP009423.1 GGTCC - - - - - GGAATATCCA 1220
CP016855.1 GGTCC - - - - - GGAATATCCA 1220
CP013231.1 GGTCC - - - - - GGAATATCCA 1220
CP010300.1 GGTCC - - - - - GGAATATCCA 1220
CP010299.1 GGTCC - - - - - GGAATATCCA 1220
CP010298.1 GGTCC - - - - - GGAATATCCA 1220
CP010297.1 GGTCC - - - - - GGAATATCCA 1220
CP010296.1 GGTCC - - - - - GGAATATCCA 1220
CP010295.1 GGTCC - - - - - GGAATATCCA 1220
CP007690.1 GGTCC - - - - - GGAATATCCA 1220
CP007176.1 GGTCC - - - - - GGAATATCCA 1220
CP000730.1 GGTCC - - - - - GGAATATCCA 1220
CP000255.1 GGTCC - - - - - GGAATATCCA 1220
CP007539.1 GGTCC - - - - - GGAATATCCA 1220
CP007657.1 GGTCC - - - - - GGAATATCCA 1220
AP014921.1 GGTCC - - - - - GGAATATCCA 1220
AB436955.1 GGTCC - - - - - GGAATATCCA 1128
AB489885.1 GGTCC - - - - - GGAATATCCA 1128
AB489873.1 GGTCC - - - - - GGAATATCCA 1128
AB489883.1 GGTCC - - - - - GGAATATCCA 1128
AB489874.1 GGTCC - - - - - GGAATATCCA 1116
LT615218.1 GGTCC - - - - - AGACTATCCA 1220
AB436976.1 GGTCC - - - - - AGACTATCCA 1220
AB489892.1 GGTCC - - - - - AGACTATCCA 1128
AJ306908.1 GGTCC - - - - - AGACTATCCA 1220
CP015646.1 GGTCC - - - - - AGACTATCCA 1220
AB488510.1 GGTCC - - - - - GGAATATCCA 1128
EU105387.1 GGTCC - - - - - GGAATATCCA 1160
AB436975.1 GGTCC - - - - - CGACTATCTA 1220
AB488499.1 GGTCC - - - - - CGACTATCTA 1128
AJ309189.1 GGTCC - - - - - GGAATATCCA 1044
FR821779.1 GGTCC - - - - - CGACTATCTA 1205
CP012593.1 GGTCC - - - - - AGAATATCCA 649
CP012692.1 GGTCC - - - - - AGAATATCCA 649
CP013955.1 GGTCC - - - - - AGAATATCCA 649
CP013953.1 GGTCC - - - - - AGAATATCCA 649
CP014064.1 GGTCC - - - - - AGAATATCCA 649
LT009690.1 GGTCC - - - - - AGAATATCCA 649
AP017320.1 GGTCC - - - - - AGAATATCCA 649
CP010890.1 GGTCC - - - - - AGAATATCCA 649
CP001844.2 GGTCC - - - - - AGAATATCCA 649
CP001781.1 GGTCC - - - - - AGAATATCCA 649
AB488501.1 GGTCC - - - - - AGAATATCCA 649
AB489898.1 GGTCC - - - - - AGAATATCCA 649
HE579073.1 GGTCC - - - - - AGAATATCCA 649
HE579071.1 GGTCC - - - - - AGAATATCCA 649
HE579069.1 GGTCC - - - - - AGAATATCCA 649
HE579065.1 GGTCC - - - - - AGAATATCCA 649
HE579063.1 GGTCC - - - - - AGAATATCCA 649

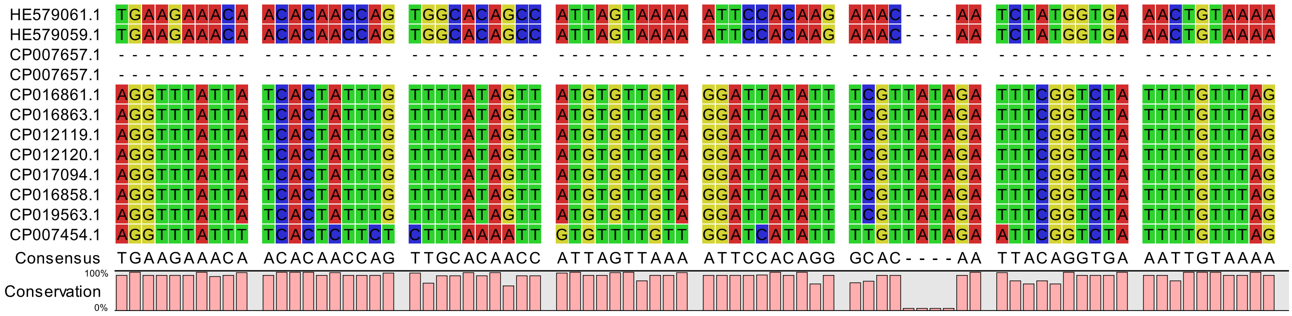

HE579061.1 TGAAGAAACA ACACAACCAG TGGCACAGCC ATTAGTAAAA ATTCCACAAG AAAC----AA TCTATGGTGA AACTGTAAAA
HE579059.1 TGAAGAAACA ACACAACCAG TGGCACAGCC ATTAGTAAAA ATTCCACAAG AAAC----AA TCTATGGTGA AACTGTAAAA
CP007657.1 ---------- ---------- ---------- ---------- ---------- ---------- ---------- ----------
CP007657.1 ---------- ---------- ---------- ---------- ---------- ---------- ---------- ----------
CP016861.1 AGGTTTATTA TCACTATTTG TTTTATAGTT ATGTGTTGTA GGATTATATT TCGTTATAGA TTTCGGTCTA TTTTGTTTAG
CP016863.1 AGGTTTATTA TCACTATTTG TTTTATAGTT ATGTGTTGTA GGATTATATT TCGTTATAGA TTTCGGTCTA TTTTGTTTAG
CP012119.1 AGGTTTATTA TCACTATTTG TTTTATAGTT ATGTGTTGTA GGATTATATT TCGTTATAGA TTTCGGTCTA TTTTGTTTAG
CP012120.1 AGGTTTATTA TCACTATTTG TTTTATAGTT ATGTGTTGTA GGATTATATT TCGTTATAGA TTTCGGTCTA TTTTGTTTAG
CP017094.1 AGGTTTATTA TCACTATTTG TTTTATAGTT ATGTGTTGTA GGATTATATT TCGTTATAGA TTTCGGTCTA TTTTGTTTAG
CP016858.1 AGGTTTATTA TCACTATTTG TTTTATAGTT ATGTGTTGTA GGATTATATT TCGTTATAGA TTTCGGTCTA TTTTGTTTAG
CP019563.1 AGGTTTATTA TCACTATTTG TTTTATAGTT ATGTGTTGTA GGATTATATT TCGTTATAGA TTTCGGTCTA TTTTGTTTAG
CP007454.1 AGGTTTATTT TCACTCTTCT CTTTAAAATT GTGTTTTGTT GGATCATATT TTGTTATAGA ATTCGGTCTA TTTTGTTTAG
Consensus TGAAGAAACA ACACAACCAG TTGCACAACC ATTAGTTAAA ATTCCACAGG GCAC----AA TTACAGGTGA AATTGTAAAA
100%
Conservation
0%

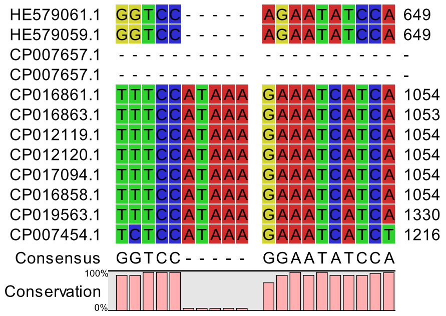
HE579061.1 GGTCC - - - - - AGAATATCCA 649
HE579059.1 GGTCC - - - - - AGAATATCCA 649
CP007657.1 -
CP007657.1 -
CP016861.1 TTTCCATAAA GAAATCATCA 1054
CP016863.1 TTTCCATAAA GAAATCATCA 1053
CP012119.1 TTTCCATAAA GAAATCATCA 1054
CP012120.1 TTTCCATAAA GAAATCATCA 1054
CP017094.1 TTTCCATAAA GAAATCATCA 1054
CP016858.1 TTTCCATAAA GAAATCATCA 1054
CP019563.1 TTTCCATAAA GAAATCATCA 1330
CP007454.1 TCTCCATAAA GAAATCATCT 1216
Consensus GGTCC - - - - - GGAATATCCA
Conservation
100%
0%

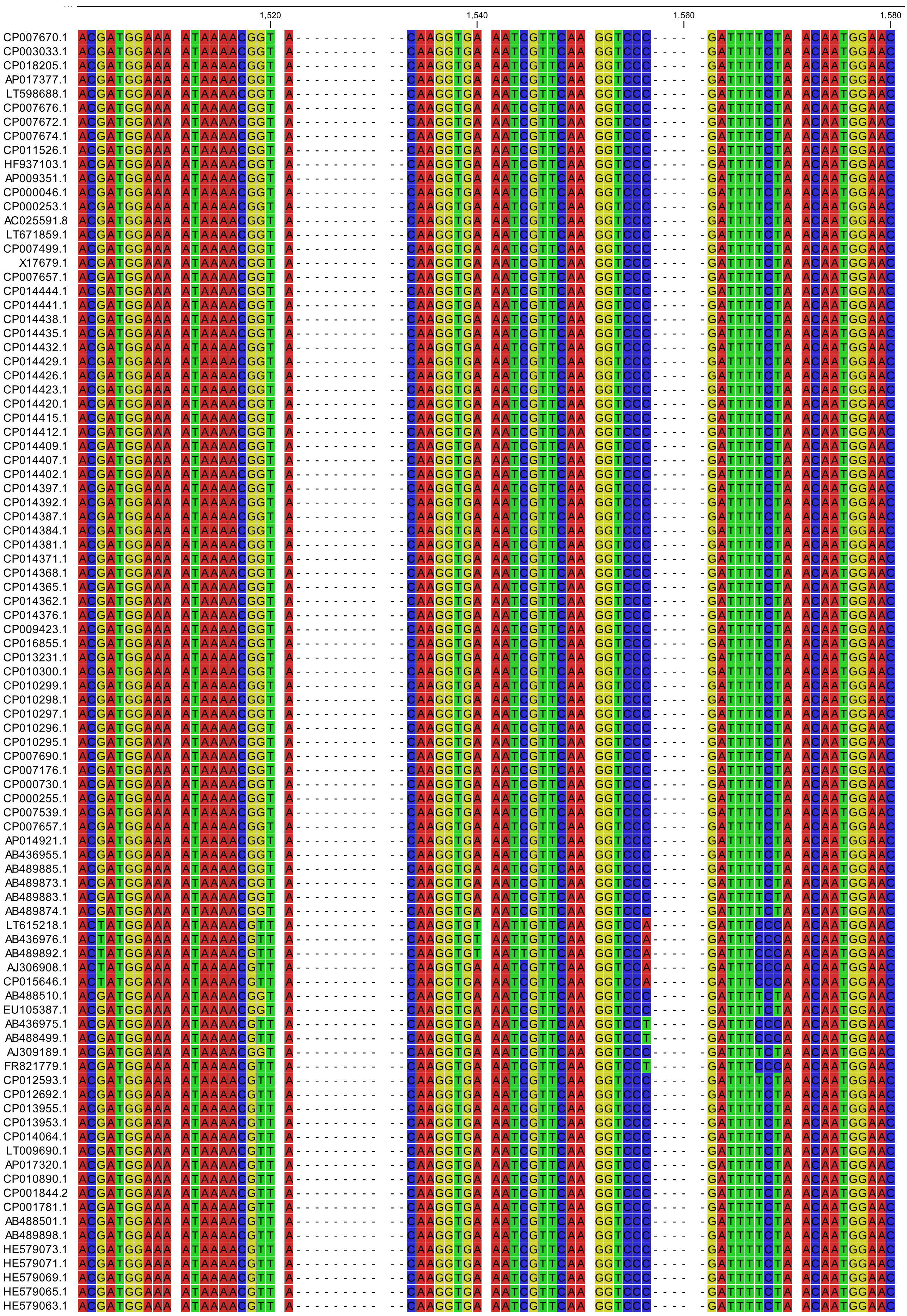
1,520
1,540
1,560
1,580
CP007670.1
CP003033.1
CP018205.1
AP017377.1
LT598688.1
CP007676.1
CP007672.1
CP007674.1
CP011526.1
HF937103.1
AP009351.1
CP000046.1
CP000253.1
AC025591.8
LT671859.1
CP007499.1
X17679.1
CP007657.1
CP014444.1
CP014441.1
CP014438.1
CP014435.1
CP014432.1
CP014429.1
CP014426.1
CP014423.1
CP014420.1
CP014415.1
CP014412.1
CP014409.1
CP014407.1
CP014402.1
CP014397.1
CP014392.1
CP014387.1
CP014384.1
CP014381.1
CP014371.1
CP014368.1
CP014365.1
CP014362.1
CP014376.1
CP009423.1
CP016855.1
CP013231.1
CP010300.1
CP010299.1
CP010298.1
CP010297.1
CP010296.1
CP010295.1
CP007690.1
CP007176.1
CP000730.1
CP000255.1
CP007539.1
CP007657.1
AP014921.1
AB436955.1
AB489885.1
AB489873.1
AB489883.1
AB489874.1
LT615218.1
AB436976.1
AB489892.1
AJ306908.1
CP015646.1
AB488510.1
EU105387.1
AB436975.1
AB488499.1
AJ309189.1
FR821779.1
CP012593.1
CP012692.1
CP013955.1
CP013953.1
CP014064.1
LT009690.1
AP017320.1
CP010890.1
CP001844.2
CP001781.1
AB488501.1
AB489898.1
HE579073.1
HE579071.1
HE579069.1
HE579065.1
HE579063.1

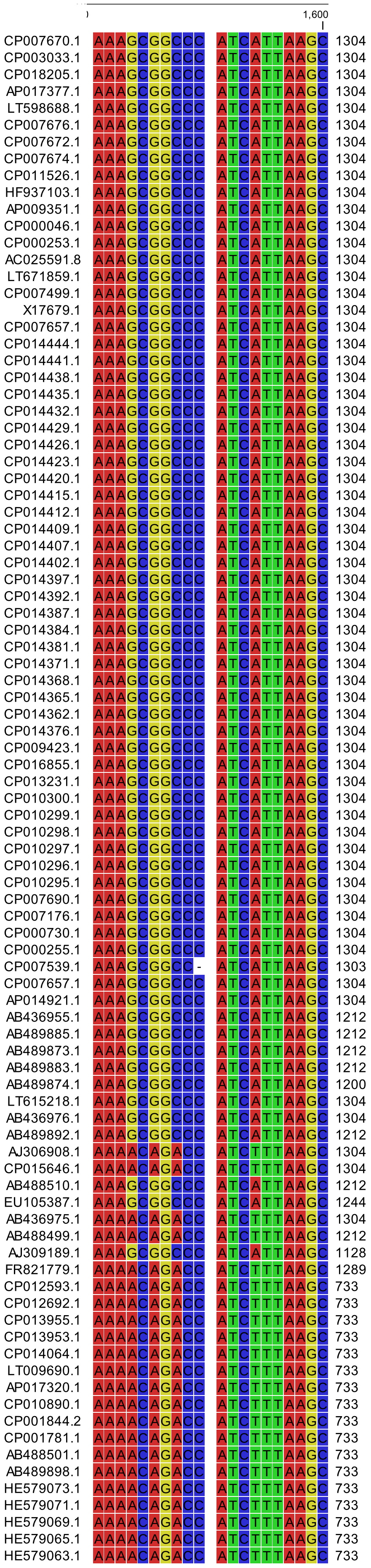
1,600
CP007670.1 AAAGCGGCCC ATCATTAAGC 1304
CP003033.1 AAAGCGGCCC ATCATTAAGC 1304
CP018205.1 AAAGCGGCCC ATCATTAAGC 1304
AP017377.1 AAAGCGGCCC ATCATTAAGC 1304
LT598688.1 AAAGCGGCCC ATCATTAAGC 1304
CP007676.1 AAAGCGGCCC ATCATTAAGC 1304
CP007672.1 AAAGCGGCCC ATCATTAAGC 1304
CP007674.1 AAAGCGGCCC ATCATTAAGC 1304
CP011526.1 AAAGCGGCCC ATCATTAAGC 1304
HF937103.1 AAAGCGGCCC ATCATTAAGC 1304
AP009351.1 AAAGCGGCCC ATCATTAAGC 1304
CP000046.1 AAAGCGGCCC ATCATTAAGC 1304
CP000253.1 AAAGCGGCCC ATCATTAAGC 1304
AC025591.8 AAAGCGGCCC ATCATTAAGC 1304
LT671859.1 AAAGCGGCCC ATCATTAAGC 1304
CP007499.1 AAAGCGGCCC ATCATTAAGC 1304
X17679.1 AAAGCGGCCC ATCATTAAGC 1304
CP007657.1 AAAGCGGCCC ATCATTAAGC 1304
CP014444.1 AAAGCGGCCC ATCATTAAGC 1304
CP014441.1 AAAGCGGCCC ATCATTAAGC 1304
CP014438.1 AAAGCGGCCC ATCATTAAGC 1304
CP014435.1 AAAGCGGCCC ATCATTAAGC 1304
CP014432.1 AAAGCGGCCC ATCATTAAGC 1304
CP014429.1 AAAGCGGCCC ATCATTAAGC 1304
CP014426.1 AAAGCGGCCC ATCATTAAGC 1304
CP014423.1 AAAGCGGCCC ATCATTAAGC 1304
CP014420.1 AAAGCGGCCC ATCATTAAGC 1304
CP014415.1 AAAGCGGCCC ATCATTAAGC 1304
CP014412.1 AAAGCGGCCC ATCATTAAGC 1304
CP014409.1 AAAGCGGCCC ATCATTAAGC 1304
CP014407.1 AAAGCGGCCC ATCATTAAGC 1304
CP014402.1 AAAGCGGCCC ATCATTAAGC 1304
CP014397.1 AAAGCGGCCC ATCATTAAGC 1304
CP014392.1 AAAGCGGCCC ATCATTAAGC 1304
CP014387.1 AAAGCGGCCC ATCATTAAGC 1304
CP014384.1 AAAGCGGCCC ATCATTAAGC 1304
CP014381.1 AAAGCGGCCC ATCATTAAGC 1304
CP014371.1 AAAGCGGCCC ATCATTAAGC 1304
CP014368.1 AAAGCGGCCC ATCATTAAGC 1304
CP014365.1 AAAGCGGCCC ATCATTAAGC 1304
CP014362.1 AAAGCGGCCC ATCATTAAGC 1304
CP014376.1 AAAGCGGCCC ATCATTAAGC 1304
CP009423.1 AAAGCGGCCC ATCATTAAGC 1304
CP016855.1 AAAGCGGCCC ATCATTAAGC 1304
CP013231.1 AAAGCGGCCC ATCATTAAGC 1304
CP010300.1 AAAGCGGCCC ATCATTAAGC 1304
CP010299.1 AAAGCGGCCC ATCATTAAGC 1304
CP010298.1 AAAGCGGCCC ATCATTAAGC 1304
CP010297.1 AAAGCGGCCC ATCATTAAGC 1304
CP010296.1 AAAGCGGCCC ATCATTAAGC 1304
CP010295.1 AAAGCGGCCC ATCATTAAGC 1304
CP007690.1 AAAGCGGCCC ATCATTAAGC 1304
CP007176.1 AAAGCGGCCC ATCATTAAGC 1304
CP000730.1 AAAGCGGCCC ATCATTAAGC 1304
CP000255.1 AAAGCGGCCC ATCATTAAGC 1304
CP007539.1 AAAGCGGCC- ATCATTAAGC 1303
CP007657.1 AAAGCGGCCC ATCATTAAGC 1304
AP014921.1 AAAGCGGCCC ATCATTAAGC 1304
AB436955.1 AAAGCGGCCC ATCATTAAGC 1212
AB489885.1 AAAGCGGCCC ATCATTAAGC 1212
AB489873.1 AAAGCGGCCC ATCATTAAGC 1212
AB489883.1 AAAGCGGCCC ATCATTAAGC 1212
AB489874.1 AAAGCGGCCC ATCATTAAGC 1200
LT615218.1 AAAGCGGCCC ATCATTAAGC 1304
AB436976.1 AAAGCGGCCC ATCATTAAGC 1304
AB489892.1 AAAGCGGCCC ATCATTAAGC 1212
AJ306908.1 AAAACAGACC ATCTTTAAGC 1304
CP015646.1 AAAACAGACC ATCTTTAAGC 1304
AB488510.1 AAAGCGGCCC ATCATTAAGC 1212
EU105387.1 AAAGCGGCCC ATCATTAAGC 1244
AB436975.1 AAAACAGACC ATCTTTAAGC 1304
AB488499.1 AAAACAGACC ATCTTTAAGC 1212
AJ309189.1 AAAGCGGCCC ATCATTAAGC 1128
FR821779.1 AAAACAGACC ATCTTTAAGC 1289
CP012593.1 AAAACAGACC ATCTTTAAGC 733
CP012692.1 AAAACAGACC ATCTTTAAGC 733
CP013955.1 AAAACAGACC ATCTTTAAGC 733
CP013953.1 AAAACAGACC ATCTTTAAGC 733
CP014064.1 AAAACAGACC ATCTTTAAGC 733
LT009690.1 AAAACAGACC ATCTTTAAGC 733
AP017320.1 AAAACAGACC ATCTTTAAGC 733
CP010890.1 AAAACAGACC ATCTTTAAGC 733
CP001844.2 AAAACAGACC ATCTTTAAGC 733
CP001781.1 AAAACAGACC ATCTTTAAGC 733
AB488501.1 AAAACAGACC ATCTTTAAGC 733
AB489898.1 AAAACAGACC ATCTTTAAGC 733
HE579073.1 AAAACAGACC ATCTTTAAGC 733
HE579071.1 AAAACAGACC ATCTTTAAGC 733
HE579069.1 AAAACAGACC ATCTTTAAGC 733
HE579065.1 AAAACAGACC ATCTTTAAGC 733
HE579063.1 AAAACAGACC ATCTTTAAGC 733

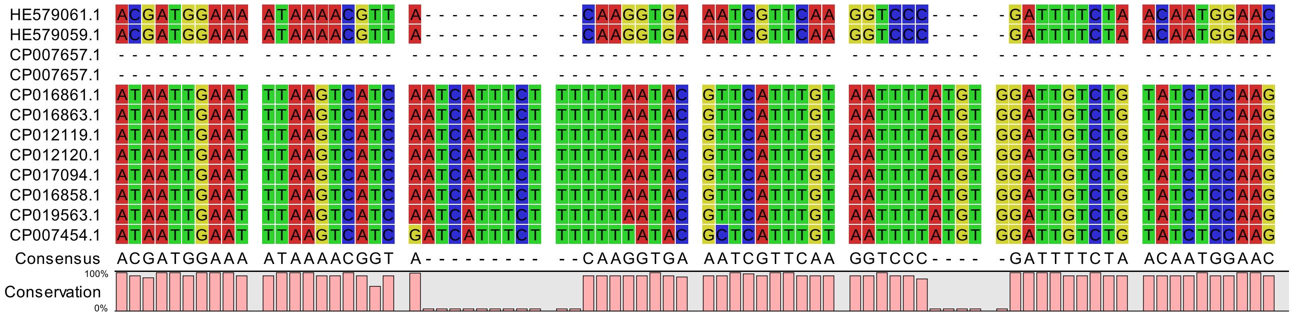
HE579061.1 ACGATGGAAA ATAAAACGTT A--------- --CAAGGTGA AATCGTTCAA GGTCCC---- -GATTTTCTA ACAATGGAAC
HE579059.1 ACGATGGAAA ATAAAACGTT A--------- --CAAGGTGA AATCGTTCAA GGTCCC---- -GATTTTCTA ACAATGGAAC
CP007657.1 ---------- ---------- ---------- ---------- ---------- ---------- ---------- ----------
CP007657.1 ---------- ---------- ---------- ---------- ---------- ---------- ---------- ----------
CP016861.1 ATAATTGAAT TTAAGTCATC AATCATTTCT TTTTTAATAC GTTCATTTGT AATTTTATGT GGATTGTCTG TATCTCCAAG
CP016863.1 ATAATTGAAT TTAAGTCATC AATCATTTCT TTTTTAATAC GTTCATTTGT AATTTTATGT GGATTGTCTG TATCTCCAAG
CP012119.1 ATAATTGAAT TTAAGTCATC AATCATTTCT TTTTTAATAC GTTCATTTGT AATTTTATGT GGATTGTCTG TATCTCCAAG
CP012120.1 ATAATTGAAT TTAAGTCATC AATCATTTCT TTTTTAATAC GTTCATTTGT AATTTTATGT GGATTGTCTG TATCTCCAAG
CP017094.1 ATAATTGAAT TTAAGTCATC AATCATTTCT TTTTTAATAC GTTCATTTGT AATTTTATGT GGATTGTCTG TATCTCCAAG
CP016858.1 ATAATTGAAT TTAAGTCATC AATCATTTCT TTTTTAATAC GTTCATTTGT AATTTTATGT GGATTGTCTG TATCTCCAAG
CP019563.1 ATAATTGAAT TTAAGTCATC AATCATTTCT TTTTTAATAC GTTCATTTGT AATTTTATGT GGATTGTCTG TATCTCCAAG
CP007454.1 ATAATTGAAT TTAAGTCATC GATCATTTCT TTTTTTATAC GCTCATTTGT AATTTTATGT GGATTGTCTG TATCTCCAAG
Consensus ACGATGGAAA ATAAAACGGT A--------- --CAAGGTGA AATCGTTCAA GGTCCC---- -GATTTTCTA ACAATGGAAC
100%
Conservation
0%

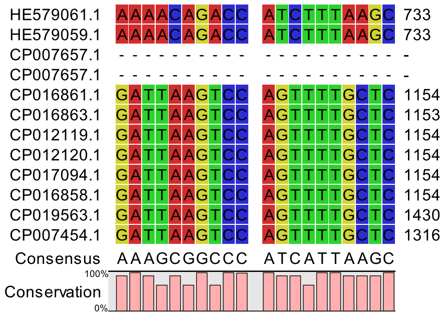
HE579061.1 AAAACAGACC ATCTTTAAGC 733
HE579059.1 AAAACAGACC ATCTTTAAGC 733
CP007657.1 -
CP007657.1 -
CP016861.1 GATTAAGTCC AGTTTTGCTC 1154
CP016863.1 GATTAAGTCC AGTTTTGCTC 1153
CP012119.1 GATTAAGTCC AGTTTTGCTC 1154
CP012120.1 GATTAAGTCC AGTTTTGCTC 1154
CP017094.1 GATTAAGTCC AGTTTTGCTC 1154
CP016858.1 GATTAAGTCC AGTTTTGCTC 1154
CP019563.1 GATTAAGTCC AGTTTTGCTC 1430
CP007454.1 GATTAAGTCC AGTTTTGCTC 1316
Consensus AAAGCGGCCC ATCATTAAGC
Conservation
100%
0%

1,620 1,640 1,660 1,680

CP007670.1	AATAATTATA	CAAACCCACC	GTTAACGAAC	CCTATT-TTA	GAAGGTCTTG	AAGGTAGCTC	ATCTAAACTT	GAAATAAAAC
CP003033.1	AATAATTATA	CAAACCCACC	GTTAACGAAC	CCTATT-TTA	GAAGGTCTTG	AAGGTAGCTC	ATCTAAACTT	GAAATAAAAC
CP018205.1	AATAATTATA	CAAACCCACC	GTTAACGAAC	CCTATT-TTA	GAAGGTCTTG	AAGGTAGCTC	ATCTAAACTT	GAAATAAAAC
AP017377.1	AATAATTATA	CAAACCCACC	GTTAACGAAC	CCTATT-TTA	GAAGGTCTTG	AAGGTAGCTC	ATCTAAACTT	GAAATAAAAC
LT598688.1	AATAATTATA	CAAACCCACC	GTTAACGAAC	CCTATT-TTA	GAAGGTCTTG	AAGGTAGCTC	ATCTAAACTT	GAAATAAAAC
CP007676.1	AATAATTATA	CAAACCCACC	GTTAACGAAC	CCTATT-TTA	GAAGGTCTTG	AAGGTAGCTC	ATCTAAACTT	GAAATAAAAC
CP007672.1	AATAATTATA	CAAACCCACC	GTTAACGAAC	CCTATT-TTA	GAAGGTCTTG	AAGGTAGCTC	ATCTAAACTT	GAAATAAAAC
CP007674.1	AATAATTATA	CAAACCCACC	GTTAACGAAC	CCTATT-TTA	GAAGGTCTTG	AAGGTAGCTC	ATCTAAACTT	GAAATAAAAC
CP011526.1	AATAATTATA	CAAACCCACC	GTTAACGAAC	CCTATT-TTA	GAAGGTCTTG	AAGGTAGCTC	ATCTAAACTT	GAAATAAAAC
HF937103.1	AATAATTATA	CAAACCCACC	GTTAACGAAC	CCTATT-TTA	GAAGGTCTTG	AAGGTAGCTC	ATCTAAACTT	GAAATAAAAC
AP009351.1	AATAATTATA	CAAACCCACC	GTTAACGAAC	CCTATT-TTA	GAAGGTCTTG	AAGGTAGCTC	ATCTAAACTT	GAAATAAAAC
CP000046.1	AATAATTATA	CAAACCCACC	GTTAACGAAC	CCTATT-TTA	GAAGGTCTTG	AAGGTAGCTC	ATCTAAACTT	GAAATAAAAC
CP000253.1	AATAATTATA	CAAACCCACC	GTTAACGAAC	CCTATT-TTA	GAAGGTCTTG	AAGGTAGCTC	ATCTAAACTT	GAAATAAAAC
AC025591.8	AATAATTATA	CAAACCCACC	GTTAACGAAC	CCTATT-TTA	GAAGGTCTTG	AAGGTAGCTC	ATCTAAACTT	GAAATAAAAC
LT671859.1	AATAATTATA	CAAACCCACC	GTTAACGAAC	CCTATT-TTA	GAAGGTCTTG	AAGGTAGCTC	ATCTAAACTT	GAAATAAAAC
CP007499.1	AATAATTATA	CAAACCCACC	GTTAACGAAC	CCTATT-TTA	GAAGGTCTTG	AAGGTAGCTC	ATCTAAACTT	GAAATAAAAC
X17679.1	AATAATTATA	CAAACCCACC	GTTAACGAAC	CCTATT-TTA	GAAGGTCTTG	AAGGTAGCTC	ATCTAAACTT	GAAATAAAAC
CP007657.1	AATAATTATA	CAAACCCACC	GTTAACGAAC	CCTATT-TTA	GAAGGTCTTG	AAGGTAGCTC	ATCTAAACTT	GAAATAAAAC
CP014444.1	AATAATTATA	CAAACCCACC	GTTAACGAAC	CCTATT-TTA	GAAGGTCTTG	AAGGTAGCTC	ATCTAAACTT	GAAATAAAAC
CP014441.1	AATAATTATA	CAAACCCACC	GTTAACGAAC	CCTATT-TTA	GAAGGTCTTG	AAGGTAGCTC	ATCTAAACTT	GAAATAAAAC
CP014438.1	AATAATTATA	CAAACCCACC	GTTAACGAAC	CCTATT-TTA	GAAGGTCTTG	AAGGTAGCTC	ATCTAAACTT	GAAATAAAAC
CP014435.1	AATAATTATA	CAAACCCACC	GTTAACGAAC	CCTATT-TTA	GAAGGTCTTG	AAGGTAGCTC	ATCTAAACTT	GAAATAAAAC
CP014432.1	AATAATTATA	CAAACCCACC	GTTAACGAAC	CCTATT-TTA	GAAGGTCTTG	AAGGTAGCTC	ATCTAAACTT	GAAATAAAAC
CP014429.1	AATAATTATA	CAAACCCACC	GTTAACGAAC	CCTATT-TTA	GAAGGTCTTG	AAGGTAGCTC	ATCTAAACTT	GAAATAAAAC
CP014426.1	AATAATTATA	CAAACCCACC	GTTAACGAAC	CCTATT-TTA	GAAGGTCTTG	AAGGTAGCTC	ATCTAAACTT	GAAATAAAAC
CP014423.1	AATAATTATA	CAAACCCACC	GTTAACGAAC	CCTATT-TTA	GAAGGTCTTG	AAGGTAGCTC	ATCTAAACTT	GAAATAAAAC
CP014420.1	AATAATTATA	CAAACCCACC	GTTAACGAAC	CCTATT-TTA	GAAGGTCTTG	AAGGTAGCTC	ATCTAAACTT	GAAATAAAAC
CP014415.1	AATAATTATA	CAAACCCACC	GTTAACGAAC	CCTATT-TTA	GAAGGTCTTG	AAGGTAGCTC	ATCTAAACTT	GAAATAAAAC
CP014412.1	AATAATTATA	CAAACCCACC	GTTAACGAAC	CCTATT-TTA	GAAGGTCTTG	AAGGTAGCTC	ATCTAAACTT	GAAATAAAAC
CP014409.1	AATAATTATA	CAAACCCACC	GTTAACGAAC	CCTATT-TTA	GAAGGTCTTG	AAGGTAGCTC	ATCTAAACTT	GAAATAAAAC
CP014407.1	AATAATTATA	CAAACCCACC	GTTAACGAAC	CCTATT-TTA	GAAGGTCTTG	AAGGTAGCTC	ATCTAAACTT	GAAATAAAAC
CP014402.1	AATAATTATA	CAAACCCACC	GTTAACGAAC	CCTATT-TTA	GAAGGTCTTG	AAGGTAGCTC	ATCTAAACTT	GAAATAAAAC
CP014397.1	AATAATTATA	CAAACCCACC	GTTAACGAAC	CCTATT-TTA	GAAGGTCTTG	AAGGTAGCTC	ATCTAAACTT	GAAATAAAAC
CP014392.1	AATAATTATA	CAAACCCACC	GTTAACGAAC	CCTATT-TTA	GAAGGTCTTG	AAGGTAGCTC	ATCTAAACTT	GAAATAAAAC
CP014387.1	AATAATTATA	CAAACCCACC	GTTAACGAAC	CCTATT-TTA	GAAGGTCTTG	AAGGTAGCTC	ATCTAAACTT	GAAATAAAAC
CP014384.1	AATAATTATA	CAAACCCACC	GTTAACGAAC	CCTATT-TTA	GAAGGTCTTG	AAGGTAGCTC	ATCTAAACTT	GAAATAAAAC
CP014381.1	AATAATTATA	CAAACCCACC	GTTAACGAAC	CCTATT-TTA	GAAGGTCTTG	AAGGTAGCTC	ATCTAAACTT	GAAATAAAAC
CP014371.1	AATAATTATA	CAAACCCACC	GTTAACGAAC	CCTATT-TTA	GAAGGTCTTG	AAGGTAGCTC	ATCTAAACTT	GAAATAAAAC
CP014368.1	AATAATTATA	CAAACCCACC	GTTAACGAAC	CCTATT-TTA	GAAGGTCTTG	AAGGTAGCTC	ATCTAAACTT	GAAATAAAAC
CP014365.1	AATAATTATA	CAAACCCACC	GTTAACGAAC	CCTATT-TTA	GAAGGTCTTG	AAGGTAGCTC	ATCTAAACTT	GAAATAAAAC
CP014362.1	AATAATTATA	CAAACCCACC	GTTAACGAAC	CCTATT-TTA	GAAGGTCTTG	AAGGTAGCTC	ATCTAAACTT	GAAATAAAAC
CP014376.1	AATAATTATA	CAAACCCACC	GTTAACGAAC	CCTATT-TTA	GAAGGTCTTG	AAGGTAGCTC	ATCTAAACTT	GAAATAAAAC
CP009423.1	AATAATTATA	CAAACCCACC	GTTAACGAAC	CCTATT-TTA	GAAGGTCTTG	AAGGTAGCTC	ATCTAAACTT	GAAATAAAAC
CP016855.1	AATAATTATA	CAAACCCACC	GTTAACGAAC	CCTATT-TTA	GAAGGTCTTG	AAGGTAGCTC	ATCTAAACTT	GAAATAAAAC
CP013231.1	AATAATTATA	CAAACCCACC	GTTAACGAAC	CCTATT-TTA	GAAGGTCTTG	AAGGTAGCTC	ATCTAAACTT	GAAATAAAAC
CP010300.1	AATAATTATA	CAAACCCACC	GTTAACGAAC	CCTATT-TTA	GAAGGTCTTG	AAGGTAGCTC	ATCTAAACTT	GAAATAAAAC
CP010299.1	AATAATTATA	CAAACCCACC	GTTAACGAAC	CCTATT-TTA	GAAGGTCTTG	AAGGTAGCTC	ATCTAAACTT	GAAATAAAAC
CP010298.1	AATAATTATA	CAAACCCACC	GTTAACGAAC	CCTATT-TTA	GAAGGTCTTG	AAGGTAGCTC	ATCTAAACTT	GAAATAAAAC
CP010297.1	AATAATTATA	CAAACCCACC	GTTAACGAAC	CCTATT-TTA	GAAGGTCTTG	AAGGTAGCTC	ATCTAAACTT	GAAATAAAAC
CP010296.1	AATAATTATA	CAAACCCACC	GTTAACGAAC	CCTATT-TTA	GAAGGTCTTG	AAGGTAGCTC	ATCTAAACTT	GAAATAAAAC
CP010295.1	AATAATTATA	CAAACCCACC	GTTAACGAAC	CCTATT-TTA	GAAGGTCTTG	AAGGTAGCTC	ATCTAAACTT	GAAATAAAAC
CP007690.1	AATAATTATA	CAAACCCACC	GTTAACGAAC	CCTATT-TTA	GAAGGTCTTG	AAGGTAGCTC	ATCTAAACTT	GAAATAAAAC
CP007176.1	AATAATTATA	CAAACCCACC	GTTAACGAAC	CCTATT-TTA	GAAGGTCTTG	AAGGTAGCTC	ATCTAAACTT	GAAATAAAAC
CP000730.1	AATAATTATA	CAAACCCACC	GTTAACGAAC	CCTATT-TTA	GAAGGTCTTG	AAGGTAGCTC	ATCTAAACTT	GAAATAAAAC
CP000255.1	AATAATTATA	CAAACCCACC	GTTAACGAAC	CCTATT-TTA	GAAGGTCTTG	AAGGTAGCTC	ATCTAAACTT	GAAATAAAAC
CP007539.1	AATAATTATA	CAAACCCACC	GTTAACGAAC	CCTATT-TTA	GAAGGTCTTG	AAGGTAGCTC	ATCTAAACTT	GAAATAAAAC
CP007657.1	AATAATTATA	CAAACCCACC	GTTAACGAAC	CCTATT-TTA	GAAGGTCTTG	AAGGTAGCTC	ATCTAAACTT	GAAATAAAAC
AP014921.1	AATAATTATA	CAAACCCACC	GTTAACGAAC	CCTATT-TTA	GAAGGTCTTG	AAGGTAGCTC	ATCTAAACTT	GAAATAAAAC
AB436955.1	AATAATTATA	CAAACCCACC	GTTAACGAAC	CCTATT-TTA	GAAGGTCTTG	AAGGTAGCTC	ATCTAAACTT	GAAATAAAAC
AB489885.1	AATAATTATA	CAAACCCACC	GTTAACGAAC	CCTATT-TTA	GAAGGTCTTG	AAGGTAGCTC	ATCTAAACTT	GAAATAAAAC
AB489873.1	AATAATTATA	CAAACCCACC	GTTAACGAAC	CCTATT-TTA	GAAGGTCTTG	AAGGTAGCTC	ATCTAAACTT	GAAATAAAAC
AB489883.1	AATAATTATA	CAAACCCACC	GTTAACGAAC	CCTATT-TTA	GAAGGTCTTG	AAGGTAGCTC	ATCTAAACTT	GAAATAAAAC
AB489874.1	AATAATTATA	CAAACCCACC	GTTAACGAAC	CCTATT-TTA	GAAGGTCTTG	AAGGTAGCTC	ATCTAAACTT	GAAATAAAAC
LT615218.1	AATAATTATA	CAAACCCACC	GTTAACGAAC	CCTATT-TTA	GAAGGTCTTG	AAGGTAGCTC	ATCTAAACTT	GAAATAAAAC
AB436976.1	AATAATTATA	CAAACCCACC	GTTAACGAAC	CCTATT-TTA	GAAGGTCTTG	AAGGTAGCTC	ATCTAAACTT	GAAATAAAAC
AB489892.1	AATAATTATA	CAAACCCACC	GTTAACGAAC	CCTATT-TTA	GAAGGTCTTG	AAGGTAGCTC	ATCTAAACTT	GAAATAAAAC
AJ306908.1	GATAATTATA	CTCAACCGAC	GACACCGAAC	CCTATT-TTA	GAAGGTCTTG	AAAGTAGCTC	ATCTAAACTT	GAAATAAAAC
CP015646.1	GATAATTATA	CTCAACCGAC	GACACCGAAC	CCTATT-TTA	GAAGGTCTTG	AAAGTAGCTC	ATCTAAACTT	GAAATAAAAC
AB488510.1	AATAATTATA	CAAACCCACC	GTTAACGAAC	CCTATT-TTA	GAAGGTCTTG	AAGGTAGCTC	ATCTAAACTT	GAAATAAAAC
EU105387.1	AATAATTATA	CAAACCCACC	GTTAACGAAC	CCTATT-TTA	GAAGGTCTTG	AAGGTAGCTC	ATCTAAACTT	GAAATAAAAC
AB436975.1	GATAATTATA	CTCAACCGAC	GACACCGAAC	CCTATT-TTA	AAAGGTATTG	AAGGAAACTC	ATCTAAACTT	GAAATAAAAC
AB488499.1	GATAATTATA	CTCAACCGAC	GACACCGAAC	CCTATT-TTA	AAAGGTATTG	AAGGAAACTC	ATCTAAACTT	GAAATAAAAC
AJ309189.1	AATAATTATA	CAAACCCACC	GTTAACGAAC	CCTATT-TTA	GAAGGTCTTG	AAGGTAGCTC	ATCTAAACTT	GAAATAAAAC
FR821779.1	GATAATTATA	CTCAACCGAC	GACACCGAAC	CCTATT-TTA	GAAGGTCTTG	AAGGTAGCTC	ATCTAAACTT	GAAATAAAAC
CP012593.1	GATAATTATA	CTCAACCGAC	GACACCGAAC	CCTATT-TTA	GAAGGTCTTG	AAGGTAGCTC	ATCTAAACTT	GAAATAAAAC
CP012692.1	GATAATTATA	CTCAACCGAC	GACACCGAAC	CCTATT-TTA	GAAGGTCTTG	AAGGTAGCTC	ATCTAAACTT	GAAATAAAAC
CP013955.1	GATAATTATA	CTCAACCGAC	GACACCGAAC	CCTATT-TTA	GAAGGTCTTG	AAGGTAGCTC	ATCTAAACTT	GAAATAAAAC
CP013953.1	GATAATTATA	CTCAACCGAC	GACACCGAAC	CCTATT-TTA	GAAGGTCTTG	AAGGTAGCTC	ATCTAAACTT	GAAATAAAAC
CP014064.1	GATAATTATA	CTCAACCGAC	GACACCGAAC	CCTATT-TTA	GAAGGTCTTG	AAGGTAGCTC	ATCTAAACTT	GAAATAAAAC
LT009690.1	GATAATTATA	CTCAACCGAC	GACACCGAAC	CCTATT-TTA	GAAGGTCTTG	AAGGTAGCTC	ATCTAAACTT	GAAATAAAAC
AP017320.1	GATAATTATA	CTCAACCGAC	GACACCGAAC	CCTATT-TTA	GAAGGTCTTG	AAGGTAGCTC	ATCTAAACTT	GAAATAAAAC
CP010890.1	GATAATTATA	CTCAACCGAC	GACACCGAAC	CCTATT-TTA	GAAGGTCTTG	AAGGTAGCTC	ATCTAAACTT	GAAATAAAAC
CP001844.2	GATAATTATA	CTCAACCGAC	GACACCGAAC	CCTATT-TTA	GAAGGTCTTG	AAGGTAGCTC	ATCTAAACTT	GAAATAAAAC
CP001781.1	GATAATTATA	CTCAACCGAC	GACACCGAAC	CCTATT-TTA	GAAGGTCTTG	AAGGTAGCTC	ATCTAAACTT	GAAATAAAAC
AB488501.1	GATAATTATA	CTCAACCGAC	GACACCGAAC	CCTATT-TTA	GAAGGTCTTG	AAGGTAGCTC	ATCTAAACTT	GAAATAAAAC
AB489898.1	GATAATTATA	CTCAACCGAC	GACACCGAAC	CCTATT-TTA	GAAGGTCTTG	AAGGTAGCTC	ATCTAAACTT	GAAATAAAAC
HE579073.1	GATAATTATA	CTCAACCGAC	GACACCGAAC	CCTATT-TTA	GAAGGTCTTG	AAGGTAGCTC	ATCTAAACTT	GAAATAAAAC
HE579071.1	GATAATTATA	CTCAACCGAC	GACACCGAAC	CCTATT-TTA	GAAGGTCTTG	AAGGTAGCTC	ATCTAAACTT	GAAATAAAAC
HE579069.1	GATAATTATA	CTCAACCGAC	GACACCGAAC	CCTATT-TTA	GAAGGTCTTG	AAGGTAGCTC	ATCTAAACTT	GAAATAAAAC
HE579065.1	GATAATTATA	CTCAACCGAC	GACACCGAAC	CCTATT-TTA	GAAGGTCTTG	AAGGTAGCTC	ATCTAAACTT	GAAATAAAAC
HE579063.1	GATAATTATA	CTCAACCGAC	GACACCGAAC	CCTATT-TTA	GAAGGTCTTG	AAGGTAGCTC	ATCTAAACTT	GAAATAAAAC

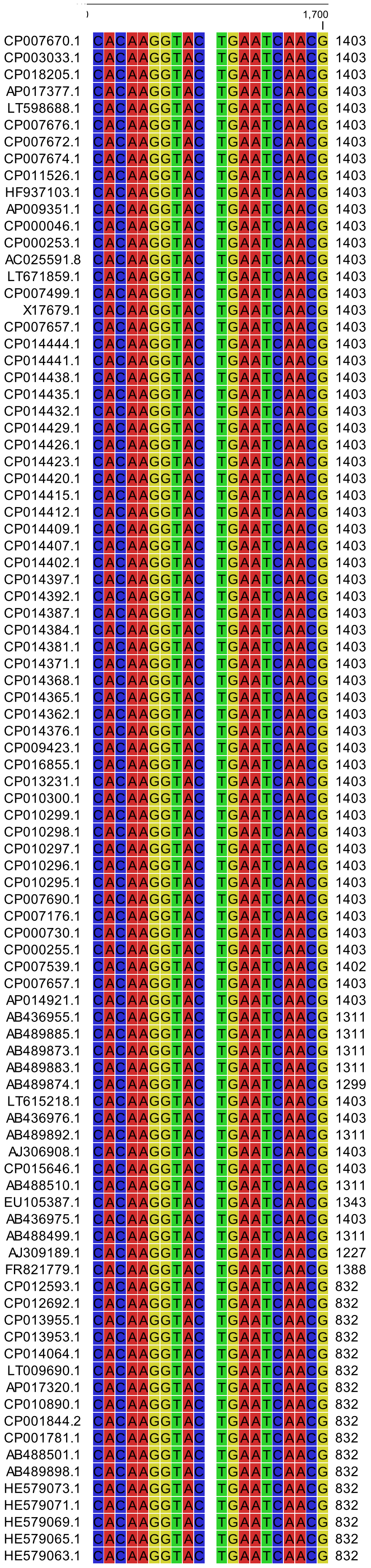
1,700
CP007670.1 CACAAGGTAC TGAATCAACG 1403
CP003033.1 CACAAGGTAC TGAATCAACG 1403
CP018205.1 CACAAGGTAC TGAATCAACG 1403
AP017377.1 CACAAGGTAC TGAATCAACG 1403
LT598688.1 CACAAGGTAC TGAATCAACG 1403
CP007676.1 CACAAGGTAC TGAATCAACG 1403
CP007672.1 CACAAGGTAC TGAATCAACG 1403
CP007674.1 CACAAGGTAC TGAATCAACG 1403
CP011526.1 CACAAGGTAC TGAATCAACG 1403
HF937103.1 CACAAGGTAC TGAATCAACG 1403
AP009351.1 CACAAGGTAC TGAATCAACG 1403
CP000046.1 CACAAGGTAC TGAATCAACG 1403
CP000253.1 CACAAGGTAC TGAATCAACG 1403
AC025591.8 CACAAGGTAC TGAATCAACG 1403
LT671859.1 CACAAGGTAC TGAATCAACG 1403
CP007499.1 CACAAGGTAC TGAATCAACG 1403
X17679.1 CACAAGGTAC TGAATCAACG 1403
CP007657.1 CACAAGGTAC TGAATCAACG 1403
CP014444.1 CACAAGGTAC TGAATCAACG 1403
CP014441.1 CACAAGGTAC TGAATCAACG 1403
CP014438.1 CACAAGGTAC TGAATCAACG 1403
CP014435.1 CACAAGGTAC TGAATCAACG 1403
CP014432.1 CACAAGGTAC TGAATCAACG 1403
CP014429.1 CACAAGGTAC TGAATCAACG 1403
CP014426.1 CACAAGGTAC TGAATCAACG 1403
CP014423.1 CACAAGGTAC TGAATCAACG 1403
CP014420.1 CACAAGGTAC TGAATCAACG 1403
CP014415.1 CACAAGGTAC TGAATCAACG 1403
CP014412.1 CACAAGGTAC TGAATCAACG 1403
CP014409.1 CACAAGGTAC TGAATCAACG 1403
CP014407.1 CACAAGGTAC TGAATCAACG 1403
CP014402.1 CACAAGGTAC TGAATCAACG 1403
CP014397.1 CACAAGGTAC TGAATCAACG 1403
CP014392.1 CACAAGGTAC TGAATCAACG 1403
CP014387.1 CACAAGGTAC TGAATCAACG 1403
CP014384.1 CACAAGGTAC TGAATCAACG 1403
CP014381.1 CACAAGGTAC TGAATCAACG 1403
CP014371.1 CACAAGGTAC TGAATCAACG 1403
CP014368.1 CACAAGGTAC TGAATCAACG 1403
CP014365.1 CACAAGGTAC TGAATCAACG 1403
CP014362.1 CACAAGGTAC TGAATCAACG 1403
CP014376.1 CACAAGGTAC TGAATCAACG 1403
CP009423.1 CACAAGGTAC TGAATCAACG 1403
CP016855.1 CACAAGGTAC TGAATCAACG 1403
CP013231.1 CACAAGGTAC TGAATCAACG 1403
CP010300.1 CACAAGGTAC TGAATCAACG 1403
CP010299.1 CACAAGGTAC TGAATCAACG 1403
CP010298.1 CACAAGGTAC TGAATCAACG 1403
CP010297.1 CACAAGGTAC TGAATCAACG 1403
CP010296.1 CACAAGGTAC TGAATCAACG 1403
CP010295.1 CACAAGGTAC TGAATCAACG 1403
CP007690.1 CACAAGGTAC TGAATCAACG 1403
CP007176.1 CACAAGGTAC TGAATCAACG 1403
CP000730.1 CACAAGGTAC TGAATCAACG 1403
CP000255.1 CACAAGGTAC TGAATCAACG 1403
CP007539.1 CACAAGGTAC TGAATCAACG 1402
CP007657.1 CACAAGGTAC TGAATCAACG 1403
AP014921.1 CACAAGGTAC TGAATCAACG 1403
AB436955.1 CACAAGGTAC TGAATCAACG 1311
AB489885.1 CACAAGGTAC TGAATCAACG 1311
AB489873.1 CACAAGGTAC TGAATCAACG 1311
AB489883.1 CACAAGGTAC TGAATCAACG 1311
AB489874.1 CACAAGGTAC TGAATCAACG 1299
LT615218.1 CACAAGGTAC TGAATCAACG 1403
AB436976.1 CACAAGGTAC TGAATCAACG 1403
AB489892.1 CACAAGGTAC TGAATCAACG 1311
AJ306908.1 CACAAGGTAC TGAATCAACG 1403
CP015646.1 CACAAGGTAC TGAATCAACG 1403
AB488510.1 CACAAGGTAC TGAATCAACG 1311
EU105387.1 CACAAGGTAC TGAATCAACG 1343
AB436975.1 CACAAGGTAC TGAATCAACG 1403
AB488499.1 CACAAGGTAC TGAATCAACG 1311
AJ309189.1 CACAAGGTAC TGAATCAACG 1227
FR821779.1 CACAAGGTAC TGAATCAACG 1388
CP012593.1 CACAAGGTAC TGAATCAACG 832
CP012692.1 CACAAGGTAC TGAATCAACG 832
CP013955.1 CACAAGGTAC TGAATCAACG 832
CP013953.1 CACAAGGTAC TGAATCAACG 832
CP014064.1 CACAAGGTAC TGAATCAACG 832
LT009690.1 CACAAGGTAC TGAATCAACG 832
AP017320.1 CACAAGGTAC TGAATCAACG 832
CP010890.1 CACAAGGTAC TGAATCAACG 832
CP001844.2 CACAAGGTAC TGAATCAACG 832
CP001781.1 CACAAGGTAC TGAATCAACG 832
AB488501.1 CACAAGGTAC TGAATCAACG 832
AB489898.1 CACAAGGTAC TGAATCAACG 832
HE579073.1 CACAAGGTAC TGAATCAACG 832
HE579071.1 CACAAGGTAC TGAATCAACG 832
HE579069.1 CACAAGGTAC TGAATCAACG 832
HE579065.1 CACAAGGTAC TGAATCAACG 832
HE579063.1 CACAAGGTAC TGAATCAACG 832

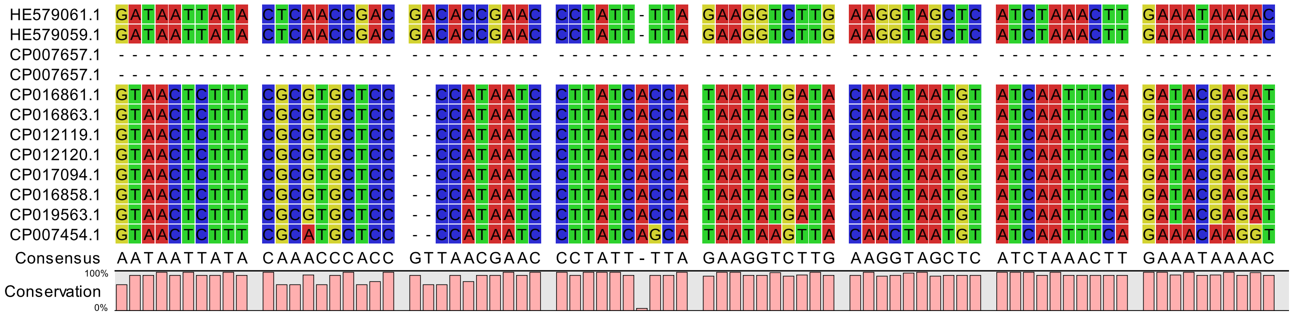
HE579061.1 GATAATTATA CTCAACCGAC GACACCGAAC CCTATT-TTA GAAGGTCTTG AAGGTAGCTC ATCTAAACTT GAAATAAAAC
HE579059.1 GATAATTATA CTCAACCGAC GACACCGAAC CCTATT-TTA GAAGGTCTTG AAGGTAGCTC ATCTAAACTT GAAATAAAAC
CP007657.1 ---------- ---------- ---------- ---------- ---------- ---------- ---------- ----------
CP007657.1 ---------- ---------- ---------- ---------- ---------- ---------- ---------- ----------
CP016861.1 GTAACTCTTT CGCGTGCTCC --CCATAATC CTTATCACCA TAATATGATA CAACTAATGT ATCAATTTCA GATACGAGAT
CP016863.1 GTAACTCTTT CGCGTGCTCC --CCATAATC CTTATCACCA TAATATGATA CAACTAATGT ATCAATTTCA GATACGAGAT
CP012119.1 GTAACTCTTT CGCGTGCTCC --CCATAATC CTTATCACCA TAATATGATA CAACTAATGT ATCAATTTCA GATACGAGAT
CP012120.1 GTAACTCTTT CGCGTGCTCC --CCATAATC CTTATCACCA TAATATGATA CAACTAATGT ATCAATTTCA GATACGAGAT
CP017094.1 GTAACTCTTT CGCGTGCTCC --CCATAATC CTTATCACCA TAATATGATA CAACTAATGT ATCAATTTCA GATACGAGAT
CP016858.1 GTAACTCTTT CGCGTGCTCC --CCATAATC CTTATCACCA TAATATGATA CAACTAATGT ATCAATTTCA GATACGAGAT
CP019563.1 GTAACTCTTT CGCGTGCTCC --CCATAATC CTTATCACCA TAATATGATA CAACTAATGT ATCAATTTCA GATACGAGAT
CP007454.1 GTAACTCTTT CGCATGCTCC --CCATAATC CTTATCAGCA TAATAAGTTA CAACTAATGT ATCAATTTCA GAAACAAGGT
Consensus AATAATTATA CAAACCCACC GTTAACGAAC CCTATT-TTA GAAGGTCTTG AAGGTAGCTC ATCTAAACTT GAAATAAAAC
100%
Conservation
0%

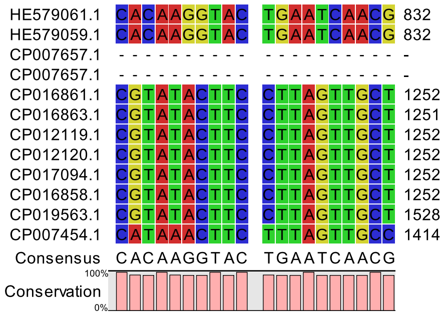
HE579061.1 CACAAGGTAC TGAATCAACG 832
HE579059.1 CACAAGGTAC TGAATCAACG 832
CP007657.1 -
CP007657.1 -
CP016861.1 CGTATACTTC CTTAGTTGCT 1252
CP016863.1 CGTATACTTC CTTAGTTGCT 1251
CP012119.1 CGTATACTTC CTTAGTTGCT 1252
CP012120.1 CGTATACTTC CTTAGTTGCT 1252
CP017094.1 CGTATACTTC CTTAGTTGCT 1252
CP016858.1 CGTATACTTC CTTAGTTGCT 1252
CP019563.1 CGTATACTTC CTTAGTTGCT 1528
CP007454.1 CATAAACTTC TTTAGTTGCC 1414
Consensus CACAAGGTAC TGAATCAACG
Conservation
100%
0%

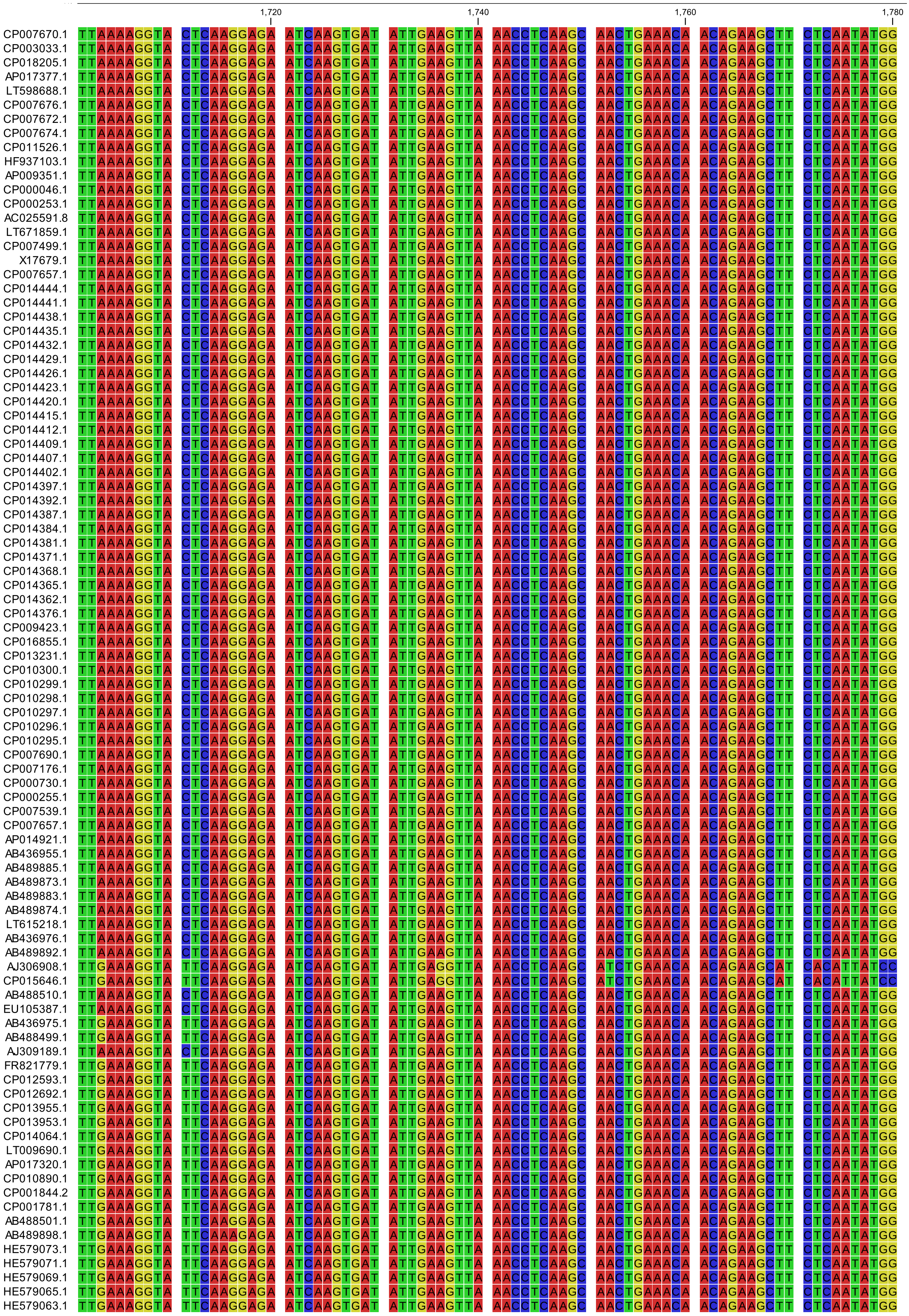
1,720
1,740
1,760
1,780
CP007670.1 TTAAAAGGTA CTCAAGGAGA ATCAAGTGAT ATTGAAGTTA AACCTCAAGC AACTGAAACA ACAGAAGCTT CTCAATATGG
CP003033.1 TTAAAAGGTA CTCAAGGAGA ATCAAGTGAT ATTGAAGTTA AACCTCAAGC AACTGAAACA ACAGAAGCTT CTCAATATGG
CP018205.1 TTAAAAGGTA CTCAAGGAGA ATCAAGTGAT ATTGAAGTTA AACCTCAAGC AACTGAAACA ACAGAAGCTT CTCAATATGG
AP017377.1 TTAAAAGGTA CTCAAGGAGA ATCAAGTGAT ATTGAAGTTA AACCTCAAGC AACTGAAACA ACAGAAGCTT CTCAATATGG
LT598688.1 TTAAAAGGTA CTCAAGGAGA ATCAAGTGAT ATTGAAGTTA AACCTCAAGC AACTGAAACA ACAGAAGCTT CTCAATATGG
CP007676.1 TTAAAAGGTA CTCAAGGAGA ATCAAGTGAT ATTGAAGTTA AACCTCAAGC AACTGAAACA ACAGAAGCTT CTCAATATGG
CP007672.1 TTAAAAGGTA CTCAAGGAGA ATCAAGTGAT ATTGAAGTTA AACCTCAAGC AACTGAAACA ACAGAAGCTT CTCAATATGG
CP007674.1 TTAAAAGGTA CTCAAGGAGA ATCAAGTGAT ATTGAAGTTA AACCTCAAGC AACTGAAACA ACAGAAGCTT CTCAATATGG
CP011526.1 TTAAAAGGTA CTCAAGGAGA ATCAAGTGAT ATTGAAGTTA AACCTCAAGC AACTGAAACA ACAGAAGCTT CTCAATATGG
HF937103.1 TTAAAAGGTA CTCAAGGAGA ATCAAGTGAT ATTGAAGTTA AACCTCAAGC AACTGAAACA ACAGAAGCTT CTCAATATGG
AP009351.1 TTAAAAGGTA CTCAAGGAGA ATCAAGTGAT ATTGAAGTTA AACCTCAAGC AACTGAAACA ACAGAAGCTT CTCAATATGG
CP000046.1 TTAAAAGGTA CTCAAGGAGA ATCAAGTGAT ATTGAAGTTA AACCTCAAGC AACTGAAACA ACAGAAGCTT CTCAATATGG
CP000253.1 TTAAAAGGTA CTCAAGGAGA ATCAAGTGAT ATTGAAGTTA AACCTCAAGC AACTGAAACA ACAGAAGCTT CTCAATATGG
AC025591.8 TTAAAAGGTA CTCAAGGAGA ATCAAGTGAT ATTGAAGTTA AACCTCAAGC AACTGAAACA ACAGAAGCTT CTCAATATGG
LT671859.1 TTAAAAGGTA CTCAAGGAGA ATCAAGTGAT ATTGAAGTTA AACCTCAAGC AACTGAAACA ACAGAAGCTT CTCAATATGG
CP007499.1 TTAAAAGGTA CTCAAGGAGA ATCAAGTGAT ATTGAAGTTA AACCTCAAGC AACTGAAACA ACAGAAGCTT CTCAATATGG
X17679.1 TTAAAAGGTA CTCAAGGAGA ATCAAGTGAT ATTGAAGTTA AACCTCAAGC AACTGAAACA ACAGAAGCTT CTCAATATGG
CP007657.1 TTAAAAGGTA CTCAAGGAGA ATCAAGTGAT ATTGAAGTTA AACCTCAAGC AACTGAAACA ACAGAAGCTT CTCAATATGG
CP014444.1 TTAAAAGGTA CTCAAGGAGA ATCAAGTGAT ATTGAAGTTA AACCTCAAGC AACTGAAACA ACAGAAGCTT CTCAATATGG
CP014441.1 TTAAAAGGTA CTCAAGGAGA ATCAAGTGAT ATTGAAGTTA AACCTCAAGC AACTGAAACA ACAGAAGCTT CTCAATATGG
CP014438.1 TTAAAAGGTA CTCAAGGAGA ATCAAGTGAT ATTGAAGTTA AACCTCAAGC AACTGAAACA ACAGAAGCTT CTCAATATGG
CP014435.1 TTAAAAGGTA CTCAAGGAGA ATCAAGTGAT ATTGAAGTTA AACCTCAAGC AACTGAAACA ACAGAAGCTT CTCAATATGG
CP014432.1 TTAAAAGGTA CTCAAGGAGA ATCAAGTGAT ATTGAAGTTA AACCTCAAGC AACTGAAACA ACAGAAGCTT CTCAATATGG
CP014429.1 TTAAAAGGTA CTCAAGGAGA ATCAAGTGAT ATTGAAGTTA AACCTCAAGC AACTGAAACA ACAGAAGCTT CTCAATATGG
CP014426.1 TTAAAAGGTA CTCAAGGAGA ATCAAGTGAT ATTGAAGTTA AACCTCAAGC AACTGAAACA ACAGAAGCTT CTCAATATGG
CP014423.1 TTAAAAGGTA CTCAAGGAGA ATCAAGTGAT ATTGAAGTTA AACCTCAAGC AACTGAAACA ACAGAAGCTT CTCAATATGG
CP014420.1 TTAAAAGGTA CTCAAGGAGA ATCAAGTGAT ATTGAAGTTA AACCTCAAGC AACTGAAACA ACAGAAGCTT CTCAATATGG
CP014415.1 TTAAAAGGTA CTCAAGGAGA ATCAAGTGAT ATTGAAGTTA AACCTCAAGC AACTGAAACA ACAGAAGCTT CTCAATATGG
CP014412.1 TTAAAAGGTA CTCAAGGAGA ATCAAGTGAT ATTGAAGTTA AACCTCAAGC AACTGAAACA ACAGAAGCTT CTCAATATGG
CP014409.1 TTAAAAGGTA CTCAAGGAGA ATCAAGTGAT ATTGAAGTTA AACCTCAAGC AACTGAAACA ACAGAAGCTT CTCAATATGG
CP014407.1 TTAAAAGGTA CTCAAGGAGA ATCAAGTGAT ATTGAAGTTA AACCTCAAGC AACTGAAACA ACAGAAGCTT CTCAATATGG
CP014402.1 TTAAAAGGTA CTCAAGGAGA ATCAAGTGAT ATTGAAGTTA AACCTCAAGC AACTGAAACA ACAGAAGCTT CTCAATATGG
CP014397.1 TTAAAAGGTA CTCAAGGAGA ATCAAGTGAT ATTGAAGTTA AACCTCAAGC AACTGAAACA ACAGAAGCTT CTCAATATGG
CP014392.1 TTAAAAGGTA CTCAAGGAGA ATCAAGTGAT ATTGAAGTTA AACCTCAAGC AACTGAAACA ACAGAAGCTT CTCAATATGG
CP014387.1 TTAAAAGGTA CTCAAGGAGA ATCAAGTGAT ATTGAAGTTA AACCTCAAGC AACTGAAACA ACAGAAGCTT CTCAATATGG
CP014384.1 TTAAAAGGTA CTCAAGGAGA ATCAAGTGAT ATTGAAGTTA AACCTCAAGC AACTGAAACA ACAGAAGCTT CTCAATATGG
CP014381.1 TTAAAAGGTA CTCAAGGAGA ATCAAGTGAT ATTGAAGTTA AACCTCAAGC AACTGAAACA ACAGAAGCTT CTCAATATGG
CP014371.1 TTAAAAGGTA CTCAAGGAGA ATCAAGTGAT ATTGAAGTTA AACCTCAAGC AACTGAAACA ACAGAAGCTT CTCAATATGG
CP014368.1 TTAAAAGGTA CTCAAGGAGA ATCAAGTGAT ATTGAAGTTA AACCTCAAGC AACTGAAACA ACAGAAGCTT CTCAATATGG
CP014365.1 TTAAAAGGTA CTCAAGGAGA ATCAAGTGAT ATTGAAGTTA AACCTCAAGC AACTGAAACA ACAGAAGCTT CTCAATATGG
CP014362.1 TTAAAAGGTA CTCAAGGAGA ATCAAGTGAT ATTGAAGTTA AACCTCAAGC AACTGAAACA ACAGAAGCTT CTCAATATGG
CP014376.1 TTAAAAGGTA CTCAAGGAGA ATCAAGTGAT ATTGAAGTTA AACCTCAAGC AACTGAAACA ACAGAAGCTT CTCAATATGG
CP009423.1 TTAAAAGGTA CTCAAGGAGA ATCAAGTGAT ATTGAAGTTA AACCTCAAGC AACTGAAACA ACAGAAGCTT CTCAATATGG
CP016855.1 TTAAAAGGTA CTCAAGGAGA ATCAAGTGAT ATTGAAGTTA AACCTCAAGC AACTGAAACA ACAGAAGCTT CTCAATATGG
CP013231.1 TTAAAAGGTA CTCAAGGAGA ATCAAGTGAT ATTGAAGTTA AACCTCAAGC AACTGAAACA ACAGAAGCTT CTCAATATGG
CP010300.1 TTAAAAGGTA CTCAAGGAGA ATCAAGTGAT ATTGAAGTTA AACCTCAAGC AACTGAAACA ACAGAAGCTT CTCAATATGG
CP010299.1 TTAAAAGGTA CTCAAGGAGA ATCAAGTGAT ATTGAAGTTA AACCTCAAGC AACTGAAACA ACAGAAGCTT CTCAATATGG
CP010298.1 TTAAAAGGTA CTCAAGGAGA ATCAAGTGAT ATTGAAGTTA AACCTCAAGC AACTGAAACA ACAGAAGCTT CTCAATATGG
CP010297.1 TTAAAAGGTA CTCAAGGAGA ATCAAGTGAT ATTGAAGTTA AACCTCAAGC AACTGAAACA ACAGAAGCTT CTCAATATGG
CP010296.1 TTAAAAGGTA CTCAAGGAGA ATCAAGTGAT ATTGAAGTTA AACCTCAAGC AACTGAAACA ACAGAAGCTT CTCAATATGG
CP010295.1 TTAAAAGGTA CTCAAGGAGA ATCAAGTGAT ATTGAAGTTA AACCTCAAGC AACTGAAACA ACAGAAGCTT CTCAATATGG
CP007690.1 TTAAAAGGTA CTCAAGGAGA ATCAAGTGAT ATTGAAGTTA AACCTCAAGC AACTGAAACA ACAGAAGCTT CTCAATATGG
CP007176.1 TTAAAAGGTA CTCAAGGAGA ATCAAGTGAT ATTGAAGTTA AACCTCAAGC AACTGAAACA ACAGAAGCTT CTCAATATGG
CP000730.1 TTAAAAGGTA CTCAAGGAGA ATCAAGTGAT ATTGAAGTTA AACCTCAAGC AACTGAAACA ACAGAAGCTT CTCAATATGG
CP000255.1 TTAAAAGGTA CTCAAGGAGA ATCAAGTGAT ATTGAAGTTA AACCTCAAGC AACTGAAACA ACAGAAGCTT CTCAATATGG
CP007539.1 TTAAAAGGTA CTCAAGGAGA ATCAAGTGAT ATTGAAGTTA AACCTCAAGC AACTGAAACA ACAGAAGCTT CTCAATATGG
CP007657.1 TTAAAAGGTA CTCAAGGAGA ATCAAGTGAT ATTGAAGTTA AACCTCAAGC AACTGAAACA ACAGAAGCTT CTCAATATGG
AP014921.1 TTAAAAGGTA CTCAAGGAGA ATCAAGTGAT ATTGAAGTTA AACCTCAAGC AACTGAAACA ACAGAAGCTT CTCAATATGG
AB436955.1 TTAAAAGGTA CTCAAGGAGA ATCAAGTGAT ATTGAAGTTA AACCTCAAGC AACTGAAACA ACAGAAGCTT CTCAATATGG
AB489885.1 TTAAAAGGTA CTCAAGGAGA ATCAAGTGAT ATTGAAGTTA AACCTCAAGC AACTGAAACA ACAGAAGCTT CTCAATATGG
AB489873.1 TTAAAAGGTA CTCAAGGAGA ATCAAGTGAT ATTGAAGTTA AACCTCAAGC AACTGAAACA ACAGAAGCTT CTCAATATGG
AB489883.1 TTAAAAGGTA CTCAAGGAGA ATCAAGTGAT ATTGAAGTTA AACCTCAAGC AACTGAAACA ACAGAAGCTT CTCAATATGG
AB489874.1 TTAAAAGGTA CTCAAGGAGA ATCAAGTGAT ATTGAAGTTA AACCTCAAGC AACTGAAACA ACAGAAGCTT CTCAATATGG
LT615218.1 TTAAAAGGTA CTCAAGGAGA ATCAAGTGAT ATTGAAGTTA AACCTCAAGC AACTGAAACA ACAGAAGCTT CTCAATATGG
AB436976.1 TTAAAAGGTA CTCAAGGAGA ATCAAGTGAT ATTGAAGTTA AACCTCAAGC AACTGAAACA ACAGAAGCTT CTCAATATGG
AB489892.1 TTAAAAGGTA CTCAAGGAGA ATCAAGTGAT ATTGAAGTTA AACCTCAAGC AACTGAAACA ACAGAAGCTT CTCAATATGG
AJ306908.1 TTGAAAGGTA TTCAAGGAGA ATCAAGTGAT ATTGAGGTTA AACCTCAAGC ATCTGAAACA ACAGAAGCAT CACATTATCC
CP015646.1 TTGAAAGGTA TTCAAGGAGA ATCAAGTGAT ATTGAGGTTA AACCTCAAGC ATCTGAAACA ACAGAAGCAT CACATTATCC
AB488510.1 TTAAAAGGTA CTCAAGGAGA ATCAAGTGAT ATTGAAGTTA AACCTCAAGC AACTGAAACA ACAGAAGCTT CTCAATATGG
EU105387.1 TTAAAAGGTA CTCAAGGAGA ATCAAGTGAT ATTGAAGTTA AACCTCAAGC AACTGAAACA ACAGAAGCTT CTCAATATGG
AB436975.1 TTGAAAGGTA TTCAAGGAGA ATCAAGTGAT ATTGAAGTTA AACCTCAAGC AACTGAAACA ACAGAAGCTT CTCAATATGG
AB488499.1 TTGAAAGGTA TTCAAGGAGA ATCAAGTGAT ATTGAAGTTA AACCTCAAGC AACTGAAACA ACAGAAGCTT CTCAATATGG
AJ309189.1 TTAAAAGGTA CTCAAGGAGA ATCAAGTGAT ATTGAAGTTA AACCTCAAGC AACTGAAACA ACAGAAGCTT CTCAATATGG
FR821779.1 TTGAAAGGTA TTCAAGGAGA ATCAAGTGAT ATTGAAGTTA AACCTCAAGC AACTGAAACA ACAGAAGCTT CTCAATATGG
CP012593.1 TTGAAAGGTA TTCAAGGAGA ATCAAGTGAT ATTGAAGTTA AACCTCAAGC AACTGAAACA ACAGAAGCTT CTCAATATGG
CP012692.1 TTGAAAGGTA TTCAAGGAGA ATCAAGTGAT ATTGAAGTTA AACCTCAAGC AACTGAAACA ACAGAAGCTT CTCAATATGG
CP013955.1 TTGAAAGGTA TTCAAGGAGA ATCAAGTGAT ATTGAAGTTA AACCTCAAGC AACTGAAACA ACAGAAGCTT CTCAATATGG
CP013953.1 TTGAAAGGTA TTCAAGGAGA ATCAAGTGAT ATTGAAGTTA AACCTCAAGC AACTGAAACA ACAGAAGCTT CTCAATATGG
CP014064.1 TTGAAAGGTA TTCAAGGAGA ATCAAGTGAT ATTGAAGTTA AACCTCAAGC AACTGAAACA ACAGAAGCTT CTCAATATGG
LT009690.1 TTGAAAGGTA TTCAAGGAGA ATCAAGTGAT ATTGAAGTTA AACCTCAAGC AACTGAAACA ACAGAAGCTT CTCAATATGG
AP017320.1 TTGAAAGGTA TTCAAGGAGA ATCAAGTGAT ATTGAAGTTA AACCTCAAGC AACTGAAACA ACAGAAGCTT CTCAATATGG
CP010890.1 TTGAAAGGTA TTCAAGGAGA ATCAAGTGAT ATTGAAGTTA AACCTCAAGC AACTGAAACA ACAGAAGCTT CTCAATATGG
CP001844.2 TTGAAAGGTA TTCAAGGAGA ATCAAGTGAT ATTGAAGTTA AACCTCAAGC AACTGAAACA ACAGAAGCTT CTCAATATGG
CP001781.1 TTGAAAGGTA TTCAAGGAGA ATCAAGTGAT ATTGAAGTTA AACCTCAAGC AACTGAAACA ACAGAAGCTT CTCAATATGG
AB488501.1 TTGAAAGGTA TTCAAGGAGA ATCAAGTGAT ATTGAAGTTA AACCTCAAGC AACTGAAACA ACAGAAGCTT CTCAATATGG
AB489898.1 TTGAAAGGTA TTCAAAGAGA ATCAAGTGAT ATTGAAGTTA AACCTCAAGC AACTGAAACA ACAGAAGCTT CTCAATATGG
HE579073.1 TTGAAAGGTA TTCAAGGAGA ATCAAGTGAT ATTGAAGTTA AACCTCAAGC AACTGAAACA ACAGAAGCTT CTCAATATGG
HE579071.1 TTGAAAGGTA TTCAAGGAGA ATCAAGTGAT ATTGAAGTTA AACCTCAAGC AACTGAAACA ACAGAAGCTT CTCAATATGG
HE579069.1 TTGAAAGGTA TTCAAGGAGA ATCAAGTGAT ATTGAAGTTA AACCTCAAGC AACTGAAACA ACAGAAGCTT CTCAATATGG
HE579065.1 TTGAAAGGTA TTCAAGGAGA ATCAAGTGAT ATTGAAGTTA AACCTCAAGC AACTGAAACA ACAGAAGCTT CTCAATATGG
HE579063.1 TTGAAAGGTA TTCAAGGAGA ATCAAGTGAT ATTGAAGTTA AACCTCAAGC AACTGAAACA ACAGAAGCTT CTCAATATGG

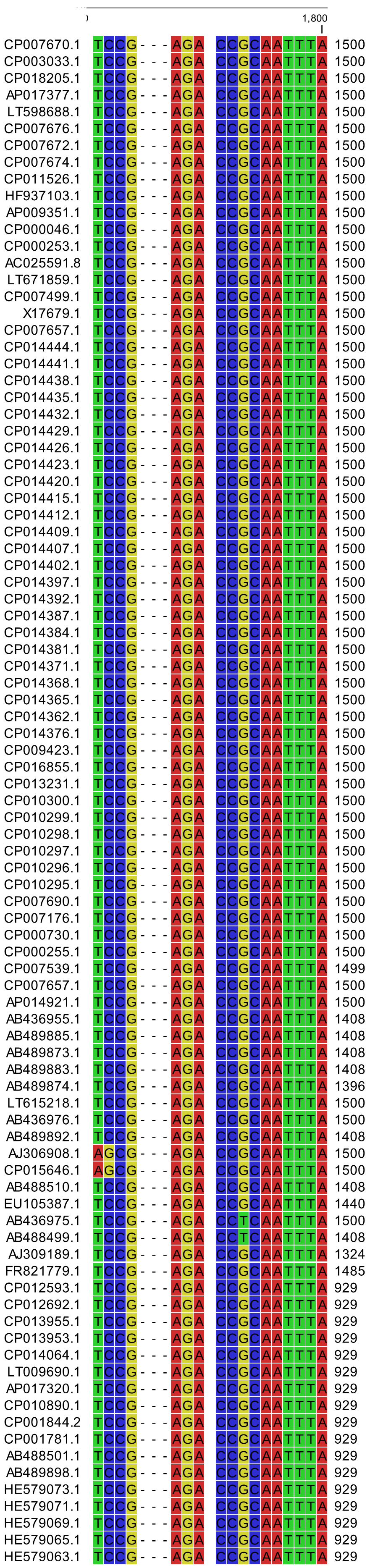
1,800
CP007670.1 TCCG---AGA CCGCAATTTA 1500
CP003033.1 TCCG---AGA CCGCAATTTA 1500
CP018205.1 TCCG---AGA CCGCAATTTA 1500
AP017377.1 TCCG---AGA CCGCAATTTA 1500
LT598688.1 TCCG---AGA CCGCAATTTA 1500
CP007676.1 TCCG---AGA CCGCAATTTA 1500
CP007672.1 TCCG---AGA CCGCAATTTA 1500
CP007674.1 TCCG---AGA CCGCAATTTA 1500
CP011526.1 TCCG---AGA CCGCAATTTA 1500
HF937103.1 TCCG---AGA CCGCAATTTA 1500
AP009351.1 TCCG---AGA CCGCAATTTA 1500
CP000046.1 TCCG---AGA CCGCAATTTA 1500
CP000253.1 TCCG---AGA CCGCAATTTA 1500
AC025591.8 TCCG---AGA CCGCAATTTA 1500
LT671859.1 TCCG---AGA CCGCAATTTA 1500
CP007499.1 TCCG---AGA CCGCAATTTA 1500
X17679.1 TCCG---AGA CCGCAATTTA 1500
CP007657.1 TCCG---AGA CCGCAATTTA 1500
CP014444.1 TCCG---AGA CCGCAATTTA 1500
CP014441.1 TCCG---AGA CCGCAATTTA 1500
CP014438.1 TCCG---AGA CCGCAATTTA 1500
CP014435.1 TCCG---AGA CCGCAATTTA 1500
CP014432.1 TCCG---AGA CCGCAATTTA 1500
CP014429.1 TCCG---AGA CCGCAATTTA 1500
CP014426.1 TCCG---AGA CCGCAATTTA 1500
CP014423.1 TCCG---AGA CCGCAATTTA 1500
CP014420.1 TCCG---AGA CCGCAATTTA 1500
CP014415.1 TCCG---AGA CCGCAATTTA 1500
CP014412.1 TCCG---AGA CCGCAATTTA 1500
CP014409.1 TCCG---AGA CCGCAATTTA 1500
CP014407.1 TCCG---AGA CCGCAATTTA 1500
CP014402.1 TCCG---AGA CCGCAATTTA 1500
CP014397.1 TCCG---AGA CCGCAATTTA 1500
CP014392.1 TCCG---AGA CCGCAATTTA 1500
CP014387.1 TCCG---AGA CCGCAATTTA 1500
CP014384.1 TCCG---AGA CCGCAATTTA 1500
CP014381.1 TCCG---AGA CCGCAATTTA 1500
CP014371.1 TCCG---AGA CCGCAATTTA 1500
CP014368.1 TCCG---AGA CCGCAATTTA 1500
CP014365.1 TCCG---AGA CCGCAATTTA 1500
CP014362.1 TCCG---AGA CCGCAATTTA 1500
CP014376.1 TCCG---AGA CCGCAATTTA 1500
CP009423.1 TCCG---AGA CCGCAATTTA 1500
CP016855.1 TCCG---AGA CCGCAATTTA 1500
CP013231.1 TCCG---AGA CCGCAATTTA 1500
CP010300.1 TCCG---AGA CCGCAATTTA 1500
CP010299.1 TCCG---AGA CCGCAATTTA 1500
CP010298.1 TCCG---AGA CCGCAATTTA 1500
CP010297.1 TCCG---AGA CCGCAATTTA 1500
CP010296.1 TCCG---AGA CCGCAATTTA 1500
CP010295.1 TCCG---AGA CCGCAATTTA 1500
CP007690.1 TCCG---AGA CCGCAATTTA 1500
CP007176.1 TCCG---AGA CCGCAATTTA 1500
CP000730.1 TCCG---AGA CCGCAATTTA 1500
CP000255.1 TCCG---AGA CCGCAATTTA 1500
CP007539.1 TCCG---AGA CCGCAATTTA 1499
CP007657.1 TCCG---AGA CCGCAATTTA 1500
AP014921.1 TCCG---AGA CCGCAATTTA 1500
AB436955.1 TCCG---AGA CCGCAATTTA 1408
AB489885.1 TCCG---AGA CCGCAATTTA 1408
AB489873.1 TCCG---AGA CCGCAATTTA 1408
AB489883.1 TCCG---AGA CCGCAATTTA 1408
AB489874.1 TCCG---AGA CCGCAATTTA 1396
LT615218.1 TCCG---AGA CCGCAATTTA 1500
AB436976.1 TCCG---AGA CCGCAATTTA 1500
AB489892.1 TCCG---AGA CCGCAATTTA 1408
AJ306908.1 AGCG---AGA CCGCAATTTA 1500
CP015646.1 AGCG---AGA CCGCAATTTA 1500
AB488510.1 TCCG---AGA CCGCAATTTA 1408
EU105387.1 TCCG---AGA CCGCAATTTA 1440
AB436975.1 TCCG---AGA CCTCAATTTA 1500
AB488499.1 TCCG---AGA CCTCAATTTA 1408
AJ309189.1 TCCG---AGA CCGCAATTTA 1324
FR821779.1 TCCG---AGA CCGCAATTTA 1485
CP012593.1 TCCG---AGA CCGCAATTTA 929
CP012692.1 TCCG---AGA CCGCAATTTA 929
CP013955.1 TCCG---AGA CCGCAATTTA 929
CP013953.1 TCCG---AGA CCGCAATTTA 929
CP014064.1 TCCG---AGA CCGCAATTTA 929
LT009690.1 TCCG---AGA CCGCAATTTA 929
AP017320.1 TCCG---AGA CCGCAATTTA 929
CP010890.1 TCCG---AGA CCGCAATTTA 929
CP001844.2 TCCG---AGA CCGCAATTTA 929
CP001781.1 TCCG---AGA CCGCAATTTA 929
AB488501.1 TCCG---AGA CCGCAATTTA 929
AB489898.1 TCCG---AGA CCGCAATTTA 929
HE579073.1 TCCG---AGA CCGCAATTTA 929
HE579071.1 TCCG---AGA CCGCAATTTA 929
HE579069.1 TCCG---AGA CCGCAATTTA 929
HE579065.1 TCCG---AGA CCGCAATTTA 929
HE579063.1 TCCG---AGA CCGCAATTTA 929

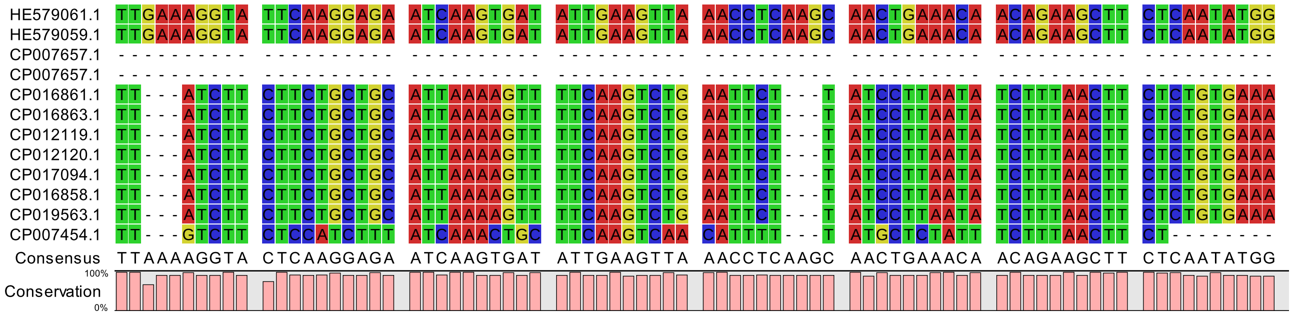
HE579061.1 TTGAAAGGTA TTCAAGGAGA ATCAAGTGAT ATTGAAGTTA AACCTCAAGC AACTGAAACA ACAGAAGCTT CTCAATATGG
HE579059.1 TTGAAAGGTA TTCAAGGAGA ATCAAGTGAT ATTGAAGTTA AACCTCAAGC AACTGAAACA ACAGAAGCTT CTCAATATGG
CP007657.1 ---------- ---------- ---------- ---------- ---------- ---------- ---------- ----------
CP007657.1 ---------- ---------- ---------- ---------- ---------- ---------- ---------- ----------
CP016861.1 TT---ATCTT CTTCTGCTGC ATTAAAAGTT TTCAAGTCTG AATTCT---T ATCCTTAATA TCTTTAACTT CTCTGTGAAA
CP016863.1 TT---ATCTT CTTCTGCTGC ATTAAAAGTT TTCAAGTCTG AATTCT---T ATCCTTAATA TCTTTAACTT CTCTGTGAAA
CP012119.1 TT---ATCTT CTTCTGCTGC ATTAAAAGTT TTCAAGTCTG AATTCT---T ATCCTTAATA TCTTTAACTT CTCTGTGAAA
CP012120.1 TT---ATCTT CTTCTGCTGC ATTAAAAGTT TTCAAGTCTG AATTCT---T ATCCTTAATA TCTTTAACTT CTCTGTGAAA
CP017094.1 TT---ATCTT CTTCTGCTGC ATTAAAAGTT TTCAAGTCTG AATTCT---T ATCCTTAATA TCTTTAACTT CTCTGTGAAA
CP016858.1 TT---ATCTT CTTCTGCTGC ATTAAAAGTT TTCAAGTCTG AATTCT---T ATCCTTAATA TCTTTAACTT CTCTGTGAAA
CP019563.1 TT---ATCTT CTTCTGCTGC ATTAAAAGTT TTCAAGTCTG AATTCT---T ATCCTTAATA TCTTTAACTT CTCTGTGAAA
CP007454.1 TT---GTCTT CTCCATCTTT ATCAAACTGC TTCAAGTCAA CATTTT---T ATGCTCTATT TCTTTAACTT CT--------
Consensus TTAAAAGGTA CTCAAGGAGA ATCAAGTGAT ATTGAAGTTA AACCTCAAGC AACTGAAACA ACAGAAGCTT CTCAATATGG
100%
Conservation
0%

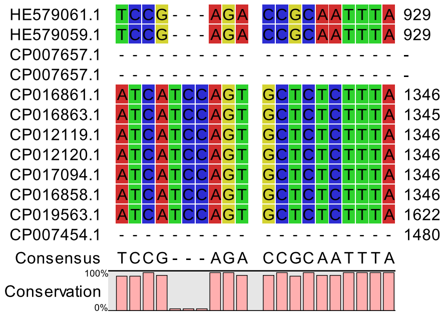
HE579061.1 TCCG---AGA CCGCAATTTA 929
HE579059.1 TCCG---AGA CCGCAATTTA 929
CP007657.1 ---------- ---------- -
CP007657.1 ---------- ---------- -
CP016861.1 ATCATCCAGT GCTCTCTTTA 1346
CP016863.1 ATCATCCAGT GCTCTCTTTA 1345
CP012119.1 ATCATCCAGT GCTCTCTTTA 1346
CP012120.1 ATCATCCAGT GCTCTCTTTA 1346
CP017094.1 ATCATCCAGT GCTCTCTTTA 1346
CP016858.1 ATCATCCAGT GCTCTCTTTA 1346
CP019563.1 ATCATCCAGT GCTCTCTTTA 1622
CP007454.1 ---------- ---------- 1480
Consensus TCCG---AGA CCGCAATTTA
100%
Conservation
0%

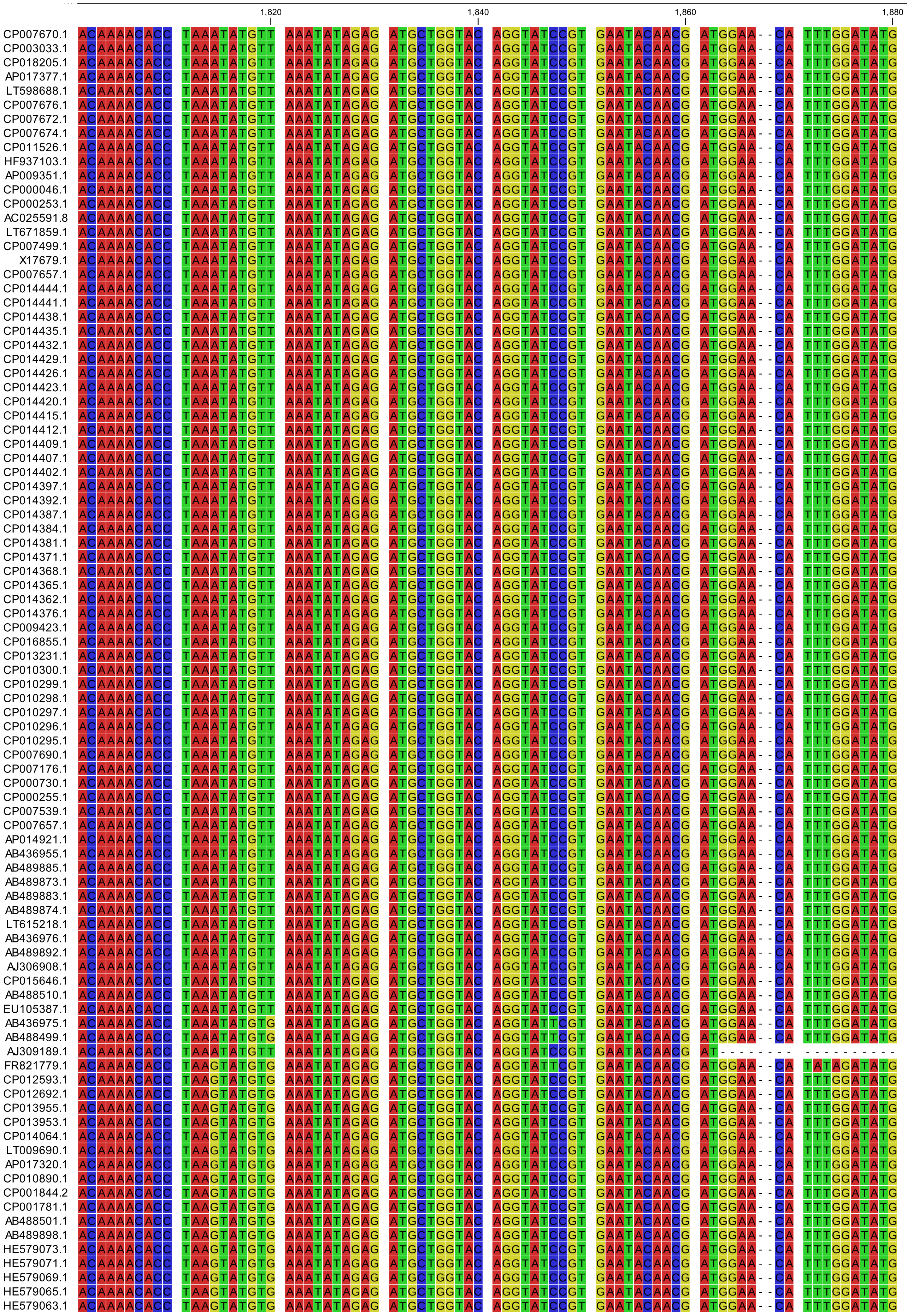
1,820
1,840
1,860
1,880
CP007670.1
CP003033.1
CP018205.1
AP017377.1
LT598688.1
CP007676.1
CP007672.1
CP007674.1
CP011526.1
HF937103.1
AP009351.1
CP000046.1
CP000253.1
AC025591.8
LT671859.1
CP007499.1
X17679.1
CP007657.1
CP014444.1
CP014441.1
CP014438.1
CP014435.1
CP014432.1
CP014429.1
CP014426.1
CP014423.1
CP014420.1
CP014415.1
CP014412.1
CP014409.1
CP014407.1
CP014402.1
CP014397.1
CP014392.1
CP014387.1
CP014384.1
CP014381.1
CP014371.1
CP014368.1
CP014365.1
CP014362.1
CP014376.1
CP009423.1
CP016855.1
CP013231.1
CP010300.1
CP010299.1
CP010298.1
CP010297.1
CP010296.1
CP010295.1
CP007690.1
CP007176.1
CP000730.1
CP000255.1
CP007539.1
CP007657.1
AP014921.1
AB436955.1
AB489885.1
AB489873.1
AB489883.1
AB489874.1
LT615218.1
AB436976.1
AB489892.1
AJ306908.1
CP015646.1
AB488510.1
EU105387.1
AB436975.1
AB488499.1
AJ309189.1
FR821779.1
CP012593.1
CP012692.1
CP013955.1
CP013953.1
CP014064.1
LT009690.1
AP017320.1
CP010890.1
CP001844.2
CP001781.1
AB488501.1
AB489898.1
HE579073.1
HE579071.1
HE579069.1
HE579065.1
HE579063.1

1,900

CP007670.1	AAGCGAGACC	AAGATTCAAT	1598
CP003033.1	AAGCGAGACC	AAGATTCAAT	1598
CP018205.1	AAGCGAGACC	AAGATTCAAT	1598
AP017377.1	AAGCGAGACC	AAGATTCAAT	1598
LT598688.1	AAGCGAGACC	AAGATTCAAT	1598
CP007676.1	AAGCGAGACC	AAGATTCAAT	1598
CP007672.1	AAGCGAGACC	AAGATTCAAT	1598
CP007674.1	AAGCGAGACC	AAGATTCAAT	1598
CP011526.1	AAGCGAGACC	AAGATTCAAT	1598
HF937103.1	AAGCGAGACC	AAGATTCAAT	1598
AP009351.1	AAGCGAGACC	AAGATTCAAT	1598
CP000046.1	AAGCGAGACC	AAGATTCAAT	1598
CP000253.1	AAGCGAGACC	AAGATTCAAT	1598
AC025591.8	AAGCGAGACC	AAGATTCAAT	1598
LT671859.1	AAGCGAGACC	AAGATTCAAT	1598
CP007499.1	AAGCGAGACC	AAGATTCAAT	1598
X17679.1	AAGCGAGACC	AAGATTCAAT	1598
CP007657.1	AAGCGAGACC	AAGATTCAAT	1598
CP014444.1	AAGCGAGACC	AAGATTCAAT	1598
CP014441.1	AAGCGAGACC	AAGATTCAAT	1598
CP014438.1	AAGCGAGACC	AAGATTCAAT	1598
CP014435.1	AAGCGAGACC	AAGATTCAAT	1598
CP014432.1	AAGCGAGACC	AAGATTCAAT	1598
CP014429.1	AAGCGAGACC	AAGATTCAAT	1598
CP014426.1	AAGCGAGACC	AAGATTCAAT	1598
CP014423.1	AAGCGAGACC	AAGATTCAAT	1598
CP014420.1	AAGCGAGACC	AAGATTCAAT	1598
CP014415.1	AAGCGAGACC	AAGATTCAAT	1598
CP014412.1	AAGCGAGACC	AAGATTCAAT	1598
CP014409.1	AAGCGAGACC	AAGATTCAAT	1598
CP014407.1	AAGCGAGACC	AAGATTCAAT	1598
CP014402.1	AAGCGAGACC	AAGATTCAAT	1598
CP014397.1	AAGCGAGACC	AAGATTCAAT	1598
CP014392.1	AAGCGAGACC	AAGATTCAAT	1598
CP014387.1	AAGCGAGACC	AAGATTCAAT	1598
CP014384.1	AAGCGAGACC	AAGATTCAAT	1598
CP014381.1	AAGCGAGACC	AAGATTCAAT	1598
CP014371.1	AAGCGAGACC	AAGATTCAAT	1598
CP014368.1	AAGCGAGACC	AAGATTCAAT	1598
CP014365.1	AAGCGAGACC	AAGATTCAAT	1598
CP014362.1	AAGCGAGACC	AAGATTCAAT	1598
CP014376.1	AAGCGAGACC	AAGATTCAAT	1598
CP009423.1	AAGCGAGACC	AAGATTCAAT	1598
CP016855.1	AAGCGAGACC	AAGATTCAAT	1598
CP013231.1	AAGCGAGACC	AAGATTCAAT	1598
CP010300.1	AAGCGAGACC	AAGATTCAAT	1598
CP010299.1	AAGCGAGACC	AAGATTCAAT	1598
CP010298.1	AAGCGAGACC	AAGATTCAAT	1598
CP010297.1	AAGCGAGACC	AAGATTCAAT	1598
CP010296.1	AAGCGAGACC	AAGATTCAAT	1598
CP010295.1	AAGCGAGACC	AAGATTCAAT	1598
CP007690.1	AAGCGAGACC	AAGATTCAAT	1598
CP007176.1	AAGCGAGACC	AAGATTCAAT	1598
CP000730.1	AAGCGAGACC	AAGATTCAAT	1598
CP000255.1	AAGCGAGACC	AAGATTCAAT	1598
CP007539.1	AAGCGAGACC	AAGATTCAAT	1597
CP007657.1	AAGCGAGACC	AAGATTCAAT	1598
AP014921.1	AAGCGAGACC	AAGATTCAAT	1598
AB436955.1	AAGCGAGACC	AAGATTCAAT	1506
AB489885.1	AAGCGAGACC	AAGATTCAAT	1506
AB489873.1	AAGCGAGACC	AAGATTCAAT	1506
AB489883.1	AAGCGAGACC	AAGATTCAAT	1506
AB489874.1	AAGCGAGACC	AAGATTCAAT	1494
LT615218.1	AAGCGAGACC	AAGATTCAAT	1598
AB436976.1	AAGCGAGACC	AAGATTCAAT	1598
AB489892.1	AAGCGAGACC	AAGATTCAAT	1506
AJ306908.1	AAGCGAGACC	AAGATTCAAT	1598
CP015646.1	AAGCGAGACC	AAGATTCAAT	1598
AB488510.1	AAGCGAGACC	AAGATTCAAT	1506
EU105387.1	AA--------	----------	1520
AB436975.1	AAGCGAGACC	AAGATTCAAT	1598
AB488499.1	AAGCGAGACC	AAGATTCAAT	1506
AJ309189.1	----------	----------	1386
FR821779.1	AAGCGAGACC	AAGATTCAAC	1583
CP012593.1	AAGCGAGACC	AAGATTCAAC	1027
CP012692.1	AAGCGAGACC	AAGATTCAAC	1027
CP013955.1	AAGCGAGACC	AAGATTCAAC	1027
CP013953.1	AAGCGAGACC	AAGATTCAAC	1027
CP014064.1	AAGCGAGACC	AAGATTCAAC	1027
LT009690.1	AAGCGAGACC	AAGATTCAAC	1027
AP017320.1	AAGCGAGACC	AAGATTCAAC	1027
CP010890.1	AAGCGAGACC	AAGATTCAAC	1027
CP001844.2	AAGCGAGACC	AAGATTCAAC	1027
CP001781.1	AAGCGAGACC	AAGATTCAAC	1027
AB488501.1	AAGCGAGACC	AAGATTCAAC	1027
AB489898.1	AAGCGAGACC	AAGATTCAAC	1027
HE579073.1	AAGCGAGACC	AAGATTCAAC	1027
HE579071.1	AAGCGAGACC	AAGATTCAAC	1027
HE579069.1	AAGCGAGACC	AAGATTCAAC	1027
HE579065.1	AAGCGAGACC	AAGATTCAAC	1027
HE579063.1	AAGCGAGACC	AAGATTCAAC	1027

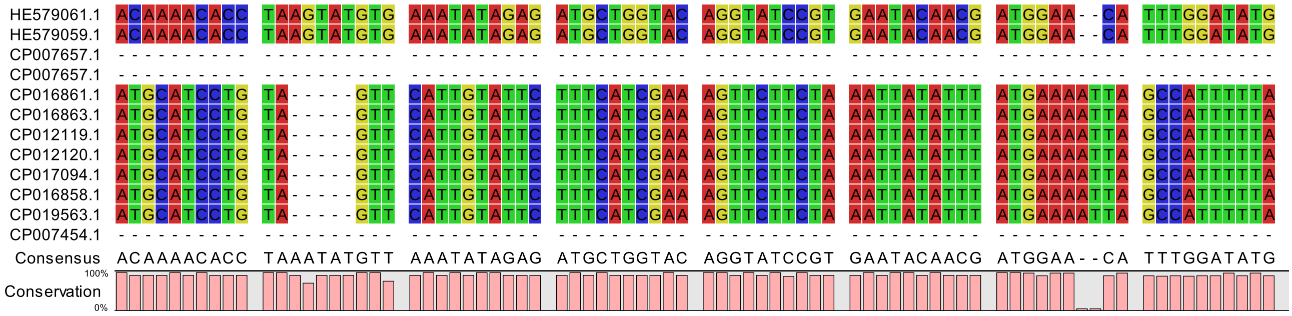
HE579061.1 ACAAAACACC TAAGTATGTG AAATATAGAG ATGCTGGTAC AGGTATCCGT GAATACAACG ATGGAA--CA TTTGGATATG
HE579059.1 ACAAAACACC TAAGTATGTG AAATATAGAG ATGCTGGTAC AGGTATCCGT GAATACAACG ATGGAA--CA TTTGGATATG
CP007657.1 ---------- ---------- ---------- ---------- ---------- ---------- ---------- ----------
CP007657.1 ---------- ---------- ---------- ---------- ---------- ---------- ---------- ----------
CP016861.1 ATGCATCCTG TA-----GTT CATTGTATTC TTTCATCGAA AGTTCTTCTA AATTATATTT ATGAAAATTA GCCATTTTTA
CP016863.1 ATGCATCCTG TA-----GTT CATTGTATTC TTTCATCGAA AGTTCTTCTA AATTATATTT ATGAAAATTA GCCATTTTTA
CP012119.1 ATGCATCCTG TA-----GTT CATTGTATTC TTTCATCGAA AGTTCTTCTA AATTATATTT ATGAAAATTA GCCATTTTTA
CP012120.1 ATGCATCCTG TA-----GTT CATTGTATTC TTTCATCGAA AGTTCTTCTA AATTATATTT ATGAAAATTA GCCATTTTTA
CP017094.1 ATGCATCCTG TA-----GTT CATTGTATTC TTTCATCGAA AGTTCTTCTA AATTATATTT ATGAAAATTA GCCATTTTTA
CP016858.1 ATGCATCCTG TA-----GTT CATTGTATTC TTTCATCGAA AGTTCTTCTA AATTATATTT ATGAAAATTA GCCATTTTTA
CP019563.1 ATGCATCCTG TA-----GTT CATTGTATTC TTTCATCGAA AGTTCTTCTA AATTATATTT ATGAAAATTA GCCATTTTTA
CP007454.1 ---------- ---------- ---------- ---------- ---------- ---------- ---------- ----------
Consensus ACAAAACACC TAAATATGTT AAATATAGAG ATGCTGGTAC AGGTATCCGT GAATACAACG ATGGAA--CA TTTGGATATG
100%
Conservation
0%

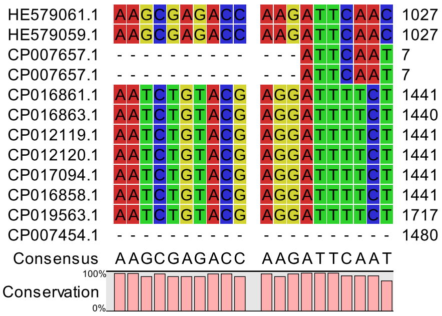
HE579061.1 AAGCGAGACC AAGATTCAAC 1027
HE579059.1 AAGCGAGACC AAGATTCAAC 1027
CP007657.1 - - - - - - - - - - - - - ATTCAAT 7
CP007657.1 - - - - - - - - - - - - - ATTCAAT 7
CP016861.1 AATCTGTACG AGGATTTTCT 1441
CP016863.1 AATCTGTACG AGGATTTTCT 1440
CP012119.1 AATCTGTACG AGGATTTTCT 1441
CP012120.1 AATCTGTACG AGGATTTTCT 1441
CP017094.1 AATCTGTACG AGGATTTTCT 1441
CP016858.1 AATCTGTACG AGGATTTTCT 1441
CP019563.1 AATCTGTACG AGGATTTTCT 1717
CP007454.1 - 1480
Consensus AAGCGAGACC AAGATTCAAT
100%
Conservation
0%

```
CP007670.1   AAGCCATCAG AAACAAATGC ATA------- ---------- ---------- ---------- ---------- ----------
CP003033.1   AAGCCATCAG AAACAAATGC ATA------- ---------- ---------- ---------- ---------- ----------
CP018205.1   AAGCCATCAG AAACAAATGC ATA------- ---------- ---------- ---------- ---------- ----------
AP017377.1   AAGCCATCAG AAACAAATGC ATA------- ---------- ---------- ---------- ---------- ----------
LT598688.1   AAGCCATCAG AAACAAATGC ATA------- ---------- ---------- ---------- ---------- ----------
CP007676.1   AAGCCATCAG AAACAAATGC ATA------- ---------- ---------- ---------- ---------- ----------
CP007672.1   AAGCCATCAG AAACAAATGC ATA------- ---------- ---------- ---------- ---------- ----------
CP007674.1   AAGCCATCAG AAACAAATGC ATA------- ---------- ---------- ---------- ---------- ----------
CP011526.1   AAGCCATCAG AAACAAATGC ATA------- ---------- ---------- ---------- ---------- ----------
HF937103.1   AAGCCATCAG AAACAAATGC ATA------- ---------- ---------- ---------- ---------- ----------
AP009351.1   AAGCCATCAG AAACAAATGC ATA------- ---------- ---------- ---------- ---------- ----------
CP000046.1   AAGCCATCAG AAACAAATGC ATA------- ---------- ---------- ---------- ---------- ----------
CP000253.1   AAGCCATCAG AAACAAATGC ATA------- ---------- ---------- ---------- ---------- ----------
AC025591.8   AAGCCATCAG AAACAAATGC ATA------- ---------- ---------- ---------- ---------- ----------
LT671859.1   AAGCCATCAG AAACAAATGC ATA------- ---------- ---------- ---------- ---------- ----------
CP007499.1   AAGCCATCAG AAACAAATGC ATA------- ---------- ---------- ---------- ---------- ----------
X17679.1     AAGCCATCAG AAACAAATGC ATA------- ---------- ---------- ---------- ---------- ----------
CP007657.1   AAGCCATCAG AAACAAATGC ATA------- ---------- ---------- ---------- ---------- ----------
CP014444.1   AAGCCATCAG AAACAAATGC ATA------- ---------- ---------- ---------- ---------- ----------
CP014441.1   AAGCCATCAG AAACAAATGC ATA------- ---------- ---------- ---------- ---------- ----------
CP014438.1   AAGCCATCAG AAACAAATGC ATA------- ---------- ---------- ---------- ---------- ----------
CP014435.1   AAGCCATCAG AAACAAATGC ATA------- ---------- ---------- ---------- ---------- ----------
CP014432.1   AAGCCATCAG AAACAAATGC ATA------- ---------- ---------- ---------- ---------- ----------
CP014429.1   AAGCCATCAG AAACAAATGC ATA------- ---------- ---------- ---------- ---------- ----------
CP014426.1   AAGCCATCAG AAACAAATGC ATA------- ---------- ---------- ---------- ---------- ----------
CP014423.1   AAGCCATCAG AAACAAATGC ATA------- ---------- ---------- ---------- ---------- ----------
CP014420.1   AAGCCATCAG AAACAAATGC ATA------- ---------- ---------- ---------- ---------- ----------
CP014415.1   AAGCCATCAG AAACAAATGC ATA------- ---------- ---------- ---------- ---------- ----------
CP014412.1   AAGCCATCAG AAACAAATGC ATA------- ---------- ---------- ---------- ---------- ----------
CP014409.1   AAGCCATCAG AAACAAATGC ATA------- ---------- ---------- ---------- ---------- ----------
CP014407.1   AAGCCATCAG AAACAAATGC ATA------- ---------- ---------- ---------- ---------- ----------
CP014402.1   AAGCCATCAG AAACAAATGC ATA------- ---------- ---------- ---------- ---------- ----------
CP014397.1   AAGCCATCAG AAACAAATGC ATA------- ---------- ---------- ---------- ---------- ----------
CP014392.1   AAGCCATCAG AAACAAATGC ATA------- ---------- ---------- ---------- ---------- ----------
CP014387.1   AAGCCATCAG AAACAAATGC ATA------- ---------- ---------- ---------- ---------- ----------
CP014384.1   AAGCCATCAG AAACAAATGC ATA------- ---------- ---------- ---------- ---------- ----------
CP014381.1   AAGCCATCAG AAACAAATGC ATA------- ---------- ---------- ---------- ---------- ----------
CP014371.1   AAGCCATCAG AAACAAATGC ATA------- ---------- ---------- ---------- ---------- ----------
CP014368.1   AAGCCATCAG AAACAAATGC ATA------- ---------- ---------- ---------- ---------- ----------
CP014365.1   AAGCCATCAG AAACAAATGC ATA------- ---------- ---------- ---------- ---------- ----------
CP014362.1   AAGCCATCAG AAACAAATGC ATA------- ---------- ---------- ---------- ---------- ----------
CP014376.1   AAGCCATCAG AAACAAATGC ATA------- ---------- ---------- ---------- ---------- ----------
CP009423.1   AAGCCATCAG AAACAAATGC ATA------- ---------- ---------- ---------- ---------- ----------
CP016855.1   AAGCCATCAG AAACAAATGC ATA------- ---------- ---------- ---------- ---------- ----------
CP013231.1   AAGCCATCAG AAACAAATGC ATA------- ---------- ---------- ---------- ---------- ----------
CP010300.1   AAGCCATCAG AAACAAATGC ATA------- ---------- ---------- ---------- ---------- ----------
CP010299.1   AAGCCATCAG AAACAAATGC ATA------- ---------- ---------- ---------- ---------- ----------
CP010298.1   AAGCCATCAG AAACAAATGC ATA------- ---------- ---------- ---------- ---------- ----------
CP010297.1   AAGCCATCAG AAACAAATGC ATA------- ---------- ---------- ---------- ---------- ----------
CP010296.1   AAGCCATCAG AAACAAATGC ATA------- ---------- ---------- ---------- ---------- ----------
CP010295.1   AAGCCATCAG AAACAAATGC ATA------- ---------- ---------- ---------- ---------- ----------
CP007690.1   AAGCCATCAG AAACAAATGC ATA------- ---------- ---------- ---------- ---------- ----------
CP007176.1   AAGCCATCAG AAACAAATGC ATA------- ---------- ---------- ---------- ---------- ----------
CP000730.1   AAGCCATCAG AAACAAATGC ATA------- ---------- ---------- ---------- ---------- ----------
CP000255.1   AAGCCATCAG AAACAAATGC ATA------- ---------- ---------- ---------- ---------- ----------
CP007539.1   AAGCCATCAG AAACAAATGC ATA------- ---------- ---------- ---------- ---------- ----------
CP007657.1   AAGCCATCAG AAACAAATGC ATA------- ---------- ---------- ---------- ---------- ----------
AP014921.1   AAGCCATCAG AAACAAATGC ATA------- ---------- ---------- ---------- ---------- ----------
AB436955.1   AAGCCATCAG AAACAAATGC ATA------- ---------- ---------- ---------- ---------- ----------
AB489885.1   AAGCCATCAG AAACAAATGC ATA------- ---------- ---------- ---------- ---------- ----------
AB489873.1   AAGCCATCAG AAACAAATGC ATA------- ---------- ---------- ---------- ---------- ----------
AB489883.1   AAGCCATCAG AAACAAATGC ATA------- ---------- ---------- ---------- ---------- ----------
AB489874.1   AAGCCATCAG AAACAAATGC ATA------- ---------- ---------- ---------- ---------- ----------
LT615218.1   AAGCCATCAG AAACAAACGC ATACAACGTA ACGACAAATC AAGATGGCAC AGTAACATAT GGCGCTCGTC CAACACAAAA
AB436976.1   AAGCCATCAG AAACAAACGC ATACAACGTA ACGACAAATC AAGATGGCAC AGTAACATAT GGCGCTCGTC CAACACAAAA
AB489892.1   AAGCCATCAG AAACAAACGC ATACAACGTA ACGACAAATC AAGATGGCAC AGTAACATAT GGCGCTCGTC CAACACAAAA
AJ306908.1   AAGCCATCAG AAACAAACGC ATACAACGTA ACGACAAATC AAGATGGCAC AGTAACATAT GGCGCTCGTC CAACACAAAA
CP015646.1   AAGCCATCAG AAACAAACGC ATACAACGTA ACGACAAATC AAGATGGCAC AGTAACATAT GGCGCTCGTC CAACACAAAA
AB488510.1   AAGCCATCAG AAACAAATGC ATA------- ---------- ---------- ---------- ---------- ----------
EU105387.1   ---------- ---------- ---------- ---------- ---------- ---------- ---------- ----------
AB436975.1   AAGCCATCAG AAACAAACGC ATACAACGTA ACGACAAACC AAGATGGCAC AGTATCATAC GGCGCTCGCC CGACATACAA
AB488499.1   AAGCCATCAG AAACAAACGC ATACAACGTA ACGACAAACC AAGATGGCAC AGTATCATAC GGCGCTCGCC CGACATACAA
AJ309189.1   ---------- ---------- ---------- ---------- ---------- ---------- ---------- ----------
FR821779.1   AAGCCAAGTG AAACAAACGC ATACAACGTA ACGACAAATC AAGATGGCAC AGTATCATAT GGGGCTCGCC CGACACAAAA
CP012593.1   AAGCCAAGTG AAACAAATGC ATACAACGTA ACGACAAATC AAGATGGCAC AGTATCATAC GGAGCTCGCC CAACACAAAA
CP012692.1   AAGCCAAGTG AAACAAATGC ATACAACGTA ACGACAAATC AAGATGGCAC AGTATCATAC GGAGCTCGCC CAACACAAAA
CP013955.1   AAGCCAAGTG AAACAAATGC ATACAACGTA ACGACAAATC AAGATGGCAC AGTATCATAC GGAGCTCGCC CAACACAAAA
CP013953.1   AAGCCAAGTG AAACAAATGC ATACAACGTA ACGACAAATC AAGATGGCAC AGTATCATAC GGAGCTCGCC CAACACAAAA
CP014064.1   AAGCCAAGTG AAACAAATGC ATACAACGTA ACGACAAATC AAGATGGCAC AGTATCATAC GGAGCTCGCC CAACACAAAA
LT009690.1   AAGCCAAGTG AAACAAATGC ATACAACGTA ACGACAAATC AAGATGGCAC AGTATCATAC GGAGCTCGCC CAACACAAAA
AP017320.1   AAGCCAAGTG AAACAAATGC ATACAACGTA ACGACAAATC AAGATGGCAC AGTATCATAC GGAGCTCGCC CAACACAAAA
CP010890.1   AAGCCAAGTG AAACAAATGC ATACAACGTA ACGACAAATC AAGATGGCAC AGTATCATAC GGAGCTCGCC CAACACAAAA
CP001844.2   AAGCCAAGTG AAACAAATGC ATACAACGTA ACGACAAATC AAGATGGCAC AGTATCATAC GGAGCTCGCC CAACACAAAA
CP001781.1   AAGCCAAGTG AAACAAATGC ATACAACGTA ACGACAAATC AAGATGGCAC AGTATCATAC GGAGCTCGCC CAACACAAAA
AB488501.1   AAGCCAAGTG AAACAAATGC ATACAACGTA ACGACAAATC AAGATGGCAC AGTATCATAC GGAGCTCGCC CAACACAAAA
AB489898.1   AAGCCAAGTG AAACAAATGC ATACAACGTA ACGACAAATC AAGATGGCAC AGTATCATAC GGAGCTCGCC CAACACAAAA
HE579073.1   AAGCCAAGTG AAACAAATGC ATACAACGTA ACGACAAATC AAGATGGCAC AGTATCATAC GGAGCTCGCC CAACACAAAA
HE579071.1   AAGCCAAGTG AAACAAATGC ATACAACGTA ACGACAAATC AAGATGGCAC AGTATCATAC GGAGCTCGCC CAACACAAAA
HE579069.1   AAGCCAAGTG AAACAAATGC ATACAACGTA ACGACAAATC AAGATGGCAC AGTATCATAC GGAGCTCGCC CAACACAAAA
HE579065.1   AAGCCAAGTG AAACAAATGC ATACAACGTA ACGACAAATC AAGATGGCAC AGTATCATAC GGAGCTCGCC CAACACAAAA
HE579063.1   AAGCCAAGTG AAACAAATGC ATACAACGTA ACGACAAATC AAGATGGCAC AGTATCATAC GGAGCTCGCC CAACACAAAA
```

		2,000	
CP007670.1	----------	----------	1621
CP003033.1	----------	----------	1621
CP018205.1	----------	----------	1621
AP017377.1	----------	----------	1621
LT598688.1	----------	----------	1621
CP007676.1	----------	----------	1621
CP007672.1	----------	----------	1621
CP007674.1	----------	----------	1621
CP011526.1	----------	----------	1621
HF937103.1	----------	----------	1621
AP009351.1	----------	----------	1621
CP000046.1	----------	----------	1621
CP000253.1	----------	----------	1621
AC025591.8	----------	----------	1621
LT671859.1	----------	----------	1621
CP007499.1	----------	----------	1621
X17679.1	----------	----------	1621
CP007657.1	----------	----------	1621
CP014444.1	----------	----------	1621
CP014441.1	----------	----------	1621
CP014438.1	----------	----------	1621
CP014435.1	----------	----------	1621
CP014432.1	----------	----------	1621
CP014429.1	----------	----------	1621
CP014426.1	----------	----------	1621
CP014423.1	----------	----------	1621
CP014420.1	----------	----------	1621
CP014415.1	----------	----------	1621
CP014412.1	----------	----------	1621
CP014409.1	----------	----------	1621
CP014407.1	----------	----------	1621
CP014402.1	----------	----------	1621
CP014397.1	----------	----------	1621
CP014392.1	----------	----------	1621
CP014387.1	----------	----------	1621
CP014384.1	----------	----------	1621
CP014381.1	----------	----------	1621
CP014371.1	----------	----------	1621
CP014368.1	----------	----------	1621
CP014365.1	----------	----------	1621
CP014362.1	----------	----------	1621
CP014376.1	----------	----------	1621
CP009423.1	----------	----------	1621
CP016855.1	----------	----------	1621
CP013231.1	----------	----------	1621
CP010300.1	----------	----------	1621
CP010299.1	----------	----------	1621
CP010298.1	----------	----------	1621
CP010297.1	----------	----------	1621
CP010296.1	----------	----------	1621
CP010295.1	----------	----------	1621
CP007690.1	----------	----------	1621
CP007176.1	----------	----------	1621
CP000730.1	----------	----------	1621
CP000255.1	----------	----------	1621
CP007539.1	----------	----------	1620
CP007657.1	----------	----------	1621
AP014921.1	----------	----------	1621
AB436955.1	----------	----------	1529
AB489885.1	----------	----------	1529
AB489873.1	----------	----------	1529
AB489883.1	----------	----------	1529
AB489874.1	----------	----------	1517
LT615218.1	CAAGCCAAGT	AAAACGAACG	1698
AB436976.1	CAAGCCAAGT	AAAACGAACG	1698
AB489892.1	CAAGCCAAGT	AAAACGAACG	1606
AJ306908.1	CAAGCCAAGT	AAAACGAACG	1698
CP015646.1	CAAGCCAAGT	AAAACGAACG	1698
AB488510.1	----------	----------	1529
EU105387.1	----------	----------	1520
AB436975.1	TAAGCCATCA	GAAACAAACG	1698
AB488499.1	TAAGCCATCA	GAAACAAACG	1606
AJ309189.1	----------	----------	1386
FR821779.1	CAAGCCAAGC	GAAACAAATG	1683
CP012593.1	CAAGCCAAGT	GAAACAAACG	1127
CP012692.1	CAAGCCAAGT	GAAACAAACG	1127
CP013955.1	CAAGCCAAGT	GAAACAAACG	1127
CP013953.1	CAAGCCAAGT	GAAACAAACG	1127
CP014064.1	CAAGCCAAGT	GAAACAAACG	1127
LT009690.1	CAAGCCAAGT	GAAACAAACG	1127
AP017320.1	CAAGCCAAGT	GAAACAAACG	1127
CP010890.1	CAAGCCAAGT	GAAACAAACG	1127
CP001844.2	CAAGCCAAGT	GAAACAAACG	1127
CP001781.1	CAAGCCAAGT	GAAACAAACG	1127
AB488501.1	CAAGCCAAGT	GAAACAAACG	1127
AB489898.1	CAAGCCAAGT	GAAACAAACG	1127
HE579073.1	CAAGCCAAGT	GAAACAAACG	1127
HE579071.1	CAAGCCAAGT	GAAACAAACG	1127
HE579069.1	CAAGCCAAGT	GAAACAAACG	1127
HE579065.1	CAAGCCAAGT	GAAACAAACG	1127
HE579063.1	CAAGCCAAGT	GAAACAAACG	1127

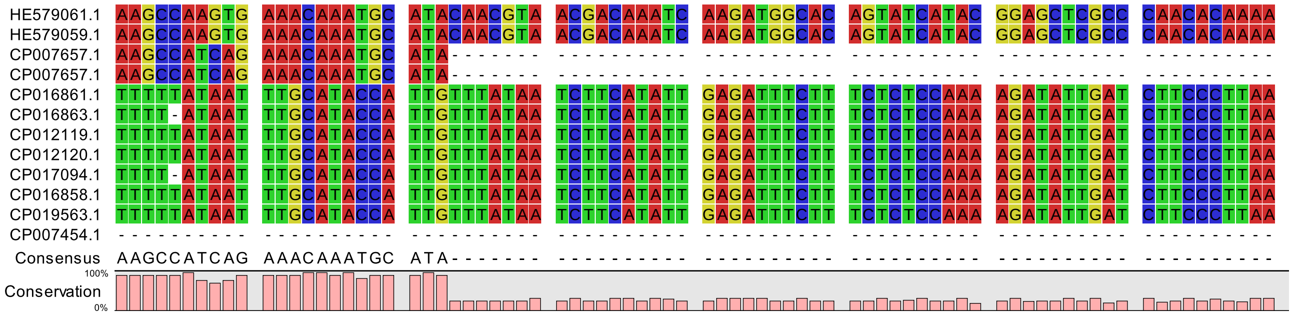
HE579061.1 AAGCCAAGTG AAACAAATGC ATACAACGTA ACGACAAATC AAGATGGCAC AGTATCATAC GGAGCTCGCC CAACACAAAA
HE579059.1 AAGCCAAGTG AAACAAATGC ATACAACGTA ACGACAAATC AAGATGGCAC AGTATCATAC GGAGCTCGCC CAACACAAAA
CP007657.1 AAGCCATCAG AAACAAATGC ATA------- ---------- ---------- ---------- ---------- ----------
CP007657.1 AAGCCATCAG AAACAAATGC ATA------- ---------- ---------- ---------- ---------- ----------
CP016861.1 TTTTTATAAT TTGCATACCA TTGTTTATAA TCTTCATATT GAGATTTCTT TCTCTCCAAA AGATATTGAT CTTCCCTTAA
CP016863.1 TTTT-ATAAT TTGCATACCA TTGTTTATAA TCTTCATATT GAGATTTCTT TCTCTCCAAA AGATATTGAT CTTCCCTTAA
CP012119.1 TTTTTATAAT TTGCATACCA TTGTTTATAA TCTTCATATT GAGATTTCTT TCTCTCCAAA AGATATTGAT CTTCCCTTAA
CP012120.1 TTTTTATAAT TTGCATACCA TTGTTTATAA TCTTCATATT GAGATTTCTT TCTCTCCAAA AGATATTGAT CTTCCCTTAA
CP017094.1 TTTT-ATAAT TTGCATACCA TTGTTTATAA TCTTCATATT GAGATTTCTT TCTCTCCAAA AGATATTGAT CTTCCCTTAA
CP016858.1 TTTTTATAAT TTGCATACCA TTGTTTATAA TCTTCATATT GAGATTTCTT TCTCTCCAAA AGATATTGAT CTTCCCTTAA
CP019563.1 TTTTTATAAT TTGCATACCA TTGTTTATAA TCTTCATATT GAGATTTCTT TCTCTCCAAA AGATATTGAT CTTCCCTTAA
CP007454.1 ---------- ---------- ---------- ---------- ---------- ---------- ---------- ----------
Consensus AAGCCATCAG AAACAAATGC ATA------- ---------- ---------- ---------- ---------- ----------
100%
Conservation
0%

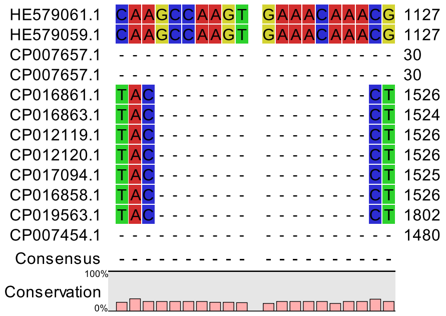
HE579061.1 CAAGCCAAGT GAAACAAACG 1127
HE579059.1 CAAGCCAAGT GAAACAAACG 1127
CP007657.1 - 30
CP007657.1 - 30
CP016861.1 TAC - - - - - - - - - - - - - - - - CT 1526
CP016863.1 TAC - - - - - - - - - - - - - - - - CT 1524
CP012119.1 TAC - - - - - - - - - - - - - - - - CT 1526
CP012120.1 TAC - - - - - - - - - - - - - - - - CT 1526
CP017094.1 TAC - - - - - - - - - - - - - - - - CT 1525
CP016858.1 TAC - - - - - - - - - - - - - - - - CT 1526
CP019563.1 TAC - - - - - - - - - - - - - - - - CT 1802
CP007454.1 - 1480
Consensus -
100%
Conservation
0%

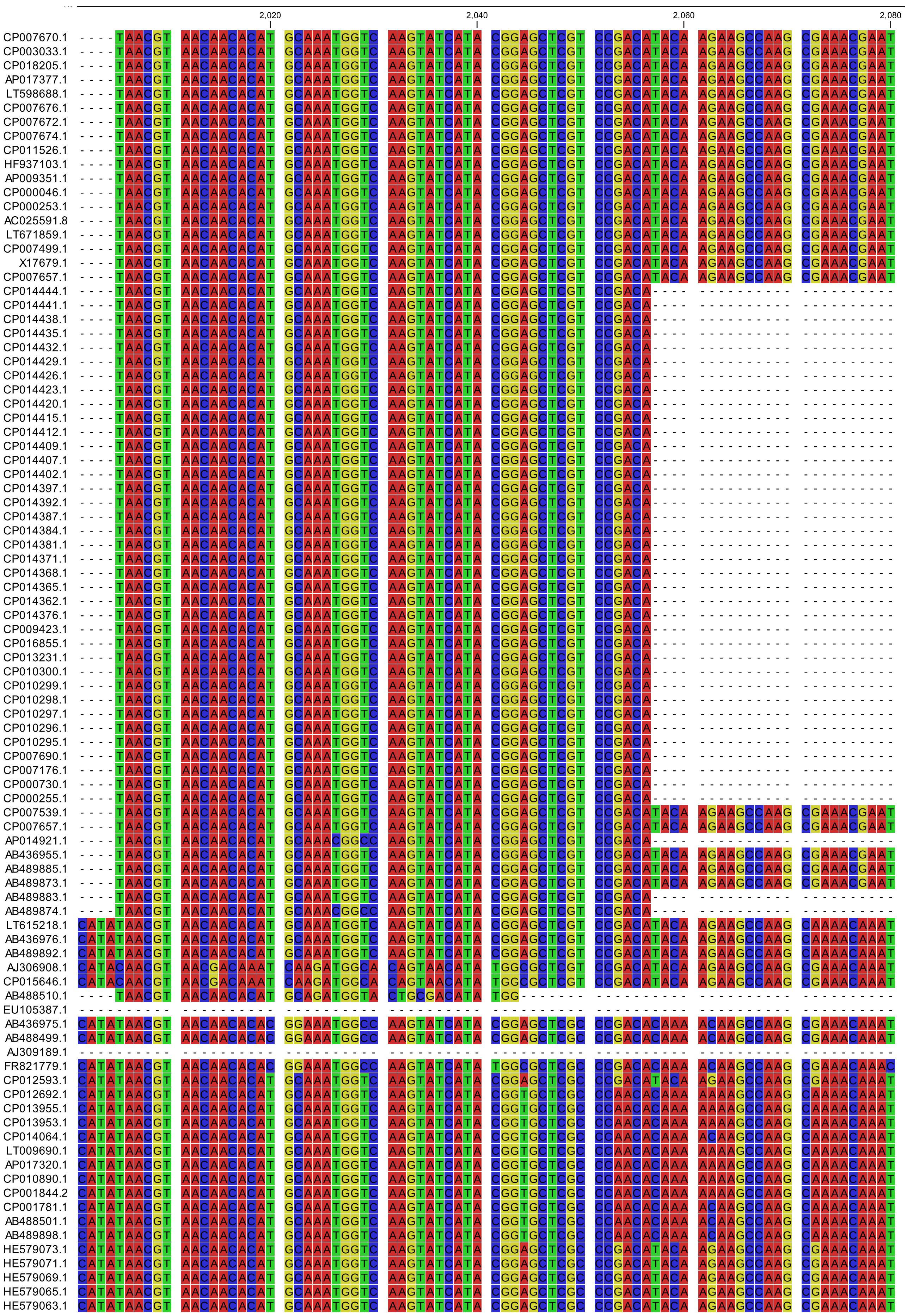
2,020
2,040
2,060
2,080
CP007670.1
CP003033.1
CP018205.1
AP017377.1
LT598688.1
CP007676.1
CP007672.1
CP007674.1
CP011526.1
HF937103.1
AP009351.1
CP000046.1
CP000253.1
AC025591.8
LT671859.1
CP007499.1
X17679.1
CP007657.1
CP014444.1
CP014441.1
CP014438.1
CP014435.1
CP014432.1
CP014429.1
CP014426.1
CP014423.1
CP014420.1
CP014415.1
CP014412.1
CP014409.1
CP014407.1
CP014402.1
CP014397.1
CP014392.1
CP014387.1
CP014384.1
CP014381.1
CP014371.1
CP014368.1
CP014365.1
CP014362.1
CP014376.1
CP009423.1
CP016855.1
CP013231.1
CP010300.1
CP010299.1
CP010298.1
CP010297.1
CP010296.1
CP010295.1
CP007690.1
CP007176.1
CP000730.1
CP000255.1
CP007539.1
CP007657.1
AP014921.1
AB436955.1
AB489885.1
AB489873.1
AB489883.1
AB489874.1
LT615218.1
AB436976.1
AB489892.1
AJ306908.1
CP015646.1
AB488510.1
EU105387.1
AB436975.1
AB488499.1
AJ309189.1
FR821779.1
CP012593.1
CP012692.1
CP013955.1
CP013953.1
CP014064.1
LT009690.1
AP017320.1
CP010890.1
CP001844.2
CP001781.1
AB488501.1
AB489898.1
HE579073.1
HE579071.1
HE579069.1
HE579065.1
HE579063.1

		2,100	
CP007670.1	GCATACAATG	TAACAACACA	1717
CP003033.1	GCATACAATG	TAACAACACA	1717
CP018205.1	GCATACAATG	TAACAACACA	1717
AP017377.1	GCATACAATG	TAACAACACA	1717
LT598688.1	GCATACAATG	TAACAACACA	1717
CP007676.1	GCATACAATG	TAACAACACA	1717
CP007672.1	GCATACAATG	TAACAACACA	1717
CP007674.1	GCATACAATG	TAACAACACA	1717
CP011526.1	GCATACAATG	TAACAACACA	1717
HF937103.1	GCATACAATG	TAACAACACA	1717
AP009351.1	GCATACAATG	TAACAACACA	1717
CP000046.1	GCATACAATG	TAACAACACA	1717
CP000253.1	GCATACAATG	TAACAACACA	1717
AC025591.8	GCATACAATG	TAACAACACA	1717
LT671859.1	GCATACAATG	TAACAACACA	1717
CP007499.1	GCATACAATG	TAACAACACA	1717
X17679.1	GCATACAATG	TAACAACACA	1717
CP007657.1	GCATACAATG	TAACAACACA	1717
CP014444.1	----------	----------	1673
CP014441.1	----------	----------	1673
CP014438.1	----------	----------	1673
CP014435.1	----------	----------	1673
CP014432.1	----------	----------	1673
CP014429.1	----------	----------	1673
CP014426.1	----------	----------	1673
CP014423.1	----------	----------	1673
CP014420.1	----------	----------	1673
CP014415.1	----------	----------	1673
CP014412.1	----------	----------	1673
CP014409.1	----------	----------	1673
CP014407.1	----------	----------	1673
CP014402.1	----------	----------	1673
CP014397.1	----------	----------	1673
CP014392.1	----------	----------	1673
CP014387.1	----------	----------	1673
CP014384.1	----------	----------	1673
CP014381.1	----------	----------	1673
CP014371.1	----------	----------	1673
CP014368.1	----------	----------	1673
CP014365.1	----------	----------	1673
CP014362.1	----------	----------	1673
CP014376.1	----------	----------	1673
CP009423.1	----------	----------	1673
CP016855.1	----------	----------	1673
CP013231.1	----------	----------	1673
CP010300.1	----------	----------	1673
CP010299.1	----------	----------	1673
CP010298.1	----------	----------	1673
CP010297.1	----------	----------	1673
CP010296.1	----------	----------	1673
CP010295.1	----------	----------	1673
CP007690.1	----------	----------	1673
CP007176.1	----------	----------	1673
CP000730.1	----------	----------	1673
CP000255.1	----------	----------	1673
CP007539.1	GCATACAATG	TAACAACACA	1716
CP007657.1	GCATACAATG	TAACAACACA	1717
AP014921.1	----------	----------	1673
AB436955.1	GCATACAATG	TAACAACACA	1625
AB489885.1	GCATACAATG	TAACAACACA	1625
AB489873.1	GCATACAATG	TAACAACACA	1625
AB489883.1	----------	----------	1581
AB489874.1	----------	----------	1569
LT615218.1	GCATACAACG	TAACAACACA	1798
AB436976.1	GCATACAACG	TAACAACACA	1798
AB489892.1	GCATACAACG	TAACAACACA	1706
AJ306908.1	GCATACAATG	TAACAACACA	1798
CP015646.1	GCATACAATG	TAACAACACA	1798
AB488510.1	----------	----------	1568
EU105387.1	----------	----------	1520
AB436975.1	GCATATAACG	TAACAACACA	1798
AB488499.1	GCATATAACG	TAACAACACA	1706
AJ309189.1	----------	----------	1386
FR821779.1	GCATATAACG	TAACAACACA	1783
CP012593.1	GCATACAACG	TAACAACACA	1227
CP012692.1	GCATACAACG	TAACAACACA	1227
CP013955.1	GCATACAACG	TAACAACACA	1227
CP013953.1	GCATACAACG	TAACAACACA	1227
CP014064.1	GCATACAACG	TAACAACACA	1227
LT009690.1	GCATACAACG	TAACAACACA	1227
AP017320.1	GCATACAACG	TAACAACACA	1227
CP010890.1	GCATACAACG	TAACAACACA	1227
CP001844.2	GCATACAACG	TAACAACACA	1227
CP001781.1	GCATACAACG	TAACAACACA	1227
AB488501.1	GCATACAACG	TAACAACACA	1227
AB489898.1	GCATACAACG	TAACAACACA	1227
HE579073.1	GCATACAACG	TAACAACACA	1227
HE579071.1	GCATACAACG	TAACAACACA	1227
HE579069.1	GCATACAACG	TAACAACACA	1227
HE579065.1	GCATACAACG	TAACAACACA	1227
HE579063.1	GCATACAACG	TAACAACACA	1227

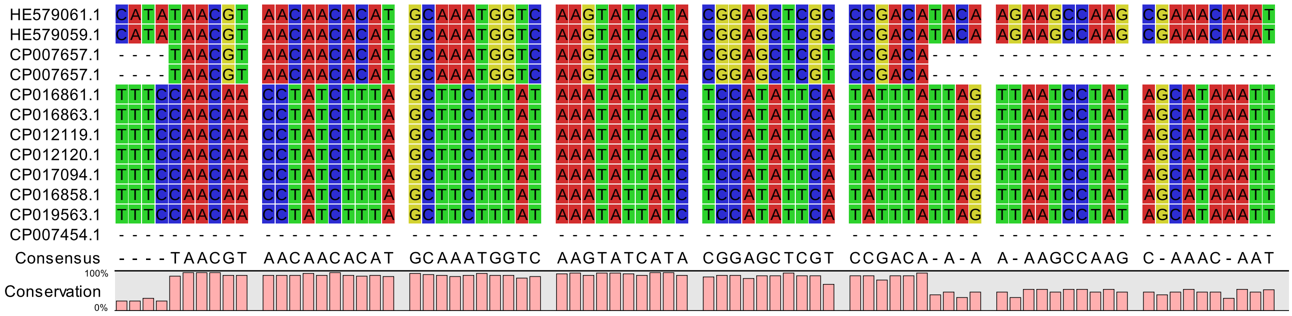
HE579061.1 CATATAACGT AACAACACAT GCAAATGGTC AAGTATCATA CGGAGCTCGC CCGACATACA AGAAGCCAAG CGAAACAAAT
HE579059.1 CATATAACGT AACAACACAT GCAAATGGTC AAGTATCATA CGGAGCTCGC CCGACATACA AGAAGCCAAG CGAAACAAAT
CP007657.1 ----TAACGT AACAACACAT GCAAATGGTC AAGTATCATA CGGAGCTCGT CCGACA---- ---------- ----------
CP007657.1 ----TAACGT AACAACACAT GCAAATGGTC AAGTATCATA CGGAGCTCGT CCGACA---- ---------- ----------
CP016861.1 TTTCCAACAA CCTATCTTTA GCTTCTTTAT AAATATTATC TCCATATTCA TATTTATTAG TTAATCCTAT AGCATAAATT
CP016863.1 TTTCCAACAA CCTATCTTTA GCTTCTTTAT AAATATTATC TCCATATTCA TATTTATTAG TTAATCCTAT AGCATAAATT
CP012119.1 TTTCCAACAA CCTATCTTTA GCTTCTTTAT AAATATTATC TCCATATTCA TATTTATTAG TTAATCCTAT AGCATAAATT
CP012120.1 TTTCCAACAA CCTATCTTTA GCTTCTTTAT AAATATTATC TCCATATTCA TATTTATTAG TTAATCCTAT AGCATAAATT
CP017094.1 TTTCCAACAA CCTATCTTTA GCTTCTTTAT AAATATTATC TCCATATTCA TATTTATTAG TTAATCCTAT AGCATAAATT
CP016858.1 TTTCCAACAA CCTATCTTTA GCTTCTTTAT AAATATTATC TCCATATTCA TATTTATTAG TTAATCCTAT AGCATAAATT
CP019563.1 TTTCCAACAA CCTATCTTTA GCTTCTTTAT AAATATTATC TCCATATTCA TATTTATTAG TTAATCCTAT AGCATAAATT
CP007454.1 ---------- ---------- ---------- ---------- ---------- ---------- ---------- ----------
Consensus ----TAACGT AACAACACAT GCAAATGGTC AAGTATCATA CGGAGCTCGT CCGACA-A-A A-AAGCCAAG C-AAAC-AAT
100%
Conservation
0%

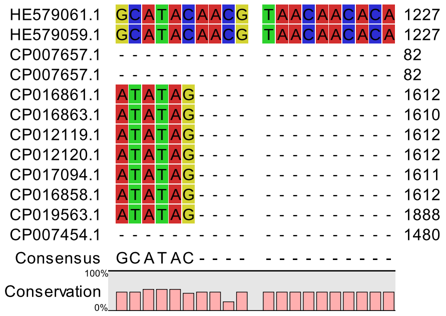
HE579061.1 GCATACAACG TAACAACACA 1227
HE579059.1 GCATACAACG TAACAACACA 1227
CP007657.1 82
CP007657.1 82
CP016861.1 ATATAG 1612
CP016863.1 ATATAG 1610
CP012119.1 ATATAG 1612
CP012120.1 ATATAG 1612
CP017094.1 ATATAG 1611
CP016858.1 ATATAG 1612
CP019563.1 ATATAG 1888
CP007454.1 1480
Consensus GCATAC
Conservation
100%
0%

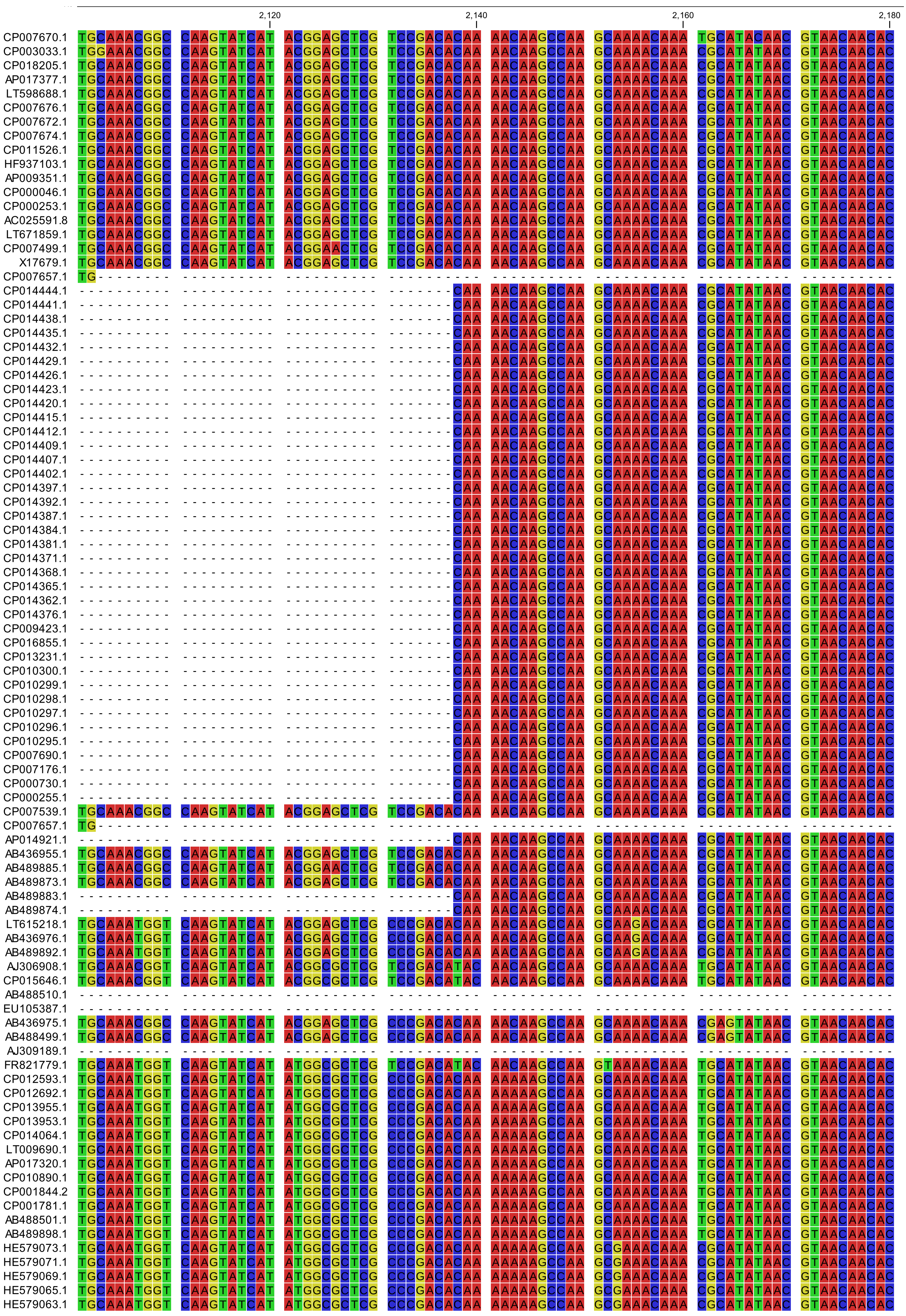
2,120
2,140
2,160
2,180
CP007670.1
CP003033.1
CP018205.1
AP017377.1
LT598688.1
CP007676.1
CP007672.1
CP007674.1
CP011526.1
HF937103.1
AP009351.1
CP000046.1
CP000253.1
AC025591.8
LT671859.1
CP007499.1
X17679.1
CP007657.1
CP014444.1
CP014441.1
CP014438.1
CP014435.1
CP014432.1
CP014429.1
CP014426.1
CP014423.1
CP014420.1
CP014415.1
CP014412.1
CP014409.1
CP014407.1
CP014402.1
CP014397.1
CP014392.1
CP014387.1
CP014384.1
CP014381.1
CP014371.1
CP014368.1
CP014365.1
CP014362.1
CP014376.1
CP009423.1
CP016855.1
CP013231.1
CP010300.1
CP010299.1
CP010298.1
CP010297.1
CP010296.1
CP010295.1
CP007690.1
CP007176.1
CP000730.1
CP000255.1
CP007539.1
CP007657.1
AP014921.1
AB436955.1
AB489885.1
AB489873.1
AB489883.1
AB489874.1
LT615218.1
AB436976.1
AB489892.1
AJ306908.1
CP015646.1
AB488510.1
EU105387.1
AB436975.1
AB488499.1
AJ309189.1
FR821779.1
CP012593.1
CP012692.1
CP013955.1
CP013953.1
CP014064.1
LT009690.1
AP017320.1
CP010890.1
CP001844.2
CP001781.1
AB488501.1
AB489898.1
HE579073.1
HE579071.1
HE579069.1
HE579065.1
HE579063.1

	2,200		
CP007670.1	ATGGAAACGG	CCAAGTATCA	1817
CP003033.1	ATGGAAACGG	CCAAGTATCA	1817
CP018205.1	ATGGAAACGG	CCAAGTATCA	1817
AP017377.1	ATGGAAACGG	CCAAGTATCA	1817
LT598688.1	ATGGAAACGG	CCAAGTATCA	1817
CP007676.1	ATGGAAACGG	CCAAGTATCA	1817
CP007672.1	ATGGAAACGG	CCAAGTATCA	1817
CP007674.1	ATGGAAACGG	CCAAGTATCA	1817
CP011526.1	ATGGAAACGG	CCAAGTATCA	1817
HF937103.1	ATGGAAACGG	CCAAGTATCA	1817
AP009351.1	ATGGAAACGG	CCAAGTATCA	1817
CP000046.1	ATGGAAACGG	CCAAGTATCA	1817
CP000253.1	ATGGAAACGG	CCAAGTATCA	1817
AC025591.8	ATGGAAACGG	CCAAGTATCA	1817
LT671859.1	ATGGAAACGG	CCAAGTATCA	1817
CP007499.1	ATGGAAACGG	CCAAGTATCA	1817
X17679.1	ATGGAAACGG	CCAAGTATCA	1817
CP007657.1	---GAAACGG	CCAAGTATCA	1736
CP014444.1	ATGGAAACGG	CCAAGTATCA	1736
CP014441.1	ATGGAAACGG	CCAAGTATCA	1736
CP014438.1	ATGGAAACGG	CCAAGTATCA	1736
CP014435.1	ATGGAAACGG	CCAAGTATCA	1736
CP014432.1	ATGGAAACGG	CCAAGTATCA	1736
CP014429.1	ATGGAAACGG	CCAAGTATCA	1736
CP014426.1	ATGGAAACGG	CCAAGTATCA	1736
CP014423.1	ATGGAAACGG	CCAAGTATCA	1736
CP014420.1	ATGGAAACGG	CCAAGTATCA	1736
CP014415.1	ATGGAAACGG	CCAAGTATCA	1736
CP014412.1	ATGGAAACGG	CCAAGTATCA	1736
CP014409.1	ATGGAAACGG	CCAAGTATCA	1736
CP014407.1	ATGGAAACGG	CCAAGTATCA	1736
CP014402.1	ATGGAAACGG	CCAAGTATCA	1736
CP014397.1	ATGGAAACGG	CCAAGTATCA	1736
CP014392.1	ATGGAAACGG	CCAAGTATCA	1736
CP014387.1	ATGGAAACGG	CCAAGTATCA	1736
CP014384.1	ATGGAAACGG	CCAAGTATCA	1736
CP014381.1	ATGGAAACGG	CCAAGTATCA	1736
CP014371.1	ATGGAAACGG	CCAAGTATCA	1736
CP014368.1	ATGGAAACGG	CCAAGTATCA	1736
CP014365.1	ATGGAAACGG	CCAAGTATCA	1736
CP014362.1	ATGGAAACGG	CCAAGTATCA	1736
CP014376.1	ATGGAAACGG	CCAAGTATCA	1736
CP009423.1	ATGGAAACGG	CCAAGTATCA	1736
CP016855.1	ATGGAAACGG	CCAAGTATCA	1736
CP013231.1	ATGGAAACGG	CCAAGTATCA	1736
CP010300.1	ATGGAAACGG	CCAAGTATCA	1736
CP010299.1	ATGGAAACGG	CCAAGTATCA	1736
CP010298.1	ATGGAAACGG	CCAAGTATCA	1736
CP010297.1	ATGGAAACGG	CCAAGTATCA	1736
CP010296.1	ATGGAAACGG	CCAAGTATCA	1736
CP010295.1	ATGGAAACGG	CCAAGTATCA	1736
CP007690.1	ATGGAAACGG	CCAAGTATCA	1736
CP007176.1	ATGGAAACGG	CCAAGTATCA	1736
CP000730.1	ATGGAAACGG	CCAAGTATCA	1736
CP000255.1	ATGGAAACGG	CCAAGTATCA	1736
CP007539.1	ATGGAAACGG	CCAAGTATCA	1816
CP007657.1	---GAAACGG	CCAAGTATCA	1736
AP014921.1	ATGGAAACGG	CCAAGTATCA	1736
AB436955.1	ATGGAAACGG	CCAAGTATCA	1725
AB489885.1	ATGGAAACGG	CCAAGTATCA	1725
AB489873.1	ATGGAAACGG	CCAAGTATCA	1725
AB489883.1	ATGGAAACGG	CCAAGTATCA	1644
AB489874.1	ATGGAAACGG	CCAAGTATCA	1632
LT615218.1	ATGCAAACGG	CCAAGTATCA	1898
AB436976.1	ATGCAAACGG	CCAAGTATCA	1898
AB489892.1	ATGCAAACGG	CCAAGTATCA	1806
AJ306908.1	ATGCAAACGG	TCAAGTATCA	1898
CP015646.1	ATGCAAACGG	TCAAGTATCA	1898
AB488510.1	----------	----------	1568
EU105387.1	----------	----------	1520
AB436975.1	ATGCAAATGG	CCAAGTATCA	1898
AB488499.1	ATGCAAATGG	CCAAGTATCA	1806
AJ309189.1	----------	----------	1386
FR821779.1	ATGCAAACGG	CCAAGTATCA	1883
CP012593.1	ATGCAAATGG	TCAAGTATCA	1327
CP012692.1	ATGCAAATGG	TCAAGTATCA	1327
CP013955.1	ATGCAAATGG	TCAAGTATCA	1327
CP013953.1	ATGCAAATGG	TCAAGTATCA	1327
CP014064.1	ATGCAAATGG	TCAAGTATCA	1327
LT009690.1	ATGCAAATGG	TCAAGTATCA	1327
AP017320.1	ATGCAAATGG	TCAAGTATCA	1327
CP010890.1	ATGCAAATGG	TCAAGTATCA	1327
CP001844.2	ATGCAAATGG	TCAAGTATCA	1327
CP001781.1	ATGCAAATGG	TCAAGTATCA	1327
AB488501.1	ATGCAAATGG	TCAAGTATCA	1327
AB489898.1	ATGCAAATGG	TCAAGTATCA	1327
HE579073.1	ATGCAAATGG	TCAAGTATCA	1327
HE579071.1	ATGCAAATGG	TCAAGTATCA	1327
HE579069.1	ATGCAAATGG	TCAAGTATCA	1327
HE579065.1	ATGCAAATGG	TCAAGTATCA	1327
HE579063.1	ATGCAAATGG	TCAAGTATCA	1327

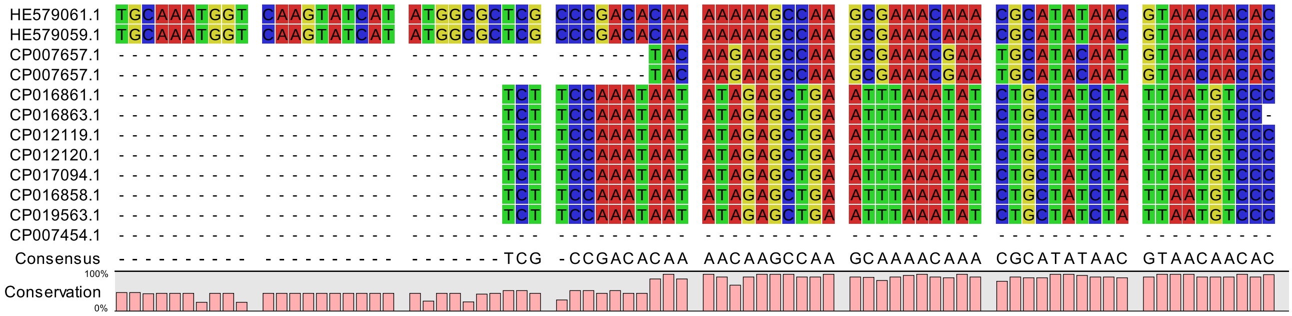
HE579061.1 TGCAAATGGT CAAGTATCAT ATGGCGCTCG CCCGACACAA AAAAAGCCAA GCGAAACAAA CGCATATAAC GTAACAACAC
HE579059.1 TGCAAATGGT CAAGTATCAT ATGGCGCTCG CCCGACACAA AAAAAGCCAA GCGAAACAAA CGCATATAAC GTAACAACAC
CP007657.1 ---------- ---------- ---------- -------TAC AAGAAGCCAA GCGAAACGAA TGCATACAAT GTAACAACAC
CP007657.1 ---------- ---------- ---------- -------TAC AAGAAGCCAA GCGAAACGAA TGCATACAAT GTAACAACAC
CP016861.1 ---------- ---------- -------TCT TCCAAATAAT ATAGAGCTGA ATTTAAATAT CTGCTATCTA TTAATGTCCC
CP016863.1 ---------- ---------- -------TCT TCCAAATAAT ATAGAGCTGA ATTTAAATAT CTGCTATCTA TTAATGTCC-
CP012119.1 ---------- ---------- -------TCT TCCAAATAAT ATAGAGCTGA ATTTAAATAT CTGCTATCTA TTAATGTCCC
CP012120.1 ---------- ---------- -------TCT TCCAAATAAT ATAGAGCTGA ATTTAAATAT CTGCTATCTA TTAATGTCCC
CP017094.1 ---------- ---------- -------TCT TCCAAATAAT ATAGAGCTGA ATTTAAATAT CTGCTATCTA TTAATGTCCC
CP016858.1 ---------- ---------- -------TCT TCCAAATAAT ATAGAGCTGA ATTTAAATAT CTGCTATCTA TTAATGTCCC
CP019563.1 ---------- ---------- -------TCT TCCAAATAAT ATAGAGCTGA ATTTAAATAT CTGCTATCTA TTAATGTCCC
CP007454.1 ---------- ---------- ---------- ---------- ---------- ---------- ---------- ----------
Consensus ---------- ---------- -------TCG -CCGACACAA AACAAGCCAA GCAAAACAAA CGCATATAAC GTAACAACAC
100%
Conservation
0%

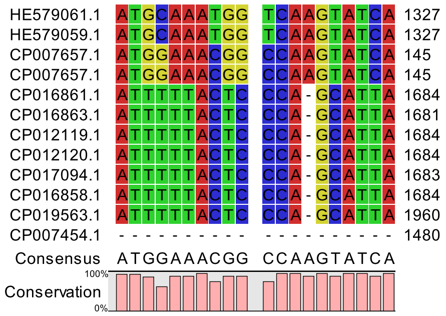
HE579061.1 ATGCAAATGG TCAAGTATCA 1327
HE579059.1 ATGCAAATGG TCAAGTATCA 1327
CP007657.1 ATGGAAACGG CCAAGTATCA 145
CP007657.1 ATGGAAACGG CCAAGTATCA 145
CP016861.1 ATTTTTACTC CCA-GCATTA 1684
CP016863.1 ATTTTTACTC CCA-GCATTA 1681
CP012119.1 ATTTTTACTC CCA-GCATTA 1684
CP012120.1 ATTTTTACTC CCA-GCATTA 1684
CP017094.1 ATTTTTACTC CCA-GCATTA 1683
CP016858.1 ATTTTTACTC CCA-GCATTA 1684
CP019563.1 ATTTTTACTC CCA-GCATTA 1960
CP007454.1 ---------- ---------- 1480
Consensus ATGGAAACGG CCAAGTATCA
Conservation
100%
0%

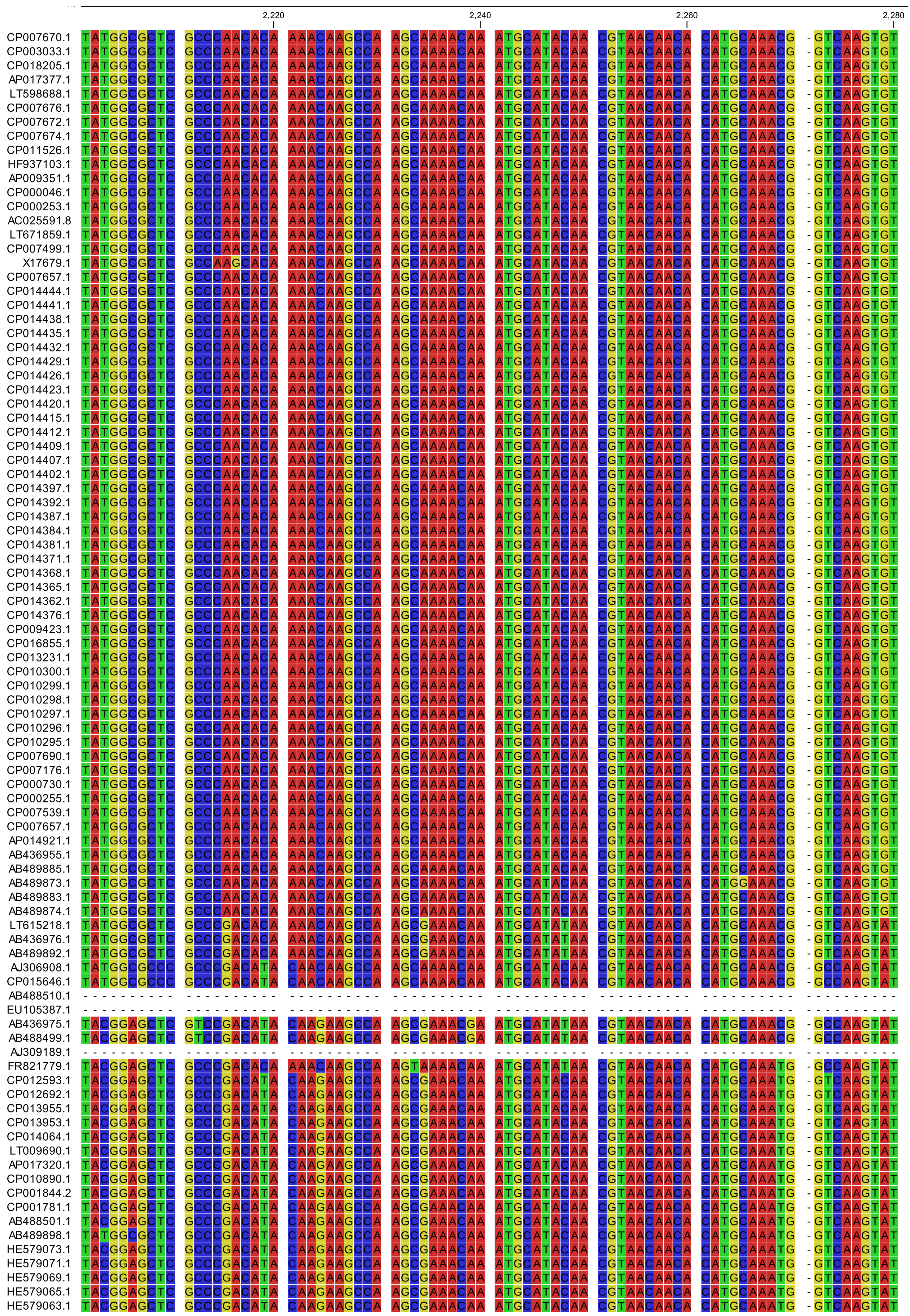
2,220
2,240
2,260
2,280
CP007670.1
CP003033.1
CP018205.1
AP017377.1
LT598688.1
CP007676.1
CP007672.1
CP007674.1
CP011526.1
HF937103.1
AP009351.1
CP000046.1
CP000253.1
AC025591.8
LT671859.1
CP007499.1
X17679.1
CP007657.1
CP014444.1
CP014441.1
CP014438.1
CP014435.1
CP014432.1
CP014429.1
CP014426.1
CP014423.1
CP014420.1
CP014415.1
CP014412.1
CP014409.1
CP014407.1
CP014402.1
CP014397.1
CP014392.1
CP014387.1
CP014384.1
CP014381.1
CP014371.1
CP014368.1
CP014365.1
CP014362.1
CP014376.1
CP009423.1
CP016855.1
CP013231.1
CP010300.1
CP010299.1
CP010298.1
CP010297.1
CP010296.1
CP010295.1
CP007690.1
CP007176.1
CP000730.1
CP000255.1
CP007539.1
CP007657.1
AP014921.1
AB436955.1
AB489885.1
AB489873.1
AB489883.1
AB489874.1
LT615218.1
AB436976.1
AB489892.1
AJ306908.1
CP015646.1
AB488510.1
EU105387.1
AB436975.1
AB488499.1
AJ309189.1
FR821779.1
CP012593.1
CP012692.1
CP013955.1
CP013953.1
CP014064.1
LT009690.1
AP017320.1
CP010890.1
CP001844.2
CP001781.1
AB488501.1
AB489898.1
HE579073.1
HE579071.1
HE579069.1
HE579065.1
HE579063.1

2,300

CP007670.1	CATACGGAGC	TCGCCC----	1912
CP003033.1	CATACGGAGC	TCGCCC----	1912
CP018205.1	CATACGGAGC	TCGCCC----	1912
AP017377.1	CATACGGAGC	TCGCCC----	1912
LT598688.1	CATACGGAGC	TCGCCC----	1912
CP007676.1	CATACGGAGC	TCGCCC----	1912
CP007672.1	CATACGGAGC	TCGCCC----	1912
CP007674.1	CATACGGAGC	TCGCCC----	1912
CP011526.1	CATACGGAGC	TCGCCC----	1912
HF937103.1	CATACGGAGC	TCGCCC----	1912
AP009351.1	CATACGGAGC	TCGCCC----	1912
CP000046.1	CATACGGAGC	TCGCCC----	1912
CP000253.1	CATACGGAGC	TCGCCC----	1912
AC025591.8	CATACGGAGC	TCGCCC----	1912
LT671859.1	CATACGGAGC	TCGCCC----	1912
CP007499.1	CATACGGAGC	TCGCCC----	1912
X17679.1	CATACGGAGC	TCGCCC----	1912
CP007657.1	CATACGGAGC	TCGCCC----	1831
CP014444.1	CATACGGAGC	TCGCCC----	1831
CP014441.1	CATACGGAGC	TCGCCC----	1831
CP014438.1	CATACGGAGC	TCGCCC----	1831
CP014435.1	CATACGGAGC	TCGCCC----	1831
CP014432.1	CATACGGAGC	TCGCCC----	1831
CP014429.1	CATACGGAGC	TCGCCC----	1831
CP014426.1	CATACGGAGC	TCGCCC----	1831
CP014423.1	CATACGGAGC	TCGCCC----	1831
CP014420.1	CATACGGAGC	TCGCCC----	1831
CP014415.1	CATACGGAGC	TCGCCC----	1831
CP014412.1	CATACGGAGC	TCGCCC----	1831
CP014409.1	CATACGGAGC	TCGCCC----	1831
CP014407.1	CATACGGAGC	TCGCCC----	1831
CP014402.1	CATACGGAGC	TCGCCC----	1831
CP014397.1	CATACGGAGC	TCGCCC----	1831
CP014392.1	CATACGGAGC	TCGCCC----	1831
CP014387.1	CATACGGAGC	TCGCCC----	1831
CP014384.1	CATACGGAGC	TCGCCC----	1831
CP014381.1	CATACGGAGC	TCGCCC----	1831
CP014371.1	CATACGGAGC	TCGCCC----	1831
CP014368.1	CATACGGAGC	TCGCCC----	1831
CP014365.1	CATACGGAGC	TCGCCC----	1831
CP014362.1	CATACGGAGC	TCGCCC----	1831
CP014376.1	CATACGGAGC	TCGCCC----	1831
CP009423.1	CATACGGAGC	TCGCCC----	1831
CP016855.1	CATACGGAGC	TCGCCC----	1831
CP013231.1	CATACGGAGC	TCGCCC----	1831
CP010300.1	CATACGGAGC	TCGCCC----	1831
CP010299.1	CATACGGAGC	TCGCCC----	1831
CP010298.1	CATACGGAGC	TCGCCC----	1831
CP010297.1	CATACGGAGC	TCGCCC----	1831
CP010296.1	CATACGGAGC	TCGCCC----	1831
CP010295.1	CATACGGAGC	TCGCCC----	1831
CP007690.1	CATACGGAGC	TCGCCC----	1831
CP007176.1	CATACGGAGC	TCGCCC----	1831
CP000730.1	CATACGGAGC	TCGCCC----	1831
CP000255.1	CATACGGAGC	TCGCCC----	1831
CP007539.1	CATACGGAGC	TCGCCC----	1911
CP007657.1	CATACGGAGC	TCGCCC----	1831
AP014921.1	CATACGGAGC	TCGCCC----	1831
AB436955.1	CATACGGAGC	TCGCCC----	1820
AB489885.1	CATACGGAGC	TCGCCC----	1820
AB489873.1	CATACGGAGC	TCGCCC----	1820
AB489883.1	CATACGGAGC	TCGCCC----	1739
AB489874.1	CATACGGAGC	TCGCCC----	1727
LT615218.1	CATATGGCGC	TCGCCCAACA	1997
AB436976.1	CATATGGCGC	TCGCCCAACA	1997
AB489892.1	CATATGGCGC	TCGCCCAACA	1905
AJ306908.1	CATATGGCGC	TCGCCCGACA	1997
CP015646.1	CATATGGCGC	TCGCCCGACA	1997
AB488510.1	----------	----------	1568
EU105387.1	----------	----------	1520
AB436975.1	CATATGGCGC	TCGCCCAACA	1997
AB488499.1	CATATGGCGC	TCGCCCAACA	1905
AJ309189.1	----------	----------	1386
FR821779.1	CATACGGTGC	TCGCCCGACA	1982
CP012593.1	CATATGGCGC	TCGCCC----	1422
CP012692.1	CATATGGCGC	TCGCCC----	1422
CP013955.1	CATATGGCGC	TCGCCC----	1422
CP013953.1	CATATGGCGC	TCGCCC----	1422
CP014064.1	CATATGGCGC	TCGCCC----	1422
LT009690.1	CATATGGCGC	TCGCCC----	1422
AP017320.1	CATATGGCGC	TCGCCC----	1422
CP010890.1	CATATGGCGC	TCGCCC----	1422
CP001844.2	CATATGGCGC	TCGCCC----	1422
CP001781.1	CATATGGCGC	TCGCCC----	1422
AB488501.1	CATATGGCGC	TCGCCC----	1422
AB489898.1	CATATGGCGC	TCGCCC----	1422
HE579073.1	CATATGGCGC	TCGCCC----	1422
HE579071.1	CATATGGCGC	TCGCCC----	1422
HE579069.1	CATATGGCGC	TCGCCC----	1422
HE579065.1	CATATGGCGC	TCGCCC----	1422
HE579063.1	CATATGGCGC	TCGCCC----	1422

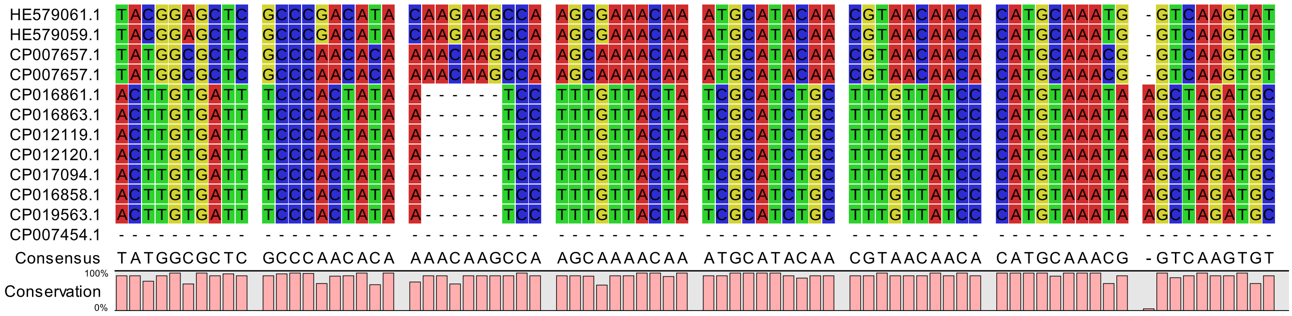
HE579061.1 TACGGAGCTC GCCCGACATA CAAGAAGCCA AGCGAAACAA ATGCATACAA CGTAACAACA CATGCAAATG -GTCAAGTAT
HE579059.1 TACGGAGCTC GCCCGACATA CAAGAAGCCA AGCGAAACAA ATGCATACAA CGTAACAACA CATGCAAATG -GTCAAGTAT
CP007657.1 TATGGCGCTC GCCCAACACA AAACAAGCCA AGCAAAACAA ATGCATACAA CGTAACAACA CATGCAAACG -GTCAAGTGT
CP007657.1 TATGGCGCTC GCCCAACACA AAACAAGCCA AGCAAAACAA ATGCATACAA CGTAACAACA CATGCAAACG -GTCAAGTGT
CP016861.1 ACTTGTGATT TCCCACTATA A------TCC TTTGTTACTA TCGCATCTGC TTTGTTATCC CATGTAAATA AGCTAGATGC
CP016863.1 ACTTGTGATT TCCCACTATA A------TCC TTTGTTACTA TCGCATCTGC TTTGTTATCC CATGTAAATA AGCTAGATGC
CP012119.1 ACTTGTGATT TCCCACTATA A------TCC TTTGTTACTA TCGCATCTGC TTTGTTATCC CATGTAAATA AGCTAGATGC
CP012120.1 ACTTGTGATT TCCCACTATA A------TCC TTTGTTACTA TCGCATCTGC TTTGTTATCC CATGTAAATA AGCTAGATGC
CP017094.1 ACTTGTGATT TCCCACTATA A------TCC TTTGTTACTA TCGCATCTGC TTTGTTATCC CATGTAAATA AGCTAGATGC
CP016858.1 ACTTGTGATT TCCCACTATA A------TCC TTTGTTACTA TCGCATCTGC TTTGTTATCC CATGTAAATA AGCTAGATGC
CP019563.1 ACTTGTGATT TCCCACTATA A------TCC TTTGTTACTA TCGCATCTGC TTTGTTATCC CATGTAAATA AGCTAGATGC
CP007454.1 ---------- ---------- ---------- ---------- ---------- ---------- ---------- ----------
Consensus TATGGCGCTC GCCCAACACA AAACAAGCCA AGCAAAACAA ATGCATACAA CGTAACAACA CATGCAAACG -GTCAAGTGT
100%
Conservation
0%

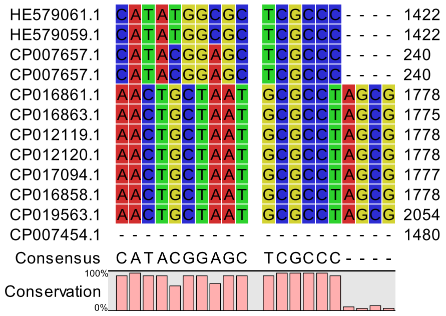
HE579061.1 CATATGGCGC TCGCCC---- 1422
HE579059.1 CATATGGCGC TCGCCC---- 1422
CP007657.1 CATACGGAGC TCGCCC---- 240
CP007657.1 CATACGGAGC TCGCCC---- 240
CP016861.1 AACTGCTAAT GCGCCTAGCG 1778
CP016863.1 AACTGCTAAT GCGCCTAGCG 1775
CP012119.1 AACTGCTAAT GCGCCTAGCG 1778
CP012120.1 AACTGCTAAT GCGCCTAGCG 1778
CP017094.1 AACTGCTAAT GCGCCTAGCG 1777
CP016858.1 AACTGCTAAT GCGCCTAGCG 1778
CP019563.1 AACTGCTAAT GCGCCTAGCG 2054
CP007454.1 ---------- ---------- 1480
Consensus CATACGGAGC TCGCCC----
Conservation
100%
0%

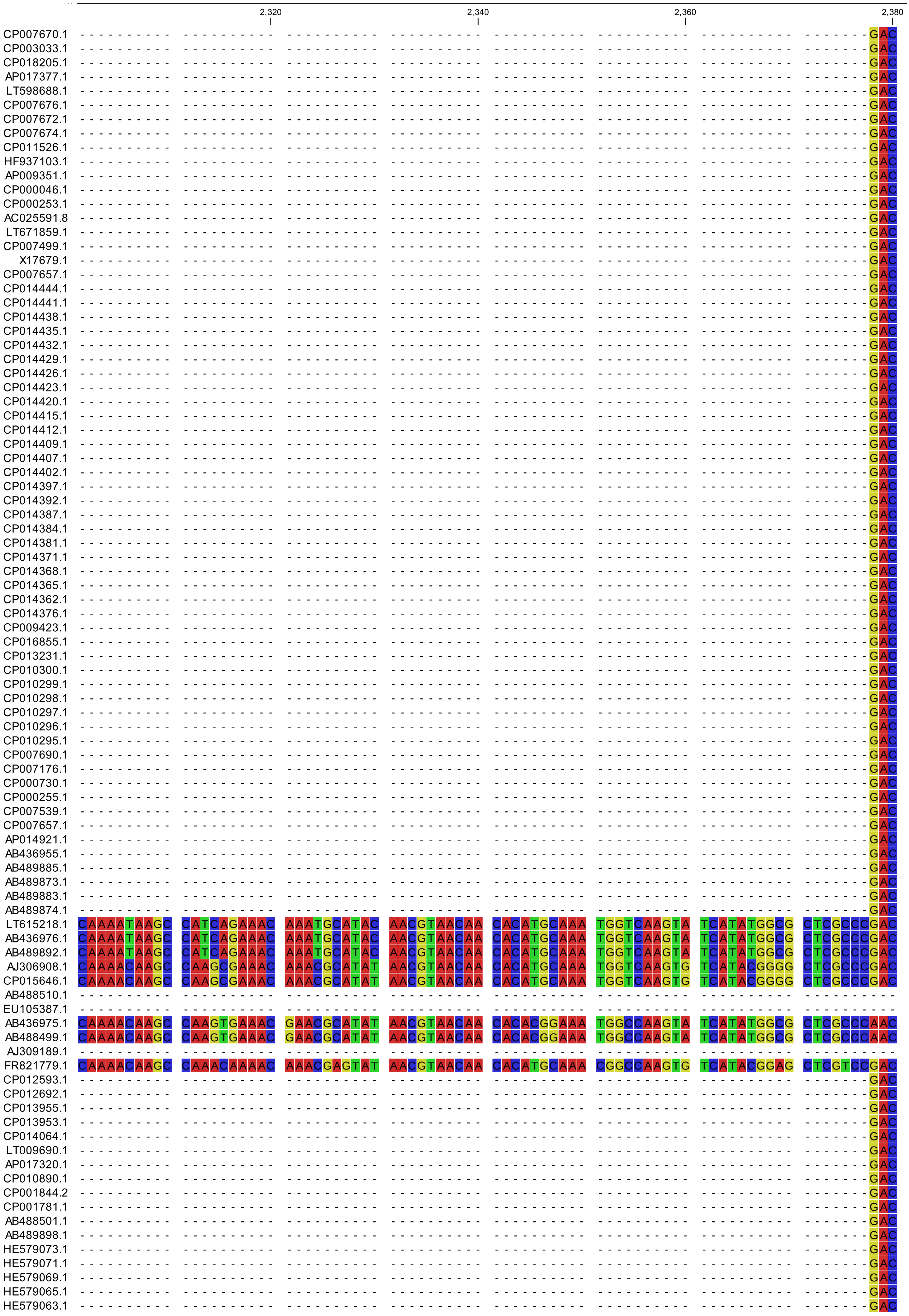

2,320
2,340
2,360
2,380
CP007670.1 ---------- ---------- ---------- ---------- ---------- ---------- ---------- -------GAC
CP003033.1 ---------- ---------- ---------- ---------- ---------- ---------- ---------- -------GAC
CP018205.1 ---------- ---------- ---------- ---------- ---------- ---------- ---------- -------GAC
AP017377.1 ---------- ---------- ---------- ---------- ---------- ---------- ---------- -------GAC
LT598688.1 ---------- ---------- ---------- ---------- ---------- ---------- ---------- -------GAC
CP007676.1 ---------- ---------- ---------- ---------- ---------- ---------- ---------- -------GAC
CP007672.1 ---------- ---------- ---------- ---------- ---------- ---------- ---------- -------GAC
CP007674.1 ---------- ---------- ---------- ---------- ---------- ---------- ---------- -------GAC
CP011526.1 ---------- ---------- ---------- ---------- ---------- ---------- ---------- -------GAC
HF937103.1 ---------- ---------- ---------- ---------- ---------- ---------- ---------- -------GAC
AP009351.1 ---------- ---------- ---------- ---------- ---------- ---------- ---------- -------GAC
CP000046.1 ---------- ---------- ---------- ---------- ---------- ---------- ---------- -------GAC
CP000253.1 ---------- ---------- ---------- ---------- ---------- ---------- ---------- -------GAC
AC025591.8 ---------- ---------- ---------- ---------- ---------- ---------- ---------- -------GAC
LT671859.1 ---------- ---------- ---------- ---------- ---------- ---------- ---------- -------GAC
CP007499.1 ---------- ---------- ---------- ---------- ---------- ---------- ---------- -------GAC
X17679.1 ---------- ---------- ---------- ---------- ---------- ---------- ---------- -------GAC
CP007657.1 ---------- ---------- ---------- ---------- ---------- ---------- ---------- -------GAC
CP014444.1 ---------- ---------- ---------- ---------- ---------- ---------- ---------- -------GAC
CP014441.1 ---------- ---------- ---------- ---------- ---------- ---------- ---------- -------GAC
CP014438.1 ---------- ---------- ---------- ---------- ---------- ---------- ---------- -------GAC
CP014435.1 ---------- ---------- ---------- ---------- ---------- ---------- ---------- -------GAC
CP014432.1 ---------- ---------- ---------- ---------- ---------- ---------- ---------- -------GAC
CP014429.1 ---------- ---------- ---------- ---------- ---------- ---------- ---------- -------GAC
CP014426.1 ---------- ---------- ---------- ---------- ---------- ---------- ---------- -------GAC
CP014423.1 ---------- ---------- ---------- ---------- ---------- ---------- ---------- -------GAC
CP014420.1 ---------- ---------- ---------- ---------- ---------- ---------- ---------- -------GAC
CP014415.1 ---------- ---------- ---------- ---------- ---------- ---------- ---------- -------GAC
CP014412.1 ---------- ---------- ---------- ---------- ---------- ---------- ---------- -------GAC
CP014409.1 ---------- ---------- ---------- ---------- ---------- ---------- ---------- -------GAC
CP014407.1 ---------- ---------- ---------- ---------- ---------- ---------- ---------- -------GAC
CP014402.1 ---------- ---------- ---------- ---------- ---------- ---------- ---------- -------GAC
CP014397.1 ---------- ---------- ---------- ---------- ---------- ---------- ---------- -------GAC
CP014392.1 ---------- ---------- ---------- ---------- ---------- ---------- ---------- -------GAC
CP014387.1 ---------- ---------- ---------- ---------- ---------- ---------- ---------- -------GAC
CP014384.1 ---------- ---------- ---------- ---------- ---------- ---------- ---------- -------GAC
CP014381.1 ---------- ---------- ---------- ---------- ---------- ---------- ---------- -------GAC
CP014371.1 ---------- ---------- ---------- ---------- ---------- ---------- ---------- -------GAC
CP014368.1 ---------- ---------- ---------- ---------- ---------- ---------- ---------- -------GAC
CP014365.1 ---------- ---------- ---------- ---------- ---------- ---------- ---------- -------GAC
CP014362.1 ---------- ---------- ---------- ---------- ---------- ---------- ---------- -------GAC
CP014376.1 ---------- ---------- ---------- ---------- ---------- ---------- ---------- -------GAC
CP009423.1 ---------- ---------- ---------- ---------- ---------- ---------- ---------- -------GAC
CP016855.1 ---------- ---------- ---------- ---------- ---------- ---------- ---------- -------GAC
CP013231.1 ---------- ---------- ---------- ---------- ---------- ---------- ---------- -------GAC
CP010300.1 ---------- ---------- ---------- ---------- ---------- ---------- ---------- -------GAC
CP010299.1 ---------- ---------- ---------- ---------- ---------- ---------- ---------- -------GAC
CP010298.1 ---------- ---------- ---------- ---------- ---------- ---------- ---------- -------GAC
CP010297.1 ---------- ---------- ---------- ---------- ---------- ---------- ---------- -------GAC
CP010296.1 ---------- ---------- ---------- ---------- ---------- ---------- ---------- -------GAC
CP010295.1 ---------- ---------- ---------- ---------- ---------- ---------- ---------- -------GAC
CP007690.1 ---------- ---------- ---------- ---------- ---------- ---------- ---------- -------GAC
CP007176.1 ---------- ---------- ---------- ---------- ---------- ---------- ---------- -------GAC
CP000730.1 ---------- ---------- ---------- ---------- ---------- ---------- ---------- -------GAC
CP000255.1 ---------- ---------- ---------- ---------- ---------- ---------- ---------- -------GAC
CP007539.1 ---------- ---------- ---------- ---------- ---------- ---------- ---------- -------GAC
CP007657.1 ---------- ---------- ---------- ---------- ---------- ---------- ---------- -------GAC
AP014921.1 ---------- ---------- ---------- ---------- ---------- ---------- ---------- -------GAC
AB436955.1 ---------- ---------- ---------- ---------- ---------- ---------- ---------- -------GAC
AB489885.1 ---------- ---------- ---------- ---------- ---------- ---------- ---------- -------GAC
AB489873.1 ---------- ---------- ---------- ---------- ---------- ---------- ---------- -------GAC
AB489883.1 ---------- ---------- ---------- ---------- ---------- ---------- ---------- -------GAC
AB489874.1 ---------- ---------- ---------- ---------- ---------- ---------- ---------- -------GAC
LT615218.1 CAAAATAAGC CATCAGAAAC AAATGCATAC AACGTAACAA CACATGCAAA TGGTCAAGTA TCATATGGCG CTCGCCCGAC
AB436976.1 CAAAATAAGC CATCAGAAAC AAATGCATAC AACGTAACAA CACATGCAAA TGGTCAAGTA TCATATGGCG CTCGCCCGAC
AB489892.1 CAAAATAAGC CATCAGAAAC AAATGCATAC AACGTAACAA CACATGCAAA TGGTCAAGTA TCATATGGCG CTCGCCCGAC
AJ306908.1 CAAAACAAGC CAAGCGAAAC AAACGCATAT AACGTAACAA CACATGCAAA TGGTCAAGTG TCATACGGGG CTCGCCCGAC
CP015646.1 CAAAACAAGC CAAGCGAAAC AAACGCATAT AACGTAACAA CACATGCAAA TGGTCAAGTG TCATACGGGG CTCGCCCGAC
AB488510.1 ---------- ---------- ---------- ---------- ---------- ---------- ---------- ----------
EU105387.1 ---------- ---------- ---------- ---------- ---------- ---------- ---------- ----------
AB436975.1 CAAAACAAGC CAAGTGAAAC GAACGCATAT AACGTAACAA CACACGGAAA TGGCCAAGTA TCATATGGCG CTCGCCCAAC
AB488499.1 CAAAACAAGC CAAGTGAAAC GAACGCATAT AACGTAACAA CACACGGAAA TGGCCAAGTA TCATATGGCG CTCGCCCAAC
AJ309189.1 ---------- ---------- ---------- ---------- ---------- ---------- ---------- ----------
FR821779.1 CAAAACAAGC CAAACAAAAC AAACGAGTAT AACGTAACAA CACATGCAAA CGGCCAAGTG TCATACGGAG CTCGTCCGAC
CP012593.1 ---------- ---------- ---------- ---------- ---------- ---------- ---------- -------GAC
CP012692.1 ---------- ---------- ---------- ---------- ---------- ---------- ---------- -------GAC
CP013955.1 ---------- ---------- ---------- ---------- ---------- ---------- ---------- -------GAC
CP013953.1 ---------- ---------- ---------- ---------- ---------- ---------- ---------- -------GAC
CP014064.1 ---------- ---------- ---------- ---------- ---------- ---------- ---------- -------GAC
LT009690.1 ---------- ---------- ---------- ---------- ---------- ---------- ---------- -------GAC
AP017320.1 ---------- ---------- ---------- ---------- ---------- ---------- ---------- -------GAC
CP010890.1 ---------- ---------- ---------- ---------- ---------- ---------- ---------- -------GAC
CP001844.2 ---------- ---------- ---------- ---------- ---------- ---------- ---------- -------GAC
CP001781.1 ---------- ---------- ---------- ---------- ---------- ---------- ---------- -------GAC
AB488501.1 ---------- ---------- ---------- ---------- ---------- ---------- ---------- -------GAC
AB489898.1 ---------- ---------- ---------- ---------- ---------- ---------- ---------- -------GAC
HE579073.1 ---------- ---------- ---------- ---------- ---------- ---------- ---------- -------GAC
HE579071.1 ---------- ---------- ---------- ---------- ---------- ---------- ---------- -------GAC
HE579069.1 ---------- ---------- ---------- ---------- ---------- ---------- ---------- -------GAC
HE579065.1 ---------- ---------- ---------- ---------- ---------- ---------- ---------- -------GAC
HE579063.1 ---------- ---------- ---------- ---------- ---------- ---------- ---------- -------GAC

2,400

CP007670.1	ATACAAGAAG	CCAAGTAA-A	1934
CP003033.1	ATACAAGAAG	CCAAGTAA-A	1934
CP018205.1	ATACAAGAAG	CCAAGTAA-A	1934
AP017377.1	ATACAAGAAG	CCAAGTAA-A	1934
LT598688.1	ATACAAGAAG	CCAAGTAA-A	1934
CP007676.1	ATACAAGAAG	CCAAGTAA-A	1934
CP007672.1	ATACAAGAAG	CCAAGTAA-A	1934
CP007674.1	ATACAAGAAG	CCAAGTAA-A	1934
CP011526.1	ATACAAGAAG	CCAAGTAA-A	1934
HF937103.1	ATACAAGAAG	CCAAGTAA-A	1934
AP009351.1	ATACAAGAAG	CCAAGTAA-A	1934
CP000046.1	ATACAAGAAG	CCAAGTAA-A	1934
CP000253.1	ATACAAGAAG	CCAAGTAA-A	1934
AC025591.8	ATACAAGAAG	CCAAGTAA-A	1934
LT671859.1	ATACAAGAAG	CCAAGTAA-A	1934
CP007499.1	ATACAAGAAG	CCAAGTAA-A	1934
X17679.1	ATACAAGAAG	CCAAGTAA-A	1934
CP007657.1	ATACAAGAAG	CCAAGTAA-A	1853
CP014444.1	ATACAAGAAG	CCAAGTAA-A	1853
CP014441.1	ATACAAGAAG	CCAAGTAA-A	1853
CP014438.1	ATACAAGAAG	CCAAGTAA-A	1853
CP014435.1	ATACAAGAAG	CCAAGTAA-A	1853
CP014432.1	ATACAAGAAG	CCAAGTAA-A	1853
CP014429.1	ATACAAGAAG	CCAAGTAA-A	1853
CP014426.1	ATACAAGAAG	CCAAGTAA-A	1853
CP014423.1	ATACAAGAAG	CCAAGTAA-A	1853
CP014420.1	ATACAAGAAG	CCAAGTAA-A	1853
CP014415.1	ATACAAGAAG	CCAAGTAA-A	1853
CP014412.1	ATACAAGAAG	CCAAGTAA-A	1853
CP014409.1	ATACAAGAAG	CCAAGTAA-A	1853
CP014407.1	ATACAAGAAG	CCAAGTAA-A	1853
CP014402.1	ATACAAGAAG	CCAAGTAA-A	1853
CP014397.1	ATACAAGAAG	CCAAGTAA-A	1853
CP014392.1	ATACAAGAAG	CCAAGTAA-A	1853
CP014387.1	ATACAAGAAG	CCAAGTAA-A	1853
CP014384.1	ATACAAGAAG	CCAAGTAA-A	1853
CP014381.1	ATACAAGAAG	CCAAGTAA-A	1853
CP014371.1	ATACAAGAAG	CCAAGTAA-A	1853
CP014368.1	ATACAAGAAG	CCAAGTAA-A	1853
CP014365.1	ATACAAGAAG	CCAAGTAA-A	1853
CP014362.1	ATACAAGAAG	CCAAGTAA-A	1853
CP014376.1	ATACAAGAAG	CCAAGTAA-A	1853
CP009423.1	ATACAAGAAG	CCAAGTAA-A	1853
CP016855.1	ATACAAGAAG	CCAAGTAA-A	1853
CP013231.1	ATACAAGAAG	CCAAGTAA-A	1853
CP010300.1	ATACAAGAAG	CCAAGTAA-A	1853
CP010299.1	ATACAAGAAG	CCAAGTAA-A	1853
CP010298.1	ATACAAGAAG	CCAAGTAA-A	1853
CP010297.1	ATACAAGAAG	CCAAGTAA-A	1853
CP010296.1	ATACAAGAAG	CCAAGTAA-A	1853
CP010295.1	ATACAAGAAG	CCAAGTAA-A	1853
CP007690.1	ATACAAGAAG	CCAAGTAA-A	1853
CP007176.1	ATACAAGAAG	CCAAGTAA-A	1853
CP000730.1	ATACAAGAAG	CCAAGTAA-A	1853
CP000255.1	ATACAAGAAG	CCAAGTAA-A	1853
CP007539.1	ATACAAGAAG	CCAAGTAA-A	1933
CP007657.1	ATACAAGAAG	CCAAGTAA-A	1853
AP014921.1	ATACAAGAAG	CCAAGTAA-A	1853
AB436955.1	ATACAAGAAG	CCAAGTAA-A	1842
AB489885.1	ATACAAGAAG	CCAAGTAA-A	1842
AB489873.1	ATACAAGAAG	CCAAGTAA-A	1842
AB489883.1	ATACAAGAAG	CCAAGTAA-A	1761
AB489874.1	ATACAAGAAG	CCAAGTAA-A	1749
LT615218.1	ACAAAAAAAG	CCAAGCAA-A	2096
AB436976.1	ACAAAAAAAG	CCAAGCAA-A	2096
AB489892.1	ACAAAAAAAG	CCAAGCAA-A	2004
AJ306908.1	ACAAAACAAG	CCAAGTAA-A	2096
CP015646.1	ACAAAACAAG	CCAAGTAA-A	2096
AB488510.1	----------	----------	1568
EU105387.1	----------	----------	1520
AB436975.1	ACAAAACAAA	CCAAGTAA-A	2096
AB488499.1	ACAAAACAAA	CCAAGTAA-A	2004
AJ309189.1	----------	----------	1386
FR821779.1	ATACAAGAAG	CCAAGCGA-A	2081
CP012593.1	ACAAAAAAAG	CCAAGCGA-A	1444
CP012692.1	ACAAAAAAAG	CCAAGCGA-A	1444
CP013955.1	ACAAAAAAAG	CCAAGCGA-A	1444
CP013953.1	ACAAAAAAAG	CCAAGCGA-A	1444
CP014064.1	ACAAAAAAAG	CCAAGCGA-A	1444
LT009690.1	ACAAAAAAAG	CCAAGCGA-A	1444
AP017320.1	ACAAAAAAAG	CCAAGCGA-A	1444
CP010890.1	ACAAAAAAAG	CCAAGCGA-A	1444
CP001844.2	ACAAAAAAAG	CCAAGCGA-A	1444
CP001781.1	ACAAAAAAAG	CCAAGCGA-A	1444
AB488501.1	ACAAAAAAAG	CCAAGCGA-A	1444
AB489898.1	ACAAAAAAAG	CCAAGCGA-A	1444
HE579073.1	ACAAAAAAAG	CCAAGCGA-A	1444
HE579071.1	ACAAAAAAAG	CCAAGCGA-A	1444
HE579069.1	ACAAAAAAAG	CCAAGCGA-A	1444
HE579065.1	ACAAAAAAAG	CCAAGCGA-A	1444
HE579063.1	ACAAAAAAAG	CCAAGCGA-A	1444

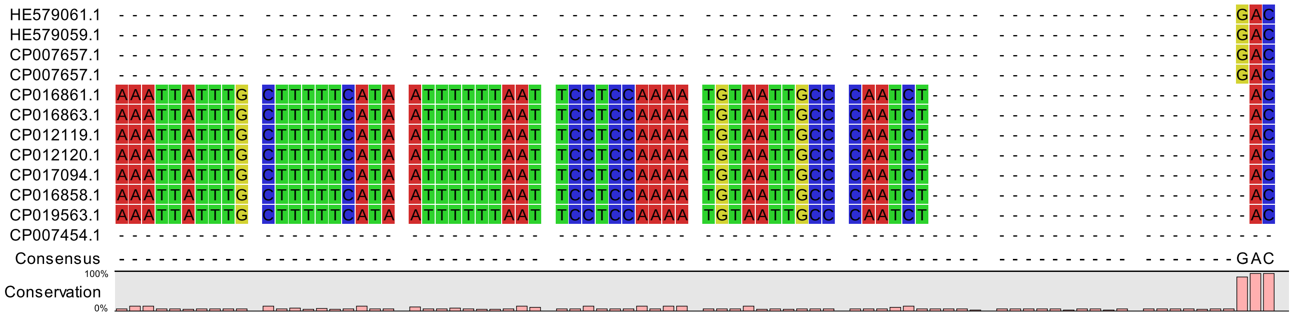
HE579061.1 ---------- ---------- ---------- ---------- ---------- ---------- ---------- -------GAC
HE579059.1 ---------- ---------- ---------- ---------- ---------- ---------- ---------- -------GAC
CP007657.1 ---------- ---------- ---------- ---------- ---------- ---------- ---------- -------GAC
CP007657.1 ---------- ---------- ---------- ---------- ---------- ---------- ---------- -------GAC
CP016861.1 AAATTATTTG CTTTTTCATA ATTTTTTAAT TCCTCCAAAA TGTAATTGCC CAATCT---- ---------- --------AC
CP016863.1 AAATTATTTG CTTTTTCATA ATTTTTTAAT TCCTCCAAAA TGTAATTGCC CAATCT---- ---------- --------AC
CP012119.1 AAATTATTTG CTTTTTCATA ATTTTTTAAT TCCTCCAAAA TGTAATTGCC CAATCT---- ---------- --------AC
CP012120.1 AAATTATTTG CTTTTTCATA ATTTTTTAAT TCCTCCAAAA TGTAATTGCC CAATCT---- ---------- --------AC
CP017094.1 AAATTATTTG CTTTTTCATA ATTTTTTAAT TCCTCCAAAA TGTAATTGCC CAATCT---- ---------- --------AC
CP016858.1 AAATTATTTG CTTTTTCATA ATTTTTTAAT TCCTCCAAAA TGTAATTGCC CAATCT---- ---------- --------AC
CP019563.1 AAATTATTTG CTTTTTCATA ATTTTTTAAT TCCTCCAAAA TGTAATTGCC CAATCT---- ---------- --------AC
CP007454.1 ---------- ---------- ---------- ---------- ---------- ---------- ---------- ----------
Consensus ---------- ---------- ---------- ---------- ---------- ---------- ---------- -------GAC
100%
Conservation
0%

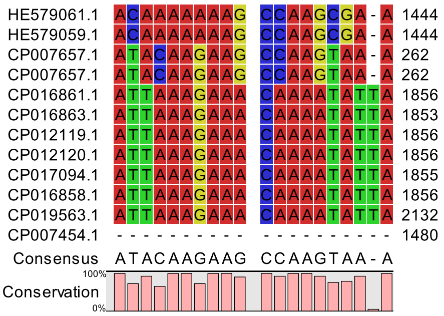
HE579061.1 ACAAAAAAAG CCAAGCGA-A 1444
HE579059.1 ACAAAAAAAG CCAAGCGA-A 1444
CP007657.1 ATACAAGAAG CCAAGTAA-A 262
CP007657.1 ATACAAGAAG CCAAGTAA-A 262
CP016861.1 ATTAAAGAAA CAAAATATTA 1856
CP016863.1 ATTAAAGAAA CAAAATATTA 1853
CP012119.1 ATTAAAGAAA CAAAATATTA 1856
CP012120.1 ATTAAAGAAA CAAAATATTA 1856
CP017094.1 ATTAAAGAAA CAAAATATTA 1855
CP016858.1 ATTAAAGAAA CAAAATATTA 1856
CP019563.1 ATTAAAGAAA CAAAATATTA 2132
CP007454.1 ---------- ---------- 1480
Consensus ATACAAGAAG CCAAGTAA-A
100%
Conservation
0%

```
                    2,420                    2,440                    2,460
CP007670.1  ACAAATGCAT ACAATGTAAC AACACATGCA ---------- ---------- ---------- --------- 1964
CP003033.1  ACAAATGCAT ACAATGTAAC AACACATGCA ---------- ---------- ---------- --------- 1964
CP018205.1  ACAAATGCAT ACAATGTAAC AACACATGCA ---------- ---------- ---------- --------- 1964
AP017377.1  ACAAATGCAT ACAATGTAAC AACACATGCA ---------- ---------- ---------- --------- 1964
LT598688.1  ACAAATGCAT ACAATGTAAC AACACATGCA ---------- ---------- ---------- --------- 1964
CP007676.1  ACAAATGCAT ACAATGTAAC AACACATGCA ---------- ---------- ---------- --------- 1964
CP007672.1  ACAAATGCAT ACAATGTAAC AACACATGCA ---------- ---------- ---------- --------- 1964
CP007674.1  ACAAATGCAT ACAATGTAAC AACACATGCA ---------- ---------- ---------- --------- 1964
CP011526.1  ACAAATGCAT ACAATGTAAC AACACATGCA ---------- ---------- ---------- --------- 1964
HF937103.1  ACAAATGCAT ACAATGTAAC AACACATGCA ---------- ---------- ---------- --------- 1964
AP009351.1  ACAAATGCAT ACAATGTAAC AACACATGCA ---------- ---------- ---------- --------- 1964
CP000046.1  ACAAATGCAT ACAATGTAAC AACACATGCA ---------- ---------- ---------- --------- 1964
CP000253.1  ACAAATGCAT ACAATGTAAC AACACATGCA ---------- ---------- ---------- --------- 1964
AC025591.8  ACAAATGCAT ACAATGTAAC AACACATGCA ---------- ---------- ---------- --------- 1964
LT671859.1  ACAAATGCAT ACAATGTAAC AACACATGCA ---------- ---------- ---------- --------- 1964
CP007499.1  ACAAATGCAT ACAATGTAAC AACACATGCA ---------- ---------- ---------- --------- 1964
X17679.1    ACAAATGCAT ACAATGTAAC AACACATGCA ---------- ---------- ---------- --------- 1964
CP007657.1  ACAAATGCAT ACAATGTAAC AACACATGCA ---------- ---------- ---------- --------- 1883
CP014444.1  ACAAATGCAT ACAATGTAAC AACACATGCA ---------- ---------- ---------- --------- 1883
CP014441.1  ACAAATGCAT ACAATGTAAC AACACATGCA ---------- ---------- ---------- --------- 1883
CP014438.1  ACAAATGCAT ACAATGTAAC AACACATGCA ---------- ---------- ---------- --------- 1883
CP014435.1  ACAAATGCAT ACAATGTAAC AACACATGCA ---------- ---------- ---------- --------- 1883
CP014432.1  ACAAATGCAT ACAATGTAAC AACACATGCA ---------- ---------- ---------- --------- 1883
CP014429.1  ACAAATGCAT ACAATGTAAC AACACATGCA ---------- ---------- ---------- --------- 1883
CP014426.1  ACAAATGCAT ACAATGTAAC AACACATGCA ---------- ---------- ---------- --------- 1883
CP014423.1  ACAAATGCAT ACAATGTAAC AACACATGCA ---------- ---------- ---------- --------- 1883
CP014420.1  ACAAATGCAT ACAATGTAAC AACACATGCA ---------- ---------- ---------- --------- 1883
CP014415.1  ACAAATGCAT ACAATGTAAC AACACATGCA ---------- ---------- ---------- --------- 1883
CP014412.1  ACAAATGCAT ACAATGTAAC AACACATGCA ---------- ---------- ---------- --------- 1883
CP014409.1  ACAAATGCAT ACAATGTAAC AACACATGCA ---------- ---------- ---------- --------- 1883
CP014407.1  ACAAATGCAT ACAATGTAAC AACACATGCA ---------- ---------- ---------- --------- 1883
CP014402.1  ACAAATGCAT ACAATGTAAC AACACATGCA ---------- ---------- ---------- --------- 1883
CP014397.1  ACAAATGCAT ACAATGTAAC AACACATGCA ---------- ---------- ---------- --------- 1883
CP014392.1  ACAAATGCAT ACAATGTAAC AACACATGCA ---------- ---------- ---------- --------- 1883
CP014387.1  ACAAATGCAT ACAATGTAAC AACACATGCA ---------- ---------- ---------- --------- 1883
CP014384.1  ACAAATGCAT ACAATGTAAC AACACATGCA ---------- ---------- ---------- --------- 1883
CP014381.1  ACAAATGCAT ACAATGTAAC AACACATGCA ---------- ---------- ---------- --------- 1883
CP014371.1  ACAAATGCAT ACAATGTAAC AACACATGCA ---------- ---------- ---------- --------- 1883
CP014368.1  ACAAATGCAT ACAATGTAAC AACACATGCA ---------- ---------- ---------- --------- 1883
CP014365.1  ACAAATGCAT ACAATGTAAC AACACATGCA ---------- ---------- ---------- --------- 1883
CP014362.1  ACAAATGCAT ACAATGTAAC AACACATGCA ---------- ---------- ---------- --------- 1883
CP014376.1  ACAAATGCAT ACAATGTAAC AACACATGCA ---------- ---------- ---------- --------- 1883
CP009423.1  ACAAATGCAT ACAATGTAAC AACACATGCA ---------- ---------- ---------- --------- 1883
CP016855.1  ACAAATGCAT ACAATGTAAC AACACATGCA ---------- ---------- ---------- --------- 1883
CP013231.1  ACAAATGCAT ACAATGTAAC AACACATGCA ---------- ---------- ---------- --------- 1883
CP010300.1  ACAAATGCAT ACAATGTAAC AACACATGCA ---------- ---------- ---------- --------- 1883
CP010299.1  ACAAATGCAT ACAATGTAAC AACACATGCA ---------- ---------- ---------- --------- 1883
CP010298.1  ACAAATGCAT ACAATGTAAC AACACATGCA ---------- ---------- ---------- --------- 1883
CP010297.1  ACAAATGCAT ACAATGTAAC AACACATGCA ---------- ---------- ---------- --------- 1883
CP010296.1  ACAAATGCAT ACAATGTAAC AACACATGCA ---------- ---------- ---------- --------- 1883
CP010295.1  ACAAATGCAT ACAATGTAAC AACACATGCA ---------- ---------- ---------- --------- 1883
CP007690.1  ACAAATGCAT ACAATGTAAC AACACATGCA ---------- ---------- ---------- --------- 1883
CP007176.1  ACAAATGCAT ACAATGTAAC AACACATGCA ---------- ---------- ---------- --------- 1883
CP000730.1  ACAAATGCAT ACAATGTAAC AACACATGCA ---------- ---------- ---------- --------- 1883
CP000255.1  ACAAATGCAT ACAATGTAAC AACACATGCA ---------- ---------- ---------- --------- 1883
CP007539.1  ACAAATGCAT ACAATGTAAC AACACATGCA ---------- ---------- ---------- --------- 1963
CP007657.1  ACAAATGCAT ACAATGTAAC AACACATGCA ---------- ---------- ---------- --------- 1883
AP014921.1  ACAAATGCAT ACAATGTAAC AACACATGCA ---------- ---------- ---------- --------- 1883
AB436955.1  ACAAATGCAT ACAATGTAAC AACACATGCA ---------- ---------- ---------- --------- 1872
AB489885.1  ACAAATGCAT ACAATGTAAC AACACATGCA ---------- ---------- ---------- --------- 1872
AB489873.1  ACAAATGCAT ACAATGTAAC AACACATGCA ---------- ---------- ---------- --------- 1872
AB489883.1  ACAAATGCAT ACAATGTAAC AACACATGCA ---------- ---------- ---------- --------- 1791
AB489874.1  ACAAATGCAT ACAATGTAAC AACACATGCA ---------- ---------- ---------- --------- 1779
LT615218.1  ACAAATGCAT ATAACGTAAC AACACATGCA GATGGTACTG CGACATATGG GCCTAGAGTA ACAAAATAA 2165
AB436976.1  ACAAATGCAT ATAACGTAAC AACACATGCA GATGGTACTG CGACATATGG GCCTAGAGTA ACAAAATAA 2165
AB489892.1  ACAAATGCAT ATAACGTAAC AACACATGCA GATGGTACTG CGACATATGG GCCTAGAGTA ACAAAATAA 2073
AJ306908.1  ACAAACGCAT ATAACGTAAC AACACATGCA GATGGTACTG CGACATATGG GCCTAGAGTA ACAAAATAA 2165
CP015646.1  ACAAACGCAT ATAACGTAAC AACACATGCA GATGGTACTG CGACATATGG TCCTAGAGTA ACAAAATAA 2165
AB488510.1  ---------- ---------- ---------- ---------- ---------- ---------- --------- 1568
EU105387.1  ---------- ---------- ---------- ---------- ---------- ---------- --------- 1520
AB436975.1  ACAAGTGCAT ATAACGTAAC AACACATGCA ---------- ---------- ---------- --------- 2126
AB488499.1  ACAAGTGCAT ATAACGTAAC AACACATGCA ---------- ---------- ---------- --------- 2034
AJ309189.1  ---------- ---------- ---------- ---------- ---------- ---------- --------- 1386
FR821779.1  ACAAATGCAT ATAACGTAAC AACACATGCA GATGGTACTG CGACATATGG TCCTAGAGTA ACAAAATAA 2150
CP012593.1  ACAAACGCAT ATAACGTAAC AACACATGCA GATGGT---- ---------- ---------- --------- 1480
CP012692.1  ACAAACGCAT ATAACGTAAC AACACATGCA GATGGT---- ---------- ---------- --------- 1480
CP013955.1  ACAAACGCAT ATAACGTAAC AACACATGCA GATGGT---- ---------- ---------- --------- 1480
CP013953.1  ACAAACGCAT ATAACGTAAC AACACATGCA GATGGT---- ---------- ---------- --------- 1480
CP014064.1  ACAAACGCAT ATAACGTAAC AACACATGCA GATGGT---- ---------- ---------- --------- 1480
LT009690.1  ACAAACGCAT ATAACGTAAC AACACATGCA GATGGT---- ---------- ---------- --------- 1480
AP017320.1  ACAAACGCAT ATAACGTAAC AACACATGCA GATGGT---- ---------- ---------- --------- 1480
CP010890.1  ACAAACGCAT ATAACGTAAC AACACATGCA GATGGT---- ---------- ---------- --------- 1480
CP001844.2  ACAAACGCAT ATAACGTAAC AACACATGCA GATGGT---- ---------- ---------- --------- 1480
CP001781.1  ACAAACGCAT ATAACGTAAC AACACATGCA GATGGT---- ---------- ---------- --------- 1480
AB488501.1  ACAAACGCAT ATAACGTAAC AACACATGCA GATGGT---- ---------- ---------- --------- 1480
AB489898.1  ACAAACGCAT ATAACGTAAC AACACATGCA GATGGT---- ---------- ---------- --------- 1480
HE579073.1  ACAAACGCAT ATAACGTAAC AACACATGCA GATGGT---- ---------- ---------- --------- 1480
HE579071.1  ACAAACGCAT ATAACGTAAC AACACATGCA GATGGT---- ---------- ---------- --------- 1480
HE579069.1  ACAAACGCAT ATAACGTAAC AACACATGCA GATGGT---- ---------- ---------- --------- 1480
HE579065.1  ACAAACGCAT ATAACGTAAC AACACATGCA GATGGT---- ---------- ---------- --------- 1480
HE579063.1  ACAAACGCAT ATAACGTAAC AACACATGCA GATGGT---- ---------- ---------- --------- 1480
```

CP007670.1
CP003033.1
CP018205.1
AP017377.1
LT598688.1
CP007676.1
CP007672.1
CP007674.1
CP011526.1
HF937103.1
AP009351.1
CP000046.1
CP000253.1
AC025591.8
LT671859.1
CP007499.1
X17679.1
CP007657.1
CP014444.1
CP014441.1
CP014438.1
CP014435.1
CP014432.1
CP014429.1
CP014426.1
CP014423.1
CP014420.1
CP014415.1
CP014412.1
CP014409.1
CP014407.1
CP014402.1
CP014397.1
CP014392.1
CP014387.1
CP014384.1
CP014381.1
CP014371.1
CP014368.1
CP014365.1
CP014362.1
CP014376.1
CP009423.1
CP016855.1
CP013231.1
CP010300.1
CP010299.1
CP010298.1
CP010297.1
CP010296.1
CP010295.1
CP007690.1
CP007176.1
CP000730.1
CP000255.1
CP007539.1
CP007657.1
AP014921.1
AB436955.1
AB489885.1
AB489873.1
AB489883.1
AB489874.1
LT615218.1
AB436976.1
AB489892.1
AJ306908.1
CP015646.1
AB488510.1
EU105387.1
AB436975.1
AB488499.1
AJ309189.1
FR821779.1
CP012593.1
CP012692.1
CP013955.1
CP013953.1
CP014064.1
LT009690.1
AP017320.1
CP010890.1
CP001844.2
CP001781.1
AB488501.1
AB489898.1
HE579073.1
HE579071.1
HE579069.1
HE579065.1
HE579063.1

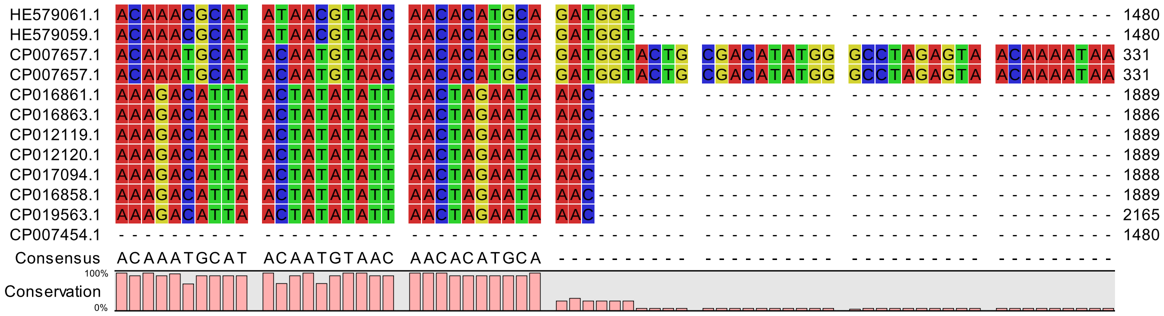
HE579061.1 ACAAACGCAT ATAACGTAAC AACACATGCA GATGGT---- ---------- ---------- --------- 1480
HE579059.1 ACAAACGCAT ATAACGTAAC AACACATGCA GATGGT---- ---------- ---------- --------- 1480
CP007657.1 ACAAATGCAT ACAATGTAAC AACACATGCA GATGGTACTG CGACATATGG GCCTAGAGTA ACAAAATAA 331
CP007657.1 ACAAATGCAT ACAATGTAAC AACACATGCA GATGGTACTG CGACATATGG GCCTAGAGTA ACAAAATAA 331
CP016861.1 AAAGACATTA ACTATATATT AACTAGAATA AAC------- ---------- ---------- --------- 1889
CP016863.1 AAAGACATTA ACTATATATT AACTAGAATA AAC------- ---------- ---------- --------- 1886
CP012119.1 AAAGACATTA ACTATATATT AACTAGAATA AAC------- ---------- ---------- --------- 1889
CP012120.1 AAAGACATTA ACTATATATT AACTAGAATA AAC------- ---------- ---------- --------- 1889
CP017094.1 AAAGACATTA ACTATATATT AACTAGAATA AAC------- ---------- ---------- --------- 1888
CP016858.1 AAAGACATTA ACTATATATT AACTAGAATA AAC------- ---------- ---------- --------- 1889
CP019563.1 AAAGACATTA ACTATATATT AACTAGAATA AAC------- ---------- ---------- --------- 2165
CP007454.1 ---------- ---------- ---------- ---------- ---------- ---------- --------- 1480
Consensus ACAAATGCAT ACAATGTAAC AACACATGCA ---------- ---------- ---------- ---------
100%
Conservation
0%

HE579061.1
HE579059.1
CP007657.1
CP007657.1
CP016861.1
CP016863.1
CP012119.1
CP012120.1
CP017094.1
CP016858.1
CP019563.1
CP007454.1
Consensus
100%
Conservation
0%
