# Supplementary figures and images for: Some coagulase negative Staphylococcus spp. isolated from buffalo can be misidentified as Staphylococcus aureus by phenotypic and Sa442 PCR methods
Source: BMC Res Notes. 2018 May 30;11:346. doi: 10.1186/s13104-018-3449-8 (PMC5977496; doi:10.1186/s13104-018-3449-8)

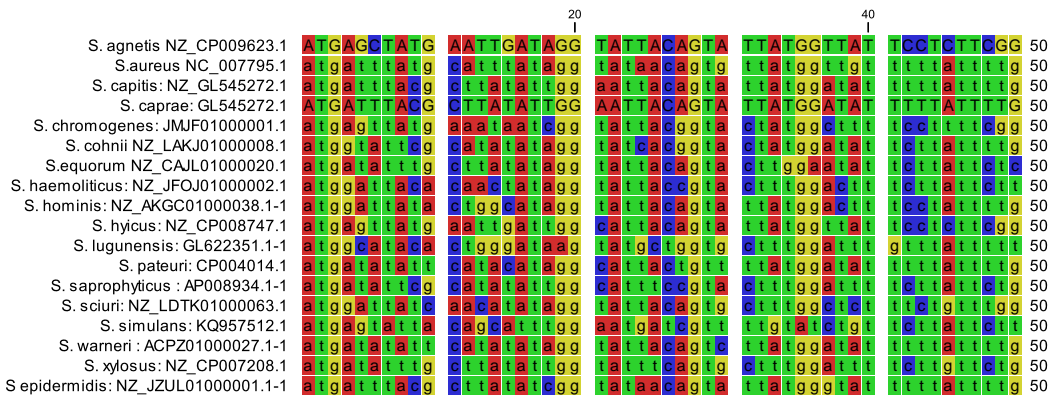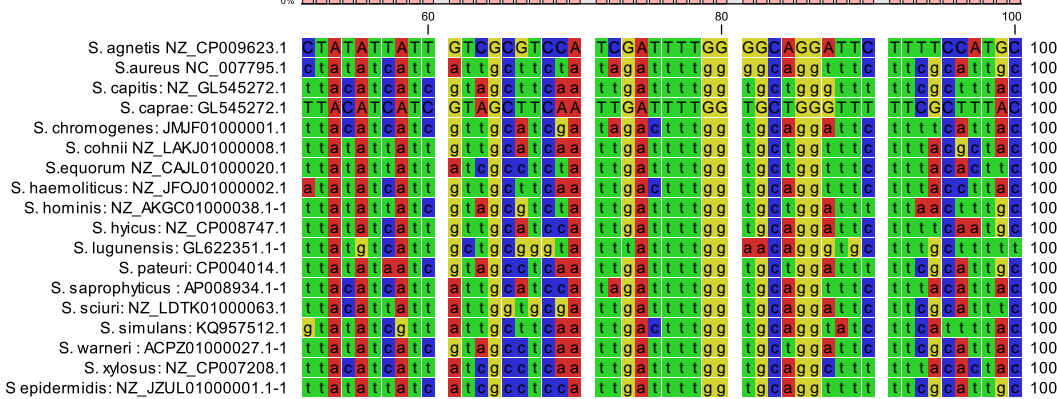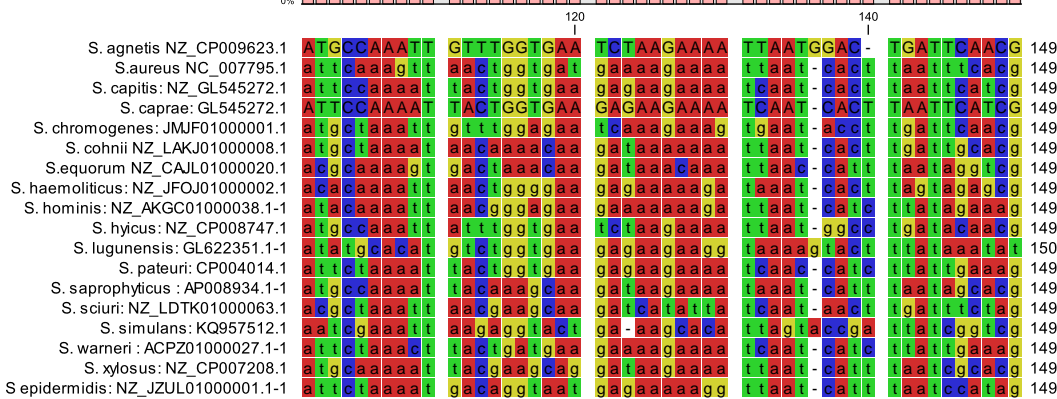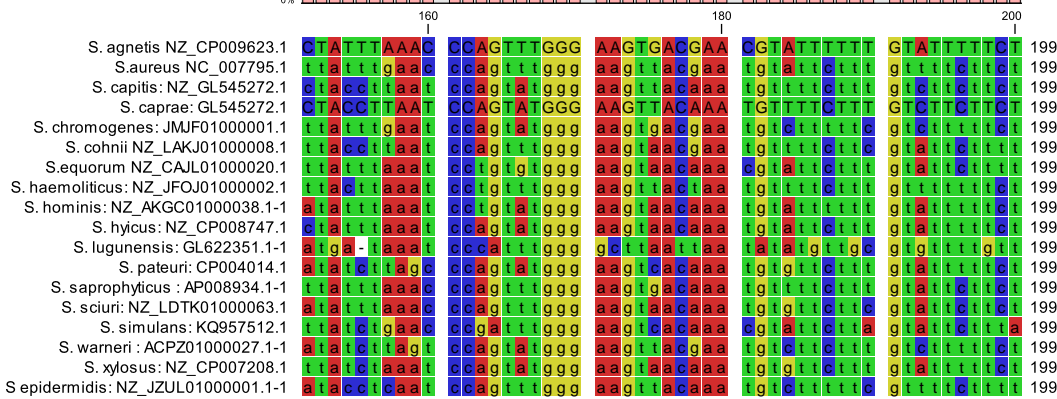

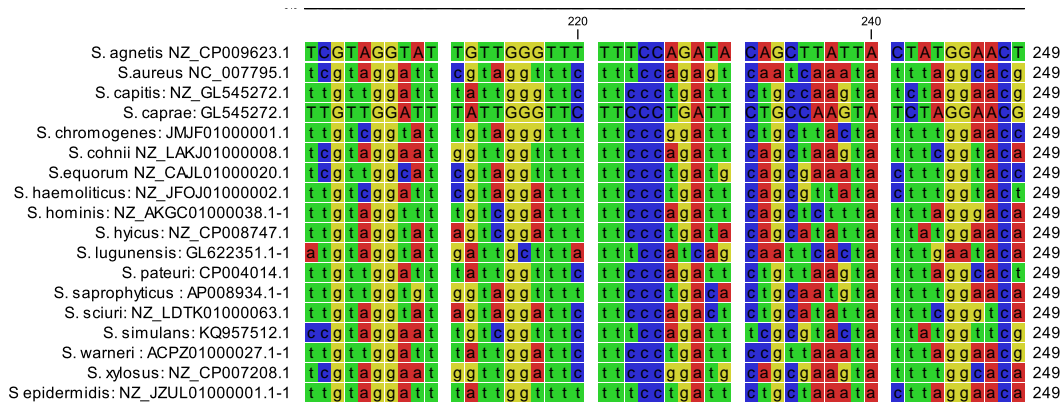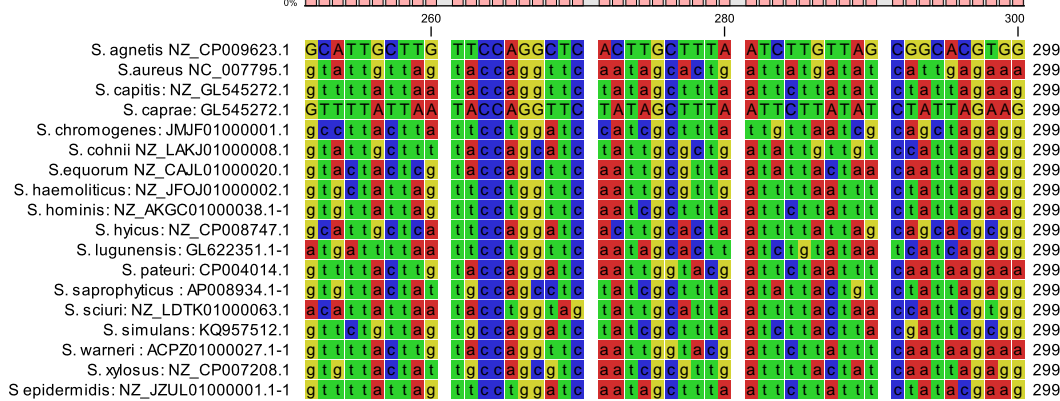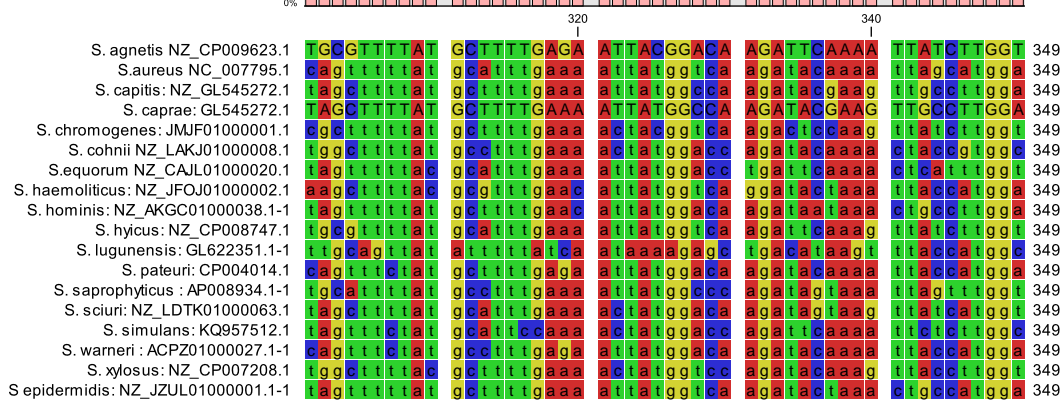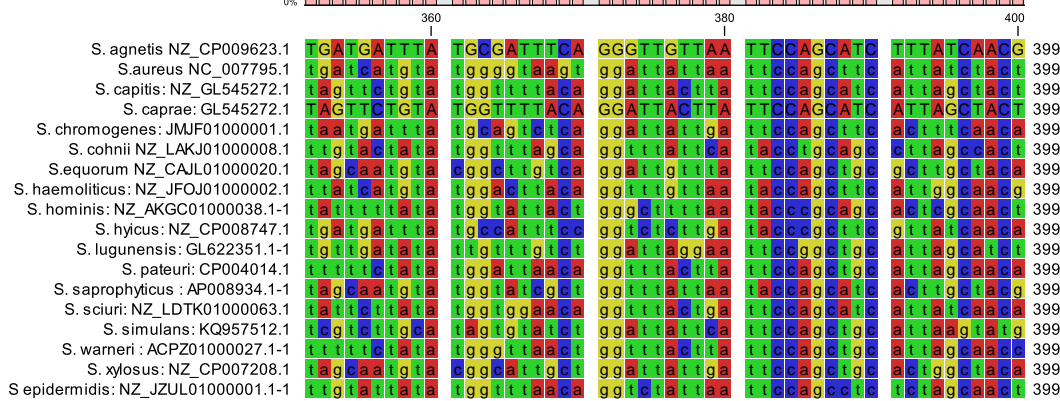

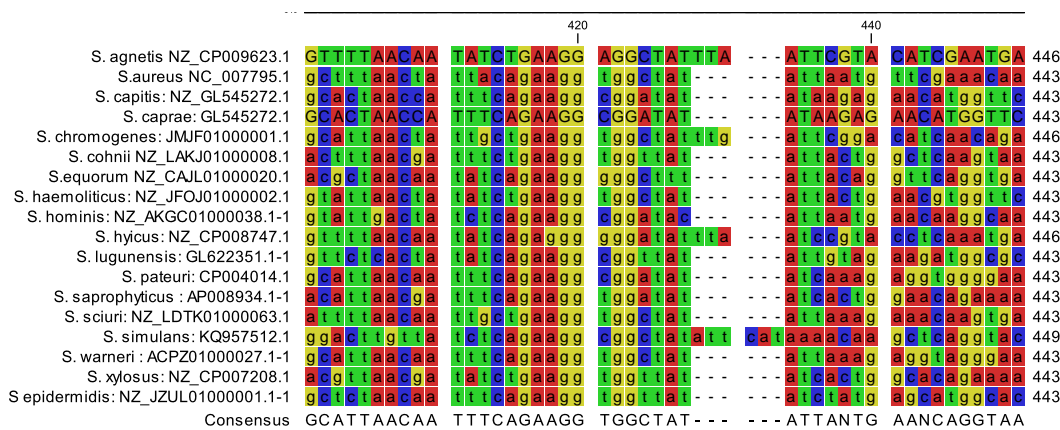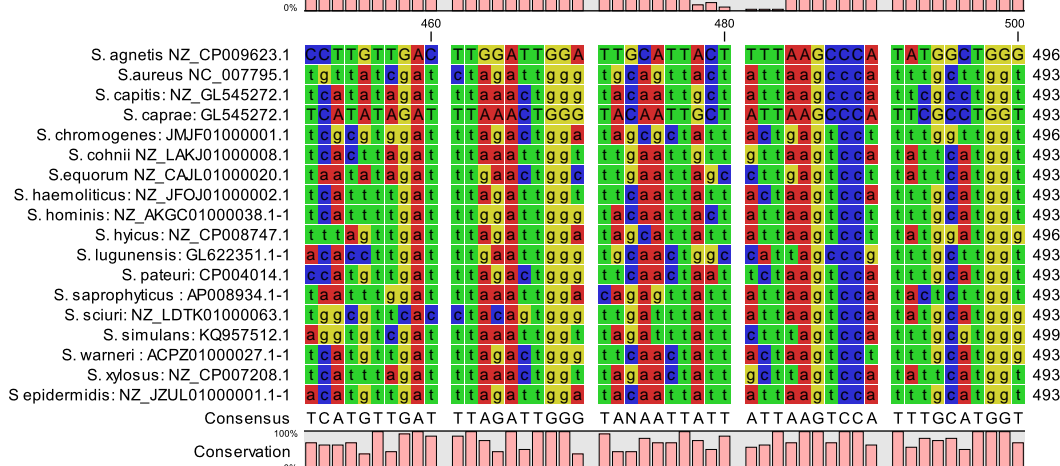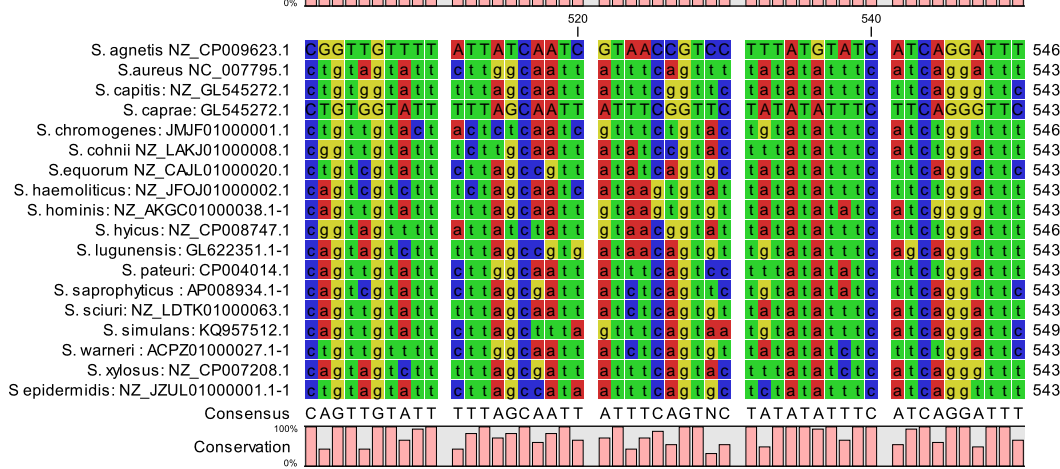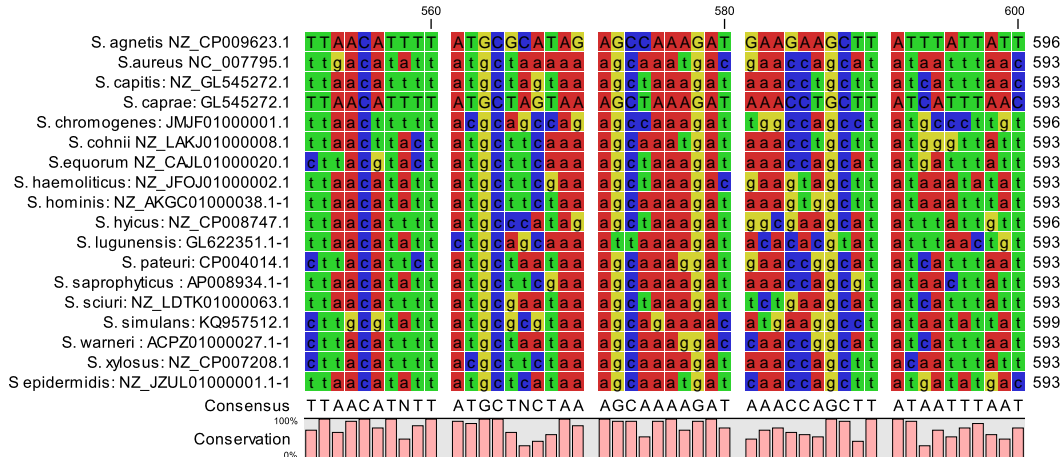

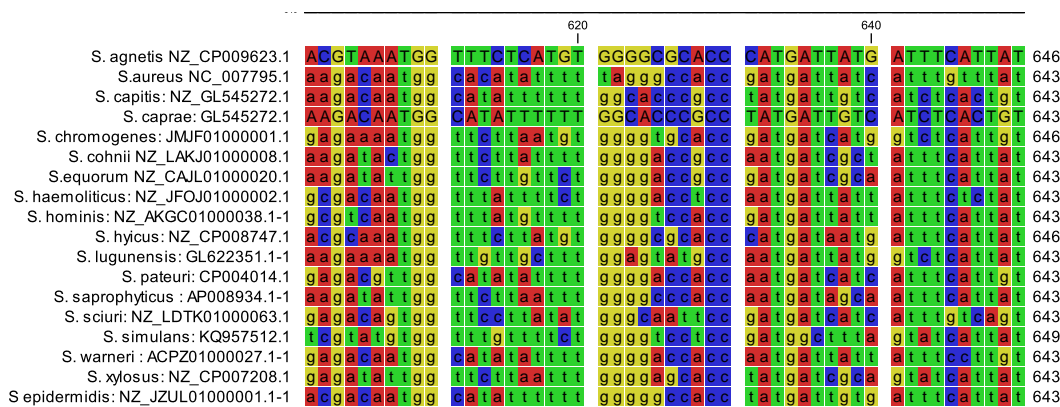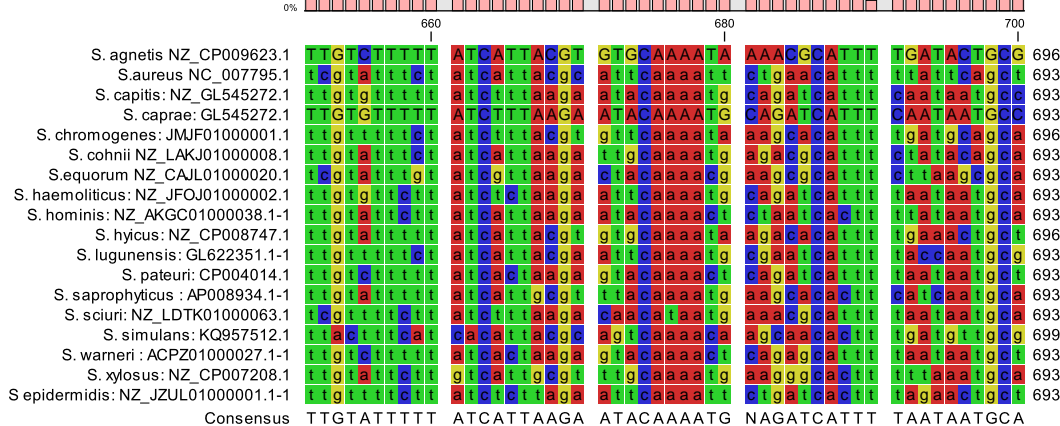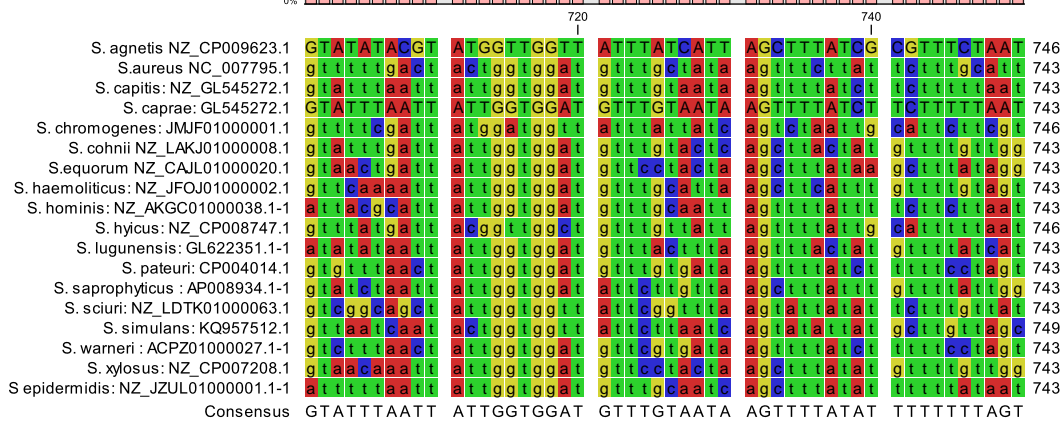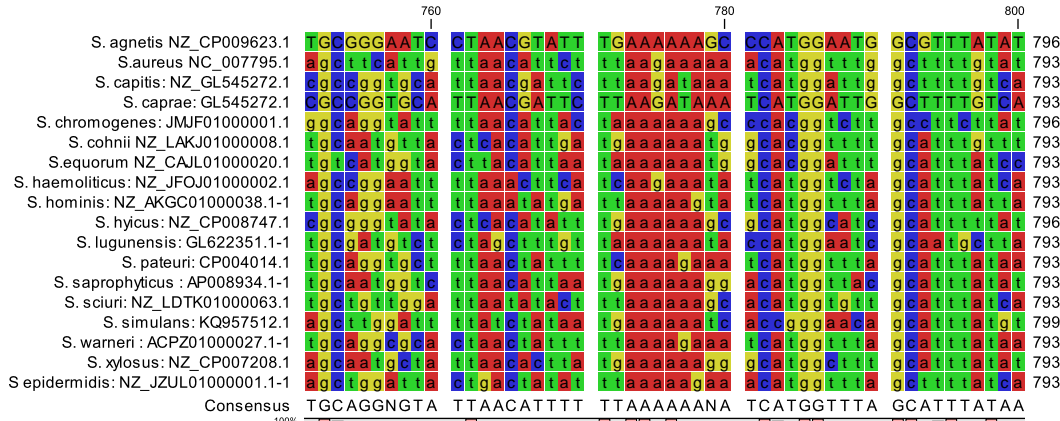

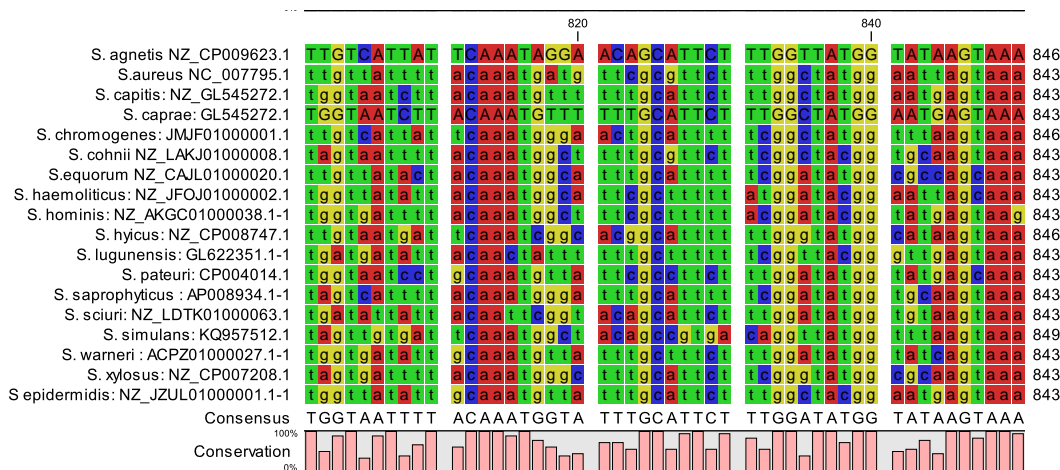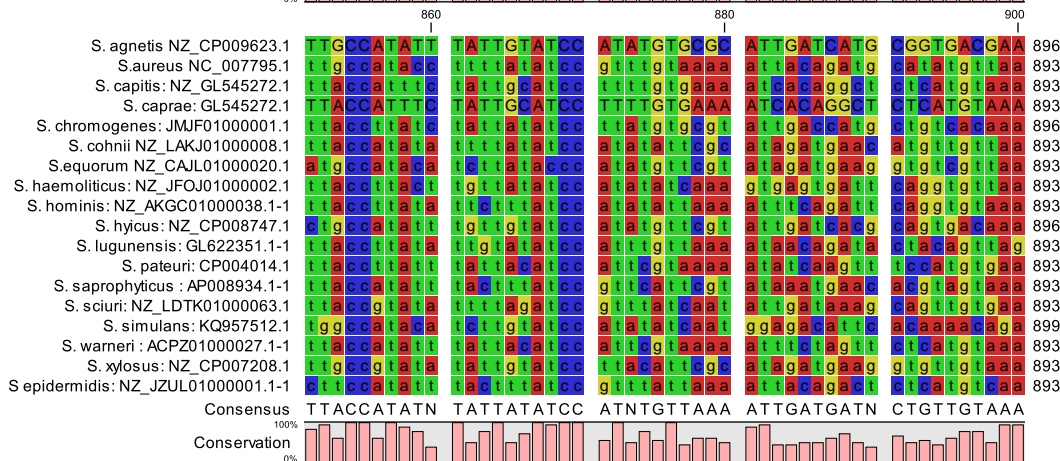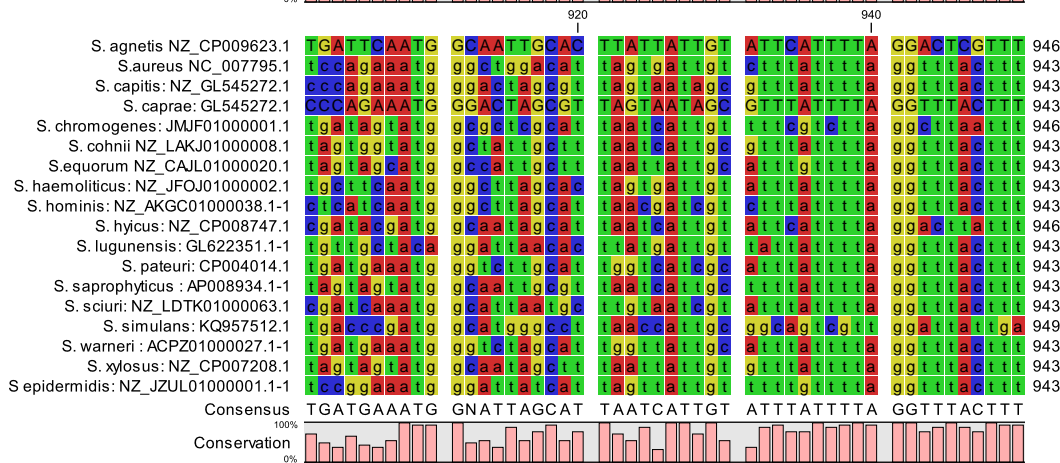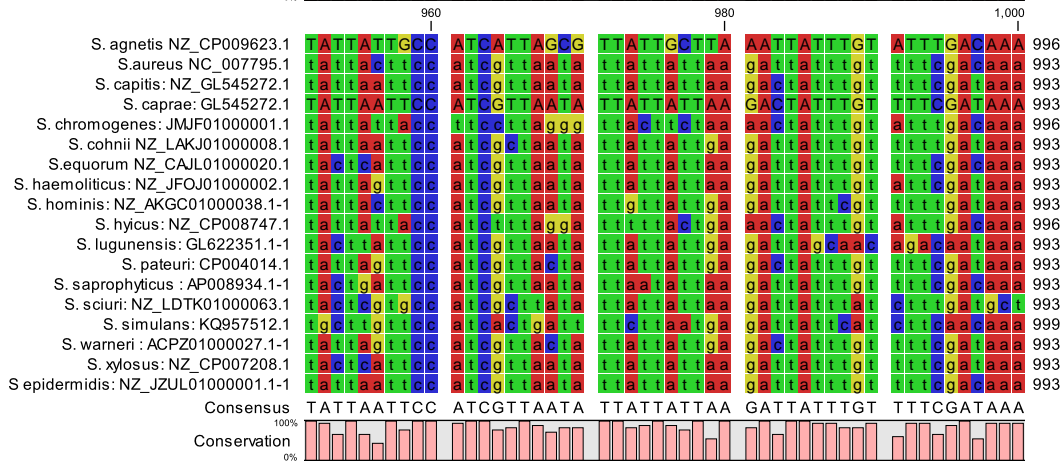

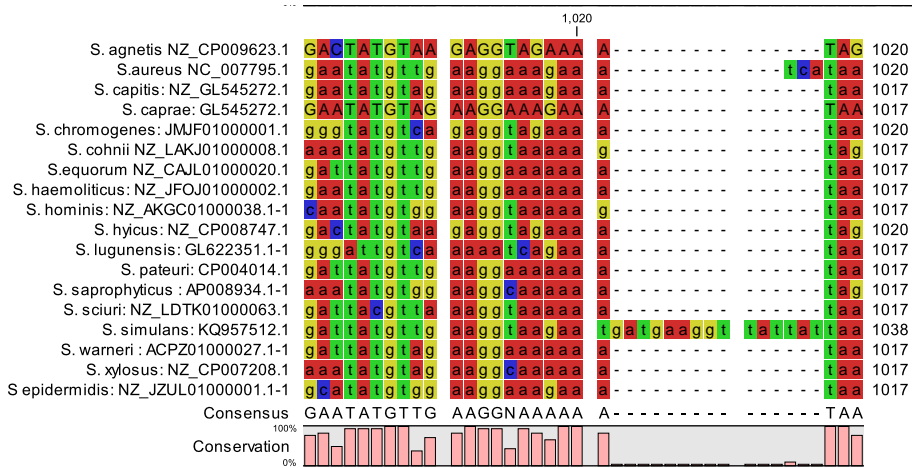

Supplement: Supplementary file 2 — Additional file 2: Fig. S2. Alignment using CLC Sequence View 7 software of the cydB gene of 18 closely related Staphylococcus spp. in this study. [file 13104_2018_3449_MOESM2_ESM.pdf]
